# Selective α-Methylation of Aryl Ketones Using Quaternary Ammonium Salts as Solid Methylating Agents

**DOI:** 10.1021/acs.joc.1c03158

**Published:** 2022-03-07

**Authors:** Johanna Templ, Michael Schnürch

**Affiliations:** Institute of Applied Synthetic Chemistry, TU Wien, Getreidemarkt 9/163, 1060 Wien, Austria

## Abstract

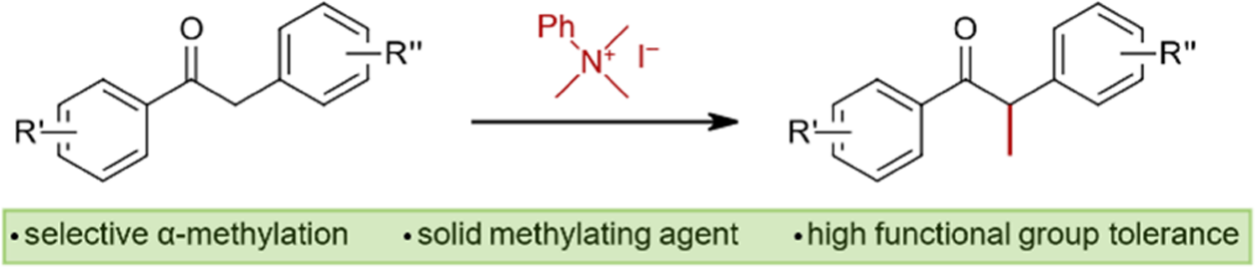

We describe the use of phenyl trimethylammonium iodide (PhMe_3_NI) as an alternative methylating agent for introducing a CH_3_ group in α-position to a carbonyl group. Compared to conventional methylating agents, quaternary ammonium salts have the advantages of being nonvolatile, noncancerogenic, and easy-to-handle solids. This regioselective method is characterized by ease of operational setup, use of anisole as green solvent, and yields up to 85%.

## Introduction

Incorporating a methyl group into small organic or bioactive molecules can positively affect their physical properties and biological effectiveness.^1,2^ The latter feature is commonly referred to as the “magic methyl effect”.^3^ This renders the methyl group a prevalent structural motif in small-molecule drugs.^4,5^ Owing to its considerable impact, a late-stage introduction of a CH_3_ group has become a particularly promising strategy in drug discovery.^6−8^ Hence, the development of efficient and new strategies for selective methylation attracts broad interest in medicinal chemistry and basic research, respectively.^9−13^

Traditionally applied methylating agents often suffer from inconvenient physical properties (*e.g.*, MeBr, b.p. 4 °C, MeI, b.p. 42 °C) or high toxicity (*e.g.*, MeI, Me_2_SO_4_). Several organometallic reagents used for methylation (*e.g.*, MeB(OH)_2_, Me_4_Sn, Me_3_Al, MeMgCl, or Me_2_Zn) are quite challenging to handle, as some are air-sensitive, show low functional group tolerance, or have to be freshly prepared.^14,15^ These toxicological and safety concerns encouraged us to search for a novel, safe, and easy-to-handle reagent for direct methylation. From previous findings in our group, we established different quaternary ammonium salts as alkylating agents in metal-catalyzed C–H activation reactions.^16,17^

**Figure 1:**
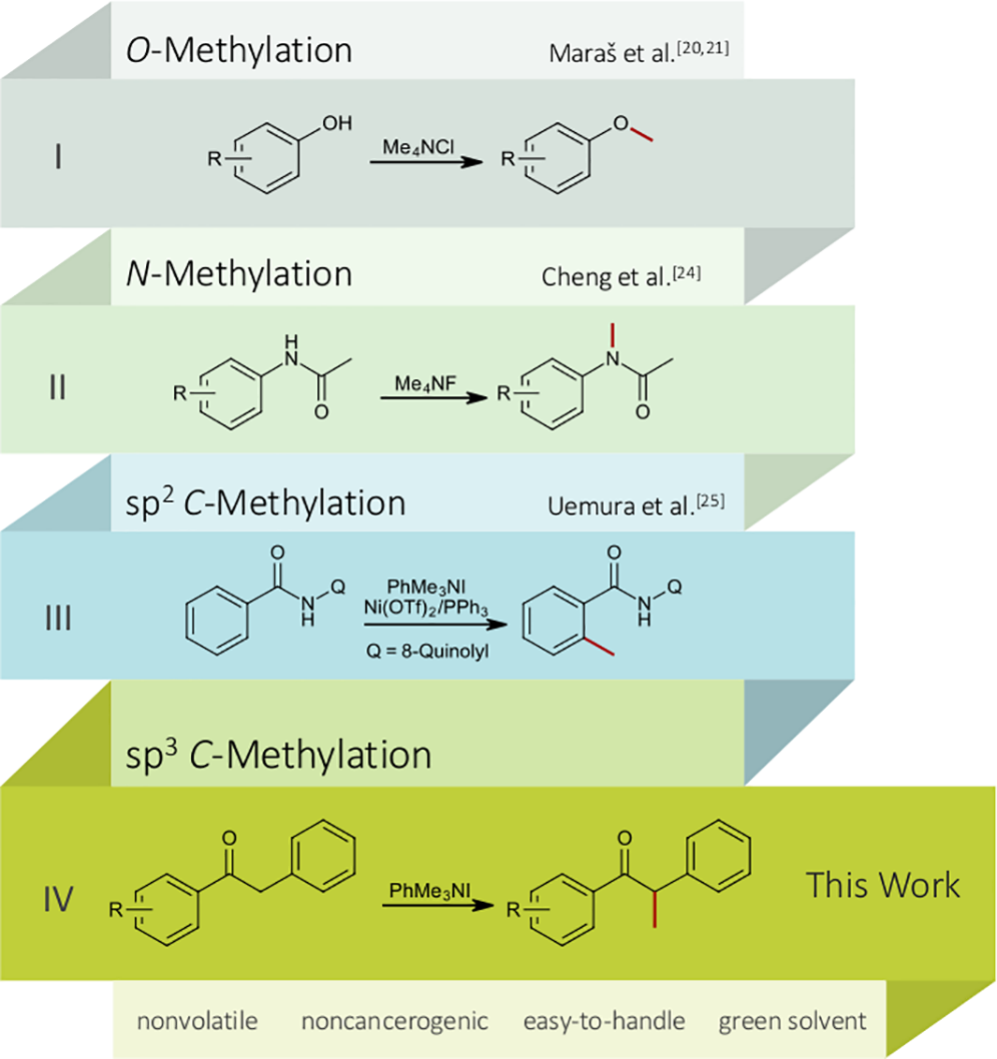
Methylation strategies using quaternary ammonium salts.

The predominant role of quaternary ammonium salts in organic reactions is their application as phase transfer catalysts^18^ and ionic liquids.^19^ However, their use as alkylating agents in general and methylating agents in particular is quite an unexplored field. There are a few reports on *O*-methylation of phenolic compounds with tetramethylammonium chloride (Me_4_NCl, Figure 1, I)^20,21^ or hydroxide (Me_4_NOH)^22^ and phenyl trimethylammonium (PhMe_3_NCl) chloride.^21^*N*-Methylation *via* ammonium salts was achieved in azaheterocycles using tetramethylammonium bromide (Me_4_NBr)^23^ and more recently in amides, N-heterocycles, alcohols, and thiols using tetramethylammonium fluoride (Me_4_NF, Figure 1, II).^24^ Direct methylation of C(sp^2^)–H bonds using phenyl trimethylammonium iodide and bromide as methyl source was realized by Uemura et al.^25^ under Ni^II^-catalysis (Figure 1, III).

With the below-described novel, safe, and metal-free method for α-methylation, we want to set a starting point in the relatively uncharted field of using quaternary ammonium salts as alkylating agents for C(sp^3^)–H bonds (Figure 1, IV).

## Results and Discussion

We started by investigating the methylation of benzyl 4-fluorophenyl ketone **1a** since quantification in all optimization steps can be accomplished *via*^19^F NMR using trifluoro toluene as an internal standard without preceding workup or solvent removal. Initially, Me_4_NBr was used as the methylating agent and KOH as the base in toluene at 130 °C. Here, we observed the methyl enol ether **2a** and the α-methylated product **3a** forming in a 1.2:1 ratio (Table 1, Entry 1). In a next step, it was investigated whether switching the solvent could shift the product distribution toward the desired product **3a**. Since the process should be as benign as possible, we aimed to find a suitable green solvent in combination with an inexpensive base. 2-Methyl-THF, anisole,^26^ and cyclopentylmethylether^27^ are considered green solvents and were tested (among others; see the SI for full list) in this transformation. Anisole (entry 3) showed the highest overall conversion and additionally slightly favored the desired product **3a** (entry 3, 1:1.08 ratio of **2a** and **3a**). 2-Methyl-THF and cyclopentylmethylether gave lower conversion and additionally favored the undesired product **2a** (entries 2 and 4). Other benign solvents proved to be inefficient (see complete solvent screening list in the SI). We further investigated the influence and efficiency of different bases. Hydroxy bases gave the best yields, with the initially used KOH surpassing NaOH. KO^*t*^Bu and Cs_2_CO_3_ showed significantly lower conversion. The other bases tested turned out to be inefficient (see the SI for details).

**Table 1:**
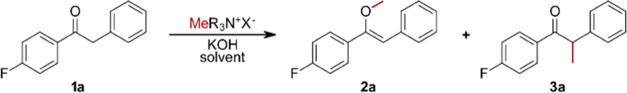
Optimization of the Reaction Conditions^a^

			yield (%)^b^		
entry	solvent	ammonium salt	**1a**	**2a**	**3a**
1	toluene	Me_4_NBr	0	41	34
2	MeTHF^c^	Me_4_NBr	25	14	11
3	anisole	Me_4_NBr	0	40	43
4	CPME	Me_4_NBr	4	36	24
5	anisole	Me_4_NCl	0	43	42
6	anisole	Me_4_NI	30	23	29
7^d^	anisole	Me_4_NOAc	0	49	9
8	anisole	PhMe_3_NCl	0	47	48
9	anisole	PhMe_3_NBr	0	38	50
10	anisole	PhMe_3_NI	0	18	78
11	anisole	Bu_3_MeNCl	6	25^e^	39
12	anisole	BnMe_3_NCl	0	0	0^f^
13	anisole	(C_16_H_33_)Me_3_NBr	0	47	25
14	anisole	betaine	30	2	5

a Reactions were performed on a 0.23 mmol scale, with KOH (2 equiv) as base, and 1.5 equiv of the ammonium salt under Ar atmosphere; reaction times: 22 h (entries 1–4) and 18 h (entries 5–15), 130 °C.

b Yield was determined by ^19^F NMR using trifluoro toluene as internal standard.

c 100 °C.

d Reaction time 3 h.

e 13% *O^n^*Bu-ether formation.

f 64% α-benzylation.

**Figure 2:**
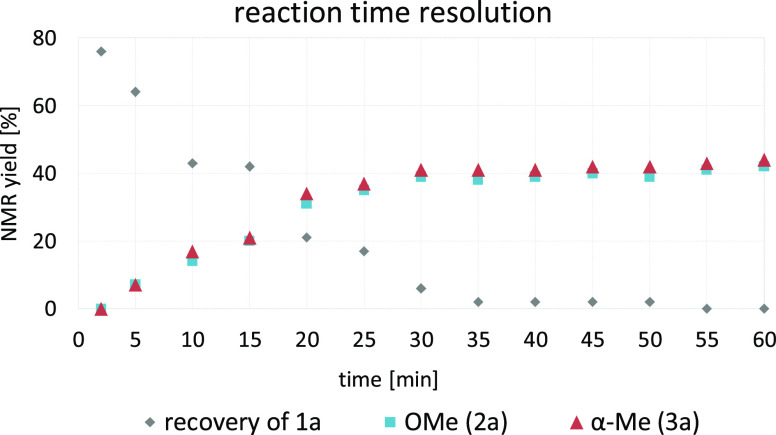
Reaction time screening; conditions: Me_4_NBr (1.5 equiv), KOH (2 equiv), anisole (0.2 M), 130 °C.

Before continuing with optimization of the methylating reagent, it was tested whether the *O*- and the α-methylated products **2a** and **3a** are formed independently or whether **2a** might be the actual methylating agent. The kinetic profile showed that both the *O*- and the α-methylated product are formed simultaneously under the given reaction conditions within 30 minutes, and no shift in product ratio could be observed at prolonged reaction times (see Figure 2). Furthermore, enol ether **2a** was subjected to the reaction conditions without any formation of **3a**.

And finally, a 1:1 mixture of **1a** and **2a** was subjected to the reaction conditions in the absence of Me_4_NBr without any formation of **3a**. This excludes that the two products are interconvertible under the applied conditions and are indeed formed independently (*cf.*Table 2).

**Table 2:**
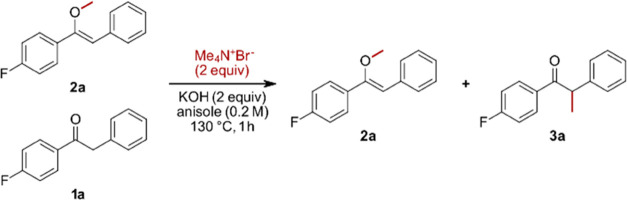
Studies for Interconversion of Products **2a** and **3a**

	substrate		yield (%)^b^		
entry	**1a** [mmol]	**2a** [mmol]	**1a**	**2a**	**3a**
1	0.093		0	42	43
2	0.047	0.044	0	73	9
3		0.088	0	86	0
4^a^	0.047	0.044	30	44	0

a The reaction was performed in the absence of the methylating agent (Me_4_NBr).

b Yield was determined by ^19^F NMR using trifluoro toluene as internal standard.

Next, we screened for different ammonium salts as methyl sources. We found that Me_4_NCl and Me_4_NBr gave equal yields and product ratios, whereas Me_4_NI gave incomplete conversion (entries 5 and 6). Tetramethylammonium acetate favored the *O*-methyl enol ether (entry 7). When using ammonium salts with different substituents on the quaternary nitrogen, we observed additional *O*-butylation (13%) with Bu_3_MeNCl (entry 11) and mainly α-benzylation (64%) with BnMe_3_NCl (entry 12). When using (C_16_H_33_)Me_3_NBr as an alkylating agent, only **2a** and **3a** were formed, but no hexadecylated compound of any kind (entry 13). The naturally occurring ammonium salt betaine was practically ineffective (entry 14). Gratifyingly, we identified phenyl trimethylammonium salts giving significantly higher overall yields. Going from the chloride and bromide to the iodide salt, we observed a shift towards the desired α-methylated product **3a** (entries 8–10). Compared with tetramethylammonium salts, a phenyl substituent on the ammonium most probably withdraws electron density from the adjacent methyl substituents, which then, in turn, are more prone to react with the “soft” α-carbon of the enolate rather than being attacked by the carbonyl oxygen. Finally, we found the optimal reaction conditions being PhMe_3_NI (1.5 equiv) and KOH (2 equiv) in anisole (0.23 M) at 130 °C, wherein the desired 1-(4-fluorophenyl)-2-phenyl-1-propanone (**3a**) was obtained in 78% yield after 18 h (entry 10) determined by ^19^F NMR.

The outcome of the optimization efforts corresponds to previous studies on quaternary ammonium compounds as alkylating agents present in the literature.^28,29^ Accordingly, for ammonium salts with different organic substituents on the nitrogen, a benzyl group is transferred preferentially from the ammonium salt to a nucleophile, followed by methyl substituents, and finally, other primary alkyl chains. The substituents on the ammonium ion further impact the cleavage rate of neighboring alkyl groups. If an aryl substituent is present within the ammonium salt, an adjacent alkyl group is transferred more readily compared to an aliphatic chain from tetraalkylammonium salts.

To exclude a reaction pathway *via* thermal decomposition of the ammonium salt to the respective methyl halide, which could act as the actual methylating reagent, we choose a reaction setup that would allow transfer of gaseous reactants between two spatially divided reaction vessels. For this purpose, a COware vial (Skrydstrup vial^30^) was used, with two separate reaction chambers connected at their upper part for gas exchange. Chamber 1 was charged with the ammonium salt, base, and anisole as solvent, and chamber 2 was charged with substrate **1a**, base, and solvent. The whole vessel was heated to 130 °C, where possibly formed methyl halide from chamber 1 should reach chamber 2 *via* the gas phase. However, no methylated product could be observed, and solely starting material was recovered. Furthermore, methylation occurred when Me_4_NOAc was used as CH_3_ source, which again corroborates the hypothesis of direct nucleophilic substitution rather than thermal decomposition to a methylating agent. Additionally, when methylating phenyl benzyl ketone by MeI, solely the α-mono- and α-bis-methylated products form, but no O-methylation is observed.^31^ Furthermore, we successfully performed α-methylation using PhMe_3_NI at lower temperatures by exploiting microwave irradiation. A decrease of reaction temperature as low as 90 °C still afforded the desired product **3** in comparable yields (see the SI for details).

**Scheme 1:**
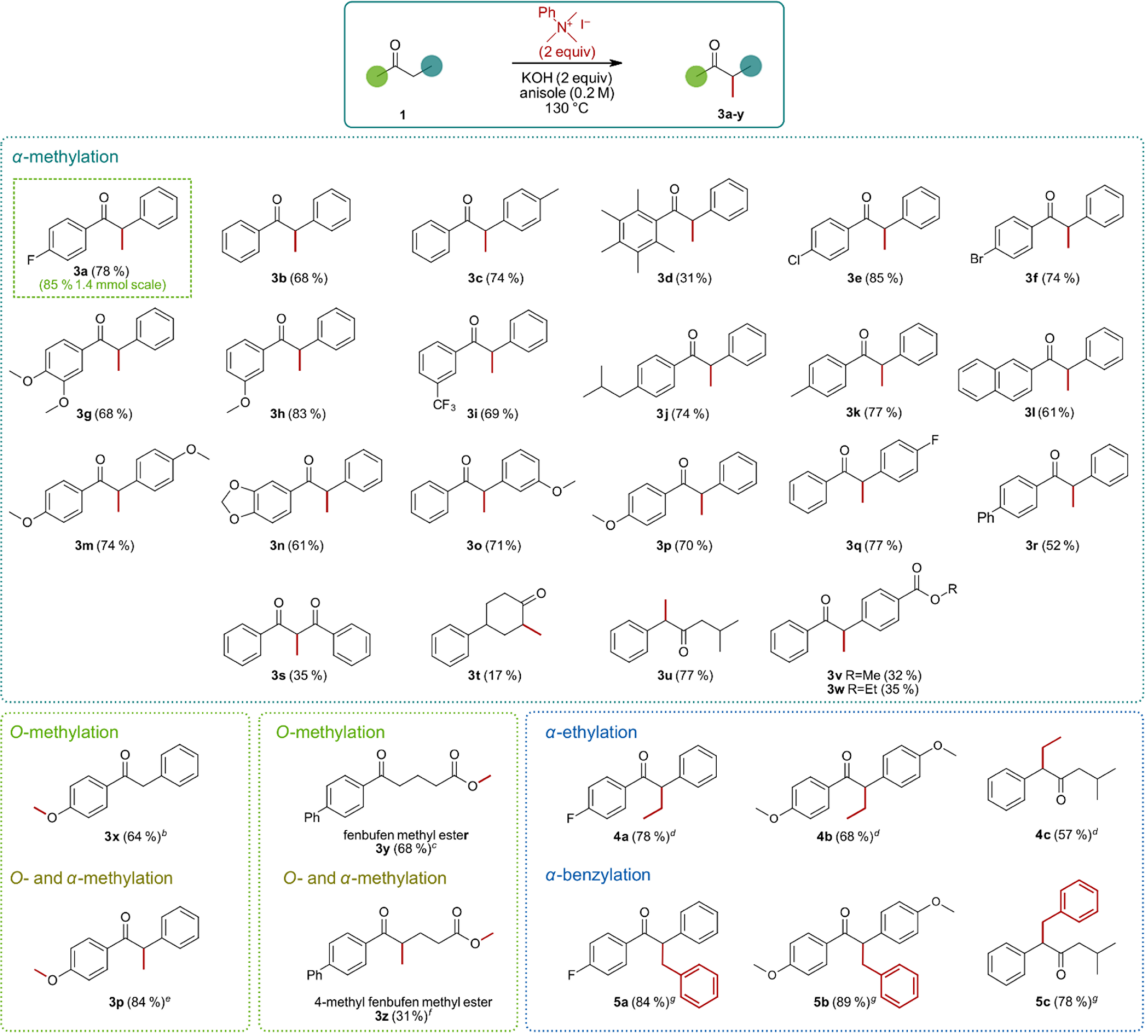
Scope of α-Methylation 1 equiv PhMe_3_NI. PhEt_3_NI (2 equiv) as ammonium salt. Reaction time 6 h, addition of KOH (2 equiv) and PhMe_3_NI (2 equiv) after 3 h. Addition of KOH (2 equiv) and PhMe_3_NI (2 equiv) after 3 h and 48 h; reaction time, 4 days. BnMe_3_NCl (1 equiv) as ammonium salt 3 equiv KOH, reaction time 24 h. Isolated yields are shown. Standard conditions: Substrate (100 mg, 1 equiv), PhMe_3_NI (2 equiv), KOH (2 equiv), in anisole (2 mL, 0.2 M) at 130 °C, 2–5 h, closed vessel, inert atmosphere.

With the optimized reaction conditions in hand, we performed α-methylation reactions on various substrates to demonstrate the scope of this direct transformation (Scheme 1).

In this reaction *N,N*-dimethylaniline is formed stoichiometrically from the methylating agent PhMe_3_NI. This byproduct, however, can be easily quenched *in situ* and fully separated from the desired product in form of its water-soluble HCl salt by a mild acidic workup procedure. The desired methylated compounds were obtained in isolated yields up to 85%. Interestingly, the formation of any α,α-dimethylated products was never observed. We performed the methylation of benzyl 4-fluorophenyl ketone **1a** on a 1.4 mmol scale to prove the scalability of this method. The desired product **3a** was isolated in a yield of 85%. Significantly lower yields were observed for the sterically more hindered substrate 1-(pentamethylphenyl)-2-phenylethanone (product **3d**). Substrates that are less susceptible to enolization, *e.g.*, 4-phenylcyclohexanone **3t**, also resulted in diminished yields, and mainly starting material was recovered. A variety of functional groups, including halides (products **3a**, **3e, 3f**, and **3q**), CF_3_ (product **3i**), ether (products **3g** & **3h, 3m–3p**), and phenyl groups (product **3r**) were well tolerated in different positions of the aryl ring. Substrates bearing even more reactive functional groups on the aryl ring, *e.g.*, ester moieties, can also be methylated in moderate yields (product **3v** and **3w**). As assumed, when 1-(4-hydroxyphenyl)-2-phenylethanone was subjected to the respective conditions, methylation initially occurred at the phenolic oxygen, and subsequently at the α-position of the carbonyl (product **3x** and **3p**). Our method, however, is not only limited to bisaromatic compounds but can also be applied for monoaromatic substrates. 4-Methyl-1-phenyl-2-pentanone was methylated regioselectively at the benzylic position giving product **3u** in 77% yield. Aliphatic ketones without any benzylic α-carbons, *e.g.*, 8-pentadecanone, formed only the aldol product and hence are not mentioned in this paper. As a proof of concept, we performed late-stage methylation of the biologically active compound fenbufen. Herein, the carboxylic acid moiety is preferentially methylated (product **3y**). Upon addition of fresh reagent after prolonged reaction times, however, the fenbufen methyl ester could be further methylated at the α-position (product **3z**; see the SI for details).

Finally, we wanted to briefly outline the applicability of this new protocol for introducing larger substituents than methyl. Selective α-ethylation can be accomplished accordingly, using phenyltriethylammonium iodide (PhEt_3_NI) as the alkyl source. Benzyl 4-fluorophenyl ketone **1a** was successfully ethylated at the α-position in 78% yield using PhEt_3_NI (product **4a**). Substrates containing electron-donating substituents on the aryl ring (product **4b**), as well as monoaromatic compounds (product **4c**) can also be ethylated in yields of 68 and 57%, respectively. Benzylation is of interest since the phenyl benzyl ketone motif can be found in several drugs or promising drug candidates, as, for example, desoxybenzoin derivatives^32^ or ring-truncated deguelin analogues.^33^ SAR studies identified the latter as promising candidates for HIF-1α inhibitors.^34^ One of those analogues, SH-1242, further inhibits Hsp90 activity and shows potent anticancer efficacy.^35^ We could demonstrate the applicability of this method for benzylation of selected substrates using BnMe_3_NCl as a benzylating agent. Products **5a–5c** were obtained in high yields of 84, 89, and 78%, respectively. Since methylating agents, however, are by far more hazardous than traditionally applied ethylating and benzylating reagents, we did not further investigate the latter strategies.

## Conclusions

In conclusion, we described the use of quaternary ammonium salts as alternative alkylating and benzylating agents. Phenyl trimethylammonium iodide and related salts were successfully established as selective, highly efficient, safe, and easy-to-handle methylating reagents for direct C(sp^3^)–C(sp^3^) bond formation.

## Experimental Section

### General

All chemicals were purchased from commercial suppliers and, unless noted otherwise, used without further purification. NaO^*t*^Bu, Pd_2_(dba)_3_, and DPE-Phos were strictly stored and handled in a glovebox under argon atmosphere. Degassed and dry THF was stored over molecular sieves under argon using AcroSeal septum. Glass vials (8 mL) were sealed with Wheaton screw caps containing a PTFE faced 14B styrene-butadiene rubber liner for small-scale reaction above room temperature and heated in a metallic reaction block. All reaction temperatures refer to external temperatures.

^1^H NMR, ^13^C NMR, and ^19^F NMR spectra were recorded on a Bruker Avance UltraShield 400 at ambient temperature. Chemical shifts (δ) are reported in ppm, using Me_4_Si as internal standard. Coupling constants (*J*) are given in hertz (Hz), and multiplicities are assigned as s = singlet, d = doublet, t = triplet, q = quartet, and m = multiplet.

Thin-layer chromatography (TLC) analysis was performed on aluminum-backed unmodified Merck silica gel 60 F_245_ plates. Visualization was realized under UV irradiation or *via* heat staining using a ceric ammonium molybdate aqueous solution. For flash column chromatography, Merck silica gel 60 (40–63 μm) was used and purification was either done by hand column or on a Büchi Pure C-850 FlashPrep System.

HRMS analysis was performed on an Agilent 6230 LC TOFMS mass spectrometer equipped with an Agilent Dual AJS ESI-Source. The mass spectrometer was connected to a liquid chromatography system of the 1100/1200 series from Agilent Technologies, Palo Alto, CA. The system consisted of a 1200SL binary gradient pump, a degasser, a column thermostat, and an HTC PAL autosampler (CTC Analytics AG, Zwingen, Switzerland). A silica-based Phenomenex C-18 Security Guard Cartridge was used as a stationary phase. Data evaluation was performed using Agilent MassHunter Qualitative Analysis B.07.00. Identification was based on peaks obtained from extracted-ion chromatograms (extraction width, ±20 ppm).

### Optimization Screening

The optimization of reaction conditions was conducted following the general procedure A (see the SI for details). Yields were determined by ^19^F NMR using trifluoro toluene as internal standard.

#### 1-Fluoro-4-(1-methoxy-2-phenylethenyl)benzene^36^ (**2a**)

An 8 mL glass vial equipped with a magnetic stirring bar was charged with benzyl 4-fluorophenyl ketone **(1)** (100 mg, 0.467 mmol, 1 equiv), Me_4_NBr (119 mg, 770 mmol, 1.65 equiv), and KOH (79 mg, 1.4 mmol, 3 equiv). The vial was sealed with a septum screw cap. Using a cannula, the vial was evacuated and backfilled with argon three times. The toluene (2 mL, 0.23 M) was added *via* a syringe. Evacuation and backfilling with argon were repeated three times under vigorous stirring that no boiling delay occurred. Subsequently, the septum screw cap was exchanged for a closed Wheaton cap, and the vial was sealed tightly. The resulting inhomogeneous mixture was heated to 130 °C in a metallic heating block. After 18 h at respective temperatures, the reaction was cooled to room temperature and solids were centrifuged off. The supernatant solution was transferred to a round-bottom flask, and the solid residue was washed three times with small amounts DCM. The combined organic phases were concentrated. The crude oil was further purified *via* hand column chromatography (8 g silica LP/Et_3_N 100:1) to yield 46 mg (43%) of the title compound as white crystals. ^1^H NMR (400 MHz, CDCl_3_): δ = 7.74–7.67 (m, 2H), 7.60–7.50 (m, 2H), 7.42–7.32 (m, 2H), 7.28–7.19 (m, 1H), 7.16–7.03 (m, 2H), 6.06 (s, 1H), 3.63 (s, 3H). ^13^C{^1^H} NMR (101 MHz, CDCl_3_): δ = 163.0 (d, *J* = 247.9 Hz), 155.4, 135.9, 132.6 (d, *J* = 3.3 Hz), 128.7, 128.6, 128.5 (d, *J* = 8.1 Hz), 126.8, 115.6 (d, *J* = 21.7 Hz), 112.8 (d, *J* = 1.4 Hz), 58.0. ^19^F NMR (376 MHz, CDCl_3_): δ = −113.2 HRMS (ESI): *m*/*z* [M + H]^+^ calcd for C_15_H_14_FO: 229.1023; found: 229.1000

### General Procedure B for Precursor Synthesis

In the glovebox, a flame-dried 8 mL glass vial equipped with a magnetic stirring bar was charged with NaO^*t*^Bu (2.6 mmol, 1.3 equiv), Pd_2_(dba)_3_ (5 mol %), and DPE-Phos (10 mol %). THF (2 mL, 1 M) was added, and the dark brownish-green mixture was stirred for 5 min at ambient temperatures. The aryl bromide (2 mmol, 1 equiv) was added *via* Eppendorf pipette, followed by rapid addition of the acetophenone (2.4 mmol, 1.2 equiv) in one portion as solid or *via* Eppendorf pipette if liquid. Immediate solid formation could be observed. The vial was closed with a Wheaton screw cap and transferred out of the glovebox. The mixture was heated to 70 °C in a metallic reaction block and stirred for 2–18 h at respective temperatures. After complete consumption of the starting material (GC-MS monitoring), water (10 mL) was added and the mixture was extracted three times with diethyl ether (30 mL each). The combined organic phases were washed once with sat. NH_4_Cl solution and once with brine, dried over anhydrous Na_2_SO_4_, filtered, and concentrated. The crude product was purified *via* gradient flash column chromatography on silica gel using a mixture of light petroleum (LP) and EtOAc.

#### 1-(3,4-Dimethoxyphenyl)-2-phenylethanone^37^ (**1g**)

Prepared following the general procedure B from 3,4-dimethoxyacetophenone and bromobenzene heated for 2 h. The crude product was purified *via* flash column chromatography (90 g silica, LP, and EtOAc 0–40%) to yield 443 mg (86%) of the title compound as a slightly yellow oil. ^1^H NMR (400 MHz, CDCl_3_): δ = 7.68 (dd, *J* = 8.4, 2.1 Hz, 1H), 7.58 (d, *J* = 2.1 Hz, 1H), 7.39–7.22 (m, 5H), 6.89 (d, *J* = 8.4 Hz, 1H), 4.26 (s, 2H), 3.95 (s, 3H), 3.93 (s, 3H).^13^C{^1^H} NMR (100 MHz, CDCl_3_): δ = 196.3, 153.3, 149.1, 135.1, 129.7, 129.3, 128.6, 126.8, 123.5, 110.7, 110.0, 56.0, 55.9, 45.2.

#### 1-(3-Methoxyphenyl)-2-phenylethanone^38^ (**1h**)

Prepared following the general procedure B from 3-methoxyacetophenone and bromobenzene heated for 3 h. The crude product was purified *via* flash hand column chromatography (60 g silica, LP/EtOAc 70:1, 60:1, 40:1) to yield 305 mg (67%) of the title compound as a colorless oil. ^1^H NMR (400 MHz, CDCl_3_): δ = 7.65–7.58 (m, 1H), 7.55 (dd, *J* = 2.7, 1.6 Hz, 1H), 7.43–7.31 (m, 3H), 7.31–7.26 (m, 3H), 7.11 (ddd, *J* = 8.2, 2.7, 0.9 Hz, 1H), 4.28 (s, 2H), 3.84 (s, 3H). ^13^C{^1^H} NMR (100 MHz, CDCl_3_): δ = 197.5, 159.9, 138.0, 134.6, 129.6, 129.5, 128.7, 126.9, 121.3, 119.7, 112.9, 55.4, 45.7.

#### 2-Phenyl-1-[3-(trifluormethyl)phenyl]ethenone^39^ (**1i**)

Prepared following the general procedure B from 3-trifluoromethylacetophenone and bromobenzene heated for 4 h. The crude product was purified *via* flash column chromatography (90 g silica, LP, and EtOAc 0–15%) to yield 391 mg (74%) of the title compound as an orange oil. ^1^H NMR (400 MHz, CDCl_3_): δ = 8.26 (tt, *J* = 1.8, 0.8 Hz, 1H), 8.16 (dt, *J* = 7.3, 1.1 Hz, 1H), 7.81–7.70 (m, 1H), 7.56 (tt, *J* = 7.9, 0.8 Hz, 1H), 7.38–7.29 (m, 2H), 7.29–7.18 (m, 3H), 4.29 (s, 2H). ^13^C{^1^H} NMR (100 MHz, CDCl_3_): δ = 196.3, 137.1, 133.9, 131.9 (d, *J* = 1.4 Hz), 131.2 (q, *J* = 34.0 Hz), 129.6 (q, *J* = 3.6 Hz), 129.5, 129.4, 128.9, 127.3, 125.5 (q, *J* = 3.8 Hz), 123.7 (d, *J* = 274.8 Hz), 45.70.

#### 1-[4-(2-Methylpropyl)phenyl]-2-phenylethanone (**1j**)

Prepared following the general procedure B from 4′-isobutylacetophenone and bromobenzene heated for 4 h. The crude product was purified *via* flash column chromatography (90 g silica, LP, and EtOAc 0–20%) to yield 415 mg (82%) of the title compound as a yellow oil. ^1^H NMR (400 MHz, CDCl_3_): δ = 8.03–7.96 (m, 2H), 7.42–7.23 (m, 7H), 4.30 (s, 2H), 2.57 (d, *J* = 7.2 Hz, 2H), 2.04–1.86 (m, *J* = 6.9 Hz, 1H), 0.96 (d, *J* = 6.7 Hz, 6H). ^13^C{^1^H} NMR (100 MHz, CDCl_3_): δ = 197.2, 147.6, 134.8, 134.4, 129.5, 129.3, 128.6, 126.8, 45.3, 30.1, 22.3.

#### 1-(4-Methylphenyl)-2-phenylethanone^40^ (**1k**)

Prepared following the general procedure B from 4-methylacetophenone and bromobenzene heated for 3 h. The crude product was purified *via* flash hand column chromatography (55 g silica, LP/EtOAc 80:1, 70:1, 60:1, 40:1) to yield 302 mg (72%) of the title compound as a colorless oil. ^1^H NMR (400 MHz, CDCl_3_): δ = 7.94 (d, *J* = 7.9 Hz, 2H), 7.36–7.24 (m, 7H), 4.28 (s, 2H), 2.42 (s, 3H). ^13^C{^1^H} NMR (100 MHz, CDCl_3_): δ = 197.4, 144.1, 134.9, 134.2, 129.5, 129.4, 128.8, 128.7, 126.9, 45.5, 21.7.

#### 1-(2-Naphthalenyl)-2-phenylethanone^40^ (**1l**)

Prepared following the general procedure B from 2-acetylnaphthalene and bromobenzene heated for 18 h. The crude product was purified *via* flash column chromatography (90 g silica, LP, and EtOAc 0–40%) to yield 396 mg (80%) of the title compound as an off-white solid. ^1^H NMR (400 MHz, CDCl_3_): δ = 8.47 (d, *J* = 1.8 Hz, 1H), 8.00 (dd, *J* = 8.6, 1.8 Hz, 1H), 7.88 (dd, *J* = 8.1, 1.4 Hz, 1H), 7.85–7.73 (m, 2H), 7.57–7.41 (m, 2H), 7.32–7.15 (m, 5H), 4.34 (s, 2H). ^13^C{^1^H} NMR (100 MHz, CDCl_3_): δ = 197.6, 135.6, 134.7, 134.0, 132.5, 130.4, 129.7, 129.5, 128.7, 128.6, 128.6, 127.8, 126.9, 126.8, 124.3, 45.6.

#### 1-(1,3-Benzodioxol-5-yl)-2-phenylethanone^40^ (**1n**)

Prepared following the general procedure B from 5-acetyl-1,3-benzodiocole and bromobenzene heated for 10 h. The crude product was purified *via* flash column chromatography (90 g silica, LP and EtOAc 0–40%) to yield 471 mg (98%) of the title compound as a slightly yellow oil. ^1^H NMR (400 MHz, CDCl_3_): δ = 7.64 (dd, *J* = 8.2, 1.8 Hz, 1H), 7.49 (d, *J* = 1.7 Hz, 1H), 7.39–7.29 (m, 2H), 7.29–7.21 (m, 3H), 6.85 (d, *J* = 8.2 Hz, 1H), 6.03 (s, 2H), 4.21 (s, 2H). ^13^C{^1^H} NMR (100 MHz, CDCl_3_): δ = 195.8, 151.9, 148.3, 134.9, 131.5, 129.4, 128.7, 128.7, 126.9, 125.1, 108.8, 108.4, 107.9, 101.9, 45.4.

#### 2-(3-Methoxyphenyl)-1-phenylethanone^40^ (**1o**)

Prepared following the general procedure B from acetophenone and 4-bromoanisole heated for 18 h. The crude product was purified *via* flash column chromatography (90 g silica, LP, and EtOAc 0–40%) to yield 267 mg (59%) of the title compound as a yellow oil. ^1^H NMR (400 MHz, CDCl_3_): δ = 8.06–7.97 (m, 2H), 7.60–7.51 (m, 1H), 7.51–7.41 (m, 2H), 7.25 (t, *J* = 7.8 Hz, 1H), 6.91–6.77 (m, 3H), 4.26 (s, 2H), 3.79 (s, 3H). ^13^C{^1^H} NMR (101 MHz, CDCl_3_): δ = 197.5, 159.8, 136.6, 136.1, 133.2, 129.7, 128.7, 121.9, 115.2, 112.4, 55.2, 45.6.

#### 2-(4-Fluorphenyl)-1-phenylethanone^38^ (**1q**)

Prepared following the general procedure B from acetophenone and 1-bromo-4-fluorobenzene heated for 18 h. The crude product was purified *via* flash column chromatography (90 g silica, LP, and EtOAc 0–40%) to yield 255 mg (60%) of the title compound as a slightly yellow oil. ^1^H NMR (400 MHz, CDCl_3_): δ = 8.06–7.94 (m, 2H), 7.62–7.52 (m, 1H), 7.52–7.39 (m, 2H), 7.28–7.19 (m, 2H), 7.08–6.97 (m, 2H), 4.27 (s, 2H). ^13^C{^1^H} NMR (100 MHz, CDCl_3_): δ = 197.5, 162.0 (d, *J* = 245.2 Hz), 136.6, 133.4, 131.2 (d, *J* = 8.0 Hz), 130.3 (d, *J* = 3.3 Hz), 128.8, 128.6, 115.6 (d, *J* = 21.4 Hz), 44.6.

#### 1-[1,1′-Biphenyl]-4-yl-2-phenylethanone^40^ (**1r**)

Prepared following the general procedure B from 4′-phenylacetophenone and bromobenzene heated for 18 h. The crude product was purified *via* flash column chromatography (90 g silica, LP, and EtOAc 0–40%) to yield 207 mg (38%) of the title compound as a colorless oil. ^1^H NMR (400 MHz, CDCl_3_): δ = 8.12–8.04 (m, 2H), 7.70–7.64 (m, 2H), 7.64–7.59 (m, 2H), 7.50–7.43 (m, 2H), 7.43–7.22 (m, 6H), 4.31 (s, 2H). ^13^C{^1^H} NMR (100 MHz, CDCl_3_): δ = 197.2, 145.8, 139.8, 135.3, 134.7, 129.5, 129.3, 129.0, 128.7, 128.3, 127.3, 127.3, 126.9, 45.6

#### Methyl 4-(2-Oxo-2-phenylethyl)benzoate^41^ (**1v**)

Prepared following the general procedure B from acetophenone methyl 4-iodobenzoate heated for 4 h. The crude product was purified *via* flash column chromatography (90 g silica, LP, and EtOAc 0–20%) to yield 285 mg (37%) of the title compound as a white solid. ^1^H NMR (400 MHz, CDCl_3_): δ = 8.04–7.98 (m, 4H), 7.62–7.54 (m, 1H), 7.51–7.44 (m, 2H), 7.37–7.32 (m, 2H), 4.35 (s, 2H), 3.90 (s, 3H). ^13^C{^1^H} NMR (100 MHz, CDCl_3_): δ = 196.9, 167.0, 139.9, 136.5, 133.5, 130.0, 129.7, 129.0, 128.8, 128.6, 77.4, 52.2, 45.5.

#### Ethyl 4-(2-Oxo-2-phenylethyl)benzoate^42^ (**1w**)

Prepared following the general procedure B from acetophenone ethyl 4-iodobenzoate heated for 4 h. The crude product was purified *via* flash column chromatography (90 g silica, LP, and EtOAc 0–20%) to yield 360 mg (45%) of the title compound as a white solid. ^1^H NMR (400 MHz, CDCl_3_): δ = 8.04–7.97 (m, 4H), 7.62–7.53 (m, 1H), 7.51–7.42 (m, 2H), 7.37–7.31 (m, 2H), 4.41–4.31 (m, 4H), 1.38 (t, *J* = 7.1 Hz, 3H) ^13^C{^1^H} NMR (100 MHz, CDCl_3_): δ = 196.9, 166.5, 139.8, 136.5, 133.5, 130.0, 129.6, 128.8, 128.6, 61.0, 45.5, 14.4.

### General Procedure C for Methylation, Ethylation, and Benzylation Reactions

An 8 mL glass vial equipped with a magnetic stirring bar was charged with the respective diaryl ethanone (100 mg, 1 equiv), the ammonium salt (1.1 for BnMe_3_NCl or 2 equiv for PhMe_3_NI and PhEt_3_NI), and KOH (2 equiv). The vial was sealed with a septum screw cap. Using a cannula, the vial was evacuated and backfilled with argon three times. Anisole (2 mL, 0.2 M) was added *via* a syringe. Evacuation and backfilling with argon were repeated three times under vigorous stirring that no boiling delay occurred. Subsequently, the septum screw cap was exchanged for a closed Wheaton cap and the vial was sealed tightly. The resulting inhomogeneous mixture was heated to 130 °C in a metallic heating block for 2–4 h. After complete consumption of the starting material (TLC analysis), the reaction was cooled to room temperature. HCl (2 N, 2 mL) was added, and the mixture was extracted three times with EtOAc (5 mL each). The combined organic phases were washed twice with 2 N HCl (1 mL each) and once with brine, dried over anhydrous Na_2_SO_4_, filtered, and concentrated. For benzylation reactions, the mixture was not subjected to aqueous workup but filtered over a short plug of silica, washed with EtOAc, and concentrated. The obtained crude product was purified *via* hand column with unmodified silica.

#### 1-(4-Fluorophenyl)-2-phenyl-1-propanone^43^ (**3a**)

Prepared following the general procedure C from commercially available starting material with a reaction time of 3 h. The crude product was purified *via* column chromatography (8 g silica LP/EtOAc 50:1, 45:1, 40:1) to yield 83 mg (78%) of the title compound.^1^H NMR (400 MHz, CDCl_3_): δ = 7.97–7.87 (m, 2H), 7.28–7.18 (m, 4H), 7.18–7.11 (m, 1H), 7.03–6.93 (m, 2H), 4.57 (q, *J* = 6.8 Hz, 1H), 1.48 (d, *J* = 6.9 Hz, 3H). ^13^C{^1^H} NMR (101 MHz, CDCl_3_): δ = 198.8, 165.6 (d, *J* = 254.6 Hz), 141.5, 133.0 (d, *J* = 3.0 Hz), 131.5 (d, *J* = 9.3 Hz), 129.20, 127.8, 127.1, 115.7 (d, *J* = 21.7 Hz), 48.1, 19.6.^19^F NMR (376 MHz, CDCl_3_): δ = −105.6. HRMS (ESI): *m*/*z* [M + H]^+^ calcd for C_15_H_14_FO: 229.1023; found: 229.1000

Compound **3*****a*** was also prepared on a 1.4 mmol scale as follows: A 25 mL round-bottom flask was charged with benzyl 4-fluorophenyl ketone **(1a)** (300 mg, 1.4 mmol, 1 equiv), PhMe_3_NI (751 mg, 2.8 mmol, 2 equiv), and KOH (157 mg, 2.8 mmol, 2 equiv). The flask was closed with a septum. Using a cannula, the flask was evacuated and backfilled with argon three times. Anisole (6 mL, 0.23 M) was added *via* a syringe. Evacuation and backfilling with argon were repeated three times under vigorous stirring that no boiling delay occurred. The resulting inhomogeneous mixture was heated to 130 °C in an oil bath. After 5 h at respective temperatures, the reaction was cooled to room temperature. HCl (2 N, 10 mL) were added, and the mixture was extracted three times with EtOAc (25 mL each). The combined organic phases were washed twice with 2 N HCl (3–5 mL each) and once with brine, dried over anhydrous Na_2_SO_4_, filtered, and concentrated. The obtained crude product was purified *via* flash column chromatography (90 g silica, LP, and EtOAc 0–40%) to yield 273 mg (85%) of the title compound as a colorless oil. Analytical data were in accordance with the previous finding.

#### 1,2-Diphenyl-1-propanone^38^ (**3b**)

Prepared following the general procedure C from commercially available starting material with a reaction time of 3 h. The crude product was purified *via* column chromatography (8 g silica, LP/EtOAc 50:1, 40:1) to yield 73 mg (68%) of the title compound as a colorless oil. ^1^H NMR (400 MHz, CDCl_3_): δ = 8.01–7.93 (m, 2H), 7.53–7.43 (m, 1H), 7.43–7.34 (m, 2H), 7.30 (d, *J* = 4.3 Hz, 4H), 7.21 (ddd, *J* = 8.8, 4.8, 3.9 Hz, 1H), 4.70 (q, *J* = 6.9 Hz, 1H), 1.55 (d, *J* = 6.9 Hz, 3H). ^13^C{^1^H} NMR (101 MHz, CDCl_3_): δ = 200.4, 141.6, 136.6, 132.9, 129.1, 128.9, 128.6, 127.9, 127.0, 48.0, 19.6.

#### 2-(4-Methylphenyl)-1-phenyl-1-propanone^44^ (**3c**)

Prepared following the general procedure C from commercially available starting material with a reaction time of 2 h. The crude product was purified *via* column chromatography (8 g silica, LP/EtOAc 50:1) to yield 76 mg (74%) of the title compound as a slightly yellow oil. ^1^H NMR (400 MHz, CDCl_3_): δ = 8.01–7.94 (m, 2H), 7.52–7.43 (m, 1H), 7.43–7.34 (m, 2H), 7.23–7.16 (m, 2H), 7.15–7.08 (m, 2H), 4.67 (q, *J* = 6.8 Hz, 1H), 2.30 (s, 3H), 1.54 (d, *J* = 6.9 Hz, 3H). ^13^C{^1^H} NMR (101 MHz, CDCl_3_): δ = 200.5, 138.6, 136.6, 136.6, 132.8, 129.8, 128.9, 128.6, 127.7, 47.6, 21.1, 19.6. HRMS (ESI): *m*/*z* [M + H]^+^ calcd for C_16_H_17_O: 225.1274; found: 225.1265

#### 2-(2,3,4,5,6-Pentamethylphenyl)-1-phenyl-1-propanone (**3d**)

Prepared following the general procedure C from commercially available starting material with a reaction time of 3.5 h. The crude product was purified *via* column chromatography (8 g silica, LP/EtOAc 50:1) to yield 31 mg (31%) of the title compound as off-white crystals. ^1^H NMR (400 MHz, CDCl_3_): δ = 7.32–7.16 (m, 5H), 4.13 (q, *J* = 7.0 Hz, 1H), 2.23 (s, 15H), 1.64 (d, *J* = 7.0 Hz, 3H). ^13^C{^1^H} NMR (101 MHz, CDCl_3_): δ = 211.1, 140.1, 138.7, 135.5, 132.8, 128.8, 128.5, 127.1, 54.9, 16.9, 16.8, 16.0. HRMS (ESI): *m*/*z* [M + H]^+^ calcd for C_20_H_25_O: 281.1900; found: 281.1895

#### 1-(4-Chlorophenyl)-2-phenyl-1-propanone^45^ (**3e**)

Prepared following the general procedure C from commercially available starting material with a reaction time of 2 h. The crude product was purified *via* column chromatography (8 g silica, LP/EtOAc 50:1) to yield 87 mg (85%) of the title compound. ^1^H NMR (400 MHz, CDCl_3_): δ = 7.92–7.84 (m, 2H), 7.38–7.16 (m, 7H), 4.62 (q, *J* = 6.8 Hz, 1H), 1.53 (d, *J* = 6.8 Hz, 3H). ^13^C{^1^H} NMR (101 MHz, CDCl_3_): δ = 199.1, 141.3, 139.3, 134.9, 130.3, 129.2, 128.9, 127.8, 127.2, 48.2, 19.5. HRMS (ESI): *m*/*z* [M + H]^+^ calcd for C_15_H_14_ClO: 245.0728; found: 245.0712

#### 1-(4-Bromophenyl)-2-phenyl-1-propanone^45^ (**3f**)

Prepared following the general procedure C from commercially available starting material with a reaction time of 2 h. The crude product was purified *via* column chromatography (8 g silica, LP/EtOAc 50:1) to yield 75 mg (74%) of the title compound. ^1^H NMR (400 MHz, CDCl_3_): δ = 7.84–7.76 (m, 2H), 7.56–7.46 (m, 2H), 7.36–7.25 (m, 3H), 7.25–7.16 (m, 2H), 4.61 (q, *J* = 6.8 Hz, 1H), 1.53 (d, *J* = 6.8 Hz, 3H). ^13^C{^1^H} NMR (101 MHz, CDCl_3_): δ = 199.3, 141.3, 135.2, 131.9, 130.4, 129.2, 128.0, 127.8, 127.2, 48.2, 19.5. HRMS (ESI): *m*/*z* [M + H]^+^ calcd for C_15_H_14_BrO: 289.0223; found: 289.0218

#### 1-(3,4-Dimethoxyphenyl)-2-phenyl-1-propanone^46^ (**3g**)

Prepared following the general procedure C from compound **1g** with a reaction time of 2 h. The crude product was purified *via* column chromatography (8 g silica, LP/EtOAc 40:1, 20:1, 10:1) to yield 72 mg (68%) of the title compound. R_f_ = 0.54 (LP/EtOAc 2:1) ^1^H NMR (400 MHz, CDCl_3_): δ = 7.59 (dd, *J* = 8.4, 2.0 Hz, 1H), 7.53 (d, *J* = 2.0 Hz, 1H), 7.29 (d, *J* = 4.4 Hz, 4H), 7.19 (ddd, *J* = 8.6, 4.9, 3.9 Hz, 1H), 6.80 (d, *J* = 8.5 Hz, 1H), 4.65 (q, *J* = 6.9 Hz, 1H), 3.88 (d, *J* = 4.1 Hz, 6H), 1.52 (d, *J* = 6.8 Hz, 3H). ^13^C{^1^H} NMR (101 MHz, CDCl_3_): δ = 199.0, 153.1, 149.0, 142.2, 129.7, 129.1, 127.7, 126.9, 123.5, 111.1, 110.0, 56.1, 56.0, 47.6, 19.7. HRMS (ESI): *m*/*z* [M + H]^+^ calcd for C_17_H_19_O_3_: 271.1329; found: 271.1323

#### 1-(3-Methoxyphenyl)-2-phenyl-1-propanone^47^ (**3h**)

Prepared following the general procedure C from compound **1h** with a reaction time of 2 h. The crude product was purified *via* column chromatography (8 g silica, LP/EtOAc 70:1) to yield 88 mg (83%) of the title compound as a slightly orange oil. ^1^H NMR (400 MHz, CDCl_3_): δ = 7.51 (ddd, *J* = 7.7, 1.6, 1.0 Hz, 1H), 7.47 (dd, *J* = 2.7, 1.6 Hz, 1H), 7.34–7.21 (m, 5H), 7.21–7.12 (m, 1H), 6.99 (ddd, *J* = 8.3, 2.7, 1.0 Hz, 1H), 4.64 (q, *J* = 6.9 Hz, 1H), 3.76 (s, 3H), 1.51 (d, *J* = 6.9 Hz, 3H). ^13^C{^1^H} NMR (101 MHz, CDCl_3_): δ = 200.2, 159.8, 141.6, 138.0, 129.5, 129.1, 127.8, 127.0, 121.5, 119.4, 113.2, 55.4, 48.1, 19.6. HRMS (ESI): *m*/*z* [M + H]^+^ calcd for C_16_H_17_O_2_: 241.1223; found: 241.1205

#### 2-Phenyl-1-[3-(trifluoromethyl)phenyl]-1-propanone^44^ (**3i**)

Prepared following the general procedure C from compound **1i** with a reaction time of 2 h. The crude product was purified *via* column chromatography (8 g silica, LP/EtOAc 80:1) to yield 73 mg (69%) of the title compound. ^1^H NMR (400 MHz, CDCl_3_): δ = 8.21 (tt, *J* = 1.8, 0.8 Hz, 1H), 8.11–8.04 (m, 1H), 7.73–7.66 (m, 1H), 7.52–7.43 (m, 1H), 7.33–7.28 (m, 1H), 7.28–7.25 (m, 2H), 7.25–7.15 (m, 2H), 4.65 (q, *J* = 6.8 Hz, 1H), 1.54 (d, *J* = 6.8 Hz, 3H). ^13^C{^1^H} NMR (101 MHz, CDCl_3_): δ = 199.0, 140.9, 137.1, 132.0 (d, *J* = 1.5 Hz), 131.2 (q, *J* = 32.8 Hz), 129.3, 129.3 (q, *J* = 3.8 Hz), 129.2, 127.8, 127.3, 125.7 (q, *J* = 3.9 Hz), 123.0 (d, *J* = 275.8 Hz), 48.43, 19.47. HRMS (ESI): *m*/*z* [M + H]^+^ calcd for C_16_H_14_F_3_O: 279.0991; found: 279.0986

#### 1-[4-(2-Methylpropyl)phenyl]-2-phenyl-1-propanone (**3j**)

Prepared following the general procedure C from compound **1j** with a reaction time of 2 h. The crude product was purified *via* column chromatography (8 g silica, LP/EtOAc 100:1) to yield 78 mg (74%) of the title compound. ^1^H NMR (400 MHz, CDCl_3_): δ = 7.93–7.85 (m, 2H), 7.35–7.25 (m, 4H), 7.25–7.18 (m, 1H), 7.18–7.12 (m, 2H), 4.69 (q, *J* = 6.9 Hz, 1H), 2.48 (d, *J* = 7.2 Hz, 2H), 1.85 (dh, *J* = 13.4, 6.7 Hz, 1H), 1.54 (d, *J* = 6.9 Hz, 3H), 0.88 (dd, *J* = 6.6, 0.6 Hz, 6H). ^13^C{^1^H} NMR (101 MHz, CDCl_3_): δ = 200.0, 147.3, 141.7, 134.3, 129.3, 128.9, 128.8, 127.8, 126.8, 47.7, 45.4, 30.1, 22.4, 22.3, 19.6. HRMS (ESI): *m*/*z* [M + H]^+^ calcd for C_19_H_23_O: 267.1743; found: 267.1739

#### 1-(4-Methylphenyl)-2-phenyl-1-propanone^48^ (**3k**)

Prepared following the general procedure C from compound **1k** with a reaction time of 2 h. The crude product was purified *via* column chromatography (8 g silica, LP/EtOAc 70:1) to yield 82 mg (77%) of the title compound as a yellow oil. ^1^H NMR (400 MHz, CDCl_3_): δ = 7.91–7.84 (m, 2H), 7.34–7.25 (m, 4H), 7.25–7.14 (m, 3H), 4.68 (q, *J* = 6.9 Hz, 1H), 2.35 (s, 3H), 1.54 (d, *J* = 6.9 Hz, 3H). ^13^C{^1^H} NMR (101 MHz, CDCl_3_): δ = 200.0, 143.6, 141.8, 134.1, 129.3, 129.0, 129.0, 127.8, 126.9, 47.8, 21.7, 19.6. HRMS (ESI): *m*/*z* [M + H]^+^ calcd for C_16_H_17_O: 225.1274; found: 225.1252

#### 1-(2-Naphthyl)-2-phenyl-1-propanone^49^ (**3l**)

Prepared following the general procedure C from compound **1l** with a reaction time of 2 h. The crude product was purified *via* column chromatography (8 g silica, LP/EtOAc 80:1) to yield 64 mg (61%) of the title compound. ^1^H NMR (400 MHz, CDCl_3_): δ = 8.52–8.47 (m, 1H), 8.03 (dd, *J* = 8.7, 1.8 Hz, 1H), 7.90 (dd, *J* = 8.1, 1.4 Hz, 1H), 7.82 (dd, *J* = 8.6, 1.8 Hz, 2H), 7.58-7.49 (m, 2H), 7.40–7.24 (m, 4H), 7.24–7.15 (m, 1H), 4.86 (q, *J* = 6.9 Hz, 1H), 1.61 (d, *J* = 6.9 Hz, 3H). ^13^C{^1^H} NMR (101 MHz, CDCl_3_): δ = 200.4, 141.7, 135.5, 134.0, 132.6, 130.6, 129.7, 129.1, 128.5, 128.4, 127.9, 127.8, 127.0, 126.8, 124.7, 48.1, 19.7. HRMS (ESI): *m*/*z* [M + H]^+^ calcd for C_19_H_17_O: 261.1274; found: 261.1267

#### 1,2-Bis(4-methoxyphenyl)-1-propanone^50^ (**3m**)

Prepared following the general procedure C from commercially available starting material with a reaction time of 4 h. The crude product was purified *via* column chromatography (8 g silica, LP/EtOAc 10:1) to yield 76 mg (74%) of the title compound. ^1^H NMR (400 MHz, CDCl_3_): δ = 7.98–7.90 (m, 2H), 7.24–7.16 (m, 2H), 6.90–6.78 (m, 4H), 4.60 (q, *J* = 6.8 Hz, 1H), 3.81 (s, 3H), 3.75 (s, 3H), 1.49 (d, *J* = 6.9 Hz, 3H). ^13^C{^1^H} NMR (101 MHz, CDCl_3_): δ = 199.2, 163.3, 158.5, 134.1, 131.2, 129.6, 128.8, 114.5, 113.8, 55.5, 55.3, 46.7, 19.7. HRMS (ESI): *m*/*z* [M + H]^+^ calcd for C_17_H_19_O_3_: 271.1329; found: 271.1326

#### 1-(2*H*-1,3-Benzodioxol-5-yl)-2-phenyl-1-propanone^51^ (**3n**)

Prepared following the general procedure C from compound **1n** with a reaction time of 2 h. The crude product was purified *via* column chromatography (8 g silica, LP/EtOAc 80:1) to yield 65 mg (61%) of the title compound as white crystals. ^1^H NMR (400 MHz, CDCl_3_): δ = 7.57 (dd, *J* = 8.2, 1.8 Hz, 1H), 7.44 (d, *J* = 1.7 Hz, 1H), 7.34–7.22 (m, 4H), 7.22–7.15 (m, 1H), 6.76 (d, *J* = 8.2 Hz, 1H), 5.98 (s, 2H), 4.59 (q, *J* = 6.8 Hz, 1H), 1.51 (d, *J* = 6.9 Hz, 3H). ^13^C{^1^H} NMR (101 MHz, CDCl_3_): δ = 198.5, 151.6, 148.2, 141.9, 131.4, 129.1, 127.8, 127.0, 125.1, 108.7, 107.9, 101.9, 47.8, 19.7. HRMS (ESI): *m*/*z* [M + H]^+^ calcd for C_16_H_15_O_3_: 255.1016; found: 255.1006

#### 2-(3-Methoxyphenyl)-1-phenyl-1-propanone^44^ (**3o**)

Prepared following the general procedure C from compound **1o** with a reaction time of 3 h. The crude product was purified *via* column chromatography (8 g silica, LP/EtOAc 80:1) to yield 75 mg (71%) of the title compound as a yellow oil. ^1^H NMR (400 MHz, CDCl_3_): δ = 8.01–7.93 (m, 2H), 7.53–7.42 (m, 1H), 7.42–7.33 (m, 2H), 7.26–7.16 (m, 1H), 6.89 (ddd, *J* = 7.7, 1.7, 1.0 Hz, 1H), 6.84 (dd, *J* = 2.6, 1.7 Hz, 1H), 6.75 (ddd, *J* = 8.2, 2.6, 0.9 Hz, 1H), 4.66 (q, *J* = 6.8 Hz, 1H), 3.76 (s, 3H), 1.54 (d, *J* = 6.8 Hz, 3H). ^13^C{^1^H} NMR (101 MHz, CDCl_3_): δ = 200.2, 160.1, 143.1, 136.6, 132.9, 130.1, 128.8, 128.6, 120.3, 113.6, 112.2, 55.3, 48.0, 19.5. HRMS (ESI): *m*/*z* [M + H]^+^ calcd for C_16_H_17_O_2_: 241.1223; found: 241.1217

#### 1-(4-Methoxyphenyl)-2-phenyl-1-propanone^44^ (**3p**)

Prepared following the general procedure C from commercially available starting material with a reaction time of 3 h. The crude product was purified *via* column chromatography (8 g silica, LP/EtOAc 60:1, 50:1) to yield 73 mg (70%) of the title compound as a slightly yellow oil.

### Procedure for One-Pot O- and α-Methylation

Prepared following the general procedure C from commercially available 1-(4-hydroxyphenyl)-2-phenylethanone with a reaction time of 6 h. After 3 h reaction time and before the workup, another 2 equiv of PhMe_3_NI and KOH each were added at room temperature, and the reaction was subsequently heated up again to 130 °C for another 3 h. The crude product was purified *via* column chromatography (8 g silica, LP/EtOAc 70:1-50:1) to yield 95 mg (84%) of the title compound as a slightly yellow oil. Spectra were according to compound **3p**. ^1^H NMR (400 MHz, CDCl_3_): δ = 8.00–7.91 (m, 2H), 7.33–7.24 (m, 4H), 7.24–7.15 (m, 1H), 6.90–6.81 (m, 2H), 4.65 (q, *J* = 6.9 Hz, 1H), 3.81 (s, 3H), 1.52 (d, *J* = 6.9 Hz, 3H). ^13^C{^1^H} NMR (101 MHz, CDCl_3_): δ = 199.0, 163.3, 142.0, 131.2, 129.6, 129.0, 127.8, 126.9, 113.8, 55.5, 47.6, 19.7. HRMS (ESI): *m*/*z* [M + H]^+^ calcd for C_16_H_17_O_2_: 241.1223; found: 241.1236

#### 2-(4-Fluorophenyl)-1-phenyl-1-propanone^44^ (**3q**)

Prepared following the general procedure C from compound **1q** with a reaction time of 2.5 h. The crude product was purified *via* column chromatography (8 g silica, LP/EtOAc 80:1) to yield 82 mg (77%) of the title compound as a colorless oil. ^1^H NMR (400 MHz, CDCl_3_): δ = 7.98–7.91 (m, 2H), 7.54–7.45 (m, 1H), 7.44–7.34 (m, 2H), 7.31–7.21 (m, 2H), 7.04–6.93 (m, 2H), 4.70 (q, *J* = 6.9 Hz, 1H), 1.53 (d, *J* = 6.9 Hz, 3H). ^13^C{^1^H} NMR (101 MHz, CDCl_3_): δ = 200.3, 161.9 (d, *J* = 245.4 Hz), 137.2 (d, *J* = 3.3 Hz), 136.4, 133.0, 129.4 (d, *J* = 8.0 Hz), 128.8, 128.7, 115.9 (d, *J* = 21.3 Hz), 47.0, 19.7. HRMS (ESI): *m*/*z* [M + H]^+^ calcd for C_15_H_14_FO: 229.1023; found: 229.1003

#### 1-[1,1′-Biphenyl]-4-yl-2-phenyl-1-propanone^44^ (**3r**)

Prepared following the general procedure C from compound **1r** with a reaction time of 2.5 h. The crude product was purified *via* column chromatography (8 g silica, LP/EtOAc 75:1) to yield 55 mg (52%) of the title compound as an off-white solid. ^1^H NMR (400 MHz, CDCl_3_): δ = 8.09–8.02 (m, 2H), 7.65–7.55 (m, 4H), 7.48–7.36 (m, 4H), 7.36–7.28 (m, 4H), 7.28–7.19 (m, 1H), 4.74 (q, *J* = 6.8 Hz, 1H), 1.59 (d, *J* = 6.8 Hz, 3H). ^13^C{^1^H} NMR (101 MHz, CDCl_3_): δ = 199.9, 145.5, 141.6, 139.9, 135.2, 129.4, 129.1, 129.0, 128.2, 127.8, 127.3, 127.2, 127.0, 48.0, 19.6. HRMS (ESI): *m*/*z* [M + H]^+^ calcd for C_21_H_19_O: 287.1430; found: 287.1439

#### 2-Methyl-1,3-diphenyl-1,3-propanedione^52^ (**3s**)

Prepared following the general procedure C from commercially available starting material with a reaction time of 2.5 h. The crude product was purified *via* column chromatography (LP/EtOAc 80:1, 50:1, 20:1) to yield 36 mg (35%) of the title compound as white crystals. ^1^H NMR (400 MHz, CDCl_3_): δ = 8.00–7.92 (m, 4H), 7.60–7.50 (m, 2H), 7.49–7.39 (m, 4H), 5.28 (q, *J* = 7.0 Hz, 1H), 1.60 (d, *J* = 7.0 Hz, 3H). ^13^C{^1^H} NMR (100 MHz, CDCl_3_): δ = 197.3, 135.7, 133.5, 128.9, 128.6, 51.0, 14.4. HRMS (ESI): *m*/*z* [M + H]^+^ calcd for C_16_H_15_O_2_: 239.1067; found: 239.1052

#### 2-Methyl-4-phenylcyclohexanone^53^ (**3t**)

Prepared following the general procedure C from commercially available starting material with a reaction time of 18 h. The crude product was purified *via* column chromatography (8 g silica LP/EtOAc 80:1-10:1) to yield 18 mg (17%) of the title compound as a colorless oil. ^1^H NMR (400 MHz, CDCl_3_): δ = 7.33–7.23 (m, 2H), 7.23–7.14 (m, 3H), 3.10 (tt, *J* = 12.4, 3.5 Hz, 1H), 2.64–2.53 (m, 1H), 2.53–2.42 (m, 2H), 2.26–2.14 (m, 2H), 1.98–1.81 (m, 1H), 1.69–1.57 (m, 1H), 1.03 (d, *J* = 6.5 Hz, 3H). ^13^C{^1^H} NMR (101 MHz, CDCl_3_): δ = 212.7, 144.9, 128.7, 126.8, 126.7, 44.9, 43.6, 43.5, 41.7, 35.1, 14.6. HRMS (ESI): *m*/*z* [M + H]^+^ calcd for C_13_H_17_O: 189.1274; found: 189.1277

#### 5-Methyl-2-phenyl-3-hexanone^54^ (**3u**)

Prepared following the general procedure C from commercially available starting material with a reaction time of 16 h. The crude product was purified *via* flash column chromatography (15 g silica, LP/EtOAc 80:1) to yield 80 mg (77%) of the title compound as a colorless oil. ^1^H NMR (400 MHz, CDCl_3_): δ = 7.36–7.27 (m, 2H), 7.29–7.19 (m, 1H), 7.21–7.16 (m, 2H), 3.71 (q, *J* = 7.0 Hz, 1H), 2.30–2.15 (m, 2H), 2.09 (dp, *J* = 13.4, 6.6 Hz, 1H), 1.38 (d, *J* = 6.9 Hz, 3H), 0.84 (d, *J* = 6.6 Hz, 3H), 0.75 (d, *J* = 6.6 Hz, 3H). ^13^C{^1^H} NMR (100 MHz, CDCl_3_): δ = 210.5, 140.6, 128.9, 128.0, 127.2, 53.4, 50.1, 24.5, 22.7, 22.4, 17.5.

#### Methyl 4-(1-Methyl-2-oxo-2-phenylethyl)benzoate^55^ (**3v**)

Prepared following the general procedure C from compound **1v** with a reaction time of 2 h. The crude product was purified *via* flash column chromatography (15 g silica, LP/EtOAc 20:1, 10:1) to yield 34 mg (32%) of the title compound as a colorless oil. ^1^H NMR (400 MHz, CDCl_3_): δ = 8.00–7.89 (m, 4H), 7.53–7.44 (m, 1H), 7.43–7.32 (m, 4H), 4.74 (q, *J* = 6.8 Hz, 1H), 3.87 (s, 3H), 1.55 (d, *J* = 6.9 Hz, 3H) ^13^C{^1^H} NMR (100 MHz, CDCl_3_): δ = 199.79, 166.89, 146.74, 136.33, 133.17, 130.41, 128.99, 128.84, 128.71, 127.97, 52.19, 47.97, 19.42. HRMS (ESI): *m*/*z* [M + H]^+^ calcd for C_17_H_17_O_3_: 269.1172; found: 269.1196

#### Ethyl 4-(1-Methyl-2-oxo-2-phenylethyl)benzoate^51^ (**3w**)

Prepared following the general procedure C from compound **1w** with a reaction time of 2 h. The crude product was purified *via* flash column chromatography (15 g silica, LP/EtOAc 20:1, 10:1) to yield 37 mg (37%) of the title compound as a colorless oil. ^1^H NMR (400 MHz, CDCl_3_): δ = 8.01–7.95 (m, 2H), 7.95–7.89 (m, 2H), 7.52–7.44 (m, 1H), 7.43–7.32 (m, 4H), 4.74 (q, *J* = 6.9 Hz, 1H), 4.34 (q, *J* = 7.1 Hz, 2H), 1.55 (d, *J* = 6.9 Hz, 3H), 1.35 (t, *J* = 7.1 Hz, 3H). ^13^C{^1^H} NMR (100 MHz, CDCl_3_): δ = 199.79, 166.41, 146.63, 136.34, 133.14, 130.38, 129.35, 128.84, 128.70, 127.91, 61.03, 48.00, 27.90, 19.42, 14.44. HRMS (ESI): *m*/*z* [M + H]^+^ calcd for C_18_H_19_O_3_: 283.1329; found: 283.1338

#### 1-(4-Methoxyphenyl)-2-phenylethanone^56^ (**3x**)

Prepared following the general procedure C, with the deviation of using only 1 equiv of PhMe_3_NI, from commercially available starting material with a reaction time of 2 h. The crude product was purified *via* column chromatography (LP/EtOAc 70:1–50:1) to yield 68 mg (64%) of the title compound as a colorless oil. ^1^H NMR (400 MHz, CDCl_3_): δ = 8.02–7.94 (m, 2H), 7.34–7.17 (m, 5H), 6.95–6.86 (m, 2H), 4.21 (s, 2H), 3.82 (s, 3H). ^13^C{^1^H} NMR (100 MHz, CDCl_3_): δ = 196.3, 163.6, 135.1, 131.0, 129.7, 129.5, 128.7, 126.9, 113.9, 55.5, 45.3.

#### Methyl 4-(Biphenyl-4-yl)-4-oxobutanoate^57^ (**3y**)

Prepared following the general procedure C, with the deviation of using 3 equiv of KOH, from commercially available fenbufen with a reaction time of 24 h. The crude product was purified *via* column chromatography (LP/EtOAc 30:1–1:1) to yield 72 mg (68%) of the title compound as yellow crystals. ^1^H NMR (400 MHz, CDCl_3_): δ = 8.10–8.02 (m, 2H), 7.73–7.66 (m, 2H), 7.66–7.59 (m, 2H), 7.52–7.43 (m, 2H), 7.43–7.36 (m, 1H), 3.72 (s, 3H), 3.35 (t, *J* = 6.7 Hz, 2H), 2.80 (t, *J* = 6.6 Hz, 2H). ^13^C{^1^H} NMR (100 MHz, CDCl_3_): δ = 197.7, 173.5, 146.0, 139.9, 135.3, 129.1, 128.7, 128.4, 127.4, 51.9, 33.5, 28.2. HRMS (ESI): *m*/*z* [M + H]^+^ calcd for C_18_H_19_O_3_: 283.1329; found: 283.1333

#### Methyl 3-Methyl-4-oxo-4-(4-phenylphenyl)butanoate (**3z**)

An 8 mL glass vial equipped with a magnetic stirring bar was charged with fenbufen (100 mg, 1 equiv), PhMe_3_NI (2 equiv), and KOH (3 equiv). The vial was sealed with a septum screw cap. Using a cannula, the vial was evacuated and backfilled with argon three times. Anisole (2 mL, 0.2 M) was added *via* a syringe. Evacuation and backfilling with argon were repeated three times under vigorous stirring that no boiling delay occurred. Subsequently, the septum screw cap was exchanged for a closed Wheaton cap, and the vial was sealed tightly. The resulting inhomogeneous mixture was heated to 130 °C in a metallic heating block for 3 h. The reaction mixture was cooled to room temperature, and additional PhMe_3_NI (2 equiv) and KOH (2 equiv) were added. Subsequently, the reaction was heated up to 130 °C and stirred for 4 days (with further addition of 2 equiv PhMe_3_NI and 2 equiv KOH after 48 h). The reaction was cooled to room temperature. HCl (2 N, 2 mL) were added, and the mixture was extracted three times with EtOAc (20 mL each). The combined organic phases were washed twice with 2 N HCl (3 mL each) and once with brine, dried over anhydrous Na_2_SO_4_, filtered, and concentrated. The obtained crude product was purified *via* hand column with unmodified silica gel (15 g silica, LP/EtOAc 30:1–1:1), yielding 34 mg (31%) of the title compound as a colorless oil. ^1^H NMR (400 MHz, CDCl_3_): δ = 8.11–8.04 (m, 2H), 7.74–7.67 (m, 2H), 7.67–7.59 (m, 2H), 7.52–7.43 (m, 2H), 7.43–7.36 (m, 1H), 3.99 (dqd, *J* = 8.5, 7.2, 5.7 Hz, 1H), 3.66 (s, 3H), 3.00 (dd, *J* = 16.8, 8.4 Hz, 1H), 2.49 (dd, *J* = 16.8, 5.7 Hz, 1H), 1.27 (d, *J* = 7.2 Hz, 3H) ^13^C{^1^H} NMR (100 MHz, CDCl_3_): δ = 202.3, 172.9, 145.8, 140.0, 134.6, 129.1, 129.0, 128.3, 127.4, 127.3, 51.8, 37.3, 37.3, 18.0. HRMS (ESI): *m*/*z* [M + H]^+^ calcd for C_19_H_21_O_3_: 297.1485; found: 297.1490

#### 1-(4-Fluorophenyl)-2-phenyl-1-butanone^53^ (**4a**)

Prepared following the general procedure C, except for the use of PhEt_3_NI (2 equiv) instead of PhMe_3_NI, from commercially available starting material with a reaction time of 5 h. The crude product was purified *via* column chromatography (8 g silica LP/EtOAc 50:1–40:1) to yield 83 mg (78%) of the title compound. ^1^H NMR (400 MHz, CDCl_3_): δ = 7.96 (dd, *J* = 8.9, 5.4 Hz, 2H), 7.52–7.11 (m, 5H), 7.03 (dd, *J* = 9.0, 8.3 Hz, 1H), 4.36 (t, *J* = 7.2 Hz, 2H), 2.17 (dp, *J* = 12.8, 7.3 Hz, 1H), 1.83 (dp, *J* = 13.6, 7.4 Hz, 1H), 0.88 (t, *J* = 7.4 Hz, 3H). ^13^C{^1^H} NMR (101 MHz, CDCl_3_): δ = 198.6, 165.6 (d, *J* = 254.6 Hz), 139.6, 133.5, 131.4 (d, *J* = 9.2 Hz), 129.0, 128.3, 127.2, 115.7 (d, *J* = 21.8 Hz), 55.6, 27.2, 12.4 ^19^F NMR (376 MHz, CDCl_3_): δ = −105.7 HRMS (ESI): *m*/*z* [M + H]^+^ calcd for C_16_H_16_FO: 243.1180; found: 243.1186

#### 1,2-Bis(4-methoxyphenyl)-1-butanone^58^ (**4b**)

Prepared following the general procedure C, except for the use of PhEt_3_NI (2 equiv) instead of PhMe_3_NI, from commercially available starting material with a reaction time of 4 h. The crude product was purified *via* column chromatography (15 g silica LP/EtOAc 30:1–20:1) to yield 74 mg (68%) of the title compound. ^1^H NMR (400 MHz, CDCl_3_): δ = 8.00–7.91 (m, 2H), 7.25–7.17 (m, 2H), 6.90–6.78 (m, 4H), 4.35 (t, *J* = 7.3 Hz, 1H), 3.81 (s, 3H), 3.75 (s, 3H), 2.23–2.08 (m, 1H), 1.82 (dq, *J* = 13.6, 7.4 Hz, 1H), 0.89 (t, *J* = 7.4 Hz, 3H). ^13^C{^1^H} NMR (101 MHz, CDCl_3_): δ = 198.9, 163.2, 158.6, 132.2, 131.0, 130.1, 129.3, 114.3, 113.7, 55.5, 55.3, 54.2, 27.2, 12.4. HRMS (ESI): *m*/*z* [M + H]^+^ calcd for C_18_H_21_O_3_: 285.1485; found: 285.1492

#### 2-Methyl-5-phenyl-4-heptanone (**4c**)

Prepared following the general procedure C, except for the use of PhEt_3_NI (2 equiv) instead of PhMe_3_NI, from commercially available starting material with a reaction time of 18 h. The crude product was purified *via* column chromatography (15 g silica LP/Et_2_O 100:1–100:3) to yield 65 mg (57%) of the title compound as a slightly yellow oil. ^1^H NMR (400 MHz, CDCl_3_): δ = 7.38–7.30 (m, 2H), 7.29–7.24 (m, 1H), 7.24–7.18 (m, 2H), 3.51 (t, *J* = 7.4 Hz, 1H), 2.33–2.17 (m, 2H), 2.17–2.00 (m, 2H), 1.72 (dp, *J* = 13.7, 7.5 Hz, 1H), 0.89–0.80 (m, 6H), 0.76 (d, *J* = 6.6 Hz, 3H). ^13^C{^1^H} NMR (101 MHz, CDCl_3_): δ = 210.2, 139.0, 128.8, 128.4, 127.1, 61.3, 51.0, 25.3, 24.3, 22.7, 22.3, 12.2. HRMS (ESI): *m*/*z* [M + H]^+^ calcd for C_14_H_21_O: 205.1587; found: 205.1593

#### 1-(4-Fluorophenyl)-2,3-diphenylpropan-1-one^59^ (**5a**)

Prepared following the general procedure C, except for the use of BnMe_3_NCl (1.1 equiv) instead of PhMe_3_NI, from commercially available starting material with a reaction time of 1 h. The crude product was purified *via* column chromatography (15 g silica, LP/EtOAc 150:1–100:1) to yield 119 mg (84%) of the title compound as white crystals. ^1^H NMR (400 MHz, CDCl_3_): δ = 7.99–7.89 (m, 2H), 7.32–7.13 (m, 8H), 7.13–7.06 (m, 2H), 7.05–6.96 (m, 2H), 4.78 (t, *J* = 7.2 Hz, 1H), 3.58 (dd, *J* = 13.7, 7.5 Hz, 1H), 3.08 (dd, *J* = 13.7, 7.0 Hz, 1H). ^13^C{^1^H} NMR (100 MHz, CDCl_3_): δ = 197.7, 165.6 (d, *J* = 254.9 Hz), 139.7, 139.0, 133.2 (d, *J* = 3.0 Hz), 131.4, 131.3, 129.2, 129.1, 128.3 (d, *J* = 4.6 Hz), 127.3, 126.3, 115.6 (d, *J* = 21.9 Hz), 56.0, 40.2. HRMS (ESI): *m*/*z* [M + H]^+^ calcd for C_21_H_18_FO: 305.1336; found: 305.1354

#### 1,2-Bis-(4-methoxy-phenyl)-3-phenyl-propan-1-one^60^ (**5b**)

Prepared following the general procedure C, except for the use of BnMe_3_NCl (1.1 equiv) instead of PhMe_3_NI, from commercially available starting material with a reaction time of 2 h. The crude product was purified *via* column chromatography (15 g silica, LP/EtOAc 100:1, 40:1, 20:1, 10:1) to yield 121 mg (89%) of the title compound as white crystals.^1^H NMR (400 MHz, CDCl_3_): δ = 7.96–7.87 (m, 2H), 7.24–7.11 (m, 5H), 7.11–7.04 (m, 2H), 6.87–6.75 (m, 4H), 4.72 (t, *J* = 7.2 Hz, 1H), 3.79 (s, 3H), 3.75 (s, 3H), 3.53 (dd, *J* = 13.7, 7.3 Hz, 1H), 3.04 (dd, *J* = 13.7, 7.2 Hz, 1H). ^13^C{^1^H} NMR (100 MHz, CDCl_3_): δ = 198.0, 163.3, 158.6, 140.1, 131.6, 131.0, 129.8, 129.3, 129.2, 128.2, 126.1, 114.3, 113.7, 55.5, 55.2, 54.7, 40.2. HRMS (ESI): *m*/*z* [M + H]^+^ calcd for C_23_H_23_O_3_: 347.1642; found: 347.1650

#### 5-Methyl-1,2-diphenyl-3-hexanone (**5c**)

Prepared following the general procedure C, except for the use of BnMe_3_NCl (1.1 equiv) instead of PhMe_3_NI, from commercially available starting material with a reaction time of 2 h. The crude product was purified *via* column chromatography (15 g silica, LP/Et_2_O 100:1–100:3) to yield 115 mg (78%) of the title compound as white crystals. ^1^H NMR (400 MHz, CDCl_3_): δ = 7.47–7.29 (m, 7H), 7.29–7.23 (m, 1H), 7.22–7.16 (m, 2H), 4.07–3.98 (m, 1H), 3.56 (ddd, *J* = 13.7, 7.9, 1.6 Hz, 1H), 3.03 (ddd, *J* = 13.7, 6.8, 1.4 Hz, 1H), 2.29 (dt, *J* = 6.3, 1.3 Hz, 2H), 2.25–2.09 (m, 1H), 0.89 (dd, *J* = 6.6, 1.2 Hz, 3H), 0.79 (dd, *J* = 6.5, 1.2 Hz, 3H). ^13^C{^1^H} NMR (100 MHz, CDCl_3_): δ = 209.3, 139.9, 138.5, 129.1, 128.9, 128.5, 128.3, 128.0, 127.3, 127.1, 126.1, 61.3, 51.4, 38.7, 24.2, 22.6, 22.1. HRMS (ESI): *m*/*z* [M + H]^+^ calcd for C_19_H_23_O: 267.1743; found: 267.1755

## Supporting Information Available

Detailed procedure for quantitative ^19^F NMR measurements, complete optimization screening data, experimental procedures, and characterization data for all compounds isolated (PDF)
