# Supplementary material for: Selective α-Methylation of Aryl Ketones Using Quaternary Ammonium Salts as Solid Methylating Agents
Source: J Org Chem. 2022 Mar 7;87(6):4305–15. doi: 10.1021/acs.joc.1c03158 (PMC8938946; doi:10.1021/acs.joc.1c03158)
Supplement: Supplementary file 1 — jo1c03158_si_001.pdf [file jo1c03158_si_001.pdf]

*Supporting Information*

# Selective α-Methylation of Aryl Ketones using Quaternary Ammonium salts as solid Methylating Agents

Johanna Templ, Michael Schnürch*

Institute of Applied Synthetic Chemistry, TU Wien, Getreidemarkt 9/163, 1060 Wien, Austria
E-mail: : michael.schnuerch@tuwien.ac.at

# Table of Contents


General Experimental Details ..... S2

General Procedures ..... S2

- Optimization Screening ..... S2
- Precursor Synthesis ..... S4
- Substrate Scope Methylation ..... S4

Optimization Screening for Methylation Reaction ..... S5

- Solvent ..... S5
- Base ..... S5
- Reaction Time ..... S6
  - Studies for interconversion between *O*- and α-methylated product ..... S6
- Ammonium Salt ..... S7
- Heating *via* microwave irradiation ..... S8

Characterization data for all synthetic compounds ..... S9

- Precursor Synthesis ..... S9
- Substrate Scope Methylation ..... S13

NMR-Spectra ..... S25

References ..... S72

# General Experimental Details

All Chemicals were purchased from commercial suppliers and, unless noted otherwise, used without further purification. NaO$^t$Bu, $Pd_2(dba)_3$, and DPE-Phos were strictly stored and handled in the glove box under argon atmosphere. Degassed and dry THF was stored over molecular sieves under argon using AcroSeal™ septum. The 8 mL glass vials were sealed with Wheaton® screw caps containing a PTFE faced 14B styrene-butadiene rubber liner for small-scale reaction above room temperature and heated in a metallic reaction block. All reaction temperatures refer to external temperatures.

$^1$H-NMR, $^{13}$C-NMR, and $^{19}$F-NMR spectra were recorded on a Bruker Avance UltraShield 400 at ambient temperature. Chemical Shifts (δ) are reported in ppm, using $Me_4Si$ as internal standard. Coupling constants (*J*) are given in Hertz (Hz) and multiplicities are assigned as s = singlet, d = doublet, t = triplet, q = quartet, and m = multiplet.
Quantitative $^{19}$F-NMR spectra were recorded in a non-decoupled mode with a prolonged relaxation delay (d1 = 20 s), a narrowed spectral width (SW = 70 ppm), and a modified transmitter excitation frequency (O1T) to place the center of the spectrum between the peaks of interest (for details see Optimization Screening).

Thin Layer Chromatography (TLC) analysis was performed on aluminum-backed unmodified Merck silica gel 60 $F_{245}$ plates. Visualization was realized under UV irradiation or *via* heat staining using a ceric ammonium molybdate aqueous solution. For flash column chromatography, Merck silica gel 60 (40 µm – 63 µm) was used, and purification was either done by hand-column or on a Büchi® Pure C-850 FlashPrep System.

GC-MS analysis was carried out on a Thermo Finnigan Focus GC/DSQ II with a standard capillary column RXi-5Sil MS column (30 m, 0.25 mm ID, 0.25 µm df) using the following standardized temperature program: 2 min at 100 °C, 35 °C/min until 300 °C, 4 min at 300 °C.

HR-MS analysis was performed on an Agilent 6230 LC TOFMS mass spectrometer equipped with an Agilent Dual AJS ESI-Source. The mass spectrometer was connected to a liquid chromatography system of the 1100/1200 series from Agilent Technologies, Palo Alto, CA, USA. The system consisted of a 1200SL binary gradient pump, a degasser, a column thermostat, and an HTC PAL autosampler (CTC Analytics AG, Zwingen, Switzerland). A silica-based Phenomenex C-18 Security Guard Cartridge was used as a stationary phase. Data evaluation was performed using Agilent MassHunter Qualitative Analysis B.07.00. Identification was based on peaks obtained from extracted ion chromatograms (extraction width ± 20 ppm).

# General Procedures

## Optimization Screening

### General Procedure A:

An 8 mL glass vial equipped with a magnetic stirring bar was charged with benzyl 4-fluorophenyl ketone **(1a)** (50 mg, 0.233 mmol, 1 equiv.), the desired ammonium salt (350 mmol, 1.5 equiv. *or 0.467 mmol, 2 equiv.*) and the base (0.467 mmol, 2 equiv.). The vial was sealed with a septum screw cap. Using a cannula, the vial was evacuated and backfilled with argon three times. The solvent (1

mL, 0.23 M) was added *via* a syringe. Evacuation and backfilling with argon were repeated three times under vigorous stirring that no boiling delay occurred. Subsequently, the septum screw cap was exchanged for a closed Wheaton® cap, and the vial was sealed tightly. The resulting inhomogeneous mixture was heated to 100 °C (for low boiling solvents) or 130 °C in a metallic heating block. After 18 – 22 h at respective temperatures, the reaction was cooled to room temperature.

**Sample preparation for quant. $^{19}$F-NMR:**
100 µL of a solution of trifluorotoluene in $CHCl_3$ (0.49 mol/mL) was added to the reaction mixture *via* Eppendorf® pipette. The inhomogeneous mixture was centrifuged, and 0.5 mL of the supernatant solution were transferred to an NMR tube. 0.3 mL $CDCl_3$ were added to the NMR tube, and the liquid content was homogenized thoroughly.

**Quant. $^{19}$F-NMR instrument parameters and processing:**
NMR spectra were shimmed for $CDCl_3$ and recorded with the following changes in acquisition parameters:

- transmitter excitation frequency (O1T) = -87 ppm
- spectral width = 70 ppm
- relaxation delay = 20 s

After standard Fourier transformation, the recorded spectra were processed by MestReNova v12 software as following[1]:

- Apodization along t1: exponential 0.50 Hz
- Zero filling along t1: 512K
- Auto Phase Correction (Algorithms: Global, Selective, Metabonomics, Whitening, Min. Entropy, Baseline Optimization, Regions Analysis; Initial Phase: Zero)
- Auto Baseline Correction along t1: Ablative (5 Points, 10 Passes)

The following $^{19}$F-NMR should serve as an example spectrum used for evaluation.

2a

3a

1a

*Figure S1. example spectrum for quantitative $^{19}$F-NMR used for evaluation*

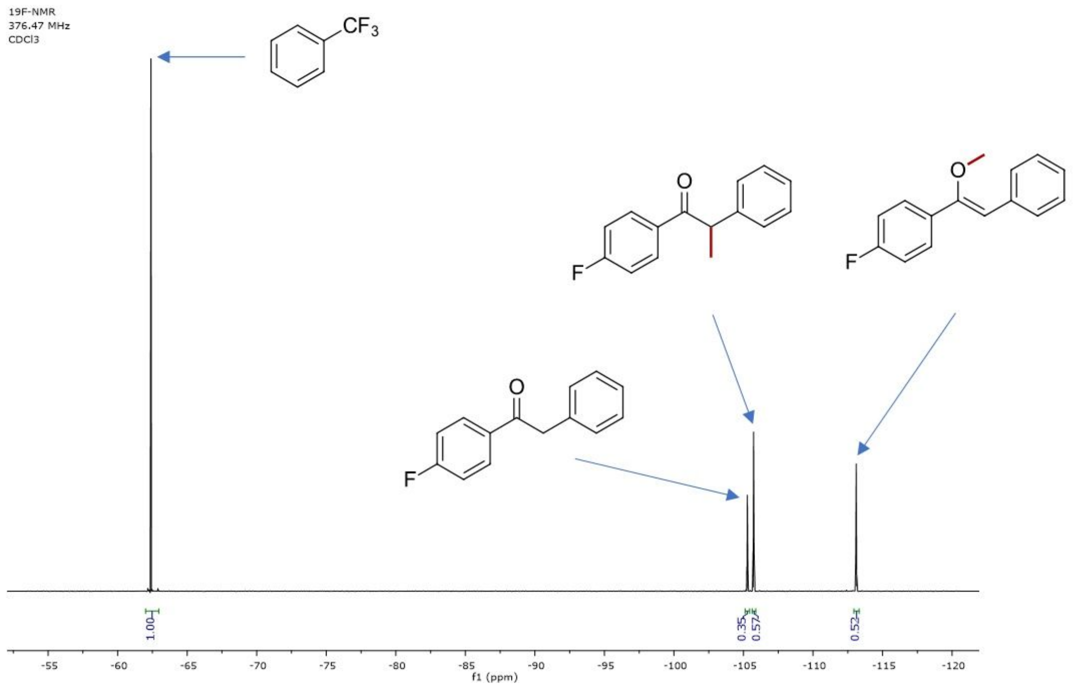

## Precursor Synthesis

The α-monoarylated acetophenones **1g-l, 1n, 1o, 1q, 1r, 1v, 1w** were synthesized *via* Heck-type coupling of the acetophenone enolates and the respective aryl bromides, according to literature.[2]

**General Procedure B:**

In the glove box, a flame dried 8 mL glass vial equipped with a magnetic stirring bar was charged with NaO$^t$Bu (2.6 mmol, 1.3 equiv.), $Pd_2(dba)_3$ (5 mol-%) and DPE-Phos (10 mol-%). THF (2 mL, 1 M) was added, and the dark brownish-green mixture was stirred for 5 minutes at ambient temperatures. The aryl bromide (2 mmol, 1 equiv.) was added *via* Eppendorf® pipette, followed by rapid addition of the acetophenone (2.4 mmol, 1.2 equiv.) in one portion as solid or *via* Eppendorf® pipette if liquid. Immediate solid formation could be observed. The vial was closed with a Wheaton® screw cap and transferred out of the glove box. The mixture was heated to 70 °C in a metallic reaction block and stirred for 2-18 h at respective temperatures. After complete consumption of the starting material (GC-MS monitoring), water (10 mL) was added, and the mixture was extracted three times with diethyl ether (30 mL each). The combined organic phases were washed once with sat. $NH_4Cl$ solution and once with brine, dried over anhydrous $Na_2SO_4$, filtered and concentrated. The crude product was purified *via* gradient flash column chromatography on silica gel using a mixture of LP and EtOAc.

## Substrate Scope Methylation

**General Procedure C:**

An 8 mL glass vial equipped with a magnetic stirring bar was charged with the respective diaryl ethanone (100 mg, 1 equiv.), trimethylphenyl ammonium iodide (2 equiv.), and KOH (2 equiv.). The vial was sealed with a septum screw cap. Using a cannula, the vial was evacuated and backfilled with argon three times. Anisole (2 mL, 0.2 M) was added *via* syringe. Evacuation and backfilling with argon were repeated three times under vigorous stirring that no boiling delay occurred. Subsequently, the septum screw cap was exchanged for a closed Wheaton® cap, and the vial was sealed tightly. The resulting inhomogeneous mixture was heated to 130 °C in a metallic heating block for 2-4 h. After complete consumption of the starting material (TLC analysis), the reaction was cooled to room temperature. 2 mL of 2 N HCl were added, and the mixture was extracted 3 times with EtOAc (5 mL each). The combined organic phases were washed twice with 2 N HCl (1 mL each) and once with brine, dried over anhydrous $Na_2SO_4$, filtered, and concentrated. The obtained crude product was purified *via* hand column with unmodified silica gel using a mixture of LP and EtOAc as eluent.

# Optimization Screening for Methylation Reaction

Yields, determined by quant. $^{19}F$-NMR, for the depicted reaction, are shown in the tables below. The following parameters were screened:

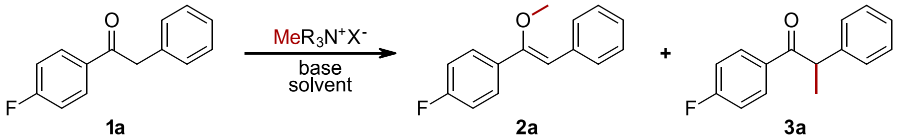


## Solvent

Reactions were performed following general procedure A using $Me_4N^+Br^-$ (0.350 mmol, 1.5 equiv.) as methylating agent and KOH (0.467 mmol, 2 equiv.) as base with 1 mL solvent (0.23 M) at respective temperatures for 22 h.

- toluene [CAS: 108-88-3] at 130 °C
- MeTHF [CAS:  96-47-9] at 100 °C
- anisole [CAS: 100-66-3] at 130 °C
- CPME [CAS: 5614-37-9] at 130 °C
- EtOAc [CAS: 141-78-6] at 100 °C
- *γ*-valerolactone [CAS: 108-29-2] at 130 °C
- sulfolane [CAS: 126-33-0] at 130 °C
- $NPr_3$ [CAS: 102-69-2] at 130 °C
- *t*-BuOH [CAS: 75-65-0] at 100 °C
- pyridine [CAS: 110-86-1] at 130 °C

*Table S1. Solvent Screening*

| entry | variation | yield (%) | | |
|---|---|---|---|---|
| | solvent | **1a** | **2a** | **3a** |
| 1 | toluene | 0 | 41 | 34 |
| 2 | MeTHF | 25 | 14 | 11 |
| 3 | anisole | 0 | 40 | 43 |
| 4 | CPME | 4 | 36 | 24 |
| 5 | EtOAc | 95 | 0 | 0 |
| 6 | γ-valerolactone | 94 | 0 | 0 |
| 7 | sulfolane | 8 | 4 | 4 |
| 8 | $NPr_3$ | 10 | 4 | 4 |
| 9 | *t*-BuOH | 38 | 6 | 6 |
| 10 | pyridine | 0 | 33 | 22 |

## Base

Reactions were performed following the general procedure A using $Me_4N^+Br^-$ (0.350 mmol, 1.5 equiv.) as methylating agent, 0.467 mmol (2 equiv.) of the respective base and anisole (1 mL, 0.23 M) as solvent at 130 °C for 18 h.

- $LiOH \cdot H_2O$ [CAS: 1310-66-3]

- NaOH [CAS: 1310-73-2]
- KOH [CAS: 1310-58-3]
- LiO$^t$Bu [CAS: 1907-33-1]
- KO$^t$Bu [CAS: 865-47-4]
- $Cs_2CO_3$ [CAS: 534-17-8]
- $K_2CO_3$ [CAS: 584-08-7]
- imidazole [CAS: 288-32-4]

*Table S2. Base Screening*

| entry | variation | yield (%) | | |
|---|---|---|---|---|
| | base | **1a** | **2a** | **3a** |
| 1 | no base | 82 | 0 | 0 |
| 2 | $LiOH \cdot H_2O$ | 99 | 0 | 0 |
| 3 | NaOH | 22 | 32 | 36 |
| 4 | KOH | 2 | 42 | 44 |
| 5 | LiO$^t$Bu | - | - | - |
| 6 | KO$^t$Bu | 8 | 32 | 22 |
| 7 | $Cs_2CO_3$ | 41 | 26 | 27 |
| 8 | $K_2CO_3$ | 94 | 0 | 0 |
| 9 | imidazole | 92 | 0 | 0 |

## Reaction Time

Reactions were performed following general procedure A using $Me_4N^+Br^-$ (0.350 mmol, 1.5 equiv.) as methylating agent, KOH (0.467 mmol, 2 equiv.) as base, and anisole (1 mL, 0.23 M) as solvent at 130 °C.

*Table S3. Reaction Time Screening*

| entry | variation | yield (%) | | |
|---|---|---|---|---|
| | time [min] | **1a** | **2a** | **3a** |
| 1 | 2 | 76 | 0 | 0 |
| 2 | 5 | 64 | 7 | 7 |
| 3 | 10 | 43 | 14 | 17 |
| 4 | 15 | 42 | 20 | 21 |
| 5 | 20 | 21 | 31 | 34 |
| 6 | 25 | 17 | 35 | 37 |
| 7 | 30 | 6 | 39 | 41 |
| 8 | 35 | 2 | 38 | 41 |
| 9 | 40 | 2 | 39 | 41 |
| 10 | 45 | 2 | 40 | 42 |
| 11 | 50 | 2 | 39 | 42 |
| 12 | 55 | 0 | 41 | 43 |
| 13 | 60 | 0 | 40 | 43 |

## Studies for interconversion between *O*- and α-methylated product

Reactions were performed following general procedure A using $Me_4N^+Br^-$ (0.187 mmol, 2 equiv.) as methylating agent, KOH (0.187 mmol, 2 equiv.) as base, and anisole (0.4 mL, 0.23 M) as solvent at 130 °C for 1 h. The vials were charged with the following substrates:

- entry 1:
  benzyl 4-fluorophenyl ketone **(1a)** (0.093 mmol)
- entry 2:
  benzyl 4-fluorophenyl ketone **(1a)** (0.047 mmol), 1-Fluoro-4-(1-methoxy-2-phenylethenyl)benzene **(2a)** (0.044 mmol)
- entry 3:
  1-Fluoro-4-(1-methoxy-2-phenylethenyl)benzene **(2a)** (0.088 mmol)
- entry 4:
  benzyl 4-fluorophenyl ketone **(1a)** (0.047 mmol), 1-Fluoro-4-(1-methoxy-2-phenylethenyl)benzene **(2a)** (0.044 mmol)

*Table S4. Screening for Independency of Product Formations*

| entry | substrate | | yield (%) | | |
|---|---|---|---|---|---|
| | **1a** [mmol] | **2a** [mmol] | **1a** | **2a** | **3a** |
| 1 | 0.093 | - | 0 | 42 | 43 |
| 2 | 0.047 | 0.044 | 0 | 73 | 9 |
| 3 | - | 0.088 | 0 | 86 | 0 |
| 4 | 0.047 | 0.044 | 30 | 44 | 0 |

## Ammonium Salt

Reactions were performed following the general procedure A using KOH (0.467 mmol, 2 equiv.) as base, 0.350 mmol (1.5 equiv.) of the respective ammonium salt and anisole (1 mL, 0.23 M) as solvent at 130 °C for 18 h.

- $Me_4NCl$ [CAS: 75-57-0]
- $Me_4NBr$ [CAS: 64-20-0]
- $Me_4NI$ [CAS: 75-58-1]
- $Me_3PhNCl$ [CAS: 138-24-9]
- $Me_3PhNBr$ [CAS: 16056-11-4]
- $Me_3PhNI$ [CAS: 98-04-4]
- $Bu_3MeNCl$ [CAS: 56375-79-2]
- $BnMe_3NCl$ [CAS: 56-93-9]
- $(C_{16}H_{33})Me_3NBr$ [CAS: 57-09-0]
- betaine [CAS: 107-43-7]

*Table S5. Ammonium Salt Screening*

| entry | variation | yield (%) | | |
|---|---|---|---|---|
| | ammonium salt | **1a** | **2a** | **3a** |
| 1 | $Me_4NCl$ | 0 | 43 | 42 |
| 2 | $Me_4NBr$ | 0 | 44 | 44 |

| | | | | |
|---|---|---|---|---|
| 3 | $Me_4NI$ | 30 | 23 | 29 |
| 4 | $Me_4NOAc$ | 0 | 49 | 9 |
| 5 | $PhMe_3NCl$ | 0 | 47 | 48 |
| 6 | $PhMe_3NBr$ | 0 | 38 | 50 |
| 7 | $PhMe_3NI$ | 0 | 18 | 78 |
| 8 | $Bu_3MeNCl$ | 6 | 25 | 39 |
| 9 | $BnMe_3NCl$ | 0 | 6 | 4 |
| 10 | $(C_{16}H_{33})Me_3NBr$ | 0 | 47 | 25 |
| 11 | betaine | 30 | 2 | 5 |

## Heating *via* microwave irradiation

An 8 mL round bottom microwave vial equipped with a magnetic stirring bar was charged with benzyl 4-fluorophenyl ketone **(1a)** (50 mg, 0.233 mmol, 1 equiv.), $PhMe_3NI$ (125 mg, 467 mmol, 2 equiv.) and KOH (26 mg, 0.467 mmol, 2 equiv.). The vial was sealed with a septum. Using a cannula, the vial was evacuated and backfilled with argon three times. The solvent (1 mL, 0.23 M) was added *via* a syringe. Evacuation and backfilling with argon were repeated three times under vigorous stirring that no boiling delay occurred. Subsequently, the septum screw cap was exchanged for a microwave vial septum cap, and the vial was sealed tightly. The resulting inhomogeneous mixture was heated to 110, 100, 90, and 80 °C, respectively, in a microwave oven. After 1 h the reaction was cooled to room temperature, and each sample was prepared for quant. $^{19}F$-NMR following the general procedure A.

*Table S6. Temperature Screening via Microwave Irradiation*

| entry | variation | yield (%) | | |
|---|---|---|---|---|
| | temperature [°C] | **1a** | **2a** | **3a** |
| 1 | 110 | 0 | 11 | 76 |
| 2 | 100 | 6 | 12 | 64 |
| 3 | 90 | 7 | 22 | 62 |
| 4 | 80 | 48 | 13 | 21 |

# Characterization data for all synthetic compounds

All compounds synthesized are described in the literature, except **3d** and **3z**. For known compounds, spectral data is in agreement with the literature.

## Precursor Synthesis

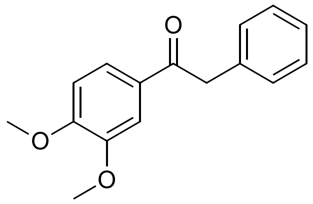

**1-(3,4-Dimethoxyphenyl)-2-phenylethanone[3] (1g) [CAS: 3141-93-3]**

Prepared, following the general procedure B from 3,4-dimethoxyacetophenone and bromobenzene heated for 2 h. The crude product was purified *via* flash column chromatography (90 g silica, LP, and EtOAc 0-40 %) to yield 443 mg (86 %) of the title compound as a slightly yellow oil.

$\mathbf{R_f}$ = 0.47 (LP:EtOAc 2:1)

**$^1$H-NMR** (400 MHz, $CDCl_3$): δ = 7.68 (dd, *J* = 8.4, 2.1 Hz, 1H), 7.58 (d, *J* = 2.1 Hz, 1H), 7.39 – 7.22 (m, 5H), 6.89 (d, *J* = 8.4 Hz, 1H), 4.26 (s, 2H), 3.95 (s, 3H), 3.93 (s, 3H).

**$^{13}$C{$^1$H}-NMR** (100 MHz, $CDCl_3$): δ = 196.3, 153.3, 149.1, 135.1, 129.7, 129.3, 128.6, 126.8, 123.5, 110.7, 110.0, 56.0, 55.9, 45.2.

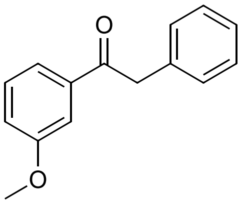

**1-(3-Methoxyphenyl)-2-phenylethanone[2] (1h) [CAS: 62381-24-2]**

Prepared, following the general procedure B from 3-methoxyacetophenone and bromobenzene heated for 3 h. The crude product was purified *via* flash hand-column chromatography (60 g silica, LP:EtOAc 70:1, 60:1, 40:1) to yield 305 mg (67%) of the title compound as a colorless oil.

$\mathbf{R_f}$ = 0.30 (LP:EtOAc 5:1)

**$^1$H-NMR** (400 MHz, $CDCl_3$): δ = 7.65 – 7.58 (m, 1H), 7.55 (dd, *J* = 2.7, 1.6 Hz, 1H), 7.43 – 7.31 (m, 3H), 7.31 – 7.26 (m, 3H), 7.11 (ddd, *J* = 8.2, 2.7, 0.9 Hz, 1H), 4.28 (s, 2H), 3.84 (s, 3H).

**$^{13}$C{$^1$H}-NMR** (100 MHz, $CDCl_3$): δ = 197.5, 159.9, 138.0, 134.6, 129.6, 129.5, 128.7, 126.9, 121.3, 119.7, 112.9, 55.4, 45.7.

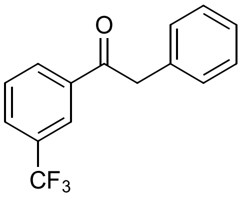

**2-Phenyl-1-[3-(trifluormethyl)phenyl]ethanone[4] (1i) [CAS: 1533-04-6]**

Prepared, following the general procedure B from 3-trifluoromethylacetophenone and bromobenzene heated for 4 h. The crude product was purified *via* flash column chromatography (90 g silica, LP, and EtOAc 0-15 %) to yield 391 mg (74 %) of the title compound as orange oil.

$\mathbf{R_f}$ = 0.36 (LP:EtOAc 5:1)

**$^1$H-NMR** (400 MHz, $CDCl_3$): δ = 8.26 (tt, *J* = 1.8, 0.8 Hz, 1H), 8.16 (dt, *J* = 7.3, 1.1 Hz, 1H), 7.81 – 7.70 (m, 1H), 7.56 (tt, *J* = 7.9, 0.8 Hz, 1H), 7.38 – 7.29 (m, 2H), 7.29 – 7.18 (m, 3H), 4.29 (s, 2H).

**$^{13}$C{$^1$H}-NMR** (100 MHz, $CDCl_3$): δ = 196.3, 137.1, 133.9, 131.9 (d, *J* = 1.4 Hz), 131.2 (q, *J* = 34.0 Hz), 129.6 (q, *J* = 3.6 Hz), 129.5, 129.4, 128.9, 127.3, 125.5 (q, *J* = 3.8 Hz), 123.7 (d, *J* = 274.8 Hz), 45.70.

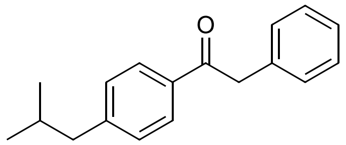

**1-[4-(2-Methylpropyl)phenyl]-2-phenylethanone (1j) [CAS: 60312-92-7]**

Prepared, following the general procedure B from 4'-Isobutylacetophenone and bromobenzene heated for 4 h. The crude product was purified *via* flash column chromatography (90 g silica, LP, and EtOAc 0-20 %) to yield 415 mg (82 %) of the title compound as a yellow oil.

**$R_f$ =** 0.45 (LP:EtOAc 5:1)

**$^1$H-NMR** (400 MHz, $CDCl_3$): δ = 8.03 – 7.96 (m, 2H), 7.42 – 7.23 (m, 7H), 4.30 (s, 2H), 2.57 (d, *J* = 7.2 Hz, 2H), 2.04 – 1.86 (m, *J* = 6.9 Hz, 1H), 0.96 (d, *J* = 6.7 Hz, 6H).

**$^{13}$C{$^1$H}-NMR** (100 MHz, $CDCl_3$): δ = 197.2, 147.6, 134.8, 134.4, 129.5, 129.3, 128.6, 126.8, 45.3, 30.1, 22.3.

---

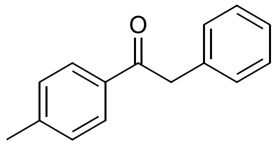

**1-(4-Methylphenyl)-2-phenylethanone[5] (1k) [CAS: 2001-28-7]**

Prepared, following the general procedure B from 4-Methylacetophenone and bromobenzene heated for 3 h. The crude product was purified *via* flash hand-column chromatography (55 g silica, LP:EtOAc 80:1, 70:1, 60:1, 40:1) to yield 302 mg (72 %) of the title compound as a colorless oil.

**$R_f$ =** 0.40 (LP:EtOAc 5:1)

**$^1$H-NMR** (400 MHz, $CDCl_3$): δ = 7.94 (d, *J* = 7.9 Hz, 2H), 7.36 – 7.24 (m, 7H), 4.28 (s, 2H), 2.42 (s, 3H).

**$^{13}$C{$^1$H}-NMR** (100 MHz, $CDCl_3$): δ = 197.4, 144.1, 134.9, 134.2, 129.5, 129.4, 128.8, 128.7, 126.9, 45.5, 21.7.

---

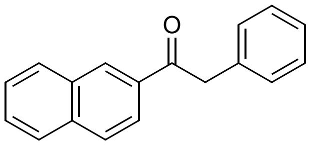

**1-(2-Naphthalenyl)-2-phenylethanone[5] (1l) [CAS: 1762-15-8]**

Prepared, following the general procedure B from 2-acetylnaphthalene and bromobenzene heated for 18 h. The crude product was purified *via* flash column chromatography (90 g silica, LP, and EtOAc 0-40 %) to yield 396 mg (80 %) of the title compound as an off-white solid.

**$R_f$ =** 0.34 (LP:EtOAc 5:1)

**$^1$H-NMR** (400 MHz, $CDCl_3$): δ = 8.47 (d, *J* = 1.8 Hz, 1H), 8.00 (dd, *J* = 8.6, 1.8 Hz, 1H), 7.88 (dd, *J* = 8.1, 1.4 Hz, 1H), 7.85 – 7.73 (m, 2H), 7.57 – 7.41 (m, 2H), 7.32 – 7.15 (m, 5H), 4.34 (s, 2H).

**$^{13}$C{$^1$H}-NMR** (100 MHz, $CDCl_3$): δ = 197.6, 135.6, 134.7, 134.0, 132.5, 130.4, 129.7, 129.5, 128.7, 128.6, 128.6, 127.8, 126.9, 126.8, 124.3, 45.6.

---

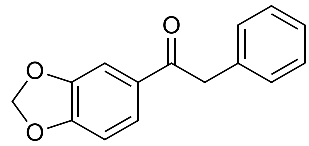

**1-(1,3-Benzodioxol-5-yl)-2-phenylethanone[5] (1n) [CAS: 126266-77-1]**

Prepared, following the general procedure B from 5-acetyl-1,3-benzodiocole and bromobenzene heated for 10 h. The crude product was purified *via* flash column chromatography (90 g silica, LP and EtOAc 0-40 %) to yield 471 mg (98%) of the title compound as a slightly yellow oil.

**$R_f$ =** 0.27 (LP:EtOAc 5:1)

**$^1$H-NMR** (400 MHz, $CDCl_3$): δ = 7.64 (dd, *J* = 8.2, 1.8 Hz, 1H), 7.49 (d, *J* = 1.7 Hz, 1H), 7.39 – 7.29 (m, 2H), 7.29 – 7.21 (m, 3H), 6.85 (d, *J* = 8.2 Hz, 1H), 6.03 (s, 2H), 4.21 (s, 2H).

**$^{13}$C{$^1$H}-NMR** (100 MHz, $CDCl_3$): δ = 195.8, 151.9, 148.3, 134.9, 131.5, 129.4, 128.7, 128.7, 126.9, 125.1, 108.8, 108.4, 107.9, 101.9, 45.4.

---

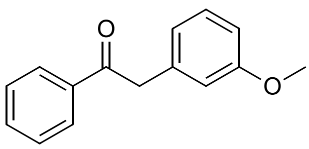


**2-(3-Methoxyphenyl)-1-phenylethanone[5] (1o) [CAS: 29955-26-8]**

Prepared, following the general procedure B from acetophenone and 4-bromoanisole heated for 18 h. The crude product was purified *via* flash column chromatography (90 g silica, LP, and EtOAc 0-40 %) to yield 267 mg (59 %) of the title compound as a yellow oil.

**$R_f$ =** 0.32 (LP:EtOAc 5:1)

**$^1$H-NMR** (400 MHz, $CDCl_3$): δ = 8.06 - 7.97 (m, 2H), 7.60 – 7.51 (m, 1H), 7.51 – 7.41 (m, 2H), 7.25 (t, *J* = 7.8 Hz, 1H), 6.91 – 6.77 (m, 3H), 4.26 (s, 2H), 3.79 (s, 3H).

**$^{13}$C{$^1$H}-NMR** (101 MHz, $CDCl_3$): δ = 197.5, 159.8, 136.6, 136.1, 133.2, 129.7, 128.7, 121.9, 115.2, 112.4, 55.2, 45.6.

---

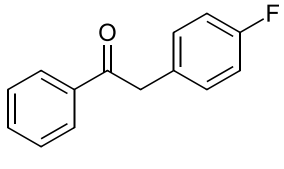


**2-(4-Fluorphenyl)-1-phenylethanone[2] (1q) [CAS: 347-91-1]**

Prepared, following the general procedure B from acetophenone and 1-bromo-4-fluorobenzene heated for 18 h. The crude product was purified *via* flash column chromatography (90 g silica, LP, and EtOAc 0-40 %) to yield 255 mg (60 %) of the title compound as a slightly yellow oil.

**$R_f$ =** 0.31 (LP:EtOAc 5:1)

**$^1$H-NMR** (400 MHz, $CDCl_3$): δ = 8.06 – 7.94 (m, 2H), 7.62 – 7.52 (m, 1H), 7.52 – 7.39 (m, 2H), 7.28 – 7.19 (m, 2H), 7.08 – 6.97 (m, 2H), 4.27 (s, 2H).

**$^{13}$C{$^1$H}-NMR** (100 MHz, $CDCl_3$): δ = 197.5, 162.0 (d, *J* = 245.2 Hz), 136.6, 133.4, 131.2 (d, *J* = 8.0 Hz), 130.3 (d, *J* = 3.3 Hz), 128.8, 128.6, 115.6 (d, *J* = 21.4 Hz), 44.6.

---

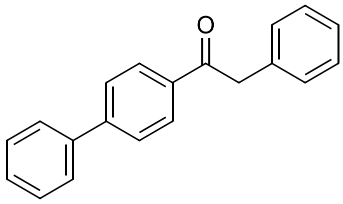


**1-[1,1’-Biphenyl]-4-yl-2-phenylethanone[5] (1r) [CAS: 2001-23-2]**

Prepared, following the general procedure B from 4’-phenylacetophenone and bromobenzene heated for 18 h. The crude product was purified *via* flash column chromatography (90 g silica, LP, and EtOAc 0-40 %) to yield 207 mg (38 %) of the title compound as a colorless oil.

**$R_f$ =** 0.31 (LP:EtOAc 5:1)

**$^1$H-NMR** (400 MHz, $CDCl_3$): δ = 8.12 – 8.04 (m, 2H), 7.70 – 7.64 (m, 2H), 7.64 – 7.59 (m, 2H), 7.50 – 7.43 (m, 2H), 7.43 – 7.22 (m, 6H), 4.31 (s, 2H).

**$^{13}$C{$^1$H}-NMR** (100 MHz, $CDCl_3$): δ = 197.2, 145.8, 139.8, 135.3, 134.7, 129.5, 129.3, 129.0, 128.7, 128.3, 127.3, 127.3, 126.9, 45.6.

---

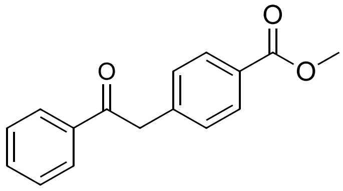


**Methyl 4-(2-oxo-2-phenylethyl)benzoate[6] (1v) [CAS: 94161-45-2]**

Prepared, following the general procedure B from acetophenone methyl 4-iodobenzoate heated for 4 h. The crude product was

purified *via* flash column chromatography (90 g silica, LP, and EtOAc 0-20 %) to yield 285 mg (37 %) of the title compound as a white solid.

**$R_f$ =** 0.58 (LP:EtOAc 1:1)

**$^1$H-NMR** (400 MHz, $CDCl_3$): δ = 8.04 – 7.98 (m, 4H), 7.62 – 7.54 (m, 1H), 7.51 – 7.44 (m, 2H), 7.37 – 7.32 (m, 2H), 4.35 (s, 2H), 3.90 (s, 3H).

**$^{13}$C{$^1$H}-NMR** (100 MHz, $CDCl_3$): δ = 196.9, 167.0, 139.9, 136.5, 133.5, 130.0, 129.7, 129.0, 128.8, 128.6, 77.4, 52.2, 45.5.

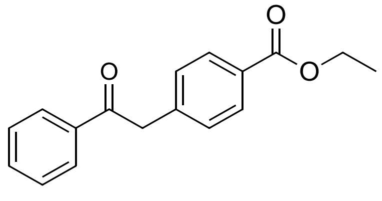

**Ethyl 4-(2-oxo-2-phenylethyl)benzoate[7] (1w) [CAS: 898776-62-0]**

Prepared, following the general procedure B from acetophenone ethyl 4-iodobenzoate heated for 4 h. The crude product was purified *via* flash column chromatography (90 g silica, LP, and EtOAc 0-20 %) to yield 360 mg (45 %) of the title compound as a white solid.

**$R_f$ =** 0.55 (LP:EtOAc 1:1)

**$^1$H-NMR** (400 MHz, $CDCl_3$): δ = 8.04 – 7.97 (m, 4H), 7.62 – 7.53 (m, 1H), 7.51 – 7.42 (m, 2H), 7.37 – 7.31 (m, 2H), 4.41 – 4.31 (m, 4H), 1.38 (t, J = 7.1 Hz, 3H)

**$^{13}$C{$^1$H}-NMR** (100 MHz, $CDCl_3$): δ = 196.9, 166.5, 139.8, 136.5, 133.5, 130.0, 129.6, 128.8, 128.6, 61.0, 45.5, 14.4.

## Substrate Scope Methylation

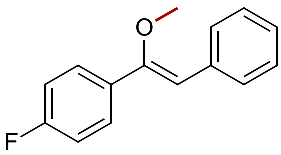

**1-Fluoro-4-(1-methoxy-2-phenylethenyl)benzene (2a)**[8] **[CAS 874394-40-8]**

An 8 mL glass vial equipped with a magnetic stirring bar was charged with benzyl 4-fluorophenyl ketone **(1a)** (100 mg, 0.467 mmol, 1 equiv.), $Me_4NBr$ (119 mg, 770 mmol, 1.65 equiv.) and KOH (79 mg, 1.4 mmol, 3 equiv.). The vial was sealed with a septum screw cap. Using a cannula, the vial was evacuated and backfilled with argon three times. The toluene (2 mL, 0.23 M) was added *via* syringe. Evacuation and backfilling with argon were repeated three times under vigorous stirring that no boiling delay occurred. Subsequently, the septum screw cap was exchanged for a closed Wheaton® cap, and the vial was sealed tightly. The resulting inhomogeneous mixture was heated to 130 °C in a metallic heating block. After 18 h at respective temperatures, the reaction was cooled to room temperature, and solids were centrifuged off. The supernatant solution was transferred to a round bottom flask, and the solid residue was washed three times with small amounts DCM. The combined organic phases were concentrated. The crude oil was further purified *via* hand-column chromatography (8 g silica LP:$Et_3N$ 100:1) to yield 46 mg (43 %) of the title compound as white crystals. NMR shifts indicate mainly Z-isomer formation.

**$R_f$ =** 0.57 (LP:EtOAc 5:1)

**$^1$H-NMR** (400 MHz, $CDCl_3$): δ = 7.74 – 7.67 (m, 2H), 7.60 – 7.50 (m, 2H), 7.42 – 7.32 (m, 2H), 7.28 – 7.19 (m, 1H), 7.16 – 7.03 (m, 2H), 6.06 (s, 1H), 3.63 (s, 3H).

**$^{13}$C{$^1$H}-NMR** (101 MHz, $CDCl_3$): δ = 163.0 (d, *J* = 247.9 Hz), 155.4, 135.9, 132.6 (d, *J* = 3.3 Hz), 128.7, 128.6, 128.5 (d, *J* = 8.1 Hz), 126.8, 115.6 (d, *J* = 21.7 Hz), 112.8 (d, *J* = 1.4 Hz), 58.0.

**$^{19}$F-NMR** (376 MHz, $CDCl_3$): δ = -113.2

**HRMS** (ESI): *m/z* $[M+H]^+$ calcd for $C_{15}H_{14}FO$: 229.1023; found: 229.1000

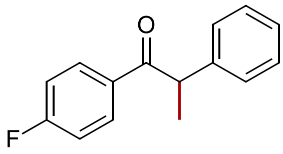

**1-(4-Fluorophenyl)-2-phenyl-1-propanone (3a)**[9] **[CAS: 49660-97-1]**

Prepared, following the general procedure C from commercially available starting material with a reaction time of 3 h. The crude product was purified *via* column chromatography (8 g silica LP:EtOAc 50:1, 45:1, 40:1) to yield 83 mg (78 %) of the title compound.

**$R_f$ =** 0.46 (LP:EtOAc 5:1)

**$^1$H-NMR** (400 MHz, $CDCl_3$): δ = 7.97 – 7.87 (m, 2H), 7.28 – 7.18 (m, 4H), 7.18 – 7.11 (m, 1H), 7.03 – 6.93 (m, 2H), 4.57 (q, *J* = 6.8 Hz, 1H), 1.48 (d, *J* = 6.9 Hz, 3H).

**$^{13}$C{$^1$H}-NMR** (101 MHz, $CDCl_3$): δ = 198.8, 165.6 (d, *J* = 254.6 Hz), 141.5, 133.0 (d, *J* = 3.0 Hz), 131.5 (d, *J* = 9.3 Hz), 129.20, 127.8, 127.1, 115.7 (d, *J* = 21.7 Hz), 48.1, 19.6.

**$^{19}$F-NMR** (376 MHz, $CDCl_3$): δ = -105.60.

**HRMS** (ESI): *m/z* $[M+H]^+$ calcd for $C_{15}H_{14}FO$: 229.1023; found: 229.1000

Compound **3a** was also prepared on a 1.4 mmol scale as follows:
A 25 mL round bottom flask was charged with benzyl 4-fluorophenyl ketone **(1a)** (300 mg, 1.4 mmol, 1 equiv.), $PhMe_3NI$ (751 mg, 2.8 mmol, 2 equiv.) and KOH (157 mg, 2.8 mmol, 2 equiv). The flask was closed with a septum. Using a cannula, the flask was evacuated and backfilled with argon three times. Anisole (6 mL, 0.23 M) was added *via* syringe. Evacuation and backfilling with argon were repeated three times under vigorous stirring that no boiling delay occurred. The resulting inhomogeneous mixture was heated to 130 °C in an oil bath. After 5 h at respective temperatures, the reaction was cooled to room temperature. 10 mL of 2 N HCl were added, and the mixture was extracted 3 times with EtOAc (25 mL each). The combined organic phases were washed twice with 2 N HCl (3-5 mL each) and once with brine, dried over anhydrous $Na_2SO_4$, filtered, and concentrated. The obtained crude product was purified *via* flash column chromatography (90 g silica, LP, and EtOAc 0-40 %) to yield 273 mg (85 %) of the title compound as a colorless oil. Analytical data were in accordance with the previous finding.

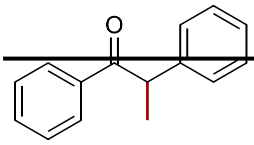


**1,2-Diphenyl-1-propanone[2] (3b) [CAS: 2024-85-5]**

Prepared, following the general procedure C from commercially available starting material with a reaction time of 3 h. The crude product was purified *via* column chromatography (8 g silica, LP:EtOAc 50:1, 40:1) to yield: 73 mg (68 %) of the title compound as a colorless oil.

**$R_f$** = 0.35 (LP:EtOAc 5:1)

**$^1$H-NMR** (400 MHz, $CDCl_3$): δ = 8.01 – 7.93 (m, 2H), 7.53 – 7.43 (m, 1H), 7.43 – 7.34 (m, 2H), 7.30 (d, *J* = 4.3 Hz, 4H), 7.21 (ddd, *J* = 8.8, 4.8, 3.9 Hz, 1H), 4.70 (q, *J* = 6.9 Hz, 1H), 1.55 (d, *J* = 6.9 Hz, 3H).

**$^{13}$C{$^1$H}-NMR** (101 MHz, $CDCl_3$): δ = 200.4, 141.6, 136.6, 132.9, 129.1, 128.9, 128.6, 127.9, 127.0, 48.0, 19.6.

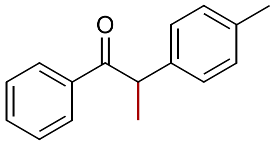


**2-(4-Methylphenyl)-1-phenyl-1-propanone[10] (3c) [CAS: 107271-15-8]**

Prepared, following the general procedure C from commercially available starting material with a reaction time of 2 h. The crude product was purified *via* column chromatography (8 g silica, LP:EtOAc 50:1) to yield 76 mg (74 %) of the title compound as a slightly yellow oil.

**$R_f$** = 0.39 (LP:EtOAc 5:1)

**$^1$H-NMR** (400 MHz, $CDCl_3$): δ = 8.01 – 7.94 (m, 2H), 7.52 – 7.43 (m, 1H), 7.43 – 7.34 (m, 2H), 7.23 – 7.16 (m, 2H), 7.15 – 7.08 (m, 2H), 4.67 (q, *J* = 6.8 Hz, 1H), 2.30 (s, 3H), 1.54 (d, *J* = 6.9 Hz, 3H).

**$^{13}$C{$^1$H}-NMR** (101 MHz, $CDCl_3$): δ = 200.5, 138.6, 136.64, 136.6, 132.8, 129.8, 128.9, 128.6, 127.7, 47.6, 21.1, 19.6.

**HRMS** (ESI): *m/z* $[M+H]^+$ calcd for $C_{16}H_{17}O$: 225.1274; found: 225.1265

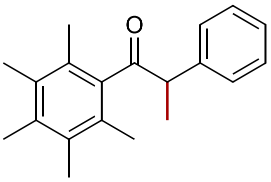


**2-(2,3,4,5,6-Pentamethylphenyl)-1-phenyl-1-propanone (3d)**

Prepared, following the general procedure C from commercially available starting material with a reaction time of 3.5 h. The crude product was purified *via* column chromatography (8 g silica, LP:EtOAc 50:1) to yield 31 mg (31 %) of the title compound as off-white crystals.

**$R_f$** = 0.47 (LP:EtOAc 5:1)

**$^1$H-NMR** (400 MHz, $CDCl_3$): δ = 7.32 – 7.16 (m, 5H), 4.13 (q, *J* = 7.0 Hz, 1H), 2.23 (s, 15H), 1.64 (d, *J* = 7.0 Hz, 3H).

**$^{13}$C{$^1$H}-NMR** (101 MHz, $CDCl_3$): δ = 211.1, 140.1, 138.7, 135.5, 132.8, 128.8, 128.5, 127.1, 54.9, 16.9, 16.8, 16.0. [10]

**HRMS** (ESI): *m/z* $[M+H]^+$ calcd for $C_{20}H_{25}O$: 281.1900; found: 281.1895

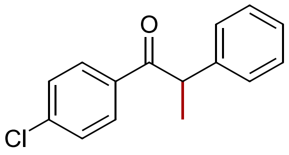


**1-(4-Chlorophenyl)-2-phenyl-1-propanone[11] (3e) [CAS: 126866-24-8]**

Prepared, following the general procedure C from commercially available starting material with a reaction time of 2 h. The crude product was purified *via* column chromatography (8 g silica, LP:EtOAc 50:1) to yield 87 mg (85%) of the title compound.

**$R_f$ =** 0.51 (LP:EtOAc 5:1)

**$^1$H-NMR** (400 MHz, $CDCl_3$): δ = 7.92 – 7.84 (m, 2H), 7.38 – 7.16 (m, 7H), 4.62 (q, *J* = 6.8 Hz, 1H), 1.53 (d, *J* = 6.8 Hz, 3H).

**$^{13}$C{$^1$H}-NMR** (101 MHz, $CDCl_3$): δ = 199.1, 141.3, 139.3, 134.9, 130.3, 129.2, 128.9, 127.8, 127.2, 48.2, 19.5.

**HRMS** (ESI): *m/z* $[M+H]^+$ calcd for $C_{15}H_{14}ClO$: 245.0728; found: 245.0712

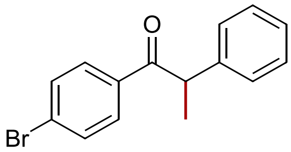


**1-(4-Bromophenyl)-2-phenyl-1-propanone[11] (3f) [CAS: 1133798-25-0]**

Prepared, following the general procedure C from commercially available starting material with a reaction time of 2 h. The crude product was purified *via* column chromatography (8 g silica, LP:EtOAc 50:1) to yield 75 mg (74 %) of the title compound.

**$R_f$ =** 0.48 (LP:EtOAc 5:1)

**$^1$H-NMR** (400 MHz, $CDCl_3$): δ = 7.84 – 7.76 (m, 2H), 7.56 – 7.46 (m, 2H), 7.36 – 7.25 (m, 3H), 7.25 – 7.16 (m, 2H), 4.61 (q, *J* = 6.8 Hz, 1H), 1.53 (d, *J* = 6.8 Hz, 3H).

**$^{13}$C{$^1$H}-NMR** (101 MHz, $CDCl_3$): δ = 199.3, 141.3, 135.2, 131.9, 130.4, 129.2, 128.0, 127.8, 127.2, 48.2, 19.5.

**HRMS** (ESI): *m/z* $[M+H]^+$ calcd for $C_{15}H_{14}BrO$: 289.0223; found: 289.0218

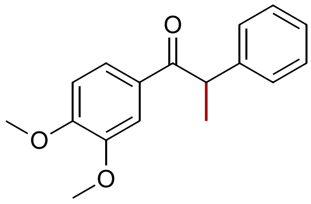


**1-(3,4-Dimethoxyphenyl)-2-phenyl-1-propanone[12] (3g) [CAS: 144053-89-4]**

Prepared, following the general procedure C from compound **1g** with a reaction time of 2 h. The crude product was purified *via* column chromatography (8 g silica, LP:EtOAc 40:1, 20:1, 10:1) to yield 72 mg (68 %) of the title compound.

**$R_f$ =** 0.54 (LP:EtOAc 2:1)

**$^1$H-NMR** (400 MHz, $CDCl_3$): δ = 7.59 (dd, *J* = 8.4, 2.0 Hz, 1H), 7.53 (d, *J* = 2.0 Hz, 1H), 7.29 (d, *J* = 4.4 Hz, 4H), 7.19 (ddd, *J* = 8.6, 4.9, 3.9 Hz, 1H), 6.80 (d, *J* = 8.5 Hz, 1H), 4.65 (q, *J* = 6.9 Hz, 1H), 3.88 (d, *J* = 4.1 Hz, 6H), 1.52 (d, *J* = 6.8 Hz, 3H).

**$^{13}C\{^1H\}$-NMR** (101 MHz, $CDCl_3$): δ = 199.0, 153.1, 149.0, 142.2, 129.7, 129.1, 127.7, 126.9, 123.5, 111.1, 110.0, 56.1, 56.0, 47.6, 19.7.

**HRMS** (ESI): *m/z* $[M+H]^+$ calcd for $C_{17}H_{19}O_3$: 271.1329; found: 271.1323

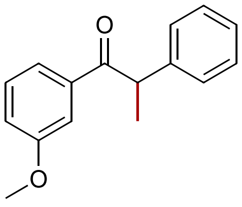

**1-(3-Methoxyphenyl)-2-phenyl-1-propanone[13] (3h) [CAS: 77669-94-4]**

Prepared, following the general procedure C from compound **1h** with a reaction time of 2 h. The crude product was purified *via* column chromatography (8 g silica, LP:EtOAc 70:1) to yield 88 mg (83 %) of the title compound as a slightly orange oil.

**$R_f$** = 0.32 (LP:EtOAc 5:1)

**$^1H$-NMR** (400 MHz, $CDCl_3$): δ = 7.51 (ddd, *J* = 7.7, 1.6, 1.0 Hz, 1H), 7.47 (dd, *J* = 2.7, 1.6 Hz, 1H), 7.34 – 7.21 (m, 5H), 7.21 – 7.12 (m, 1H), 6.99 (ddd, *J* = 8.3, 2.7, 1.0 Hz, 1H), 4.64 (q, *J* = 6.9 Hz, 1H), 3.76 (s, 3H), 1.51 (d, *J* = 6.9 Hz, 3H).

**$^{13}C\{^1H\}$-NMR** (101 MHz, $CDCl_3$): δ = 200.2, 159.8, 141.6, 138.0, 129.5, 129.1, 127.8, 127.0, 121.5, 119.4, 113.2, 55.4, 48.1, 19.6.

**HRMS** (ESI): *m/z* $[M+H]^+$ calcd for $C_{16}H_{17}O_2$: 241.1223; found: 241.1205

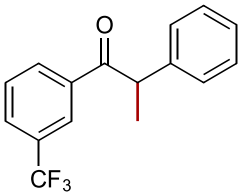

**2-Phenyl-1-[3-(trifluoromethyl)phenyl]-1-propanone[10] (3i) [CAS: 1776098-79-3]**

Prepared, following the general procedure C from compound **1i** with a reaction time of 2 h. The crude product was purified *via* column chromatography (8 g silica, LP:EtOAc 80:1) to yield 73 mg (69 %) of the title compound.

**$R_f$** = 0.40 (LP:EtOAc 5:1)

**$^1H$-NMR** (400 MHz, $CDCl_3$): δ = 8.21 (tt, *J* = 1.8, 0.8 Hz, 1H), 8.11 – 8.04 (m, 1H), 7.73 – 7.66 (m, 1H), 7.52 – 7.43 (m, 1H), 7.33 – 7.28 (m, 1H), 7.28 – 7.25 (m, 2H), 7.25 – 7.15 (m, 2H), 4.65 (q, *J* = 6.8 Hz, 1H), 1.54 (d, *J* = 6.8 Hz, 3H).

**$^{13}C\{^1H\}$-NMR** (101 MHz, $CDCl_3$): δ = 199.0, 140.9, 137.1, 132.0 (d, *J* = 1.5 Hz), 131.2 (q, *J* = 32.8 Hz), 129.3, 129.3 (q, *J* = 3.8 Hz), 129.2, 127.8, 127.3, 125.7 (q, *J* = 3.9 Hz), 123.0 (d, *J* = 275.8 Hz), 48.43, 19.47.

**HRMS** (ESI): *m/z* $[M+H]^+$ calcd for $C_{16}H_{14}F_3O$: 279.0991; found: 279.0986

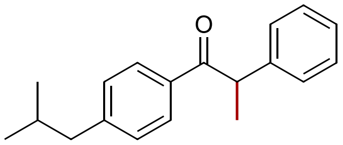

**1-[4-(2-Methylpropyl)phenyl]-2-phenyl-1-propanone (3j) [CAS: 1933951-19-9]**

Prepared, following the general procedure C from compound **1j** with a reaction time of 2 h. The crude product was purified *via* column chromatography (8 g silica, LP:EtOAc 100:1) to yield: 78 mg (74 %) of the title compound.

**$R_f$** = 0.48 (LP:EtOAc 5:1)

**$^1H$-NMR** (400 MHz, $CDCl_3$): δ = 7.93 – 7.85 (m, 2H), 7.35 – 7.25 (m, 4H), 7.25 – 7.18 (m, 1H), 7.18 – 7.12 (m, 2H), 4.69 (q, *J* = 6.9 Hz, 1H), 2.48 (d, *J* = 7.2 Hz, 2H), 1.85 (dh, *J* = 13.4, 6.7 Hz, 1H), 1.54 (d, *J* = 6.9 Hz, 3H), 0.88 (dd, *J* = 6.6, 0.6 Hz, 6H).

**$^{13}$C{$^1$H}-NMR** (101 MHz, $CDCl_3$): δ = 200.0, 147.3, 141.7, 134.3, 129.3, 128.9, 128.8, 127.8, 126.8, 47.7, 45.4, 30.1, 22.4, 22.3, 19.6.

**HRMS** (ESI): *m/z* $[M+H]^+$ calcd for $C_{19}H_{23}O$: 267.1743; found: 267.1739

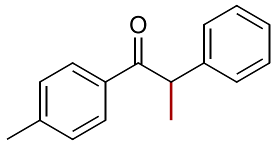

**1-(4-Methylphenyl)-2-phenyl-1-propanone[14] (3k) [CAS: 14161-82-1]**

Prepared, following the general procedure C from compound **1k** with a reaction time of 2 h. The crude product was purified *via* column chromatography (8 g silica, LP:EtOAc 70:1) to yield: 82 mg (77 %) of the title compound as a yellow oil.

**$R_f$** = 0.47 (LP:EtOAc 5:1)

**$^1$H-NMR** (400 MHz, $CDCl_3$): δ = 7.91 – 7.84 (m, 2H), 7.34 – 7.25 (m, 4H), 7.25 – 7.14 (m, 3H), 4.68 (q, *J* = 6.9 Hz, 1H), 2.35 (s, 3H), 1.54 (d, *J* = 6.9 Hz, 3H).

**$^{13}$C{$^1$H}-NMR** (101 MHz, $CDCl_3$): δ = 200.0, 143.6, 141.8, 134.1, 129.3, 129.0, 129.0, 127.8, 126.9, 47.8, 21.7, 19.6.

**HRMS** (ESI): *m/z* $[M+H]^+$ calcd for $C_{16}H_{17}O$: 225.1274; found: 225.1252

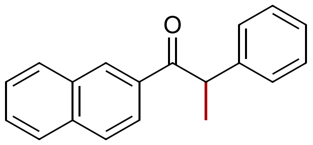

**1-(2-Naphthyl)-2-phenyl-1-propanone[15] (3l) [CAS: 113487-89-1]**

Prepared, following the general procedure C from compound **1l** with a reaction time of 2 h. The crude product was purified *via* column chromatography (8 g silica, LP:EtOAc 80:1) to yield: 64 mg (61 %) of the title compound.

**$R_f$** = 0.39 (LP:EtOAc 5:1)

**$^1$H-NMR** (400 MHz, $CDCl_3$): δ = 8.52 – 8.47 (m, 1H), 8.03 (dd, *J* = 8.7, 1.8 Hz, 1H), 7.90 (dd, *J* = 8.1, 1.4 Hz, 1H), 7.82 (dd, *J* = 8.6, 1.8 Hz, 2H), 7.58-7.49 (m, 2H), 7.40 – 7.24 (m, 4H), 7.24 – 7.15 (m, 1H), 4.86 (q, *J* = 6.9 Hz, 1H), 1.61 (d, *J* = 6.9 Hz, 3H).

**$^{13}$C{$^1$H}-NMR** (101 MHz, $CDCl_3$): δ = 200.4, 141.7, 135.5, 134.0, 132.6, 130.6, 129.7, 129.1, 128.5, 128.4, 127.9, 127.8, 127.0, 126.8, 124.7, 48.1, 19.7.

**HRMS** (ESI): *m/z* $[M+H]^+$ calcd for $C_{19}H_{17}O$: 261.1274; found: 261.1267

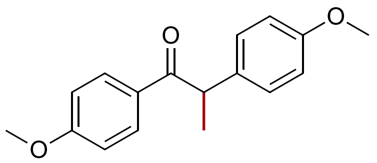

**1,2-Bis(4-methoxyphenyl)-1-propanone[16] (3m) [CAS: 35258-41-4]**

Prepared, following the general procedure C from commercially available starting material with a reaction time of 4 h. The crude product was purified *via* column chromatography (8 g silica, LP:EtOAc 10:1) to yield 76 mg (74 %) of the title compound.

**$R_f$** = 0.20 (LP:EtOAc 5:1)

**$^1$H-NMR** (400 MHz, $CDCl_3$): δ = 7.98 – 7.90 (m, 2H), 7.24 – 7.16 (m, 2H), 6.90 – 6.78 (m, 4H), 4.60 (q, *J* = 6.8 Hz, 1H), 3.81 (s, 3H), 3.75 (s, 3H), 1.49 (d, *J* = 6.9 Hz, 3H).

**$^{13}$C{$^1$H}-NMR** (101 MHz, $CDCl_3$): δ = 199.2, 163.3, 158.5, 134.1, 131.2, 129.6, 128.8, 114.5, 113.8, 55.5, 55.3, 46.7, 19.7.

**HRMS** (ESI): *m/z* $[M+H]^+$ calcd for $C_{17}H_{19}O_3$: 271.1329; found: 271.1326

### 1-(2H-1,3-Benzodioxol-5-yl)-2-phenyl-1-propanone[17] (3n)

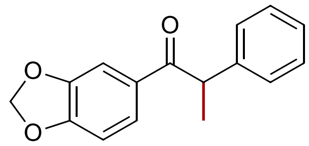

Prepared, following the general procedure C from compound **1n** with a reaction time of 2 h. The crude product was purified *via* column chromatography (8 g silica, LP:EtOAc 80:1) to yield 65 mg (61 %) of the title compound as white crystals.

**$R_f$** = 0.32 (LP:EtOAc 5:1)

**$^1$H-NMR** (400 MHz, $CDCl_3$): δ = 7.57 (dd, *J* = 8.2, 1.8 Hz, 1H), 7.44 (d, *J* = 1.7 Hz, 1H), 7.34 – 7.22 (m, 4H), 7.22 – 7.15 (m, 1H), 6.76 (d, *J* = 8.2 Hz, 1H), 5.98 (s, 2H), 4.59 (q, *J* = 6.8 Hz, 1H), 1.51 (d, *J* = 6.9 Hz, 3H).

**$^{13}$C{$^1$H}-NMR** (101 MHz, $CDCl_3$): δ = 198.5, 151.6, 148.2, 141.9, 131.4, 129.1, 127.8, 127.0, 125.1, 108.7, 107.9, 101.9, 47.8, 19.7.

**HRMS** (ESI): *m/z* $[M+H]^+$ calcd for $C_{16}H_{15}O_3$: 255.1016; found: 255.1006

---

### 2-(3-Methoxyphenyl)-1-phenyl-1-propanone[10] (3o) [CAS: 197640-99-6]

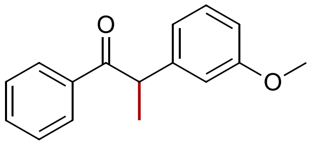

Prepared, following the general procedure C from compound **1o** with a reaction time of 3 h. The crude product was purified *via* column chromatography (8 g silica, LP:EtOAc 80:1) to yield 75 mg (71 %) of the title compound as a yellow oil.

**$R_f$** = 0.35 (LP:EtOAc 5:1)

**$^1$H-NMR** (400 MHz, $CDCl_3$): δ = 8.01 – 7.93 (m, 2H), 7.53 – 7.42 (m, 1H), 7.42 – 7.33 (m, 2H), 7.26 – 7.16 (m, 1H), 6.89 (ddd, *J* = 7.7, 1.7, 1.0 Hz, 1H), 6.84 (dd, *J* = 2.6, 1.7 Hz, 1H), 6.75 (ddd, *J* = 8.2, 2.6, 0.9 Hz, 1H), 4.66 (q, *J* = 6.8 Hz, 1H), 3.76 (s, 3H), 1.54 (d, *J* = 6.8 Hz, 3H).

**$^{13}$C{$^1$H}-NMR** (101 MHz, $CDCl_3$): δ = 200.2, 160.1, 143.1, 136.6, 132.9, 130.1, 128.8, 128.6, 120.3, 113.6, 112.2, 55.3, 48.0, 19.5.

**HRMS** (ESI): *m/z* $[M+H]^+$ calcd for $C_{16}H_{17}O_2$: 241.1223; found: 241.1217

---

### 1-(4-Methoxyphenyl)-2-phenyl-1-propanone[10] (3p) [CAS: 35258-38-9]

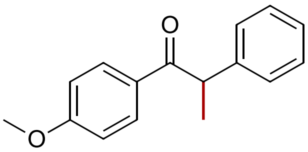

Prepared, following the general procedure C from commercially available starting material with a reaction time of 3 h. The crude product was purified *via* column chromatography (8 g silica, LP:EtOAc 60:1, 50:1) to yield 73 mg (70 %) of the title compound as a slightly yellow oil.

**Procedure for one-pot *O*- and *α*-methylation:** Prepared, following the general procedure C from commercially available 1-(4-hydroxyphenyl)-2-phenylethanone with a reaction time of 6 h. After 3 h reaction time and before the workup, another 2 equiv of $PhMe_3NI$ and KOH each were added at room temperature, and the reaction was subsequently heated up again to 130 °C for another 3 h. The crude product was purified *via* column chromatography (8 g silica, LP:EtOAc 70:1-50:1) to yield 95 mg (84 %) of the title compound as a slightly yellow oil. Spectra were according to compound **3p**.

**$R_f$** = 0.30 (LP:EtOAc 5:1)

**$^1$H-NMR** (400 MHz, $CDCl_3$): δ = 8.00 – 7.91 (m, 2H), 7.33 – 7.24 (m, 4H), 7.24 – 7.15 (m, 1H), 6.90 – 6.81 (m, 2H), 4.65 (q, *J* = 6.9 Hz, 1H), 3.81 (s, 3H), 1.52 (d, *J* = 6.9 Hz, 3H).

**$^{13}$C-NMR** (101 MHz, $CDCl_3$): δ = 199.0, 163.3, 142.0, 131.2, 129.6, 129.0, 127.8, 126.9, 113.8, 55.5, 47.6, 19.7.

**HRMS** (ESI): *m/z* $[M+H]^+$ calcd for $C_{16}H_{17}O_2$: 241.1223; found: 241.1236

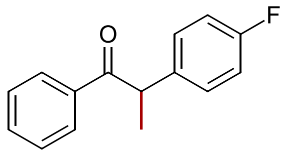

**2-(4-Fluorophenyl)-1-phenyl-1-propanone[10] (3q) [CAS: 413615-59-5]**

Prepared, following the general procedure C from compound **1q** with a reaction time of 2.5 h. The crude product was purified *via* column chromatography (8 g silica, LP:EtOAc 80:1) to yield: 82 mg (77 %) of the title compound as a colorless oil.

**$R_f$ =** 0.39 (LP:EtOAc 5:1)

**$^1$H-NMR** (400 MHz, $CDCl_3$): δ = 7.98 – 7.91 (m, 2H), 7.54 – 7.45 (m, 1H), 7.44 – 7.34 (m, 2H), 7.31 – 7.21 (m, 2H), 7.04 – 6.93 (m, 2H), 4.70 (q, *J* = 6.9 Hz, 1H), 1.53 (d, *J* = 6.9 Hz, 3H).

**$^{13}$C{$^1$H}-NMR** (101 MHz, $CDCl_3$): δ = 200.3, 161.9 (d, *J* = 245.4 Hz), 137.2 (d, *J* = 3.3 Hz), 136.4, 133.0, 129.4 (d, *J* = 8.0 Hz), 128.8, 128.7, 115.9 (d, *J* = 21.3 Hz), 47.0, 19.7.

**HRMS** (ESI): *m/z* $[M+H]^+$ calcd for $C_{15}H_{14}FO$: 229.1023; found: 229.1003

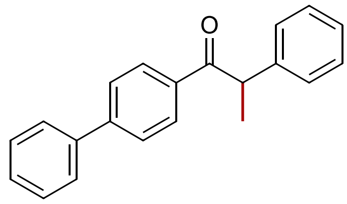

**1-[1,1'-Biphenyl]-4-yl-2-phenyl-1-propanone[10] (3r) [CAS: 187676-86-4]**

Prepared, following the general procedure C from compound **1r** with a reaction time of 2.5 h. The crude product was purified *via* column chromatography (8 g silica, LP:EtOAc 75:1) to yield: 55 mg (52 %) of the title compound as an off-white solid.

**$R_f$ =** 0.40 (LP:EtOAc 5:1)

**$^1$H-NMR** (400 MHz, $CDCl_3$): δ = 8.09 – 8.02 (m, 2H), 7.65 – 7.55 (m, 4H), 7.48 – 7.36 (m, 4H), 7.36 – 7.28 (m, 4H), 7.28-7.19 (m, 1H), 4.74 (q, *J* = 6.8 Hz, 1H), 1.59 (d, *J* = 6.8 Hz, 3H).

**$^{13}$C{$^1$H}-NMR** (101 MHz, $CDCl_3$): δ = 199.9, 145.5, 141.6, 139.9, 135.2, 129.4, 129.1, 129.0, 128.2, 127.8, 127.3, 127.2, 127.0, 48.0, 19.6.

**HRMS** (ESI): *m/z* $[M+H]^+$ calcd for $C_{21}H_{19}O$: 287.1430; found: 287.1439

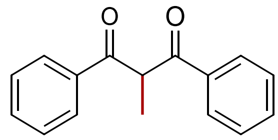

**2-Methyl-1,3-diphenyl-1,3-propanedione[18] (3s) [CAS: 1846-29-3]**

Prepared, following the general procedure C from commercially available starting material with a reaction time of 2.5 h. The crude product was purified *via* column chromatography (LP:EtOAc 80:1, 50:1, 20:1) to yield 36 mg (35 %) of the title compound as white crystals.

**$R_f$ =** 0.15 (LP:EtOAc 5:1)

**$^1$H-NMR** (400 MHz, $CDCl_3$): δ = 8.00 – 7.92 (m, 4H), 7.60 – 7.50 (m, 2H), 7.49 – 7.39 (m, 4H), 5.28 (q, *J* = 7.0 Hz, 1H), 1.60 (d, *J* = 7.0 Hz, 3H).

**$^{13}$C{$^1$H}-NMR** (100 MHz, $CDCl_3$): δ = 197.3, 135.7, 133.5, 128.9, 128.6, 51.0, 14.4.

**HRMS** (ESI): m/z $[M+H]^+$ calcd for $C_{16}H_{15}O_2$: 239.1067; found: 239.1052

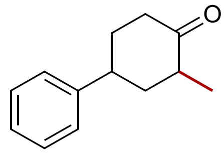

**2-Methyl-4-phenylcyclohexanone[19] (3t) [CAS: 88958-99-0]**

Prepared, following the general procedure C from commercially available starting material with a reaction time of 18 h. The crude product was purified *via* column chromatography (8 g silica LP:EtOAc 80:1-10:1) to yield 18 mg (17 %) of the title compound as a colorless oil.

**$R_f$** = 0.42 (LP:EtOAc 5:1)

**$^1$H-NMR** (400 MHz, $CDCl_3$): δ = 7.33 – 7.23 (m, 2H), 7.23 – 7.14 (m, 3H), 3.10 (tt, *J* = 12.4, 3.5 Hz, 1H), 2.64 – 2.53 (m, 1H), 2.53 – 2.42 (m, 2H), 2.26 – 2.14 (m, 2H), 1.98 – 1.81 (m, 1H), 1.69 – 1.57 (m, 1H), 1.03 (d, *J* = 6.5 Hz, 3H).

**$^{13}$C{$^1$H}-NMR** (101 MHz, $CDCl_3$): δ = 212.7, 144.9, 128.7, 126.8, 126.7, 44.9, 43.6, 43.5, 41.7, 35.1, 14.6.

**HRMS** (ESI): *m/z* $[M+H]^+$ calcd for $C_{13}H_{17}O$: 189.1274; found: 189.1277

---

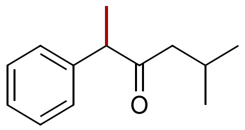

**5-Methyl-2-phenyl-3-hexanone[20] (3u) [CAS: 103392-14-9]**

Prepared, following the general procedure C from commercially available starting material with a reaction time of 16 h. The crude product was purified *via* flash column chromatography (15 g silica, LP:EtOAc 80:1) to yield 80 mg (77 %) of the title compound as a colorless oil.

**$R_f$ =** 0.44 (LP:EtOAc 5:1)

**$^1$H-NMR** (400 MHz, $CDCl_3$): δ = 7.36 – 7.27 (m, 2H), 7.29 – 7.19 (m, 1H), 7.21 – 7.16 (m, 2H), 3.71 (q, *J* = 7.0 Hz, 1H), 2.30 – 2.15 (m, 2H), 2.09 (dp, *J* = 13.4, 6.6 Hz, 1H), 1.38 (d, *J* = 6.9 Hz, 3H), 0.84 (d, *J* = 6.6 Hz, 3H), 0.75 (d, *J* = 6.6 Hz, 3H).

**$^{13}$C{$^1$H}-NMR** (100 MHz, $CDCl_3$): δ = 210.5, 140.6, 128.9, 128.0, 127.2, 53.4, 50.1, 24.5, 22.7, 22.4, 17.5.

---

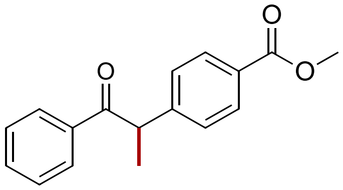

**Methyl 4-(1-methyl-2-oxo-2-phenylethyl)benzoate[21] (3v) [CAS: 255836-39-6]**

Prepared, following the general procedure C from compound **1v** with a reaction time of 2 h. The crude product was purified *via* flash column chromatography (15 g silica, LP:EtOAc 20:1, 10:1) to yield 34 mg (32 %) of the title compound as a colorless oil.

**$R_f$ =** 0.36 (LP:EtOAc 1:3)

**$^1$H-NMR** (400 MHz, $CDCl_3$): δ = 8.00 – 7.89 (m, 4H), 7.53 – 7.44 (m, 1H), 7.43 – 7.32 (m, 4H), 4.74 (q, J = 6.8 Hz, 1H), 3.87 (s, 3H), 1.55 (d, J = 6.9 Hz, 3H)

**$^{13}$C{$^1$H}-NMR** (100 MHz, $CDCl_3$): δ = 199.7, 166.8, 146.7, 136.3, 133.1, 130.4, 128.9, 128.8, 128.7, 127.9, 52.1, 47.9, 19.4.

**HRMS** (ESI): m/z $[M+H]^+$ calcd for $C_{17}H_{17}O_3$: 269.1172; found: 269.1196

---

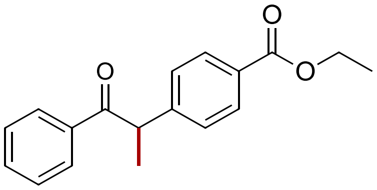

**Ethyl 4-(1-methyl-2-oxo-2-phenylethyl)benzoate[17] (3w) [CAS: 255836-41-0]**

Prepared, following the general procedure C from compound **1w** with a reaction time of 2 h. The crude product was purified *via* flash column chromatography (15 g silica, LP:EtOAc 20:1, 10:1 to yield 37 mg (37 %) of the title compound as a colorless oil.

**$R_f$ =** 0.32 (LP:EtOAc 1:3)

**$^1$H-NMR** (400 MHz, $CDCl_3$): δ = 8.01 – 7.95 (m, 2H), 7.95 – 7.89 (m, 2H), 7.52 – 7.44 (m, 1H), 7.43 – 7.32 (m, 4H), 4.74 (q, J = 6.9 Hz, 1H), 4.34 (q, J = 7.1 Hz, 2H), 1.55 (d, J = 6.9 Hz, 3H), 1.35 (t, J = 7.1 Hz, 3H).

**$^{13}$C{$^1$H}-NMR** (100 MHz, $CDCl_3$): δ = 199.7, 166.4, 146.6, 136.3, 133.1, 130.3, 129.3, 128.8, 128.7, 127.9, 61.0, 48.0, 27.9, 19.4, 14.4.

**HRMS** (ESI): m/z $[M+H]^+$ calcd for $C_{18}H_{19}O_3$: 283.1329; found: 283.1338

---

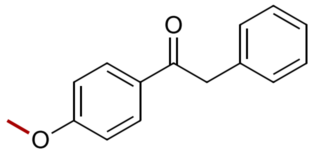

**1-(4-Methoxyphenyl)-2-phenylethanone[22] (3x) [CAS: 1023-17-2]**

Prepared, following the general procedure C, with the deviation of using only 1 equiv of $PhMe_3NI$, from commercially available starting material with a reaction time of 2 h. The crude product was purified *via* column chromatography (LP:EtOAc 70:1-50:1) to yield 68 mg (64 %) of the title compound as a colorless oil.

**$R_f$ = 0.18** (LP:EtOAc 5:1)

**$^1$H-NMR** (400 MHz, $CDCl_3$): δ = 8.02 – 7.94 (m, 2H), 7.34 – 7.17 (m, 5H), 6.95 – 6.86 (m, 2H), 4.21 (s, 2H), 3.82 (s, 3H).

**$^{13}$C{$^1$H}-NMR** (100 MHz, $CDCl_3$): δ = 196.3, 163.6, 135.1, 131.0, 129.7, 129.5, 128.7, 126.9, 113.9, 55.5, 45.3.

---

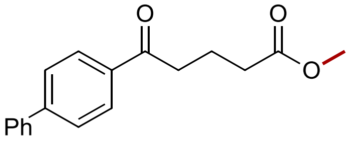

**Methyl 4-(biphenyl-4-yl)-4-oxobutanoate[23] (3y) [CAS: 54011-27-7]**

Prepared, following the general procedure C, with the deviation of using 3 equiv of KOH, from commercially available fenbufen with a reaction time of 24 h. The crude product was purified *via* column chromatography (LP:EtOAc 30:1-1:1) to yield 72 mg (68 %) of the title compound as yellow crystals.

**$R_f$ = 0.49** (LP:EtOAc 1:1)

**$^1$H-NMR** (400 MHz, $CDCl_3$): δ = 8.10 – 8.02 (m, 2H), 7.73 – 7.66 (m, 2H), 7.66 – 7.59 (m, 2H), 7.52 – 7.43 (m, 2H), 7.43 – 7.36 (m, 1H), 3.72 (s, 3H), 3.35 (t, *J* = 6.7 Hz, 2H), 2.80 (t, *J* = 6.6 Hz, 2H).

**$^{13}$C{$^1$H}-NMR** (100 MHz, $CDCl_3$): δ = 197.7, 173.5, 146.0, 139.9, 135.3, 129.1, 128.7, 128.4, 127.4, 51.9, 33.5, 28.2.

**HRMS** (ESI): m/z $[M+H]^+$ calcd for $C_{18}H_{19}O_3$: 283.1329; found: 283.1333

---

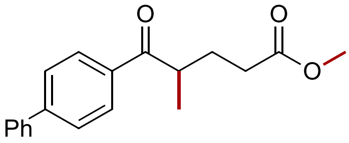

**Methyl 3-methyl-4-oxo-4-(4-phenylphenyl)butanoate (3z) [CAS: 1305108-60-4]**

An 8 mL glass vial equipped with a magnetic stirring bar was charged with fenbufen (100 mg, 1 equiv.), $PhMe_3NI$ (2 equiv.) and KOH (3 equiv.). The vial was sealed with a septum screw cap. Using a cannula, the vial was evacuated and backfilled with argon three times. Anisole (2 mL, 0.2 M) was added *via* syringe. Evacuation and backfilling with argon were repeated three times under vigorous stirring that no boiling delay occurred. Subsequently, the septum screw cap was exchanged for a closed Wheaton® cap, and the vial was sealed tightly. The resulting inhomogeneous mixture was heated to 130 °C in a metallic heating block for 3 h. The reaction mixture was cooled to room temperature, and additional $PhMe_3NI$ (2 equiv) and KOH (2 equiv) were added. Subsequently, the reaction was heated up to 130 °C and

stirred for 4 days (with further addition of 2 equiv $PhMe_3NI$ and 2 equiv KOH after 48 h). The reaction was cooled to room temperature. 2 mL of 2 N HCl were added, and the mixture was extracted 3 times with EtOAc (20 mL each). The combined organic phases were washed twice with 2 N HCl (3 mL each) and once with brine, dried over anhydrous $Na_2SO_4$, filtered, and concentrated. The obtained crude product was purified *via* hand column with unmodified silica gel (15 g silica, LP:EtOAc 30:1-1:1), yielding 34 mg (31 %) of the title compound as a colorless oil.

**$R_f$ = 0.55** (LP:EtOAc 1:1)

**$^1$H-NMR** (400 MHz, $CDCl_3$): δ = 8.11 – 8.04 (m, 2H), 7.74 – 7.67 (m, 2H), 7.67 – 7.59 (m, 2H), 7.52 – 7.43 (m, 2H), 7.43 – 7.36 (m, 1H), 3.99 (dqd, *J* = 8.5, 7.2, 5.7 Hz, 1H), 3.66 (s, 3H), 3.00 (dd, *J* = 16.8, 8.4 Hz, 1H), 2.49 (dd, *J* = 16.8, 5.7 Hz, 1H), 1.27 (d, *J* = 7.2 Hz, 3H)

**$^{13}$C{$^1$H}-NMR** (100 MHz, $CDCl_3$): δ = 202.3, 172.9, 145.8, 140.0, 134.6, 129.1, 129.0, 128.3, 127.4, 127.3, 51.8, 37.3, 37.3, 18.0.

**HRMS** (ESI): m/z $[M+H]^+$ calcd for $C_{19}H_{21}O_3$: 297.1485; found: 297.1490

---

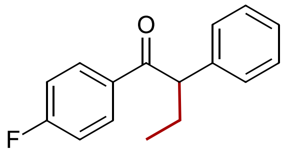


**1-(4-Fluorophenyl)-2-phenyl-1-butanone[19] (4a) [CAS: 1097034-09-7]**

Prepared, following the general procedure C, except for the use of $PhEt_3NI$ (2 equiv.) instead of $PhMe_3NI$, from commercially available starting material with a reaction time of 5 h. The crude product was purified *via* column chromatography (8 g silica LP:EtOAc 50:1-40:1) to yield 83 mg (78 %) of the title compound.

**$R_f$** = 0.36 (LP:EtOAc 7:1)

**$^1$H-NMR** (400 MHz, $CDCl_3$): δ = 7.96 (dd, *J* = 8.9, 5.4 Hz, 2H), 7.52 – 7.11 (m, 5H), 7.03 (dd, *J* = 9.0, 8.3 Hz, 1H), 4.36 (t, *J* = 7.2 Hz, 2H), 2.17 (dp, *J* = 12.8, 7.3 Hz, 1H), 1.83 (dp, *J* = 13.6, 7.4 Hz, 1H), 0.88 (t, *J* = 7.4 Hz, 3H).

**$^{13}$C{$^1$H}-NMR** (101 MHz, $CDCl_3$): δ = 198.6, 165.6 (d, *J* = 254.6 Hz), 139.6, 133.5, 131.4 (d, *J* = 9.2 Hz), 129.0, 128.3, 127.2, 115.7 (d, *J* = 21.8 Hz), 55.6, 27.2, 12.4

**$^{19}$F-NMR** (376 MHz, $CDCl_3$): δ = -105.7

**HRMS** (ESI): *m/z* $[M+H]^+$ calcd for $C_{16}H_{16}FO$: 243.1180; found: 243.1186

---

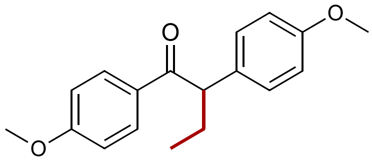


**1,2-Bis(4-methoxyphenyl)-1-butanone[24] (4b) [CAS:4390-94-7]**

Prepared, following the general procedure C, except for the use of $PhEt_3NI$ (2 equiv.) instead of $PhMe_3NI$, from commercially available starting material with a reaction time of 4 h. The crude product was purified *via* column chromatography (15 g silica LP:EtOAc 30:1-20:1) to yield 74 mg (68 %) of the title compound.

**$R_f$** = 0.57 (LP:EtOAc 1:1)

**$^1$H-NMR** (400 MHz, $CDCl_3$): δ = 8.00 – 7.91 (m, 2H), 7.25 – 7.17 (m, 2H), 6.90 – 6.78 (m, 4H), 4.35 (t, J = 7.3 Hz, 1H), 3.81 (s, 3H), 3.75 (s, 3H), 2.23 – 2.08 (m, 1H), 1.82 (dq, J = 13.6, 7.4 Hz, 1H), 0.89 (t, J = 7.4 Hz, 3H).

**$^{13}$C{$^1$H}-NMR** (101 MHz, $CDCl_3$): δ = 198.9, 163.2, 158.6, 132.2, 131.0, 130.1, 129.3, 114.3, 113.7, 55.5, 55.31, 54.2, 27.2, 12.4.

**HRMS** (ESI): *m/z* $[M+H]^+$ calcd for $C_{18}H_{21}O_3$: 285.1485; found: 285.1492

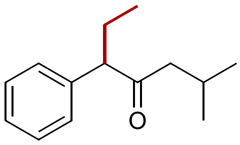

**2-Methyl-5-phenyl-4-heptanone (4c) [CAS: 1129271-28-8]**

Prepared, following the general procedure C, except for the use of $PhEt_3NI$ (2 equiv.) instead of $PhMe_3NI$, from commercially available starting material with a reaction time of 18 h. The crude product was purified *via* column chromatography (15 g silica LP:$Et_2O$ 100:1-100:3) to yield 65 mg (57 %) of the title compound as slightly yellow oil.

**$R_f$ =** 0.35 (LP:EtOAc 10:1)

**$^1$H-NMR** (400 MHz, $CDCl_3$): δ = 7.38 – 7.30 (m, 2H), 7.29 – 7.24 (m, 1H), 7.24 – 7.18 (m, 2H), 3.51 (t, J = 7.4 Hz, 1H), 2.33 – 2.17 (m, 2H), 2.17 – 2.00 (m, 2H), 1.72 (dp, J = 13.7, 7.5 Hz, 1H), 0.89 – 0.80 (m, 6H), 0.76 (d, J = 6.6 Hz, 3H).

**$^{13}$C{$^1$H}-NMR** (101 MHz, $CDCl_3$): δ = 210.2, 139.0, 128.8, 128.4, 127.1, 61.3, 51.0, 25.3, 24.3, 22.7, 22.3, 12.2.

**HRMS** (ESI): *m/z* $[M+H]^+$ calcd for $C_{14}H_{21}O$: 205.1587; found: 205.1593

---

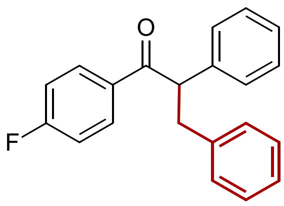

**1-(4-Fluorophenyl)-2,3-diphenylpropan-1-one[25] (5a) [CAS: 2149042-05-5]**

An 8 mL glass vial equipped with a magnetic stirring bar was charged with benzyl 4-fluorophenyl ketone **1a** (100 mg, 1 equiv.), benzyl trimethyl ammonium chloride (1.1 equiv.), and KOH (2 equiv.). The vial was sealed with a septum screw cap. Using a cannula, the vial was evacuated and backfilled with argon three times. Anisole (2 mL, 0.2 M) was added *via* syringe. Evacuation and backfilling with argon were repeated three times under vigorous stirring that no boiling delay occurred. Subsequently, the septum screw cap was exchanged for a closed Wheaton® cap, and the vial was sealed tightly. The resulting inhomogeneous mixture was heated to 130 °C in a metallic heating block for 1 h. After complete consumption of the starting material (TLC analysis), the reaction was cooled to room temperature, filtered over a short plug of silica, and concentrated. The obtained crude product was purified *via* hand column with unmodified silica gel (15 g silica, LP:EtOAc 150:1-100:1), yielding 119 mg (84 %) of the title compound as white crystals.

**$R_f$** = 0.39 (LP:EtOAc 5:1)

**$^1$H-NMR** (400 MHz, $CDCl_3$): δ = 7.99 – 7.89 (m, 2H), 7.32 – 7.13 (m, 8H), 7.13 – 7.06 (m, 2H), 7.05 – 6.96 (m, 2H), 4.78 (t, *J* = 7.2 Hz, 1H), 3.58 (dd, *J* = 13.7, 7.5 Hz, 1H), 3.08 (dd, *J* = 13.7, 7.0 Hz, 1H).

**$^{13}$C{$^1$H}-NMR** (100 MHz, $CDCl_3$): δ = 197.7, 165.6 (d, *J* = 254.9 Hz), 139.7, 139.0, 133.2 (d, *J* = 3.0 Hz), 131.4, 131.3, 129.2, 129.1, 128.3 (d, *J* = 4.6 Hz), 127.3, 126.3, 115.6 (d, *J* = 21.9 Hz), 56.0, 40.2.

**HRMS** (ESI): m/z $[M+H]^+$ calcd for $C_{21}H_{18}FO$: 305.1336; found: 305.1354

---

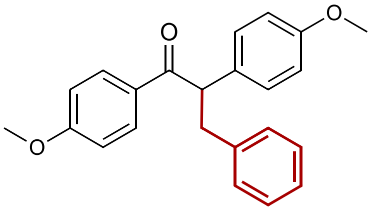

**1,2-Bis-(4-methoxy-phenyl)-3-phenyl-propan-1-one[26] (5b) [CAS: 854692-29-8]**

An 8 mL glass vial equipped with a magnetic stirring bar was charged with desoxyanisoin (100 mg, 1 equiv.), benzyl trimethyl ammonium chloride (1.1 equiv.), and KOH (2 equiv.). The vial was sealed with a septum screw cap. Using a cannula, the vial was evacuated and backfilled with argon three times. Anisole (2 mL, 0.2 M) was added *via* syringe. Evacuation and backfilling with argon were repeated three times under vigorous stirring that no

boiling delay occurred. Subsequently, the septum screw cap was exchanged for a closed Wheaton® cap, and the vial was sealed tightly. The resulting inhomogeneous mixture was heated to 130 °C in a metallic heating block for 2 h. After complete consumption of the starting material (TLC analysis), the reaction was cooled to room temperature, filtered over a short plug of silica, and concentrated. The obtained crude product was purified *via* hand column with unmodified silica gel (15 g silica, LP:EtOAc 100:1, 40:1, 20:1, 10:1), yielding 121 mg (89 %) of the title compound as colorless oil.

**$R_f$ =** 0.29 (LP:EtOAc 3:1)

**$^1$H-NMR** (400 MHz, $CDCl_3$): δ = 7.96 – 7.87 (m, 2H), 7.24 – 7.11 (m, 5H), 7.11 – 7.04 (m, 2H), 6.87 – 6.75 (m, 4H), 4.72 (t, J = 7.2 Hz, 1H), 3.79 (s, 3H), 3.75 (s, 3H), 3.53 (dd, J = 13.7, 7.3 Hz, 1H), 3.04 (dd, J = 13.7, 7.2 Hz, 1H).

**$^{13}$C{$^1$H}-NMR** (100 MHz, $CDCl_3$): δ = 198.0, 163.3, 158.6, 140.1, 131.6, 131.0, 129.8, 129.3, 129.2, 128.2, 126.1, 114.3, 113.7, 55.5, 55.2, 54.7, 40.2.

**HRMS** (ESI): m/z $[M+H]^+$ calcd for $C_{23}H_{23}O_3$: 347.1642; found: 347.1650

---

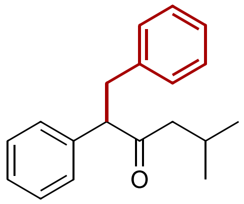


**5-Methyl-1,2-diphenyl-3-hexanone (5c)**

An 8 mL glass vial equipped with a magnetic stirring bar was charged with 1-phenyl-4-methyl-pentanone (100 mg, 1 equiv.), benzyl trimethyl ammonium chloride (1.1 equiv.), and KOH (2 equiv.). The vial was sealed with a septum screw cap. Using a cannula, the vial was evacuated and backfilled with argon three times. Anisole (2.5 mL, 0.2 M) was added *via* syringe. Evacuation and backfilling with argon were repeated three times under vigorous stirring that no boiling delay occurred. Subsequently, the septum screw cap was exchanged for a closed Wheaton® cap, and the vial was sealed tightly. The resulting inhomogeneous mixture was heated to 130 °C in a metallic heating block for 18 h. After complete consumption of the starting material (TLC analysis), the reaction was cooled to room temperature, filtered over a short plug of silica, and concentrated. The obtained crude product was purified *via* hand column with unmodified silica gel (15 g silica, LP:$Et_2O$ 100:1-100:3), yielding 115 mg (78 %) of the title compound as colorless oil.

**$R_f$ =** 0.4 (LP:EtOAc 10:1)

**$^1$H-NMR** (400 MHz, $CDCl_3$): δ = 7.47 – 7.29 (m, 7H), 7.29 – 7.23 (m, 1H), 7.22 – 7.16 (m, 2H), 4.07 – 3.98 (m, 1H), 3.56 (ddd, J = 13.7, 7.9, 1.6 Hz, 1H), 3.03 (ddd, J = 13.7, 6.8, 1.4 Hz, 1H), 2.29 (dt, J = 6.3, 1.3 Hz, 2H), 2.25 – 2.09 (m, 1H), 0.89 (dd, J = 6.6, 1.2 Hz, 3H), 0.79 (dd, J = 6.5, 1.2 Hz, 3H).

**$^{13}$C{$^1$H}-NMR** (100 MHz, $CDCl_3$): δ = 209.3, 139.9, 138.5, 129.1, 128.9, 128.5, 128.3, 128.0, 127.3, 127.1, 126.1, 61.3, 51.4, 38.7, 24.2, 22.6, 22.1.

**HRMS** (ESI): m/z $[M+H]^+$ calcd for $C_{19}H_{23}O$: 267.1743; found: 267.1755

---

# NMR-Spectra

## 1-(3,4-Dimethoxyphenyl)-2-phenylethanone (1g)

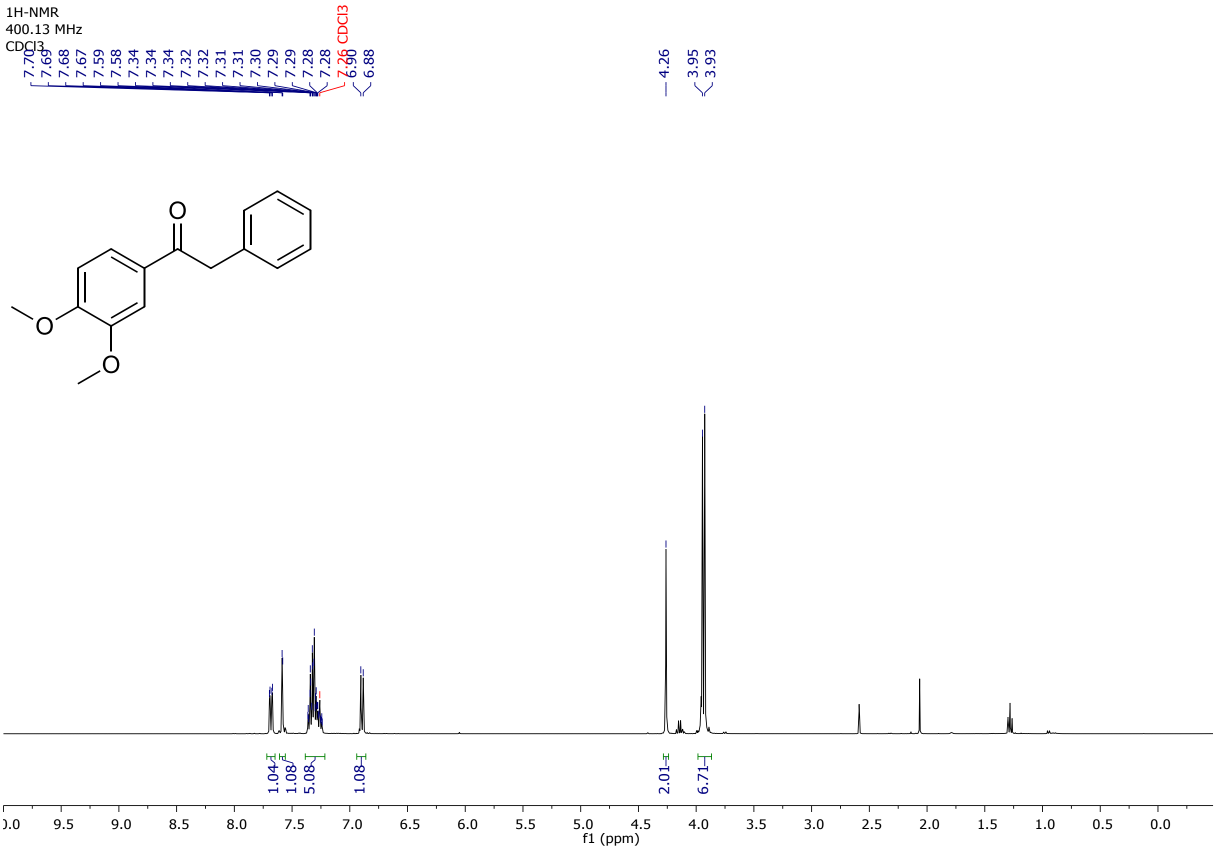


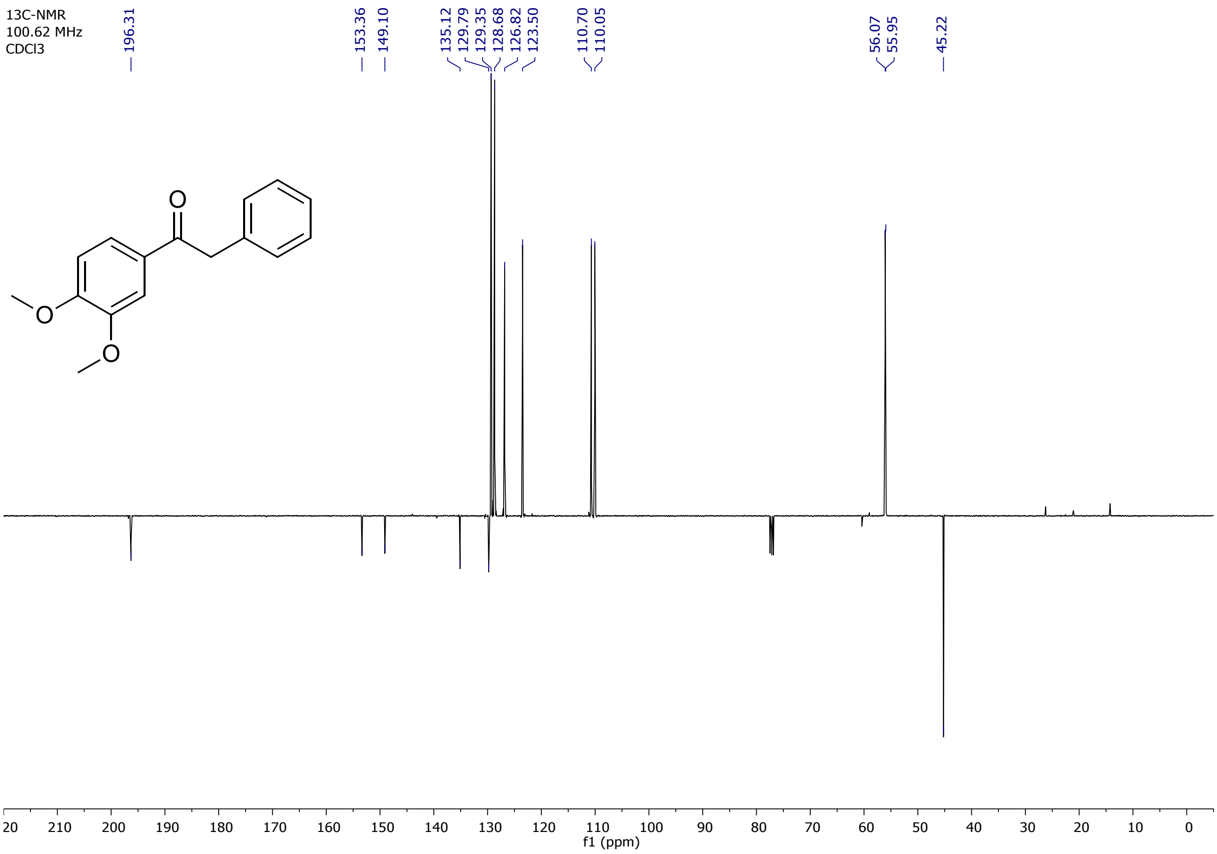

## 1-(3-Methoxyphenyl)-2-phenylethanone (1h)

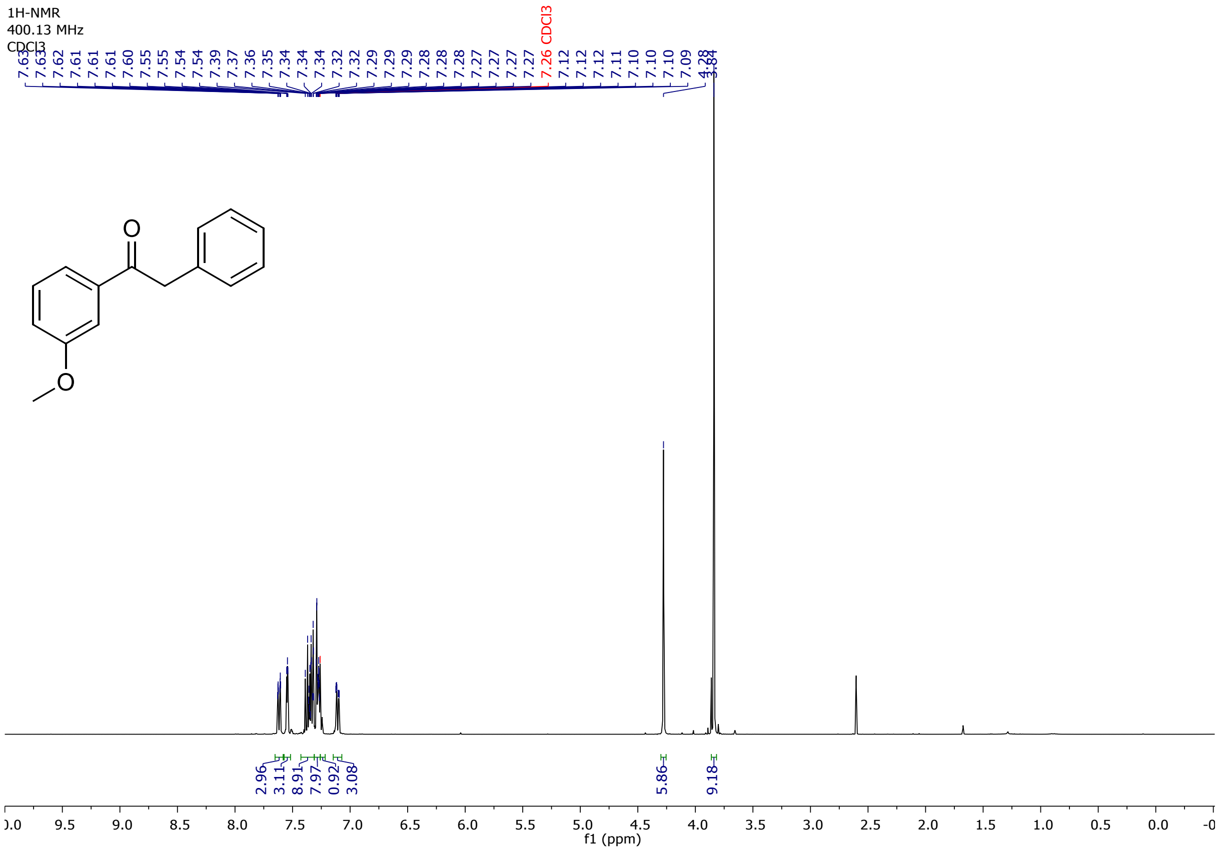


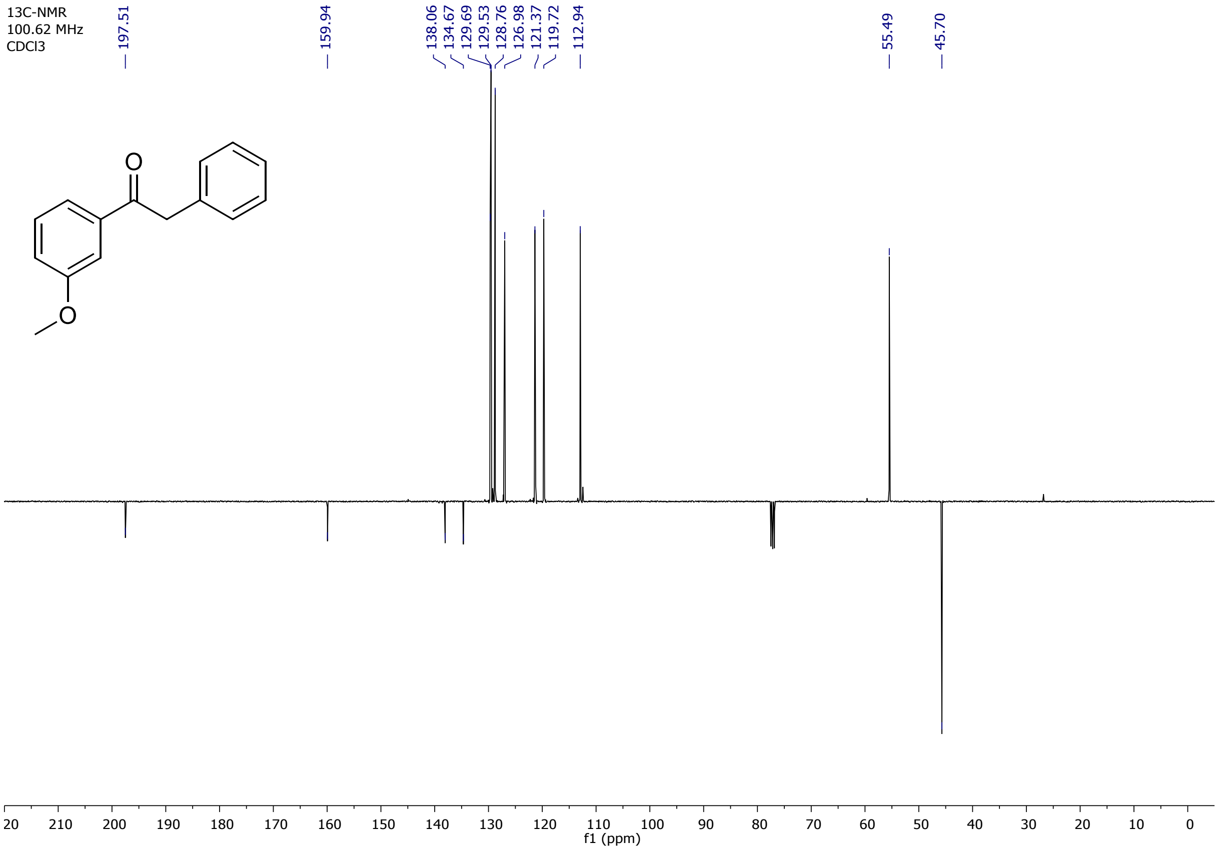

## 2-Phenyl-1-[3-(trifluormethyl)phenyl]ethanone (1i)

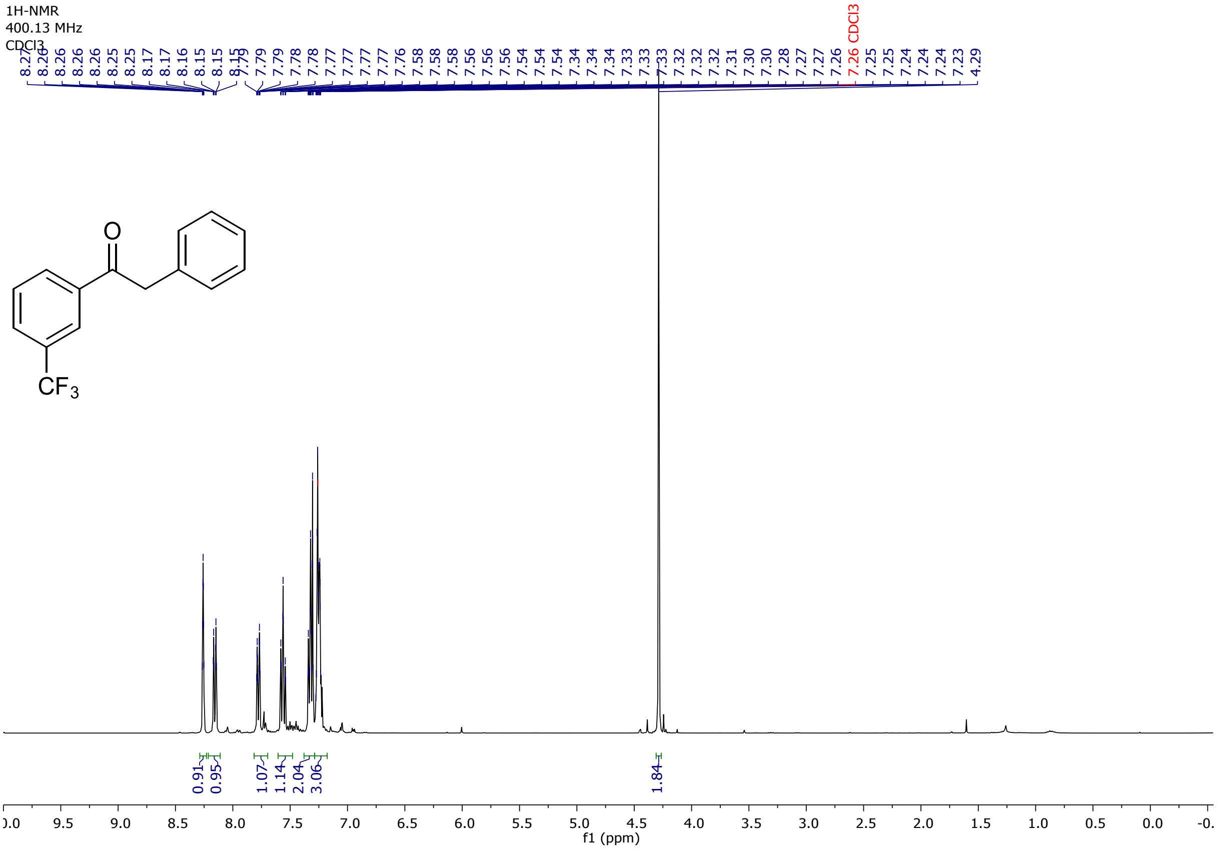


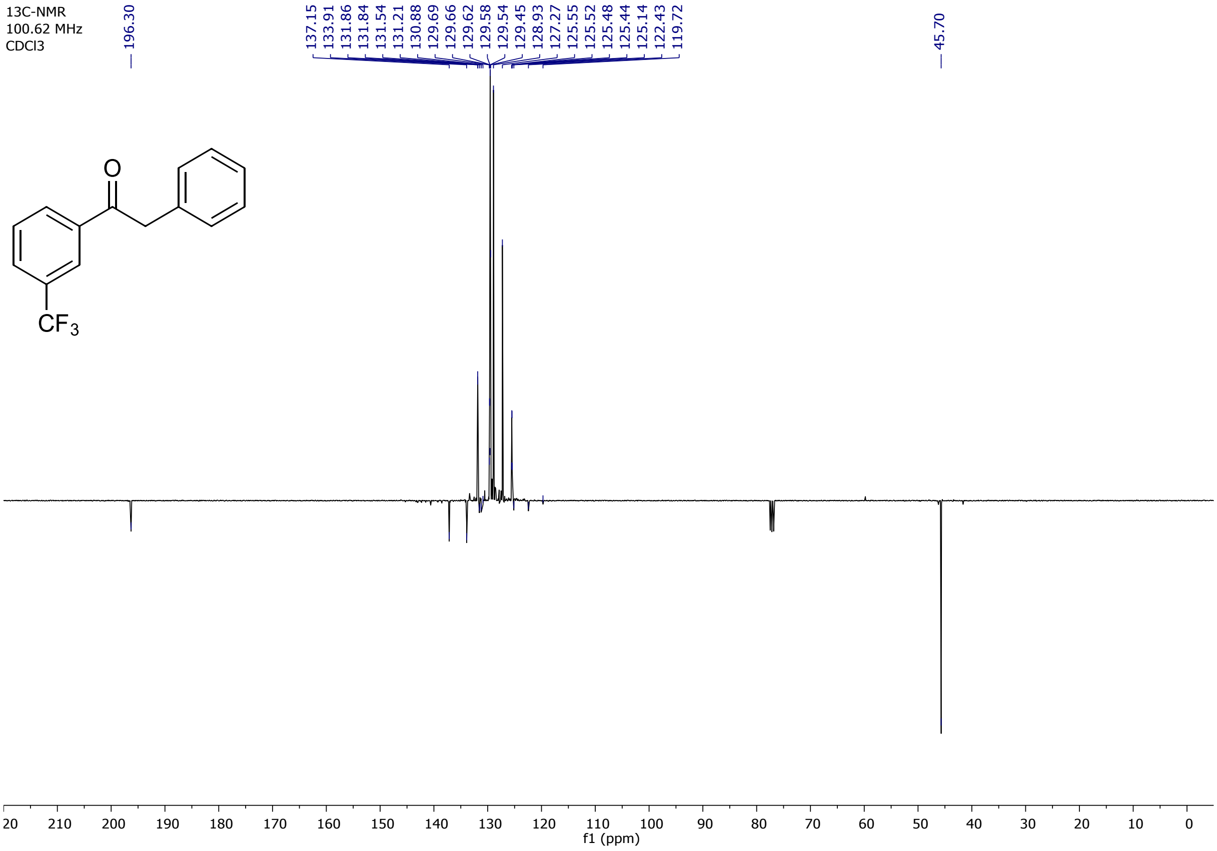

# 1-[4-(2-Methylpropyl)phenyl]-2-phenylethanone (1j)

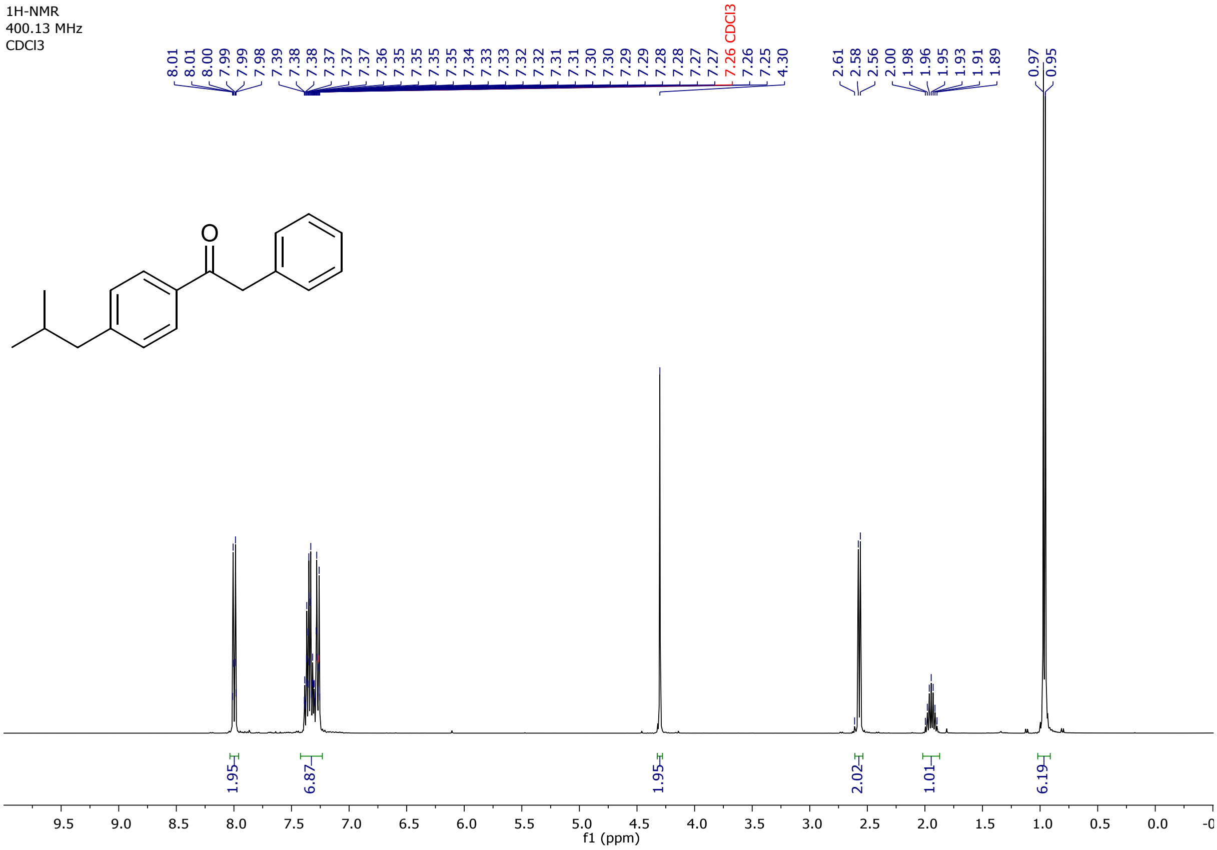


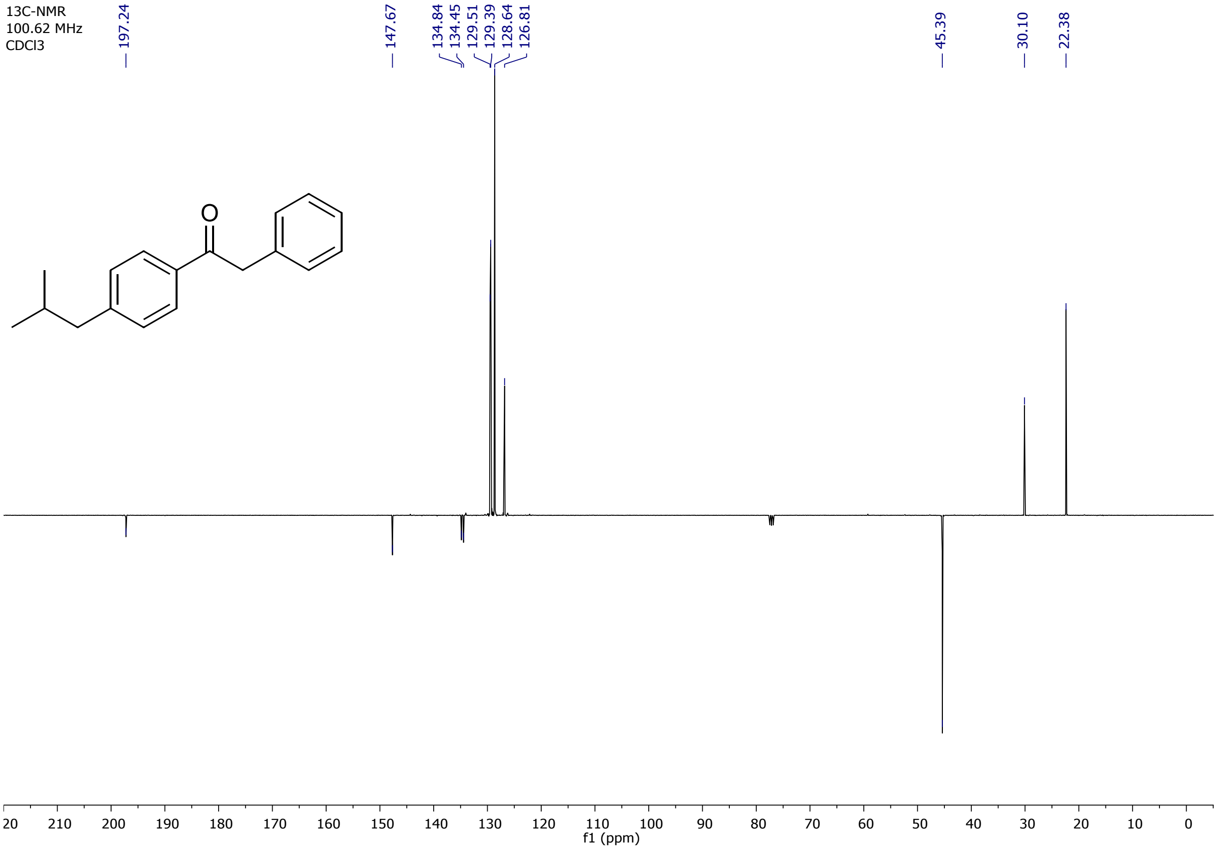

# 1-(4-Methylphenyl)-2-phenylethanone (1k)

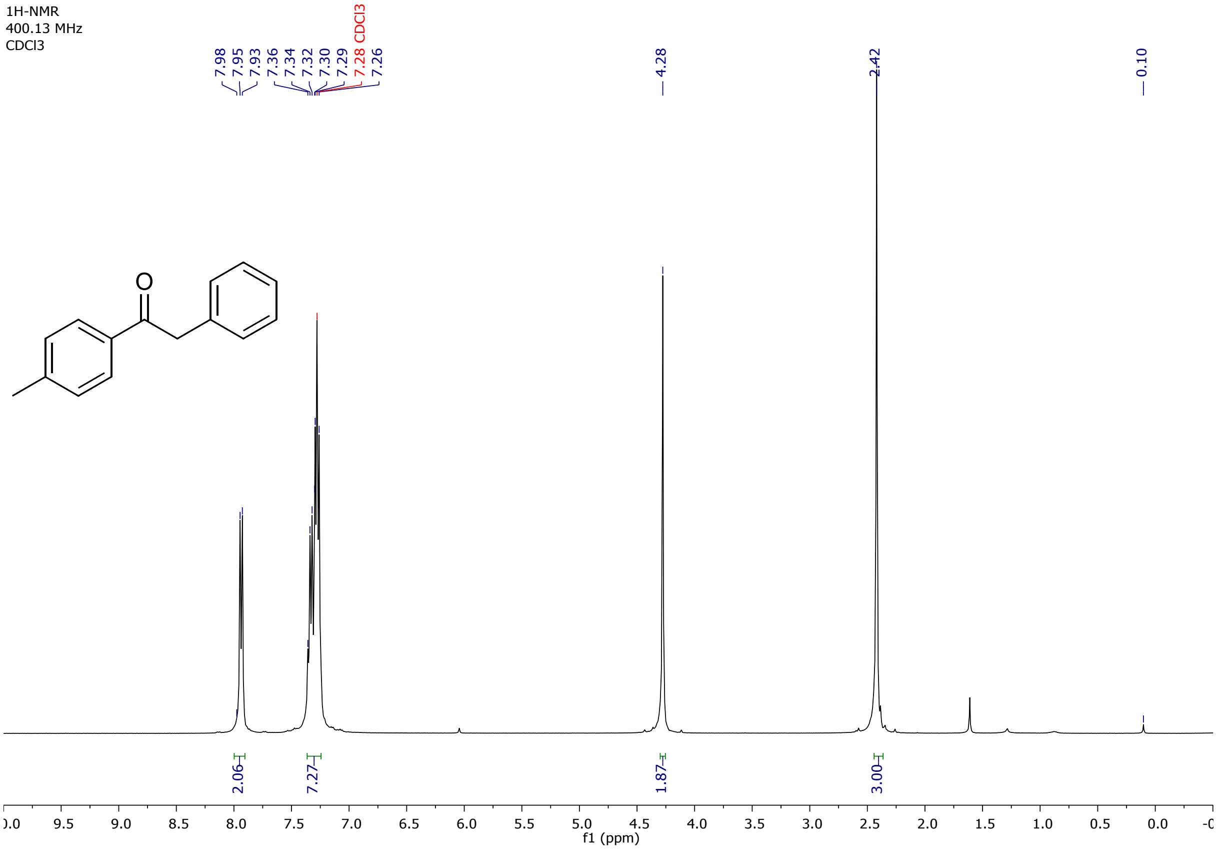


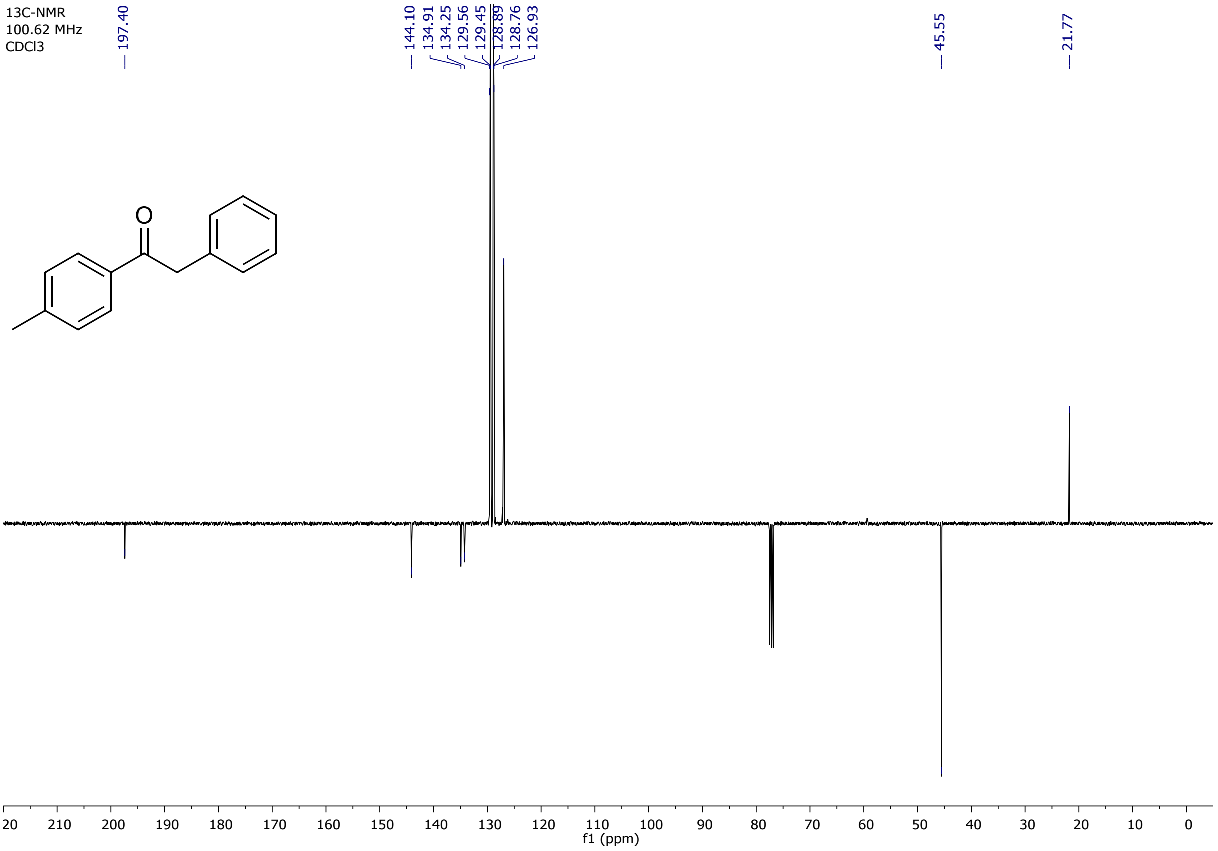

## 1-(2-Naphthalenyl)-2-phenylethanone (1l)

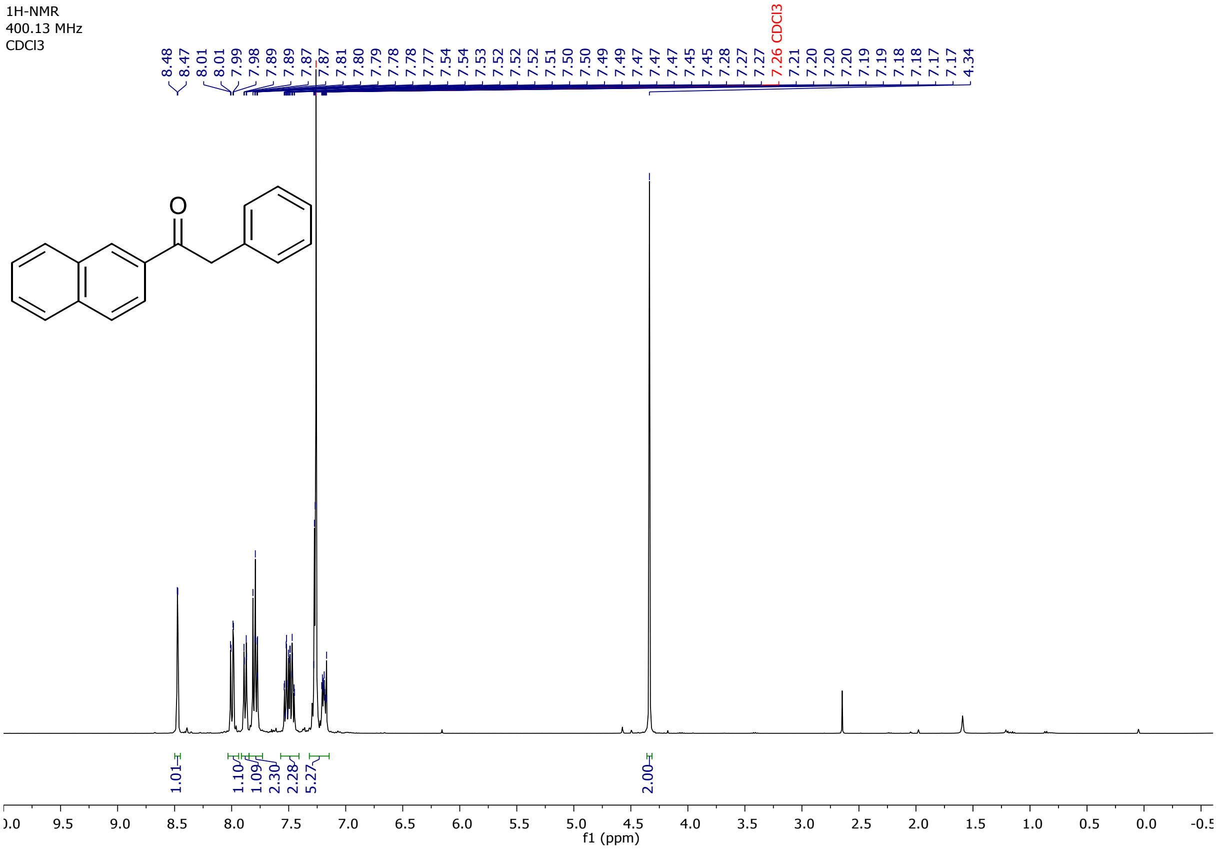


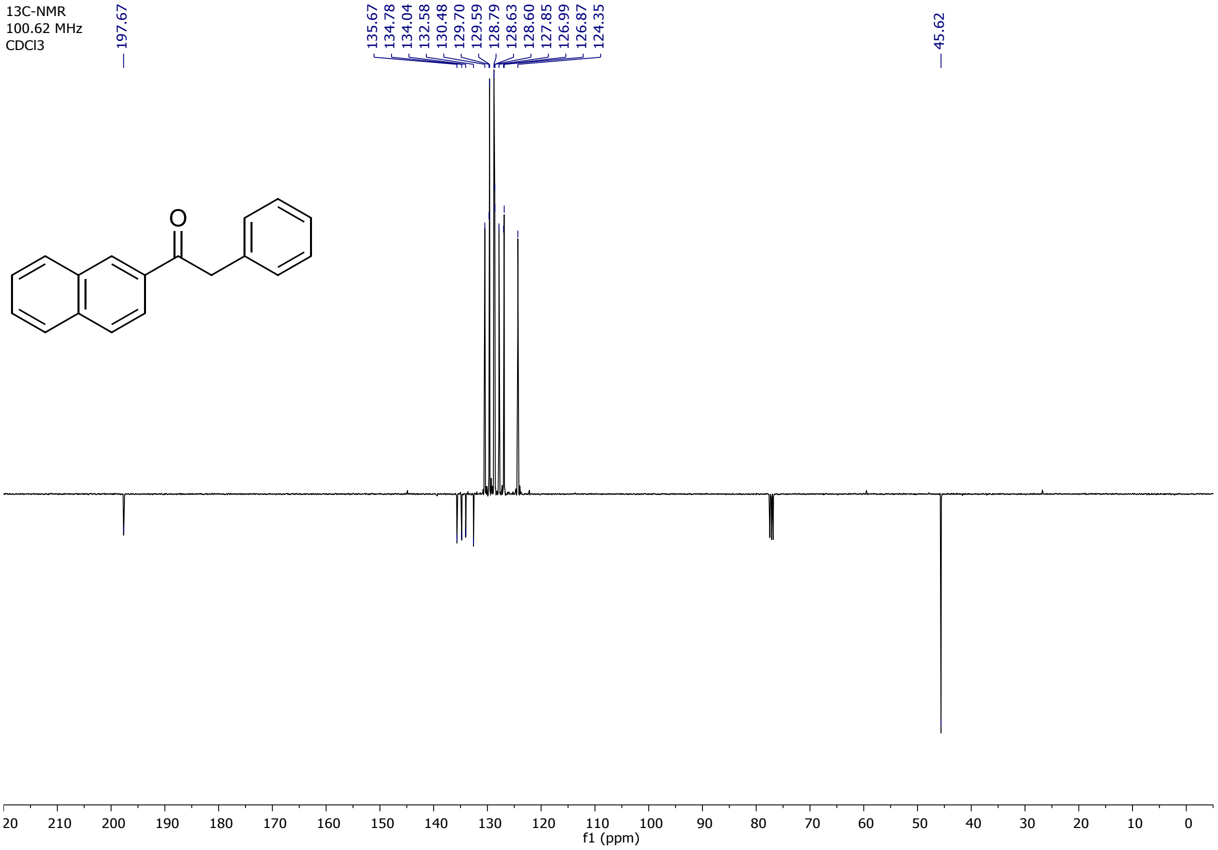

## 1-(1,3-Benzodioxol-5-yl)-2-phenylethanone (1n)

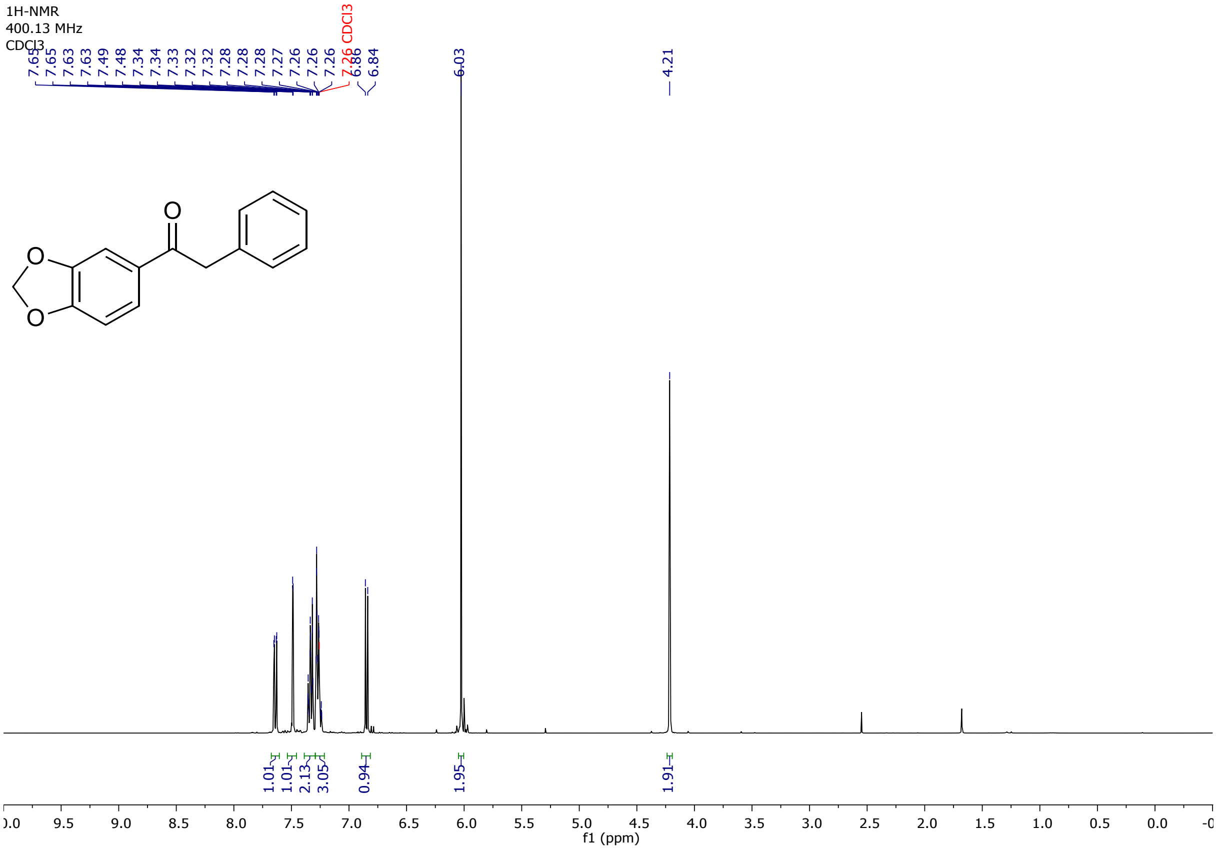


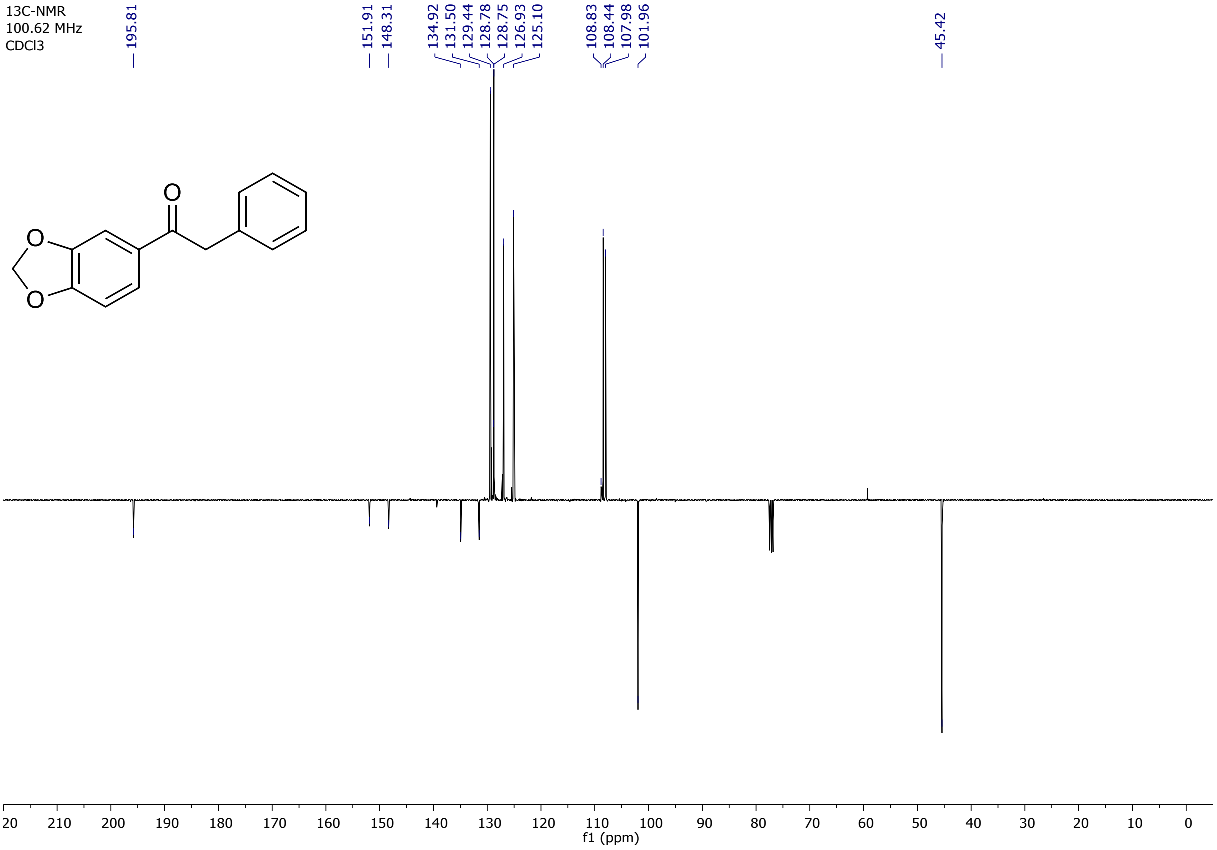

## 2-(3-Methoxyphenyl)-1-phenylethanone (1o)

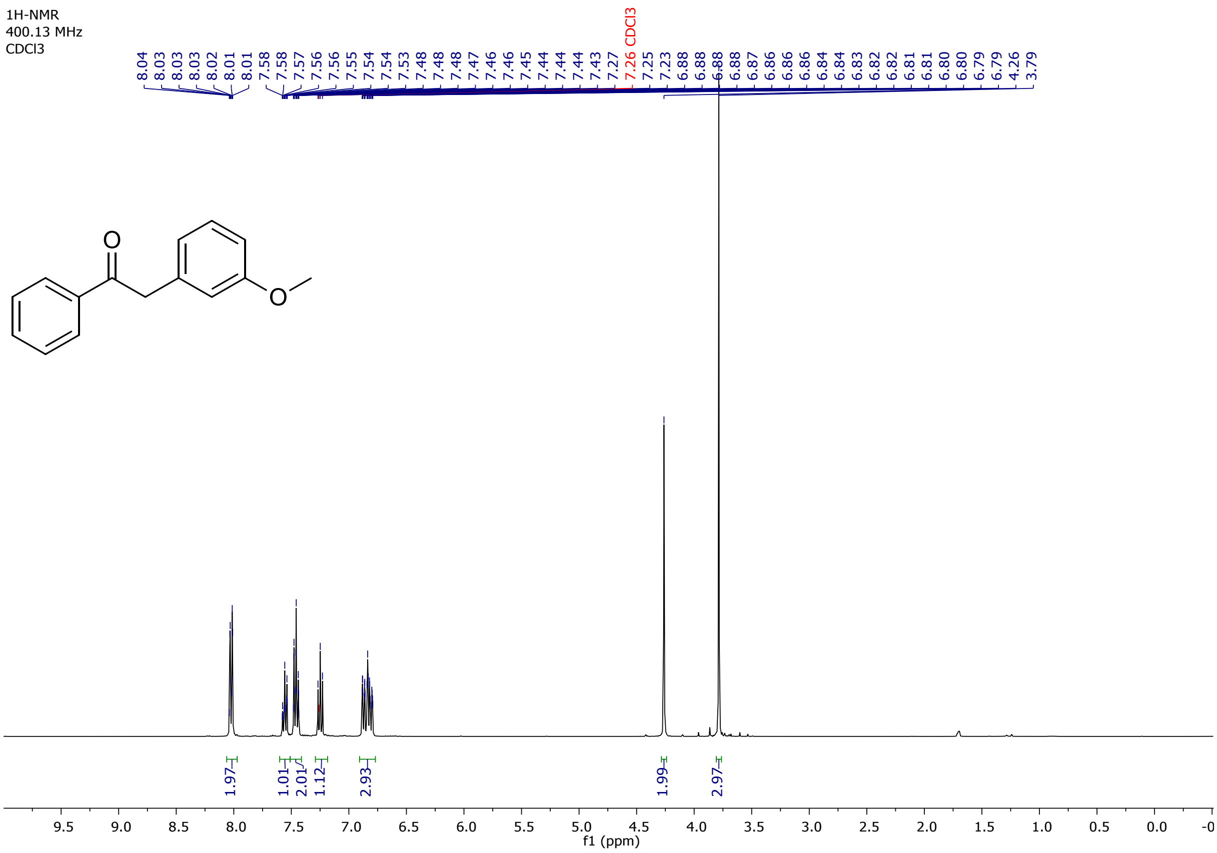


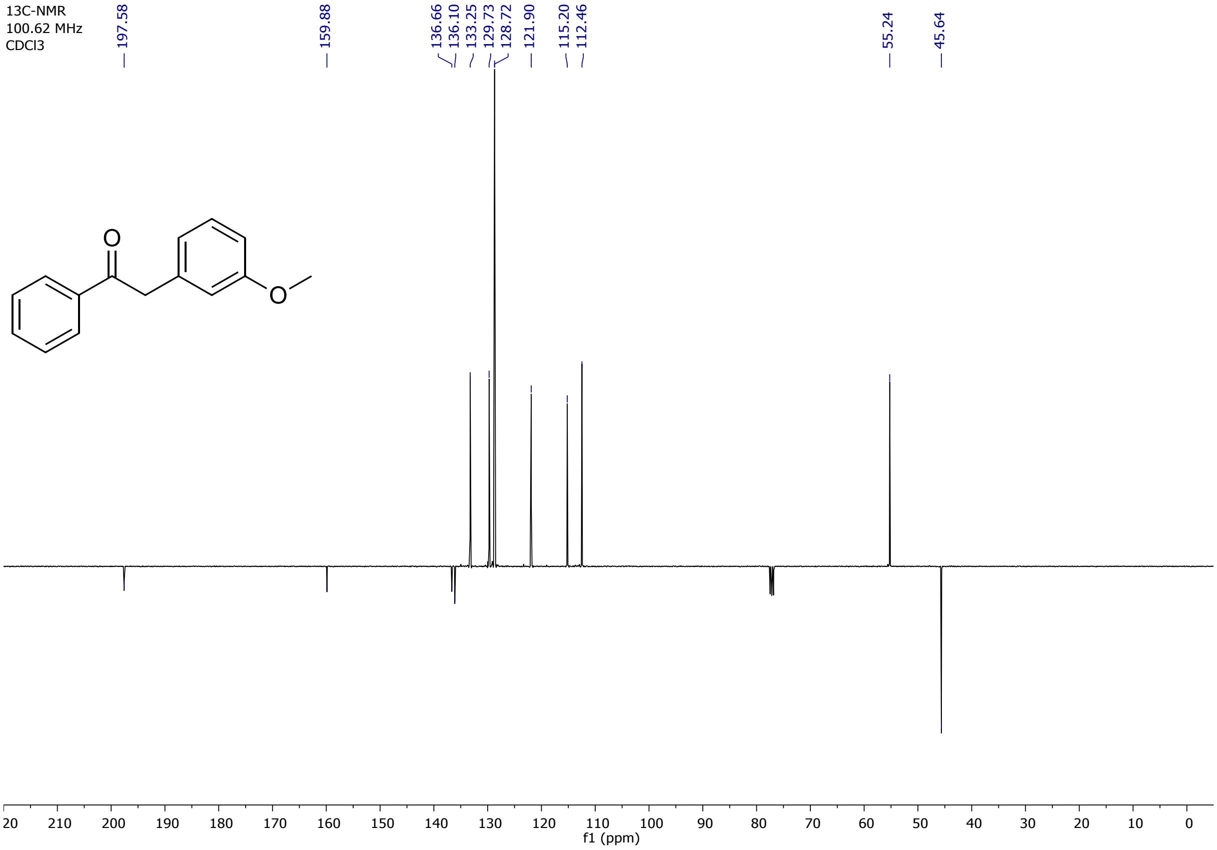

## 2-(4-Fluorphenyl)-1-phenylethanone (1q)

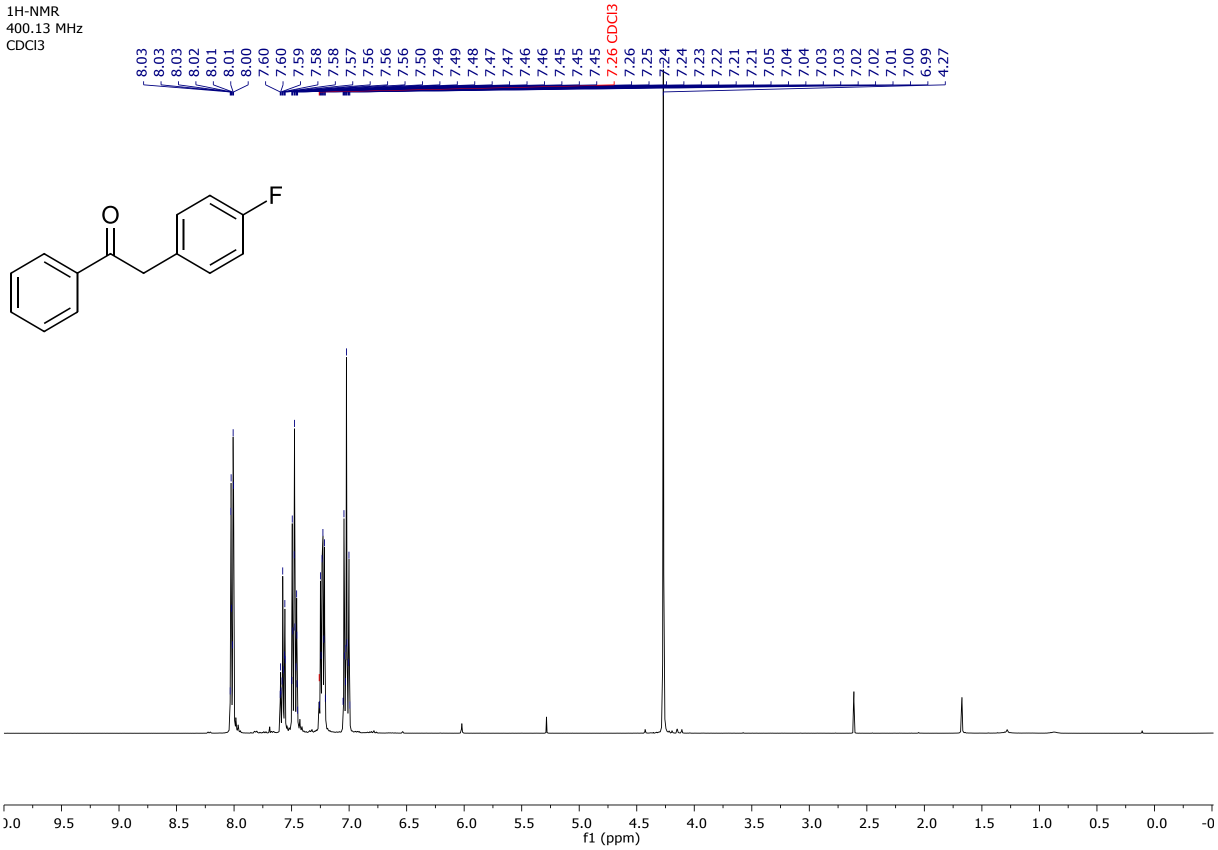


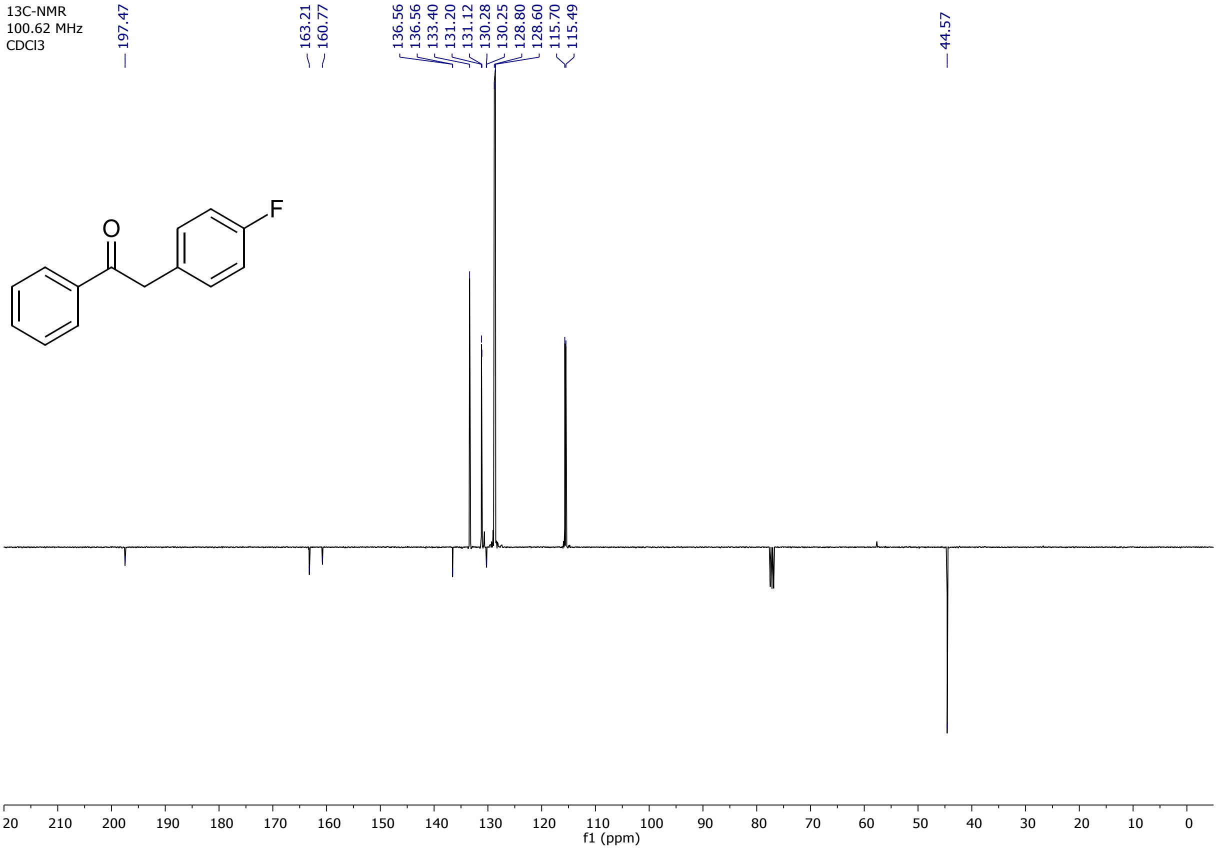

## 1-[1,1'-Biphenyl]-4-yl-2-phenylethanone (1r)

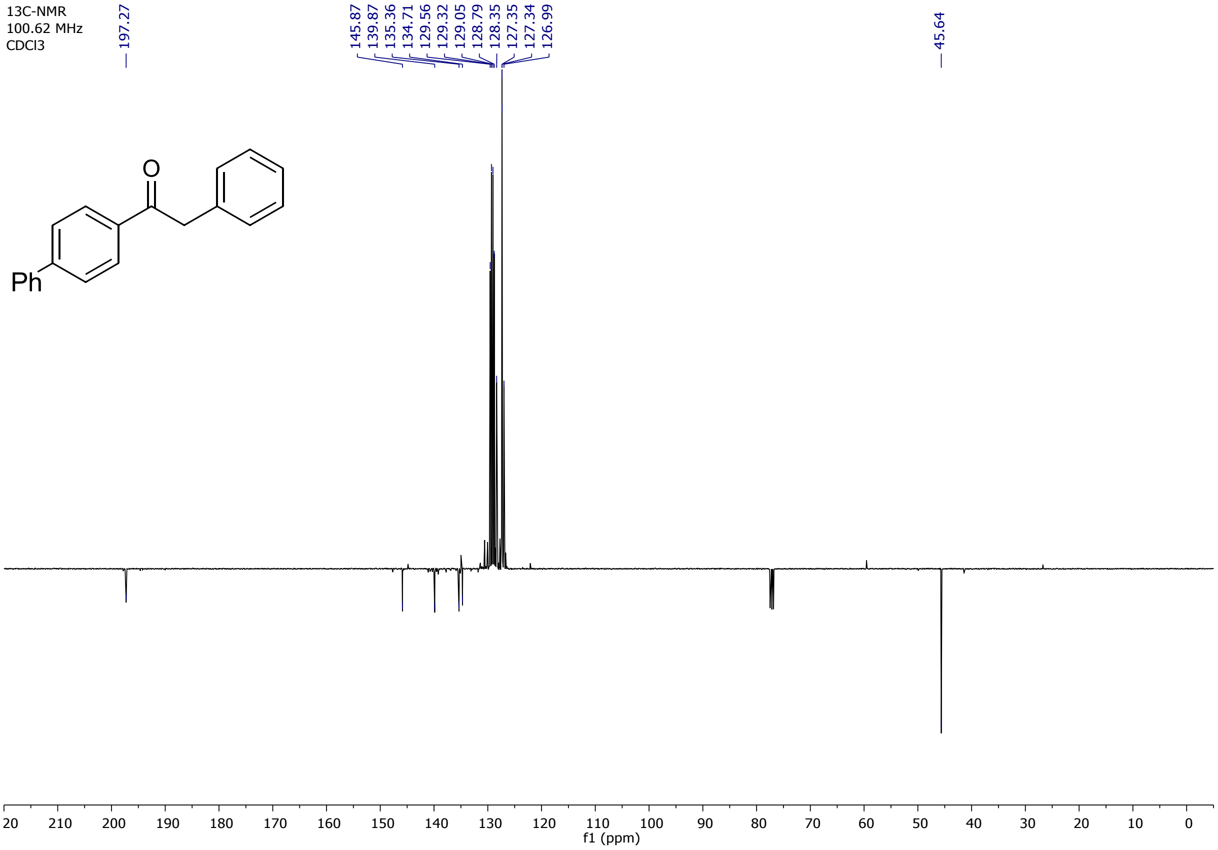


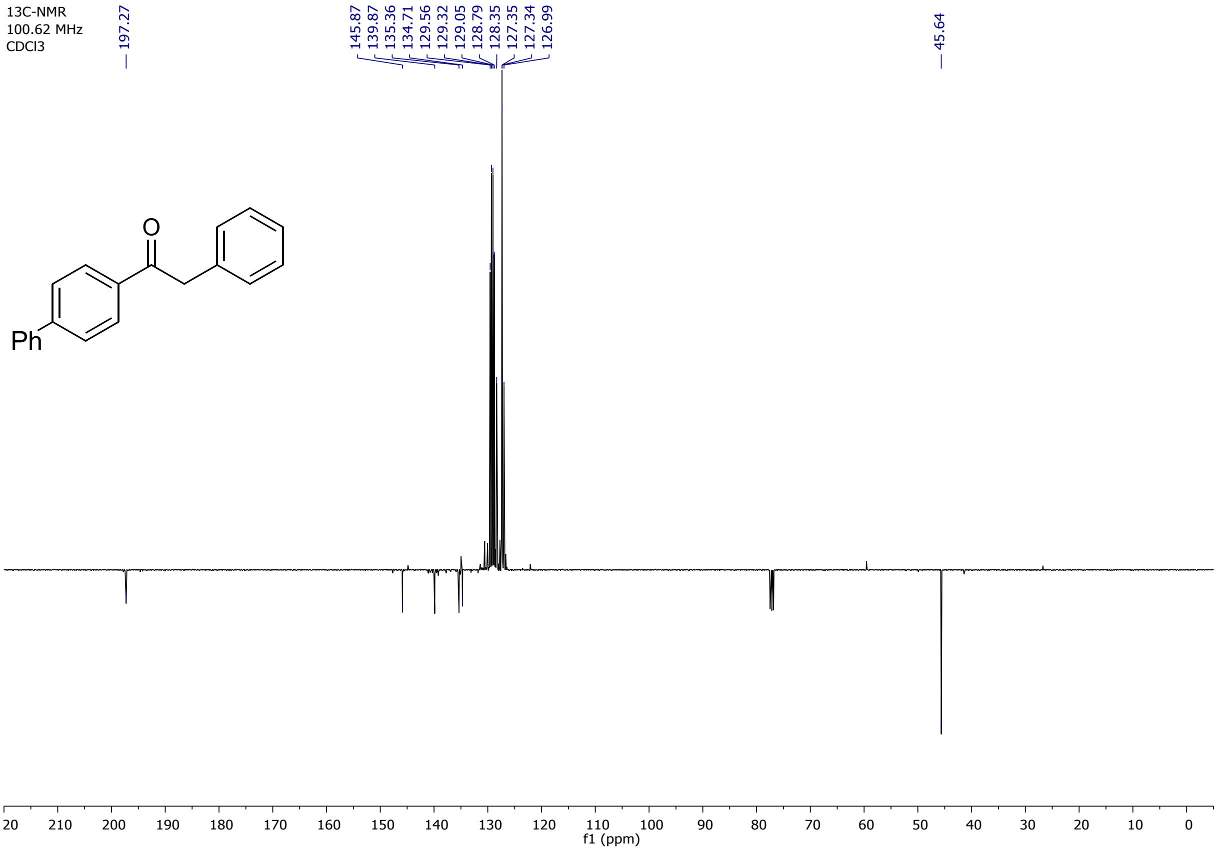

## Methyl 4-(2-oxo-2-phenylethyl)benzoate (1v)

1H-NMR
400.13 MHz
CDCl3

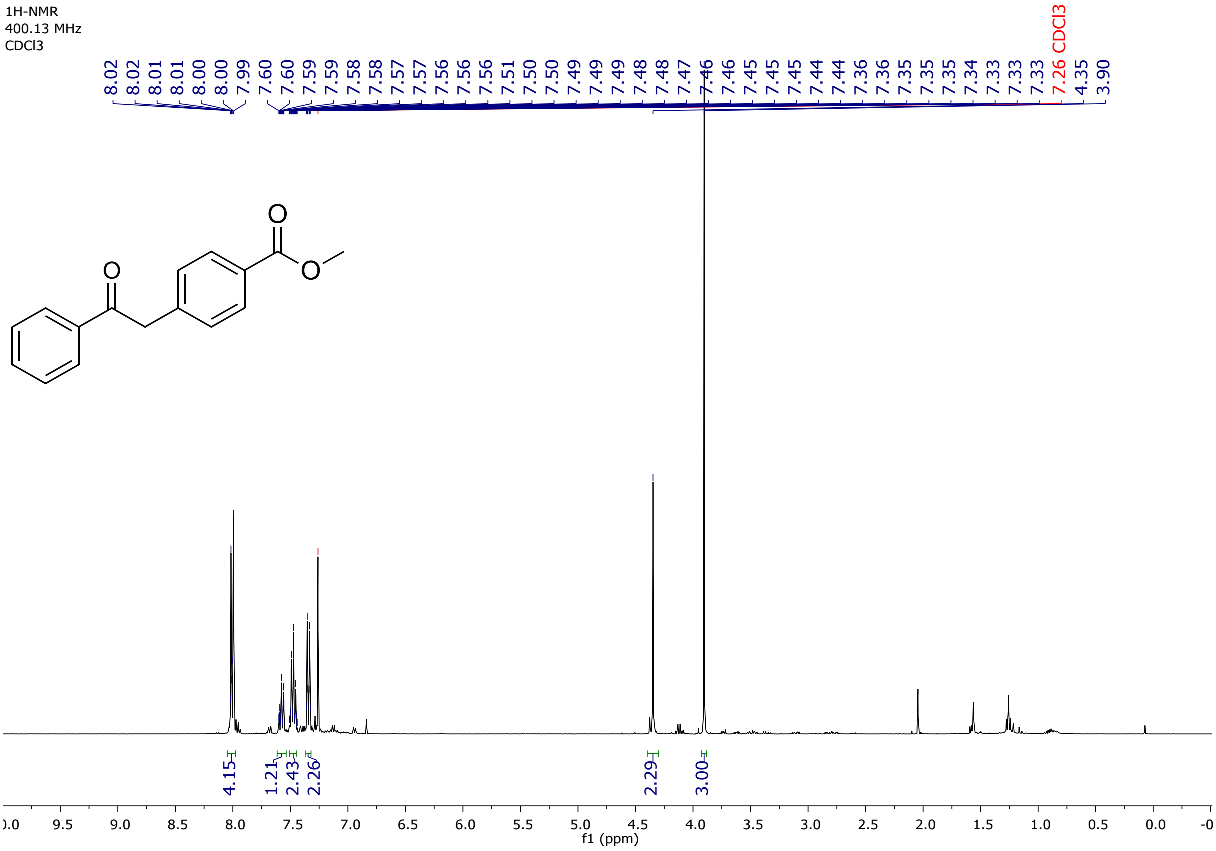


13C-NMR
100.62 MHz
CDCl3

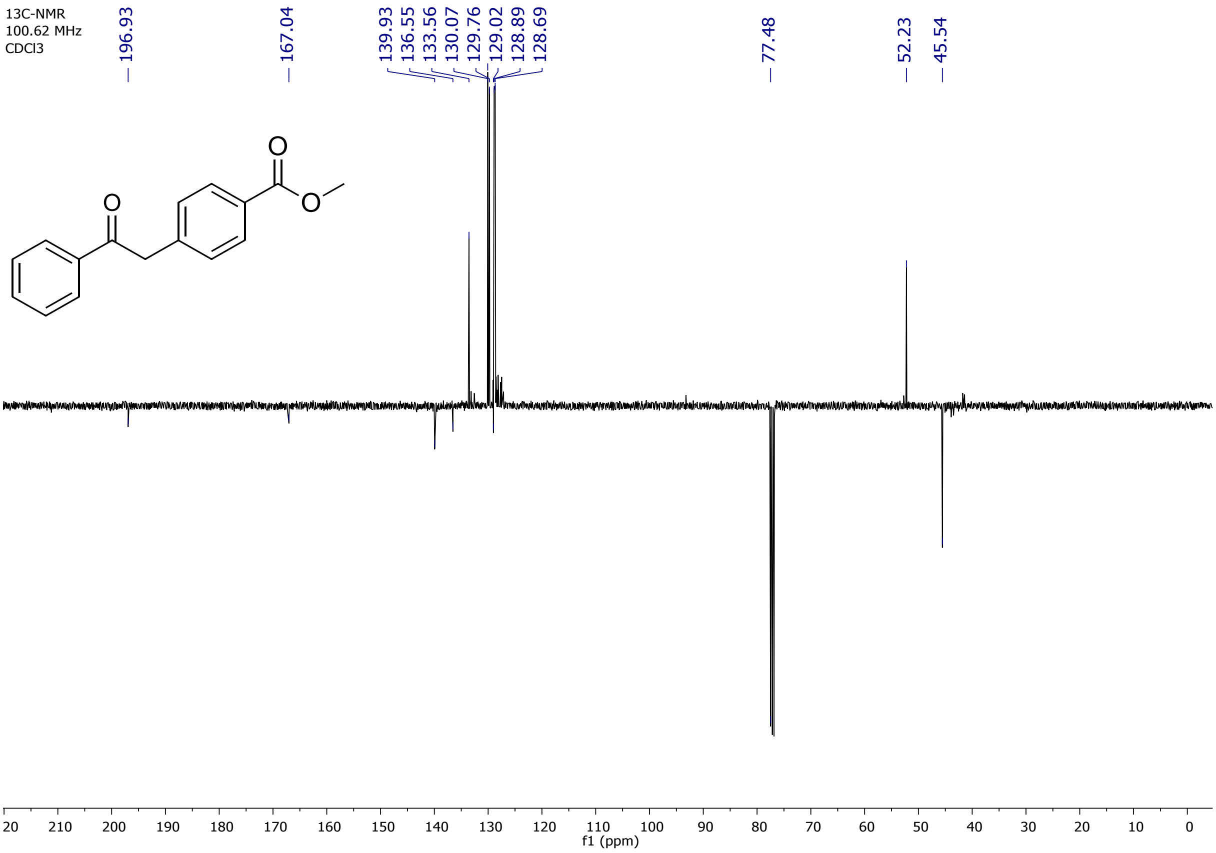

## Ethyl 4-(2-oxo-2-phenylethyl)benzoate (1w)

1H-NMR
400.13 MHz
CDCl3

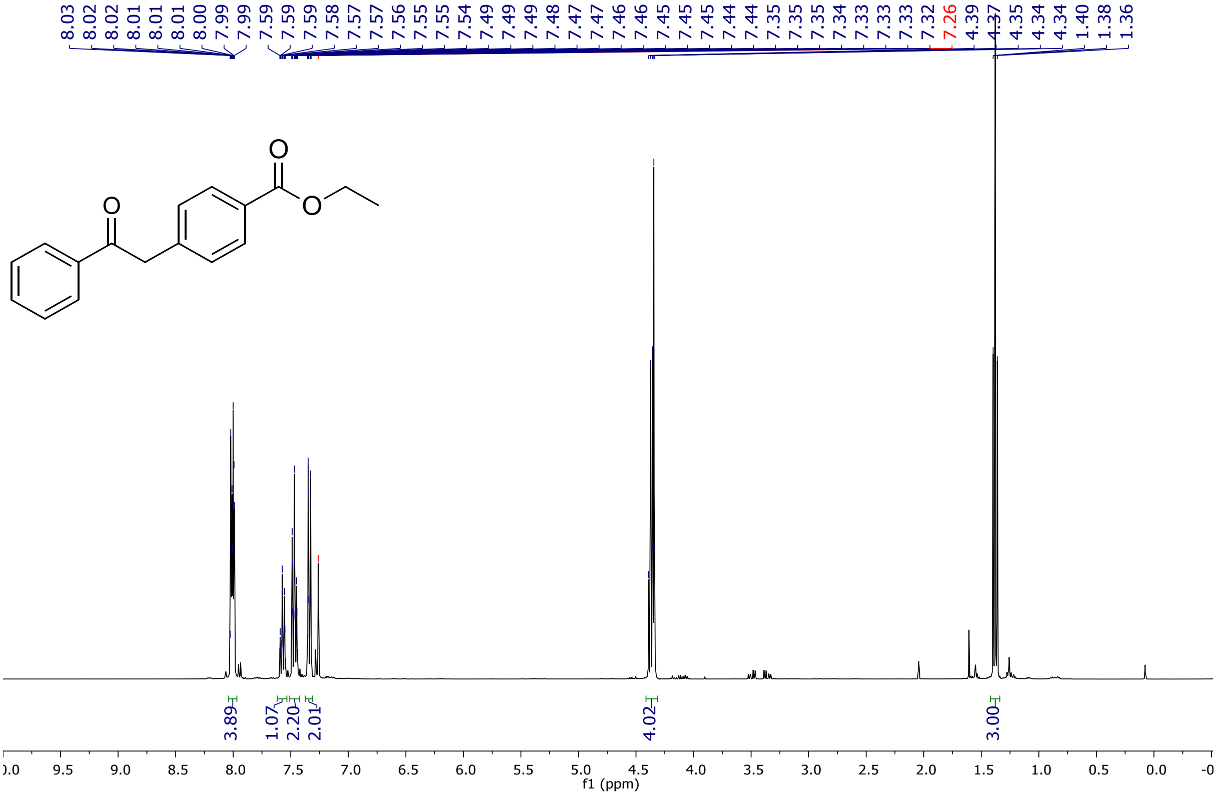


13C-NMR
100.62 MHz
CDCl3

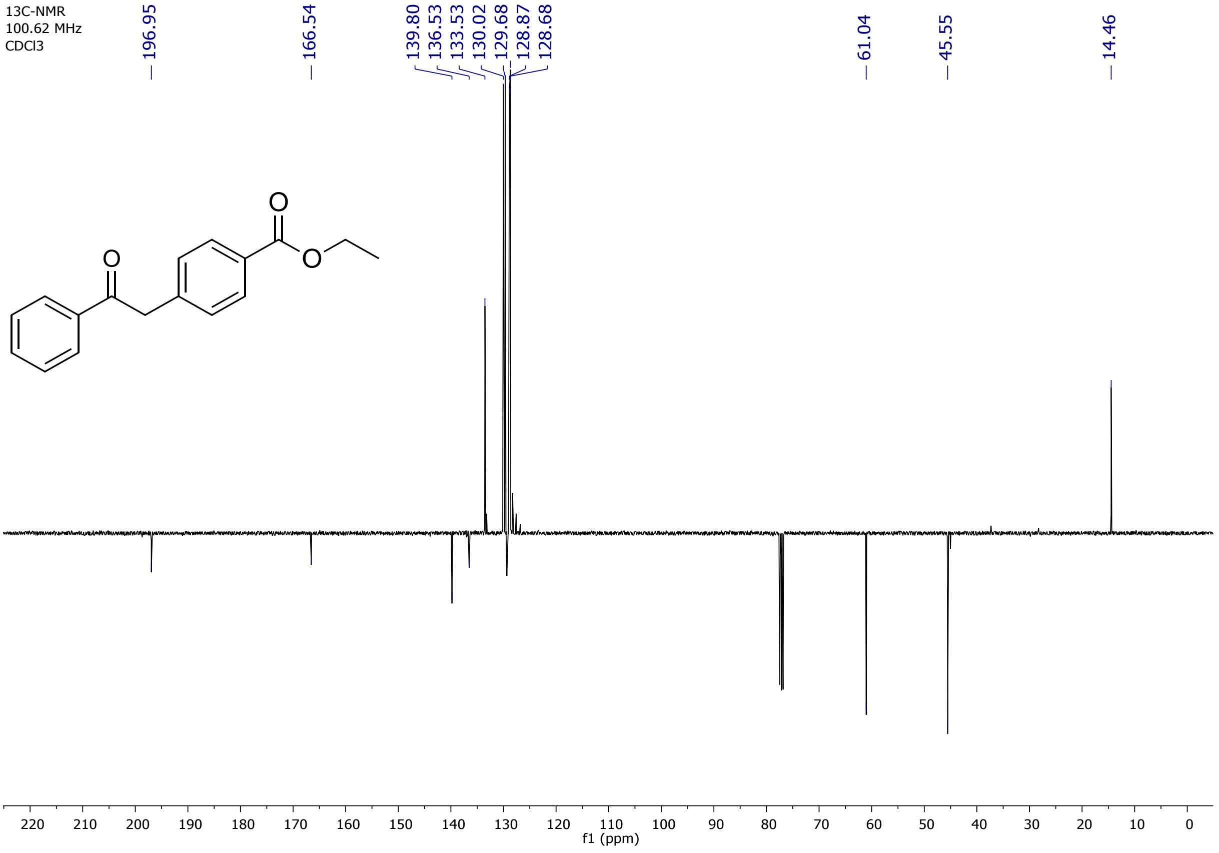

# 1-Fluoro-4-(1-methoxy-2-phenylethenyl)benzene (2a)

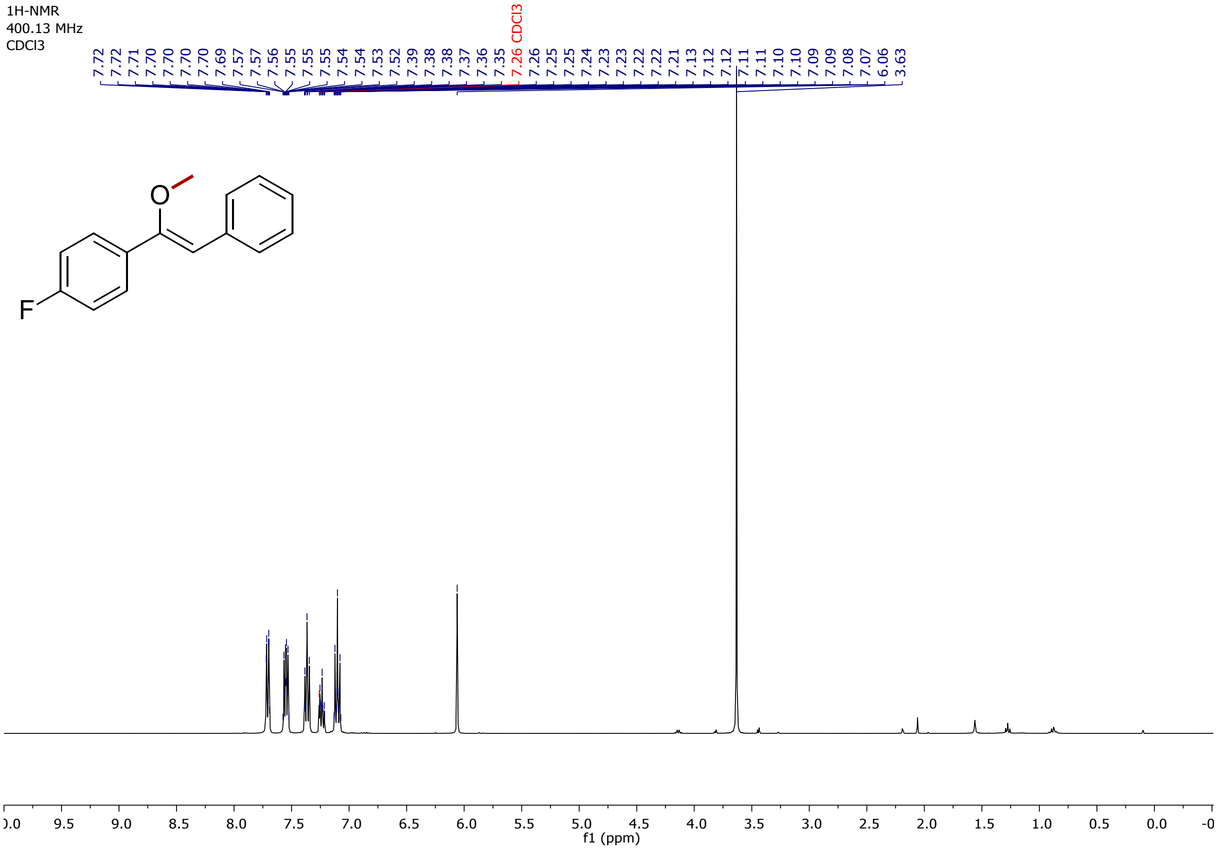


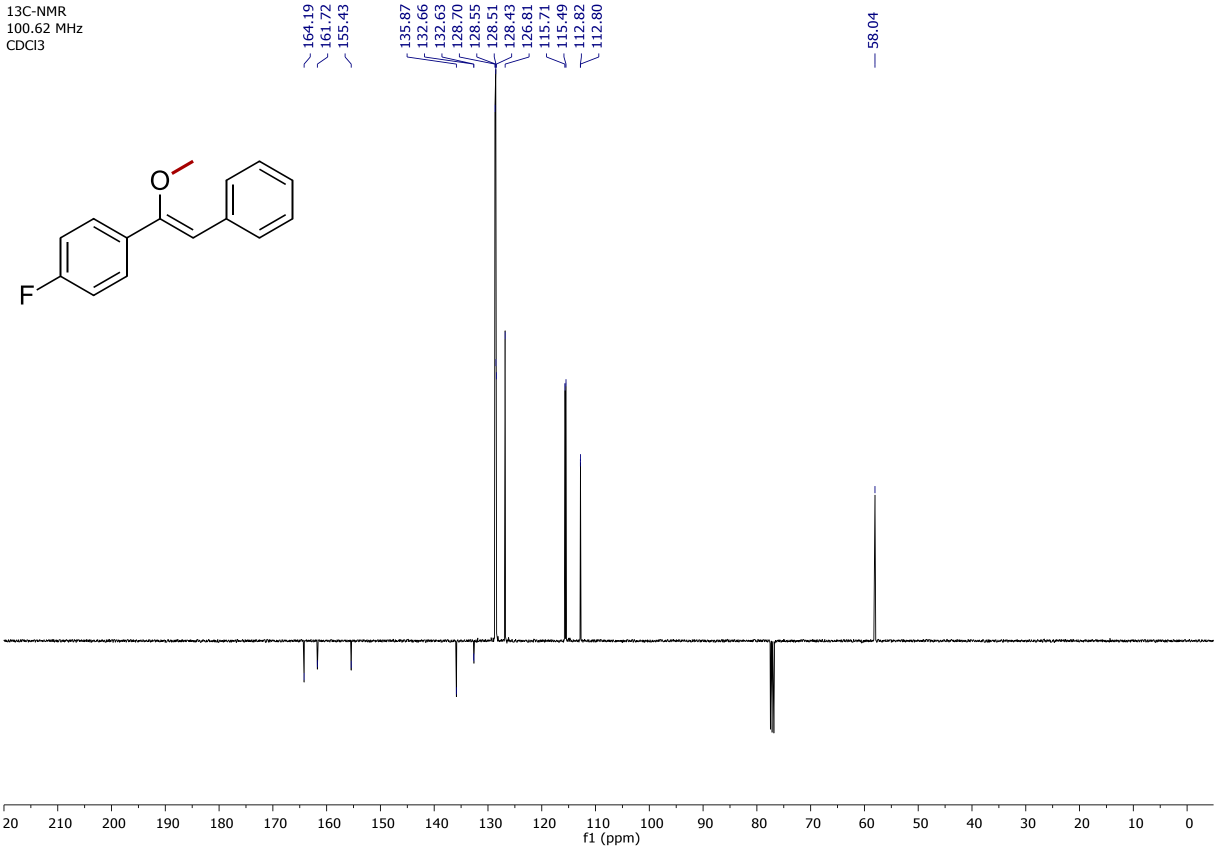

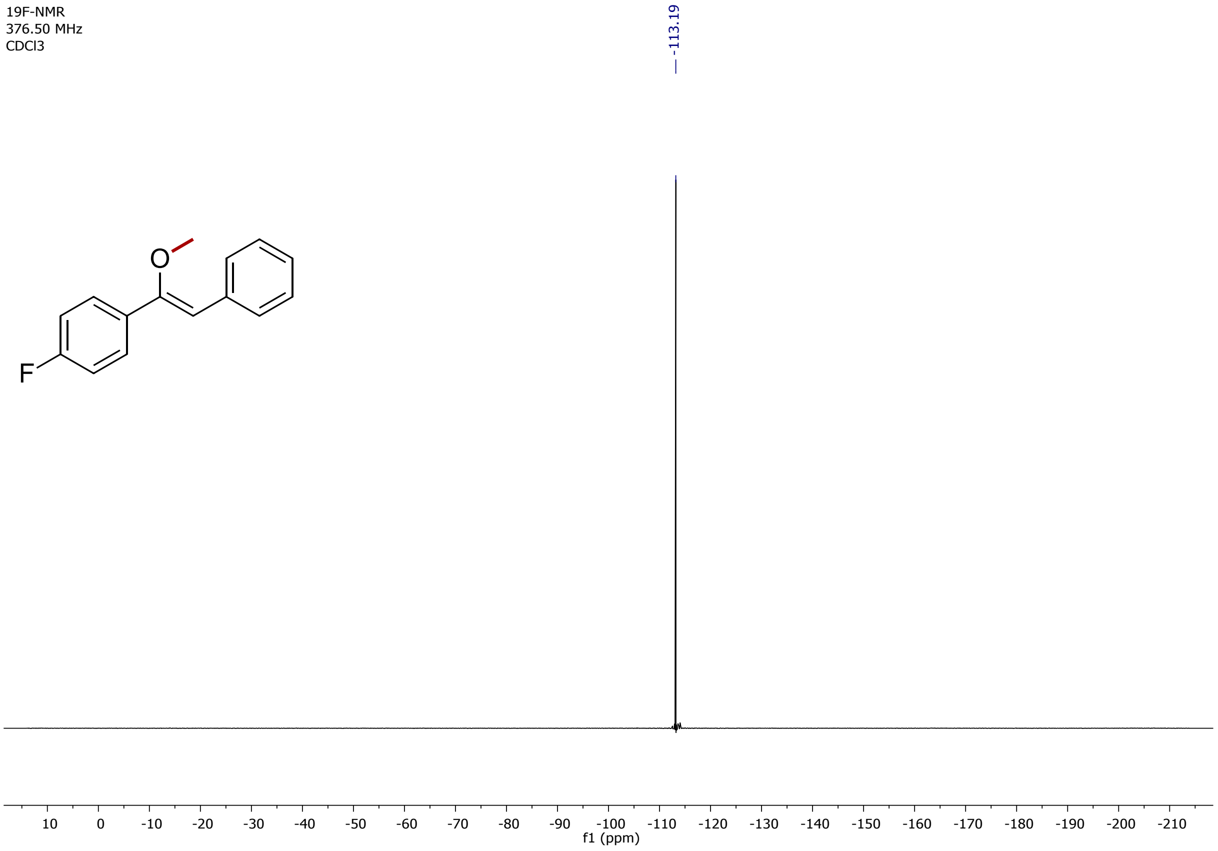
19F-NMR
376.50 MHz
CDCl3
-113.19
O
F
10 0 -10 -20 -30 -40 -50 -60 -70 -80 -90 -100 -110 -120 -130 -140 -150 -160 -170 -180 -190 -200 -210
f1 (ppm)

## 1-(4-Fluorophenyl)-2-phenyl-1-propanone (3a)

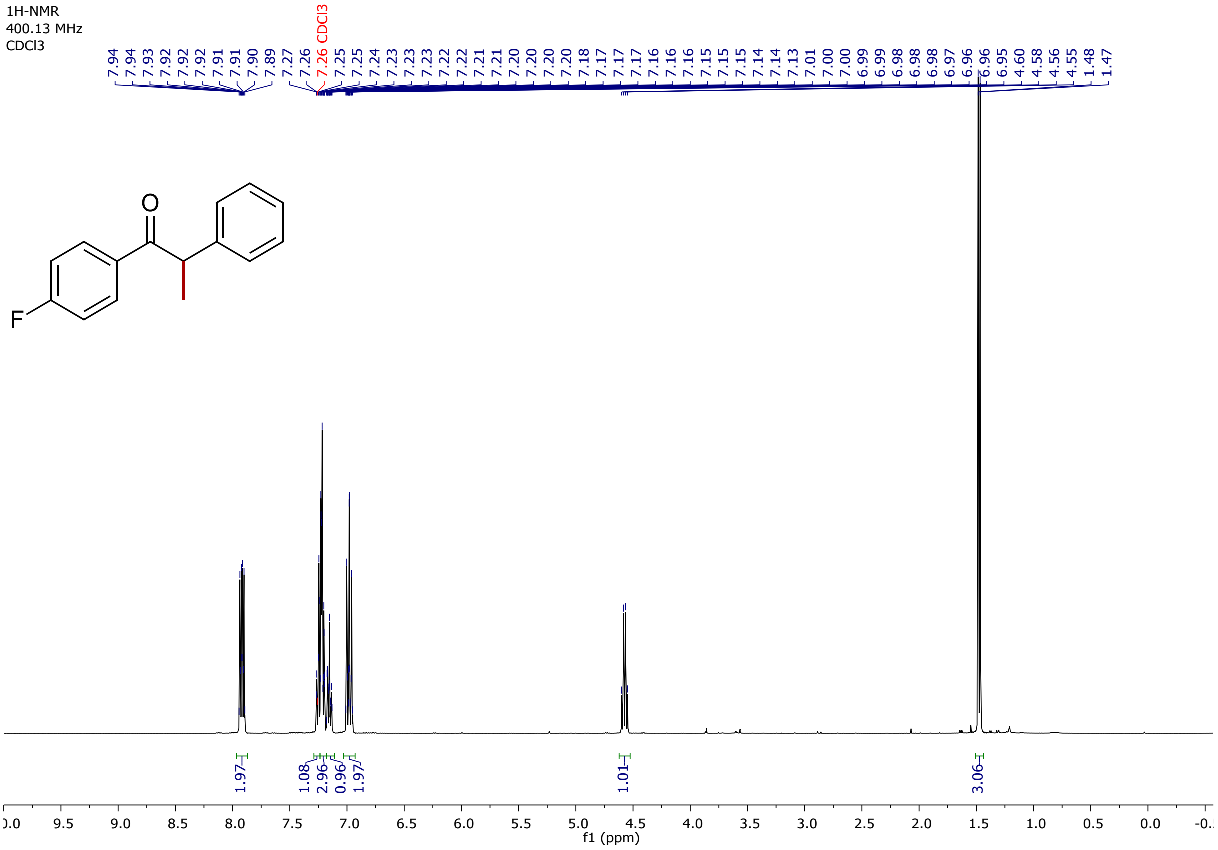


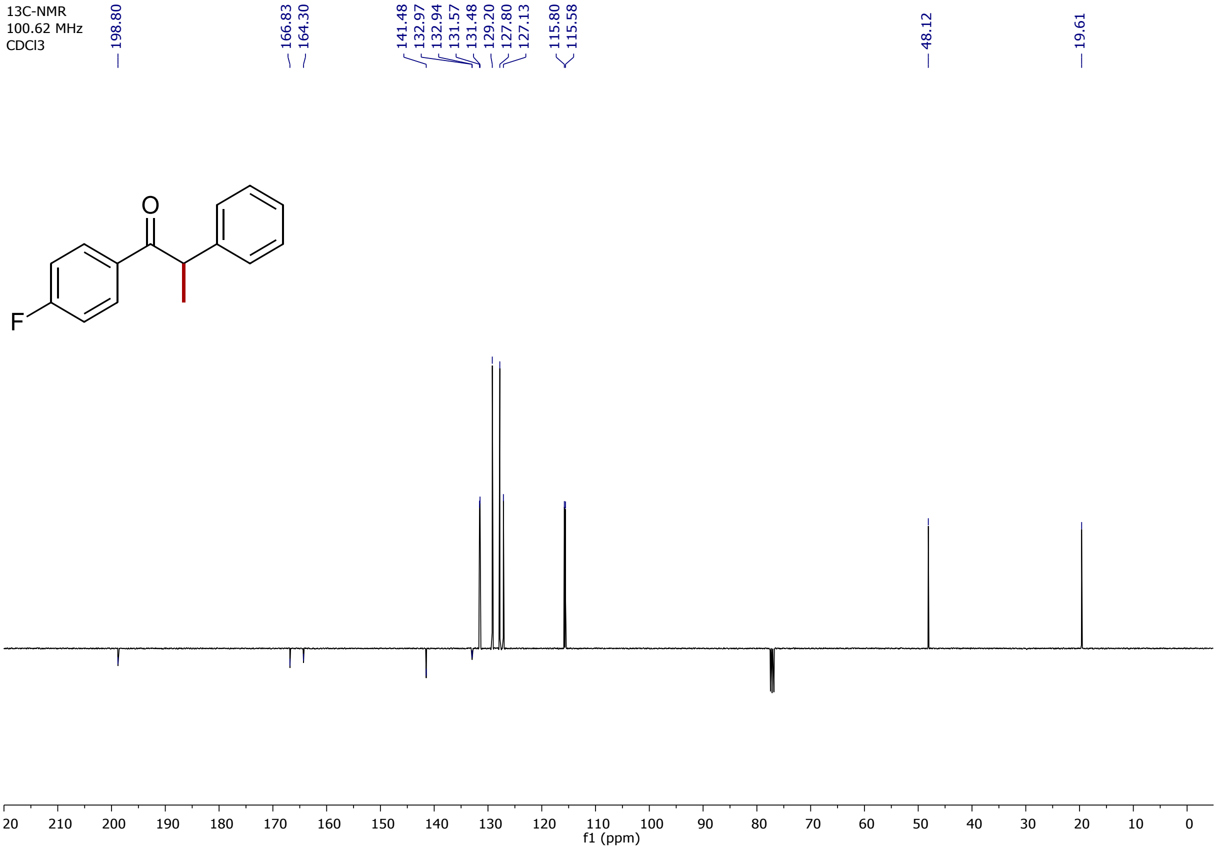

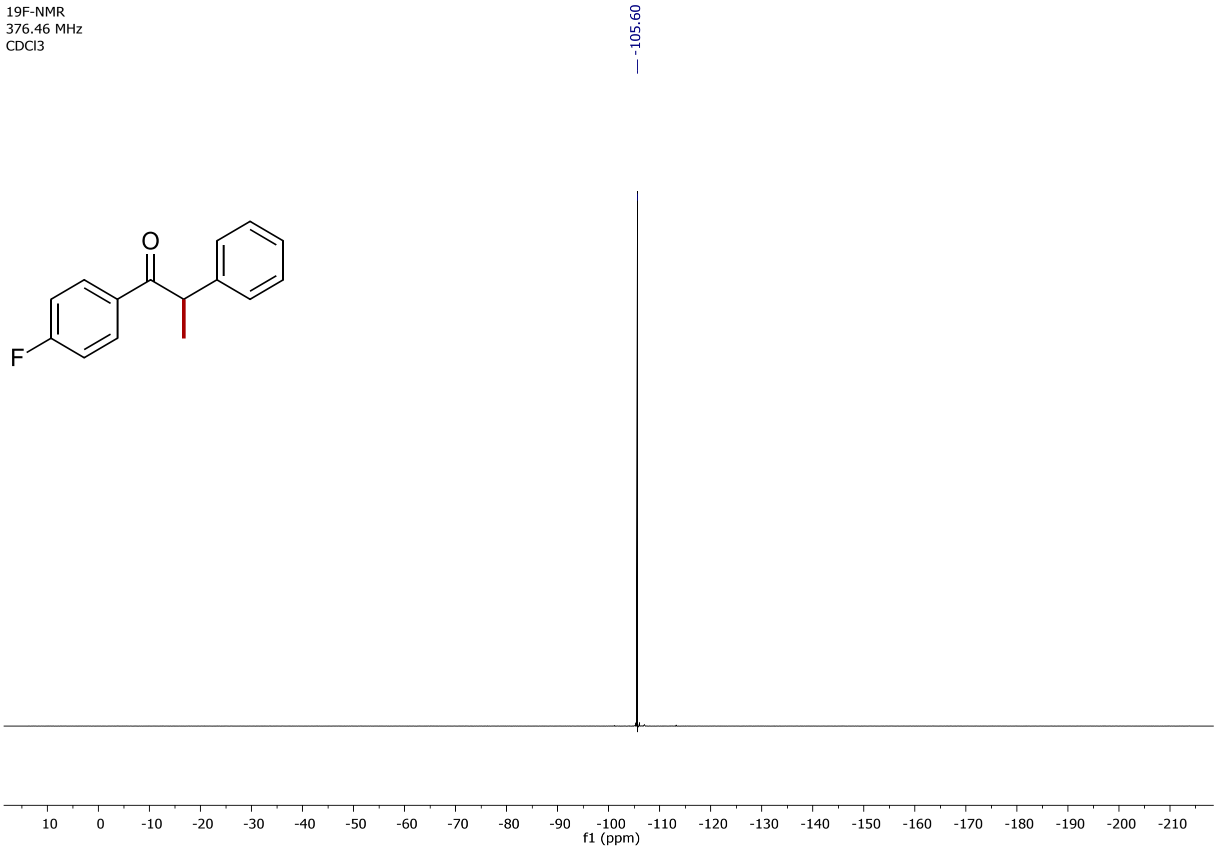
19F-NMR
376.46 MHz
CDCl3
-105.60
O
F
10 0 -10 -20 -30 -40 -50 -60 -70 -80 -90 -100 -110 -120 -130 -140 -150 -160 -170 -180 -190 -200 -210
f1 (ppm)

## 1,2-Diphenyl-1-propanone (3b)

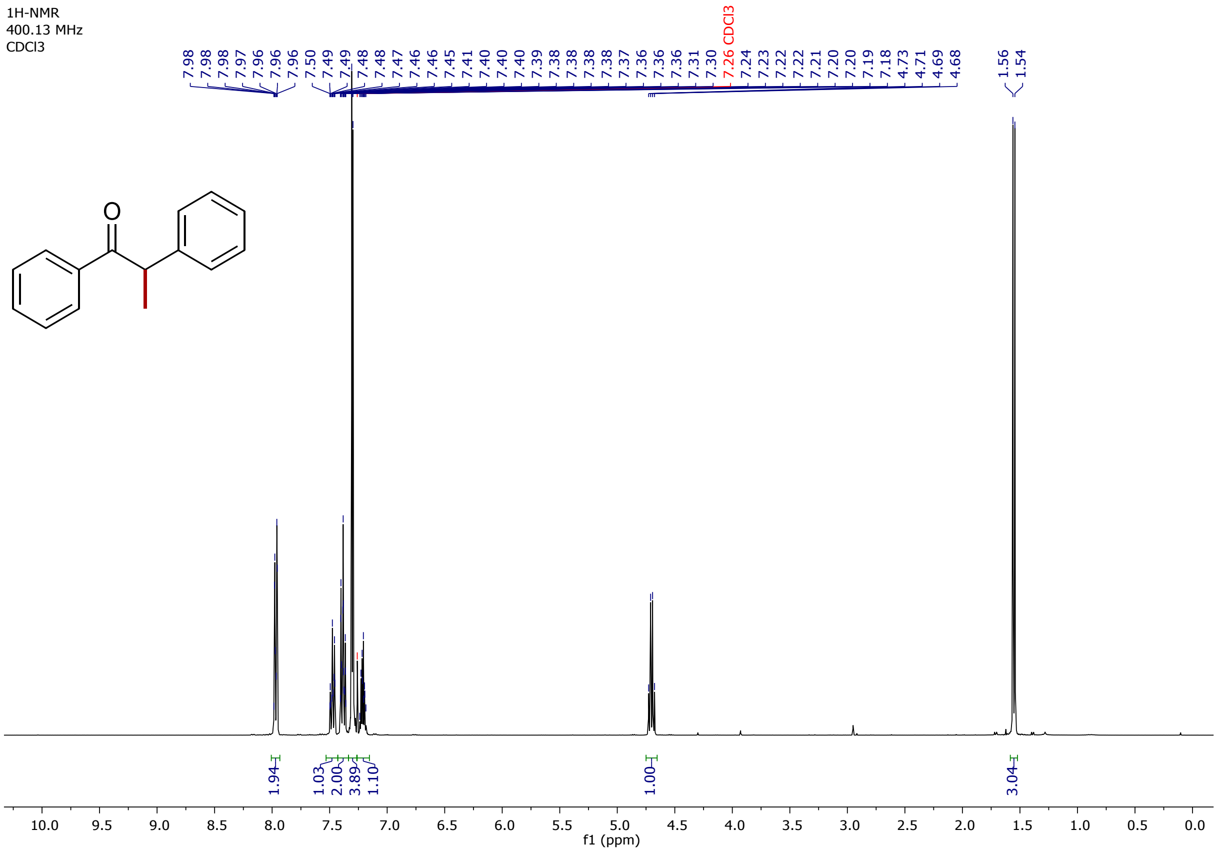


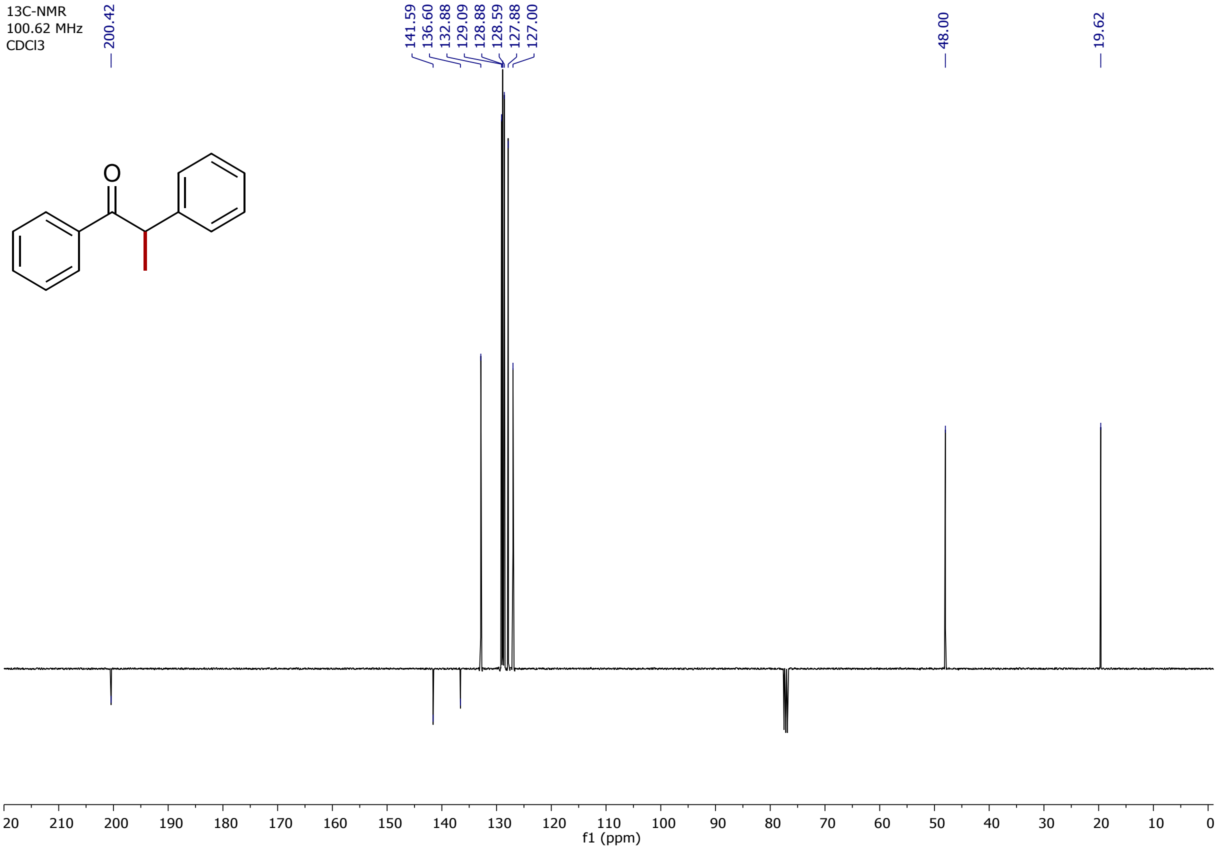

# 2-(4-Methylphenyl)-1-phenyl-1-propanone (3c)

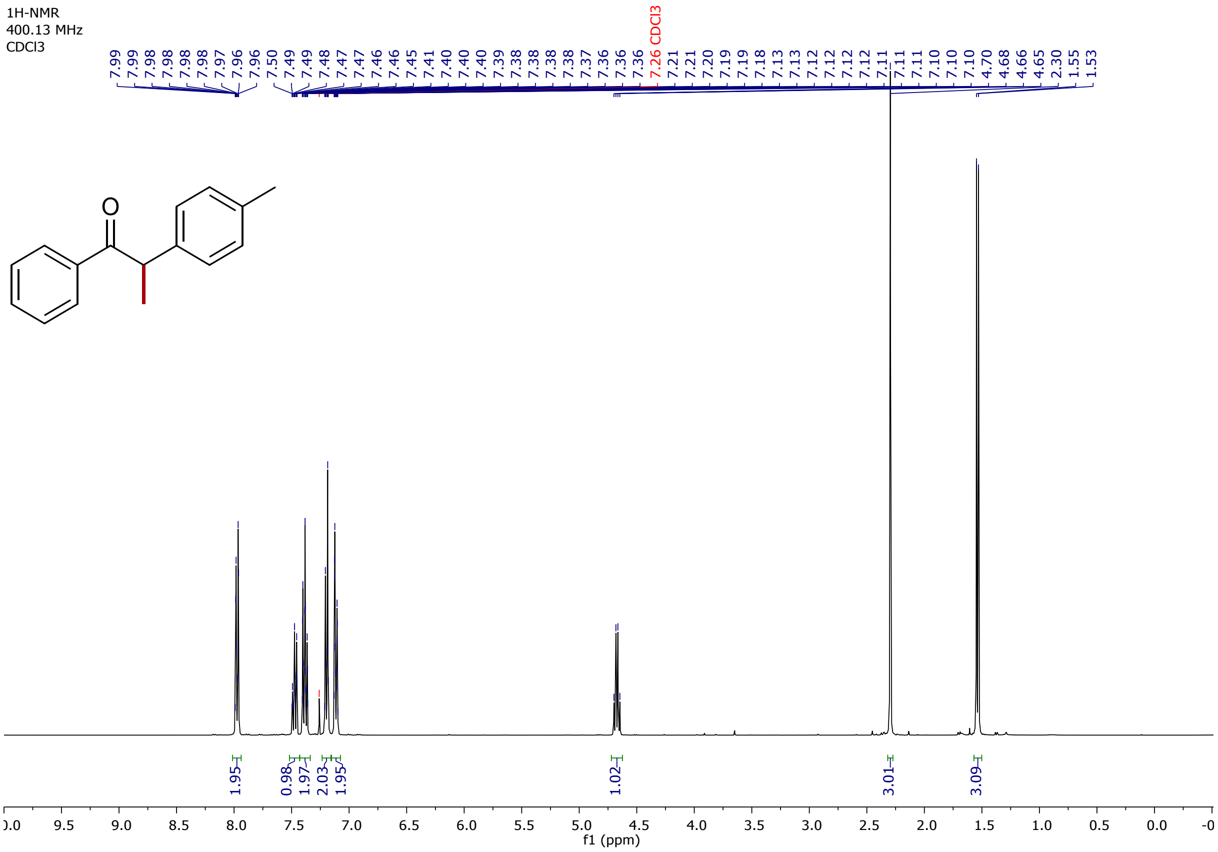


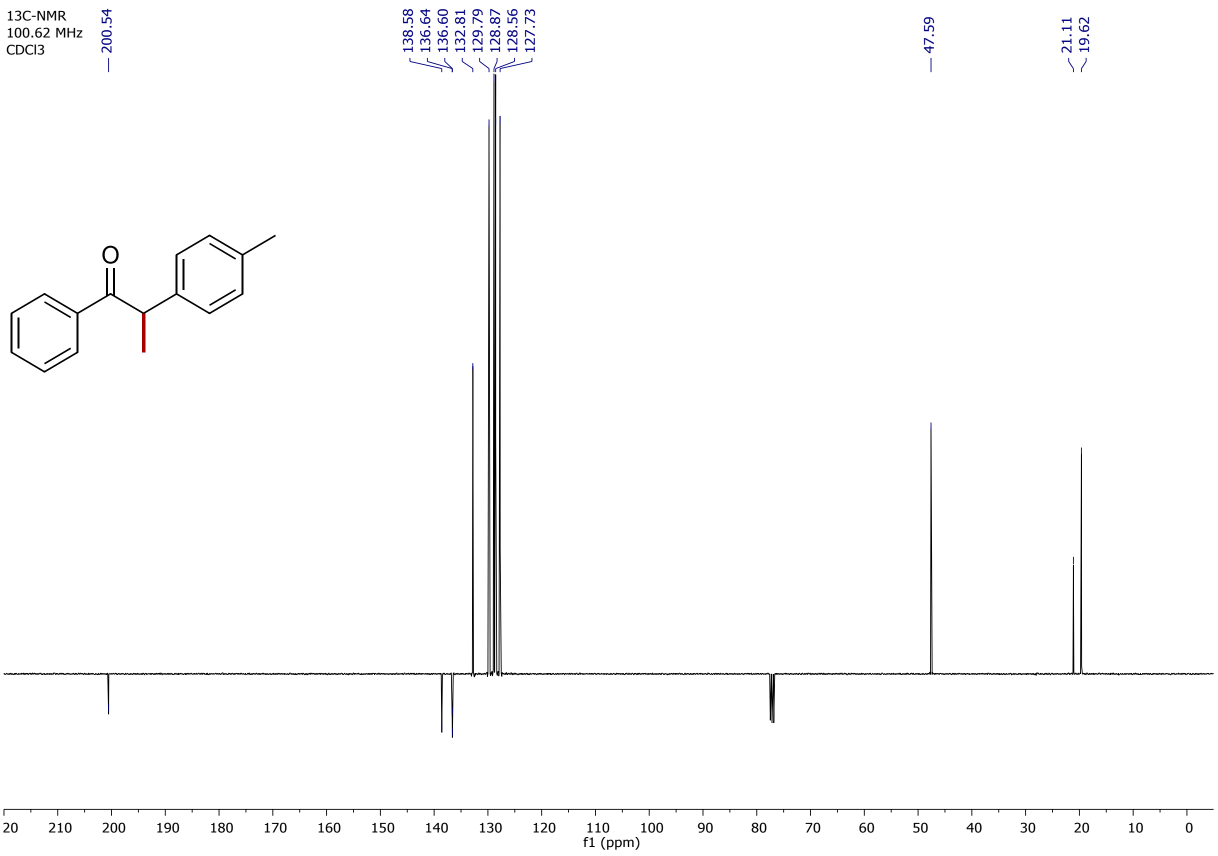

## 2-(2,3,4,5,6-Pentamethylphenyl)-1-phenyl-1-propanone (3d)

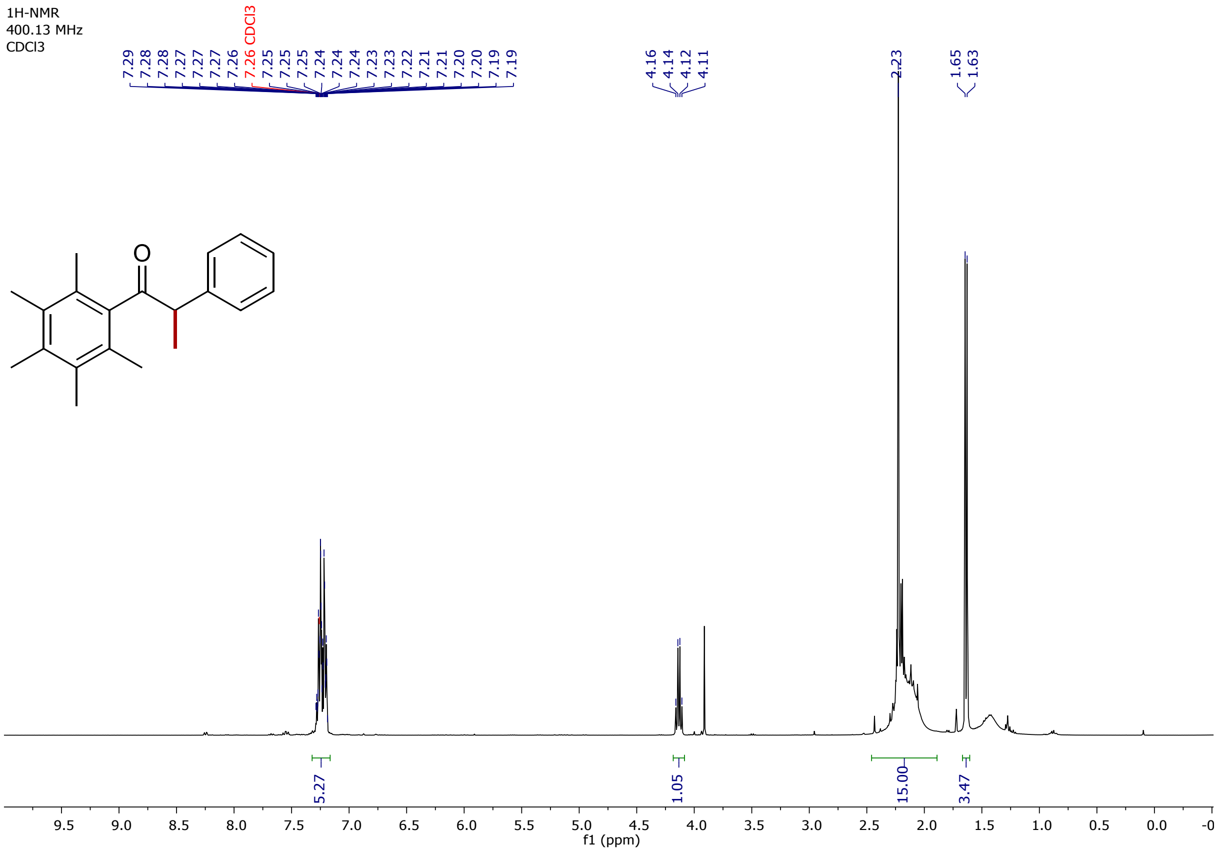


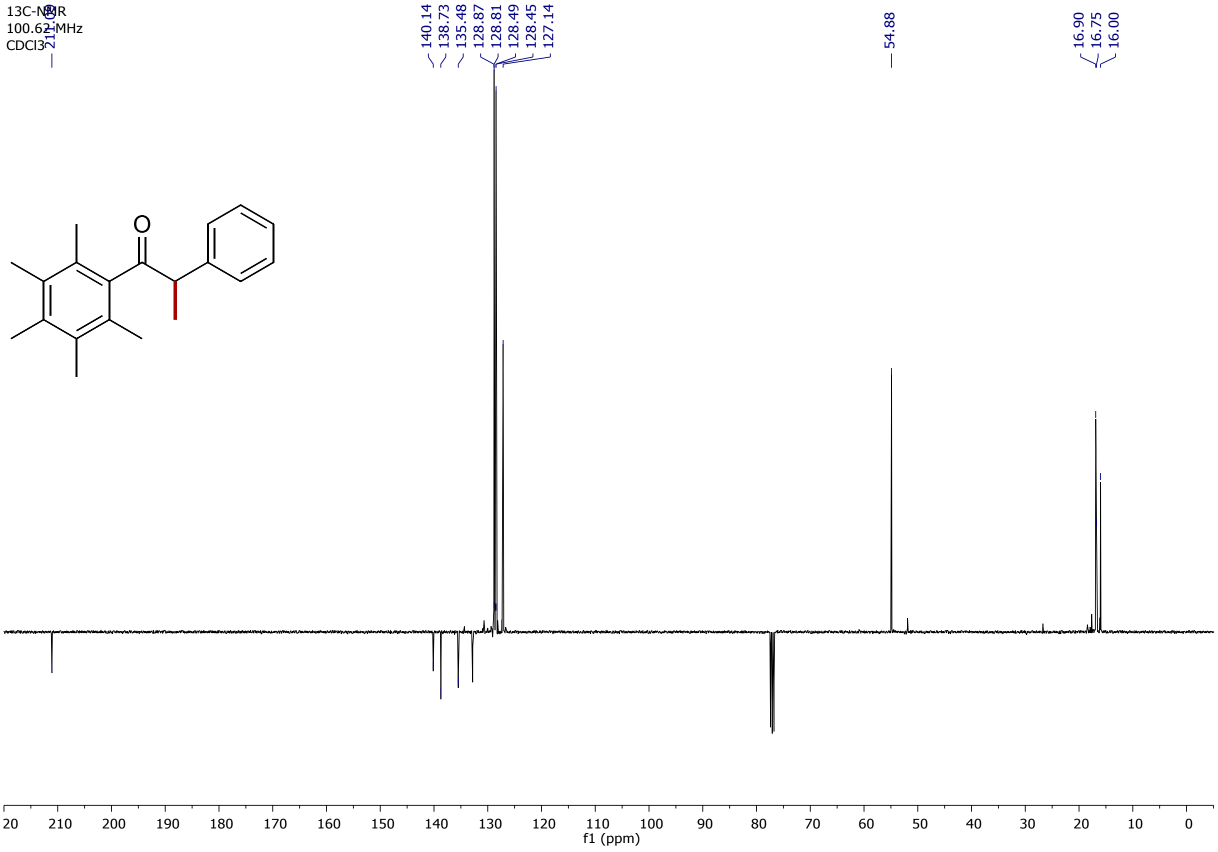

## 1-(4-Chlorophenyl)-2-phenyl-1-propanone (3e)

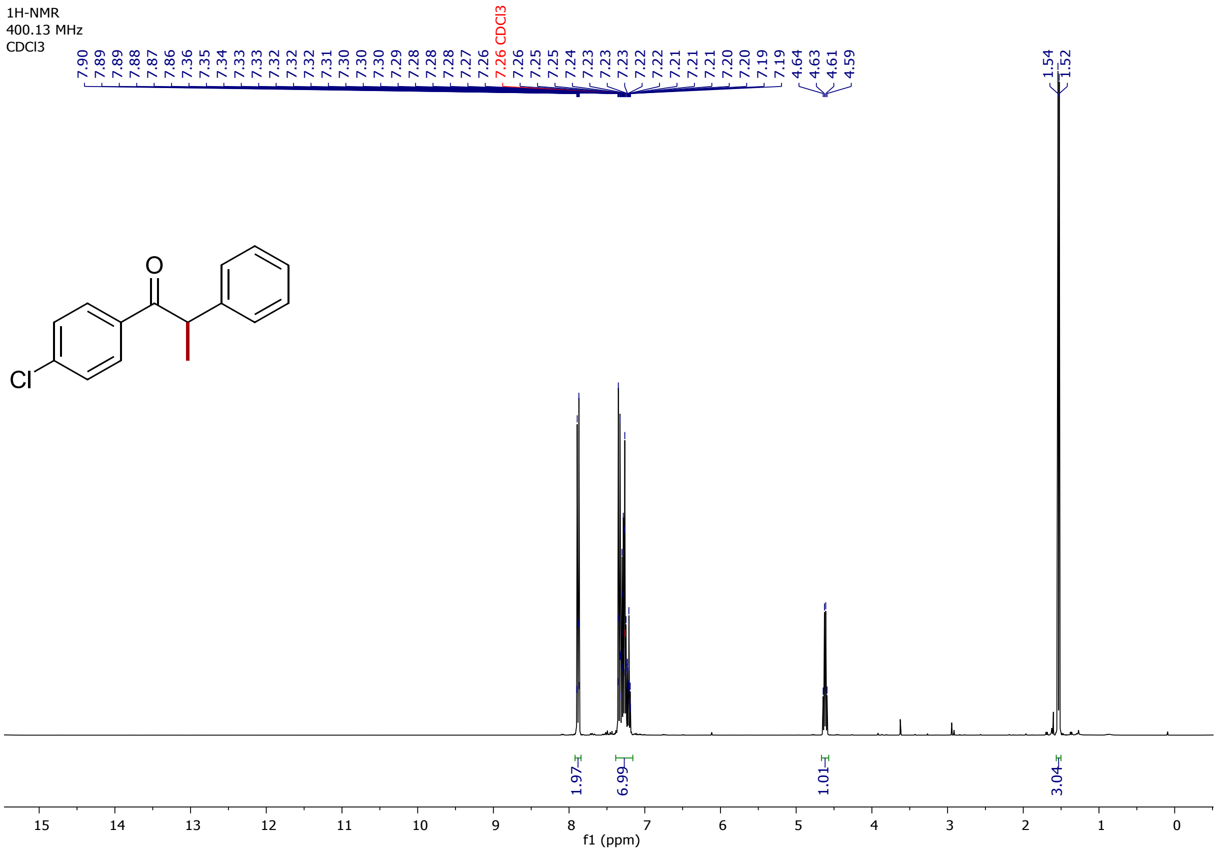


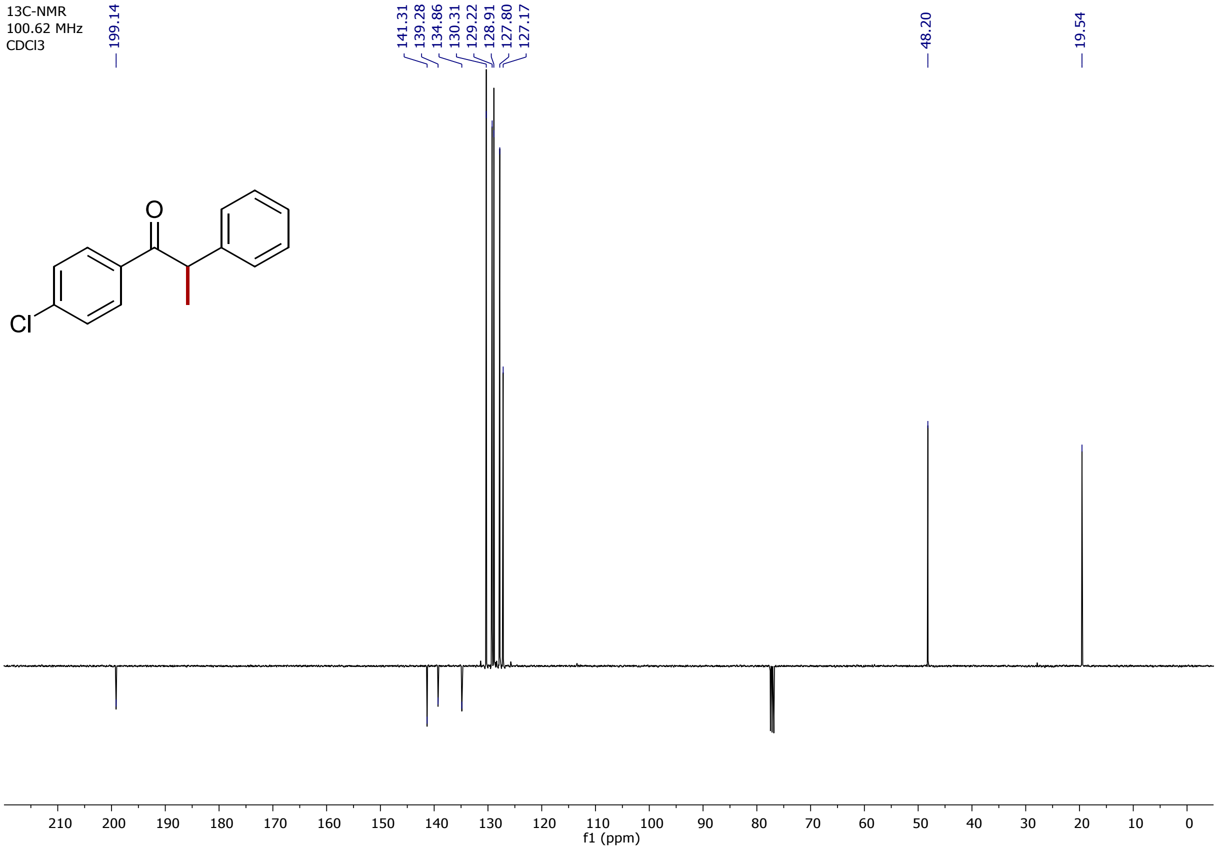

## 1-(4-Bromophenyl)-2-phenyl-1-propanone (3f)

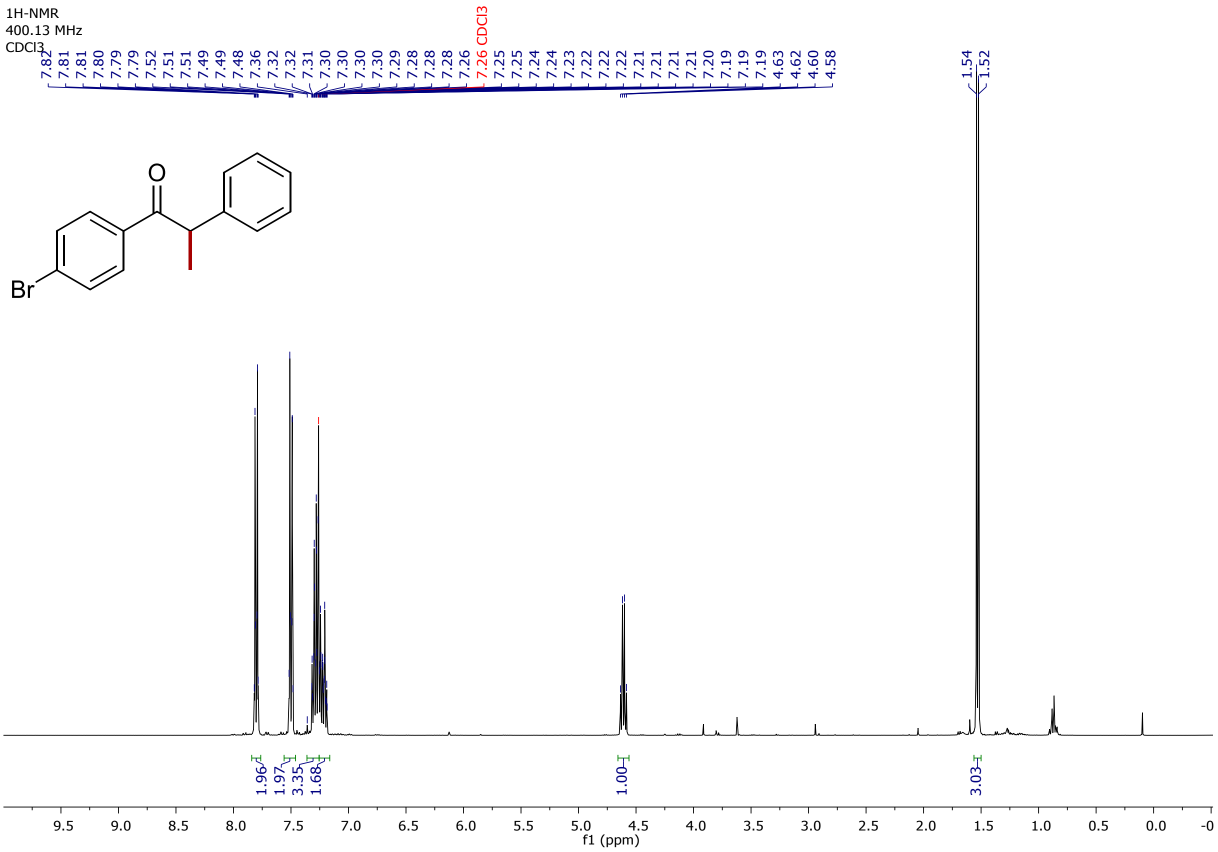


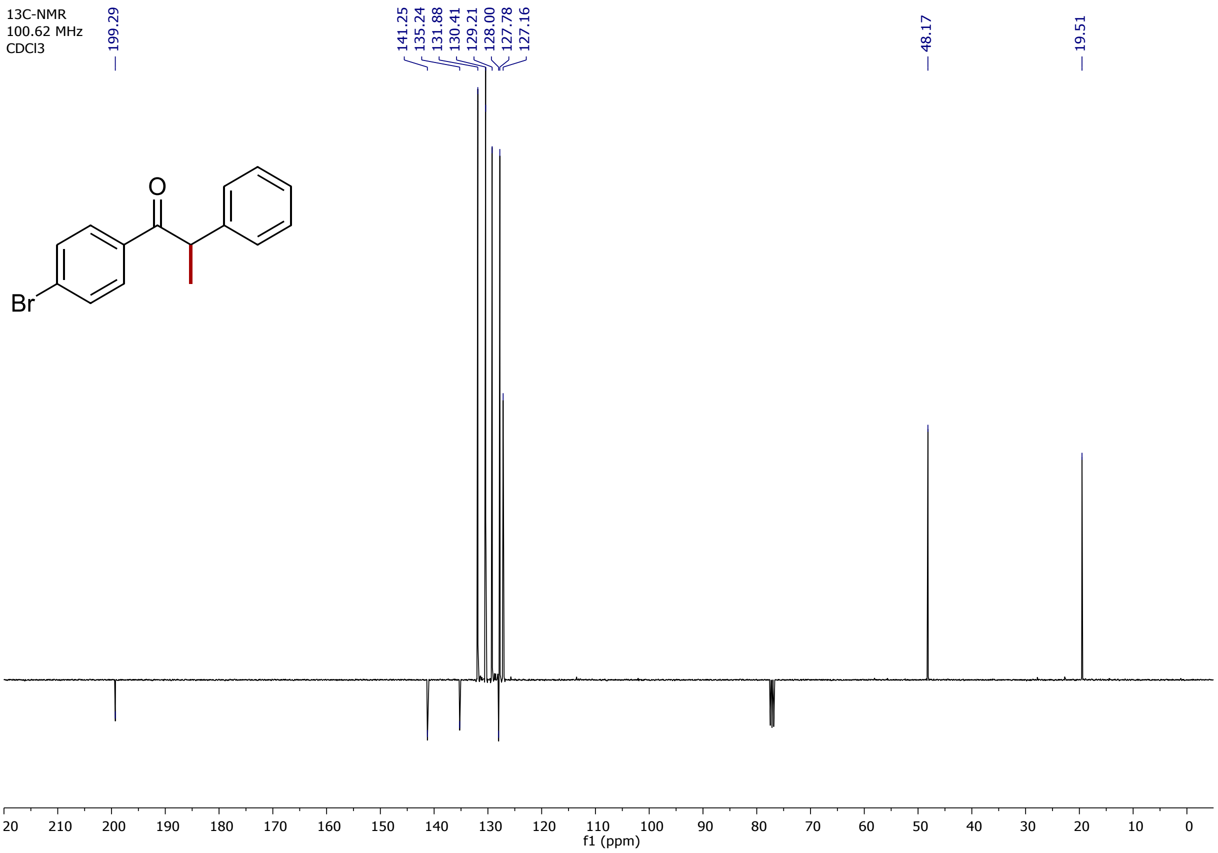

## 1-(3,4-Dimethoxyphenyl)-2-phenyl-1-propanone (3g)

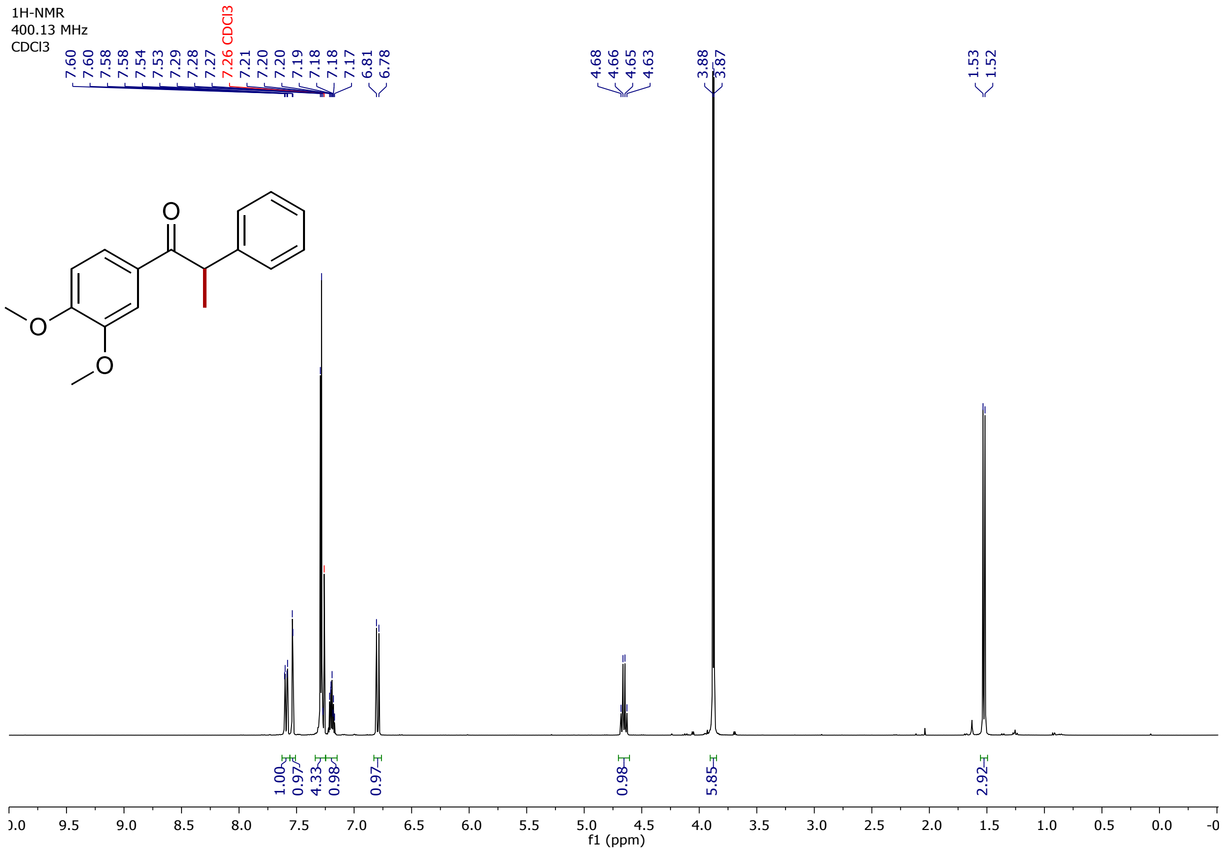


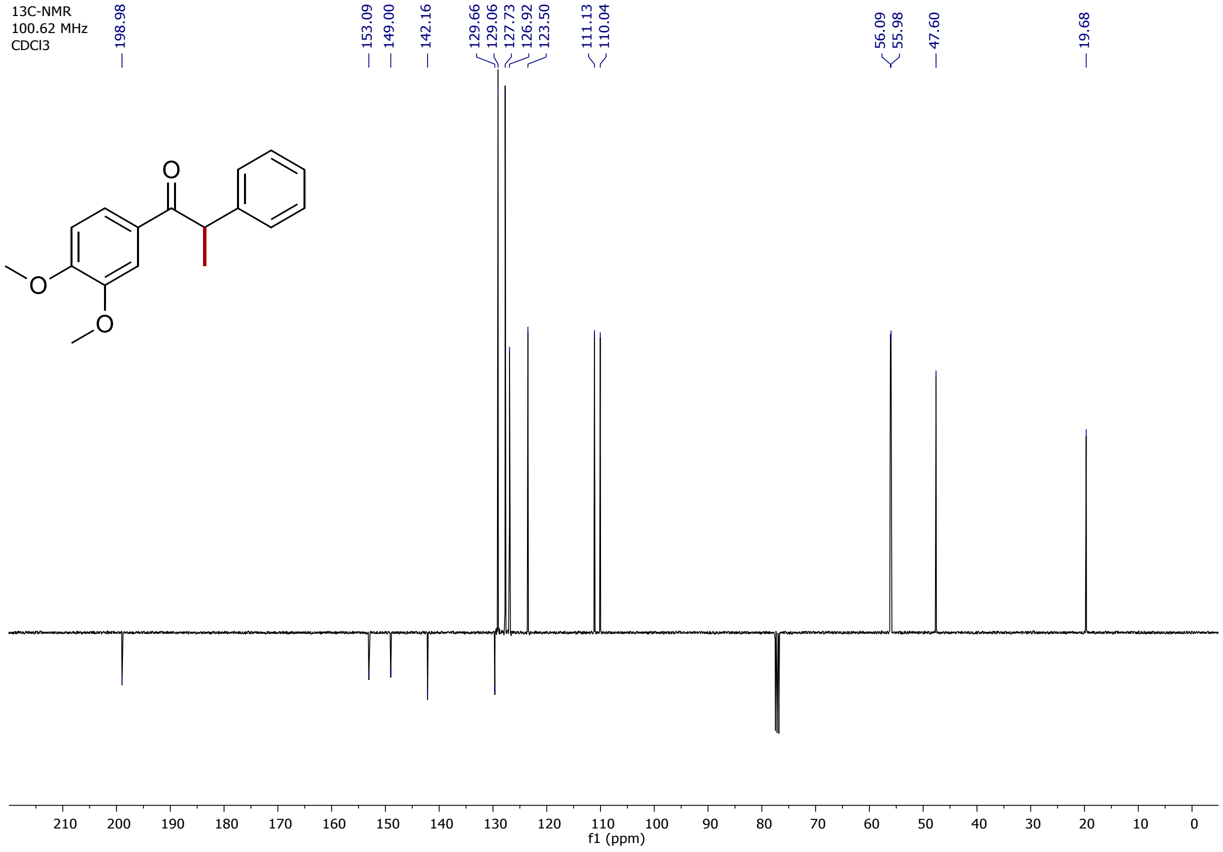

# 1-(3-Methoxyphenyl)-2-phenyl-1-propanone (3h)

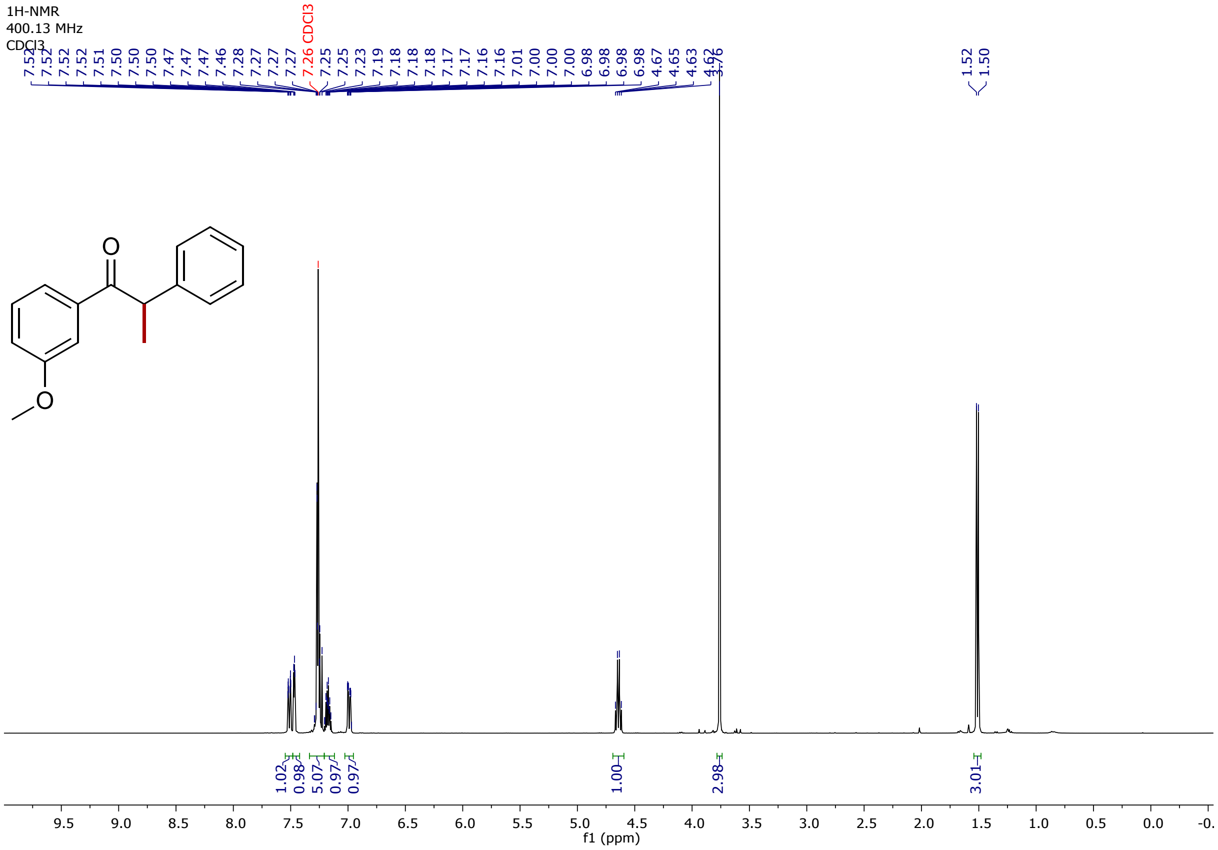


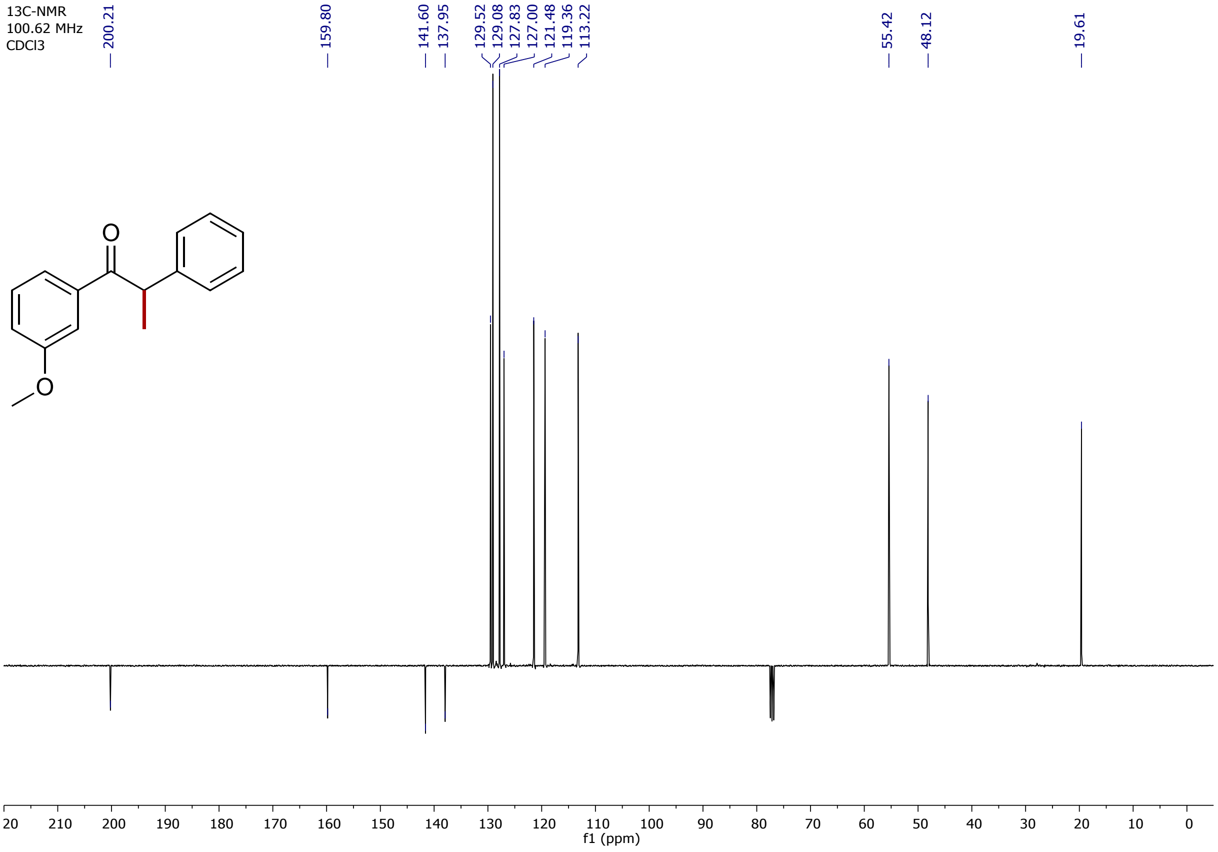

## 2-Phenyl-1-[3-(trifluoromethyl)phenyl]-1-propanone (3i)

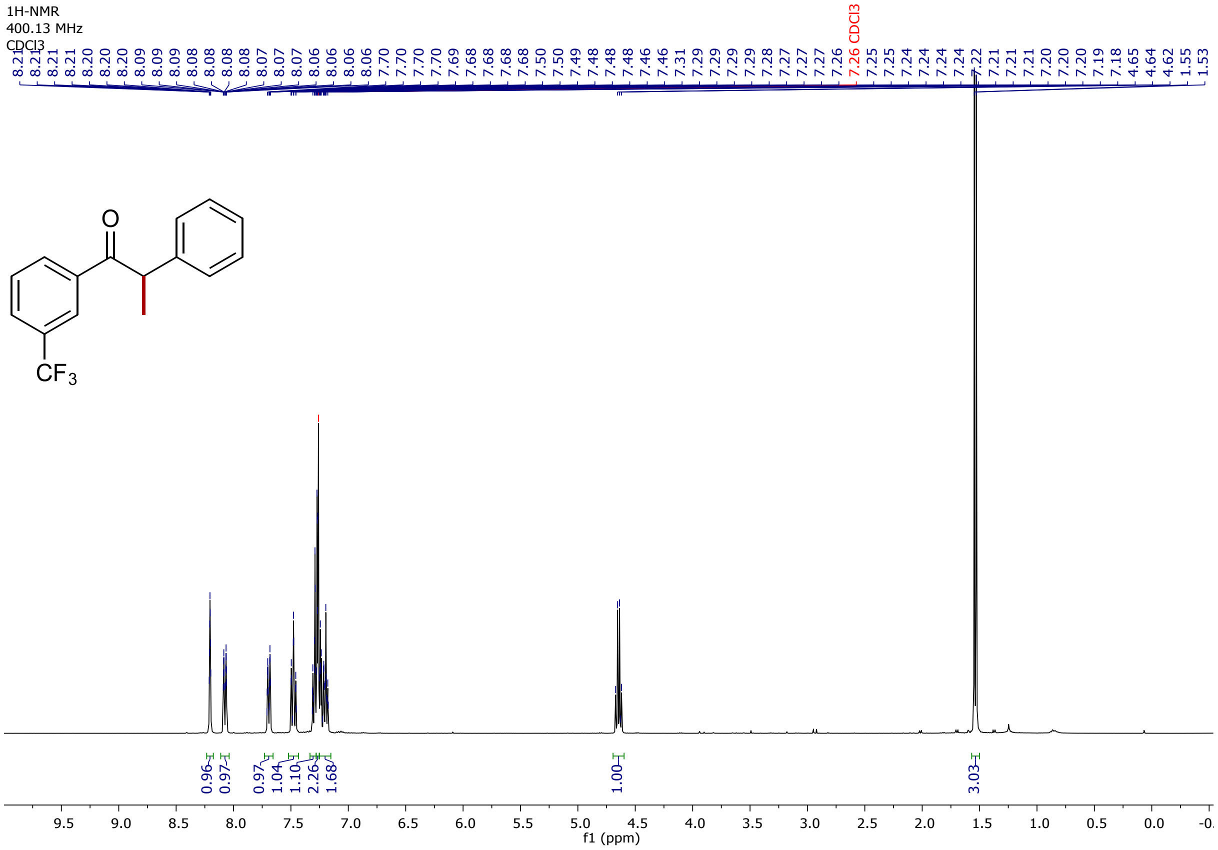


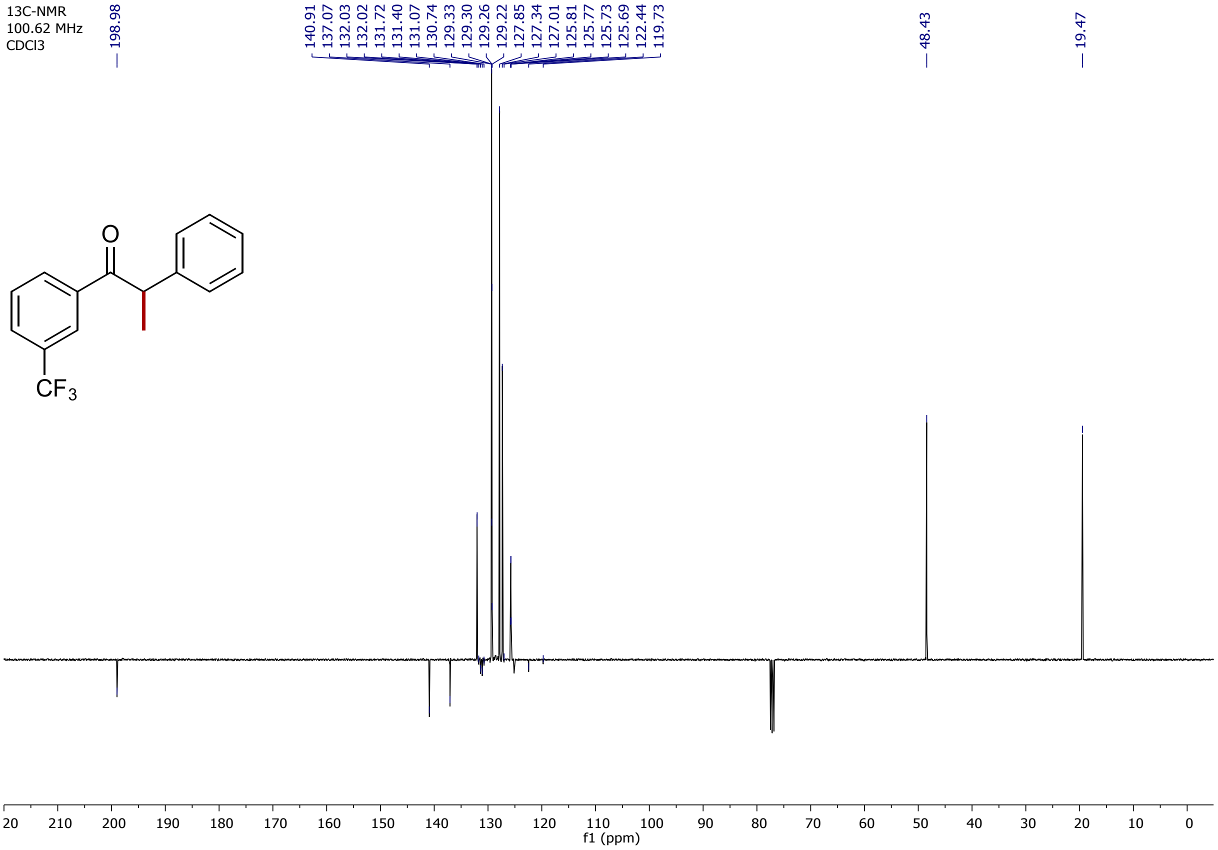

# 1-[4-(2-Methylpropyl)phenyl]-2-phenyl-1-propanone (3j)

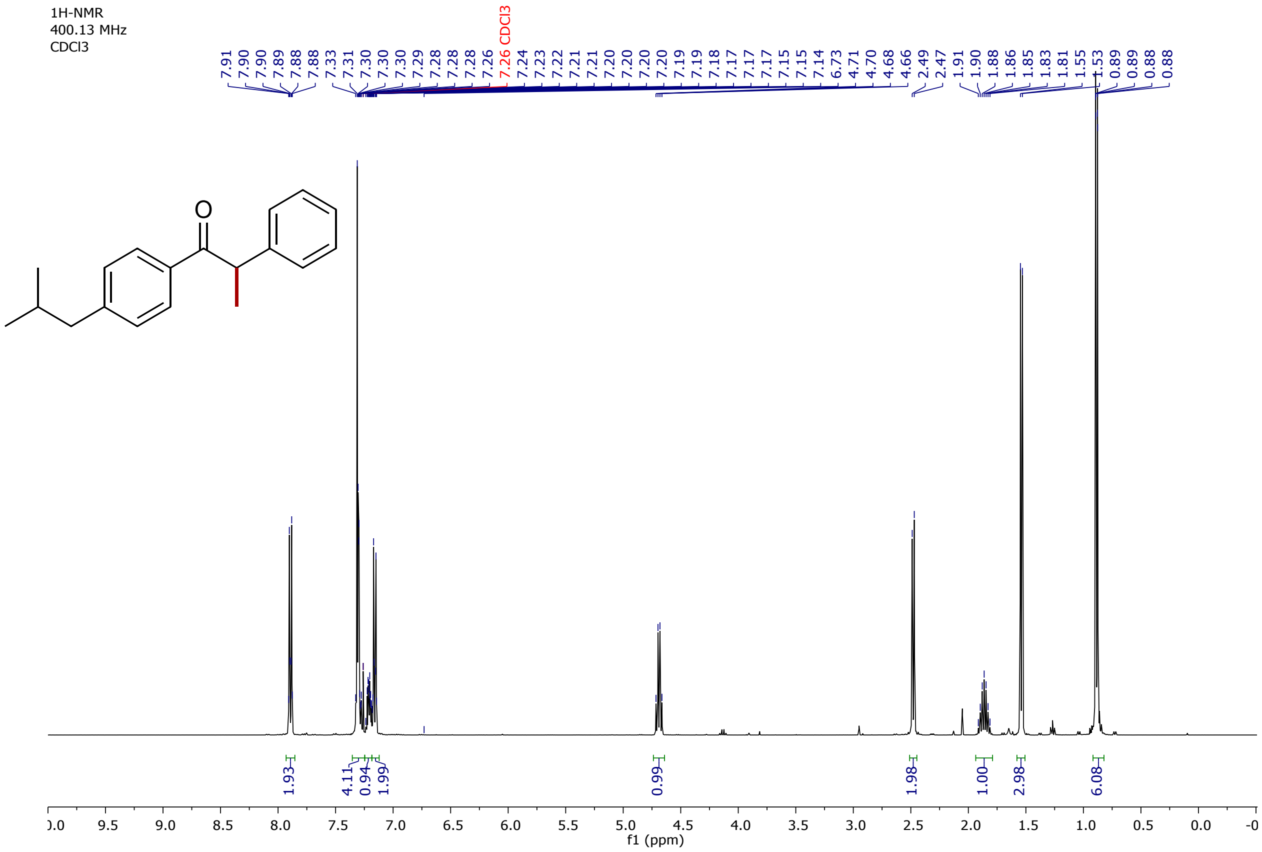


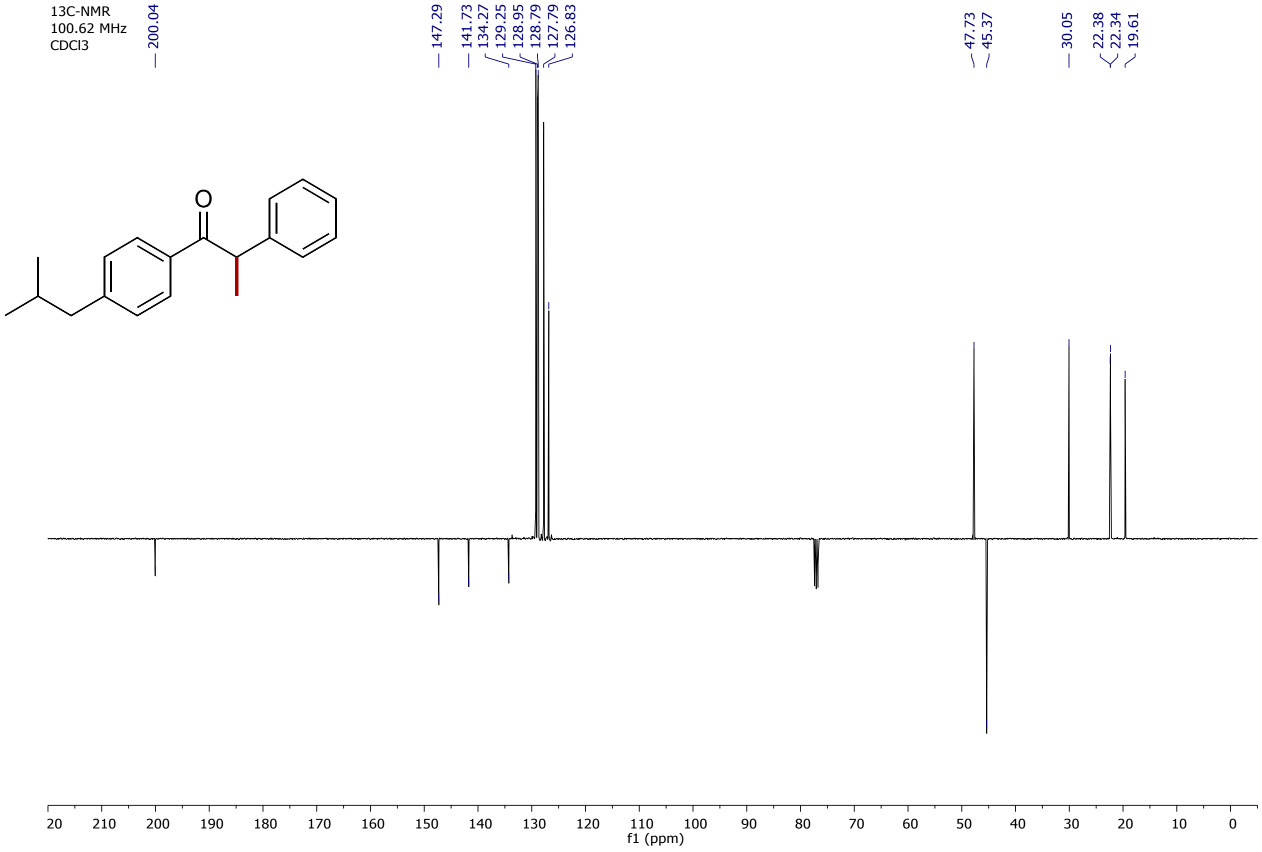

## 1-(4-Methylphenyl)-2-phenyl-1-propanone (3k)

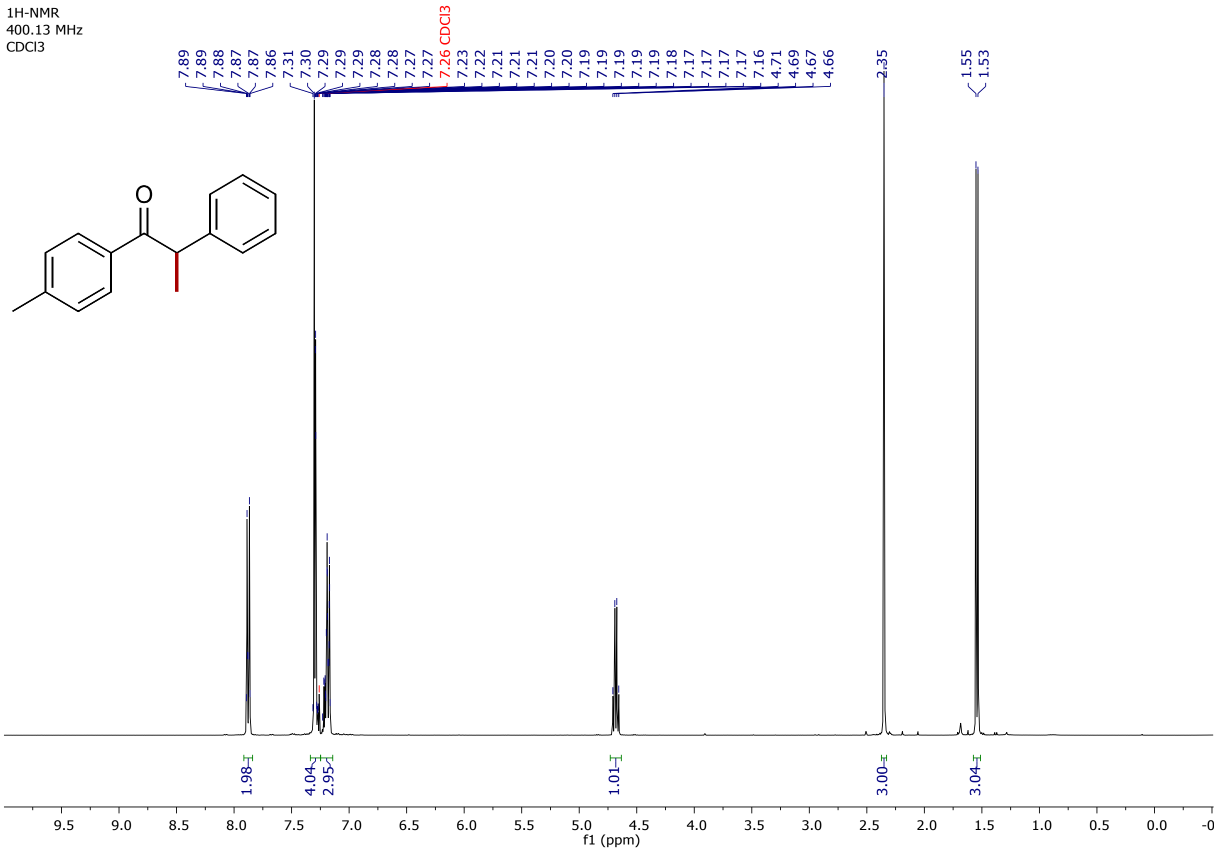


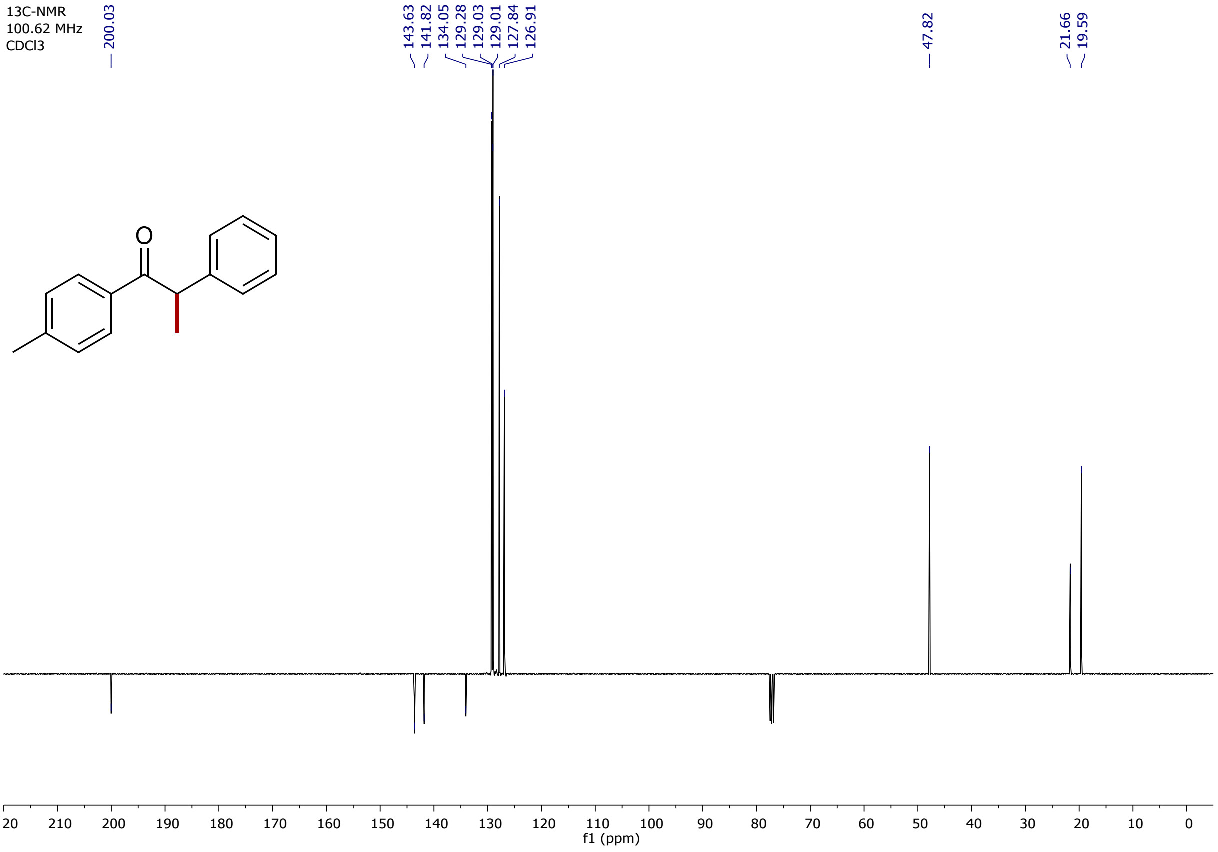

# 1-(2-Naphthyl)-2-phenyl-1-propanone (3l)

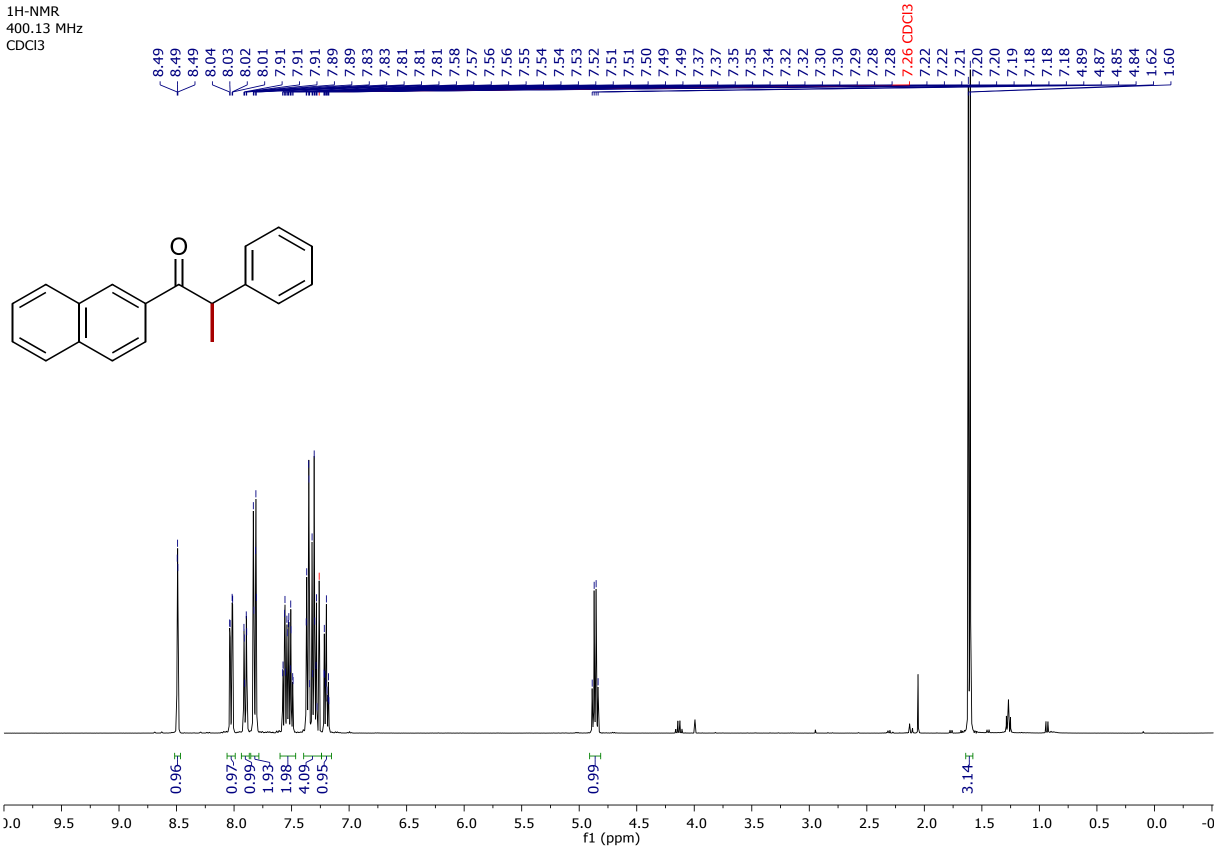


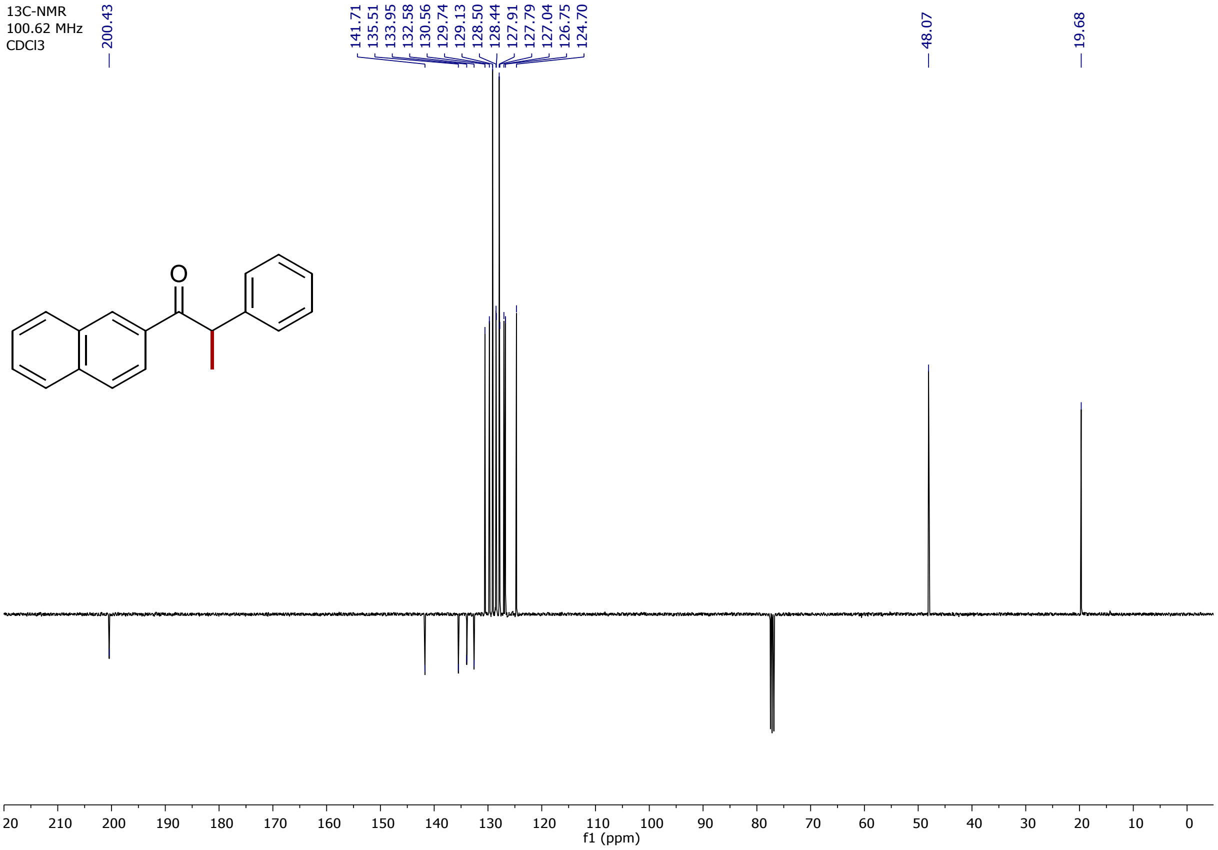

## 1,2-Bis(4-methoxyphenyl)-1-propanone (3m)

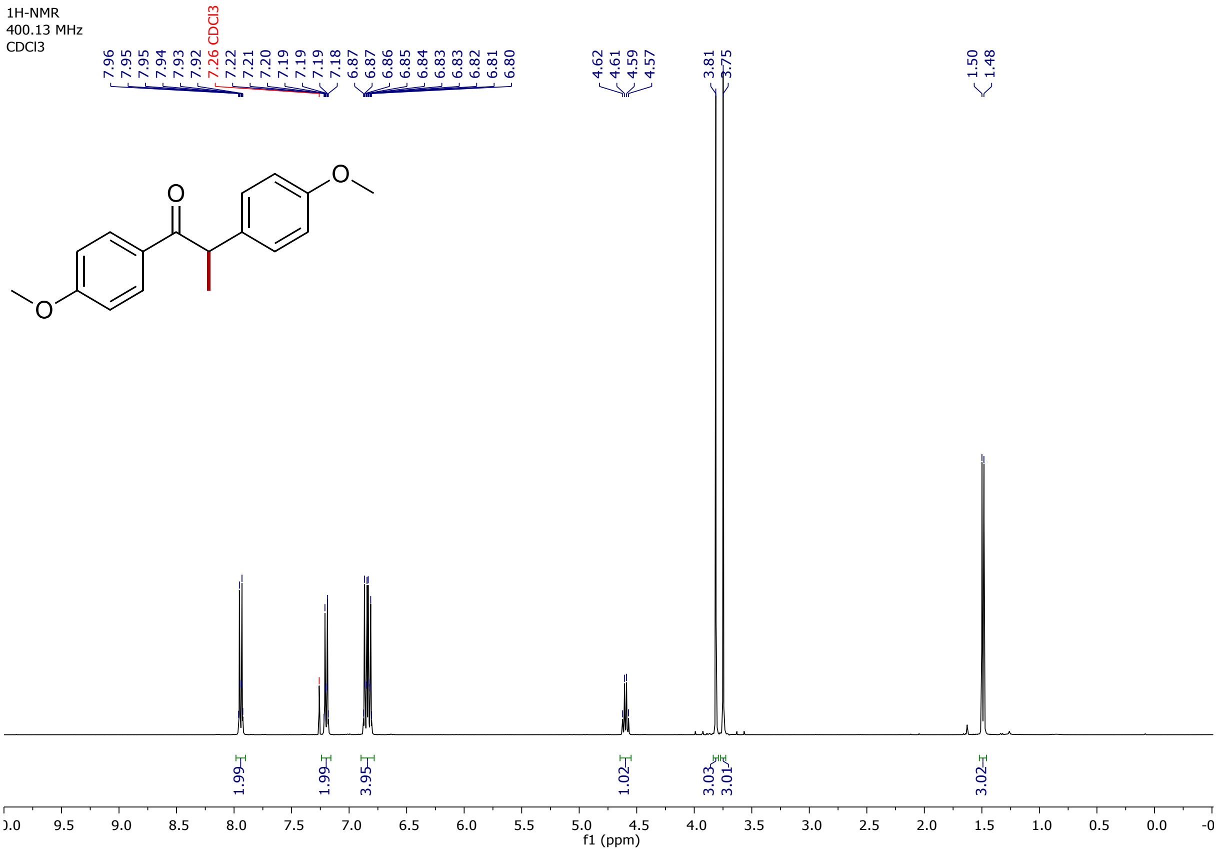


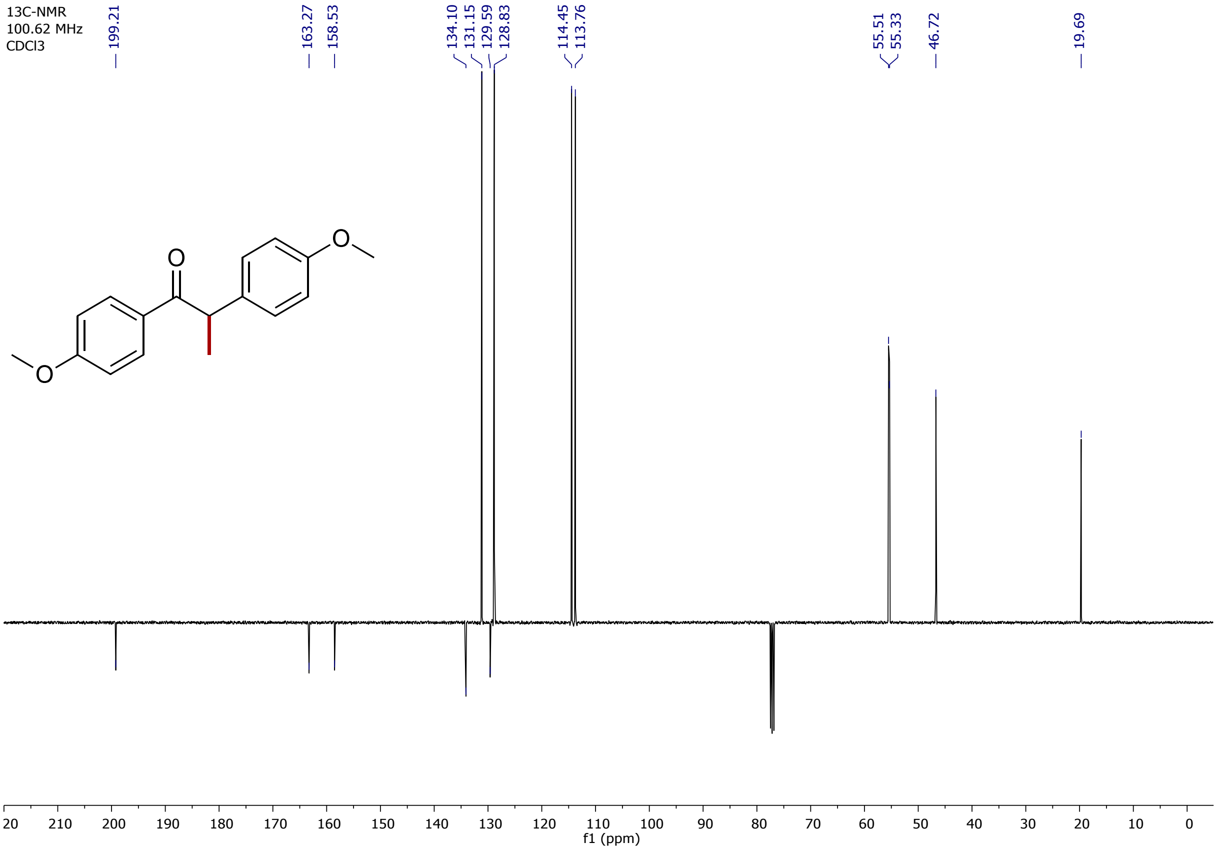

# 1-(2H-1,3-Benzodioxol-5-yl)-2-phenyl-1-propanone (3n)

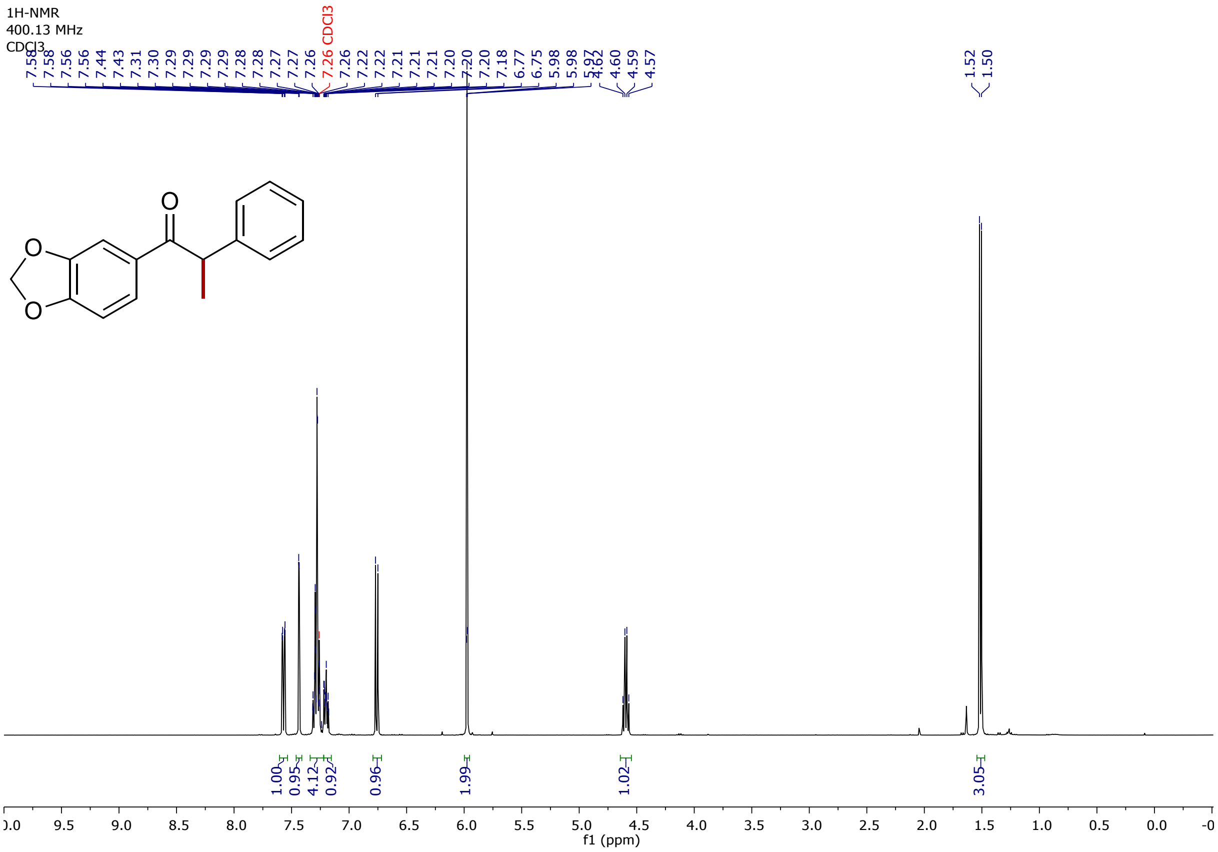


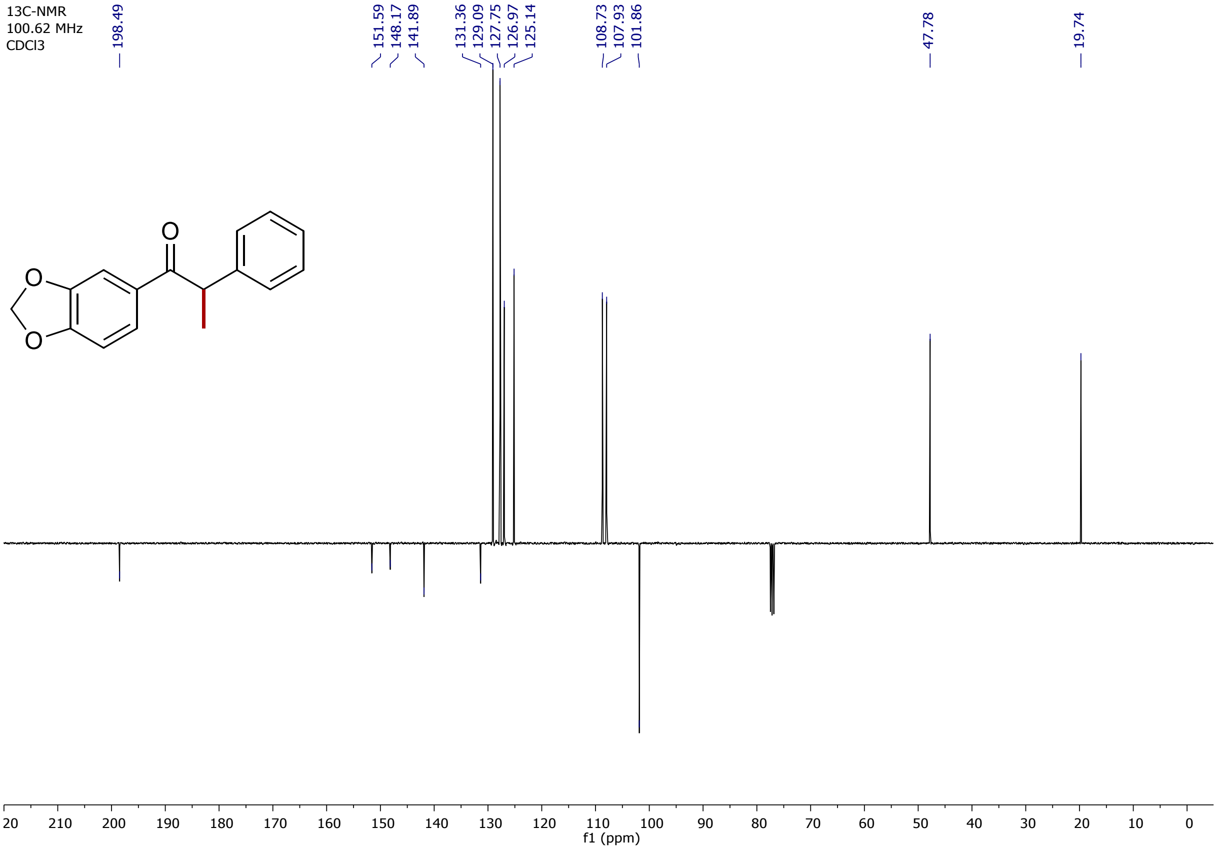

## 2-(3-Methoxyphenyl)-1-phenyl-1-propanone (3o)

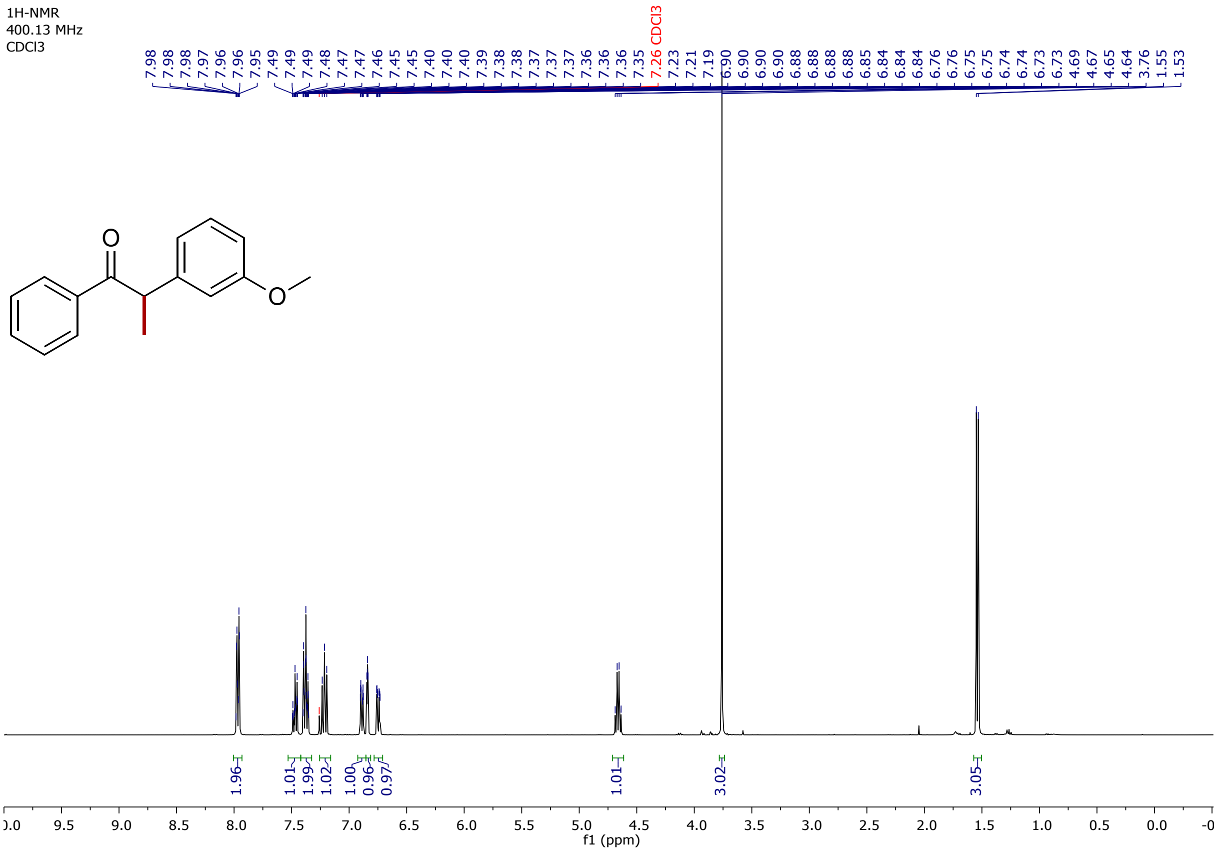


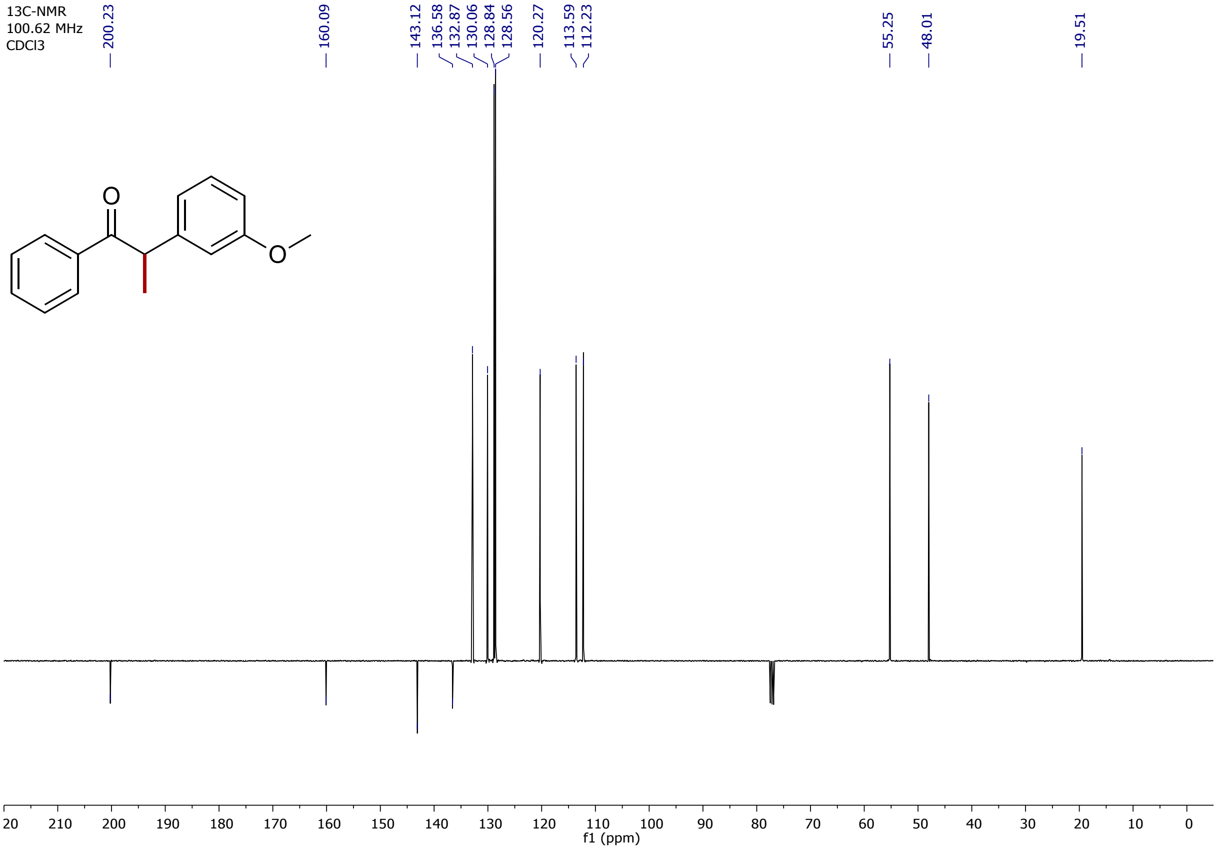

## 1-(4-Methoxyphenyl)-2-phenyl-1-propanone (3p)

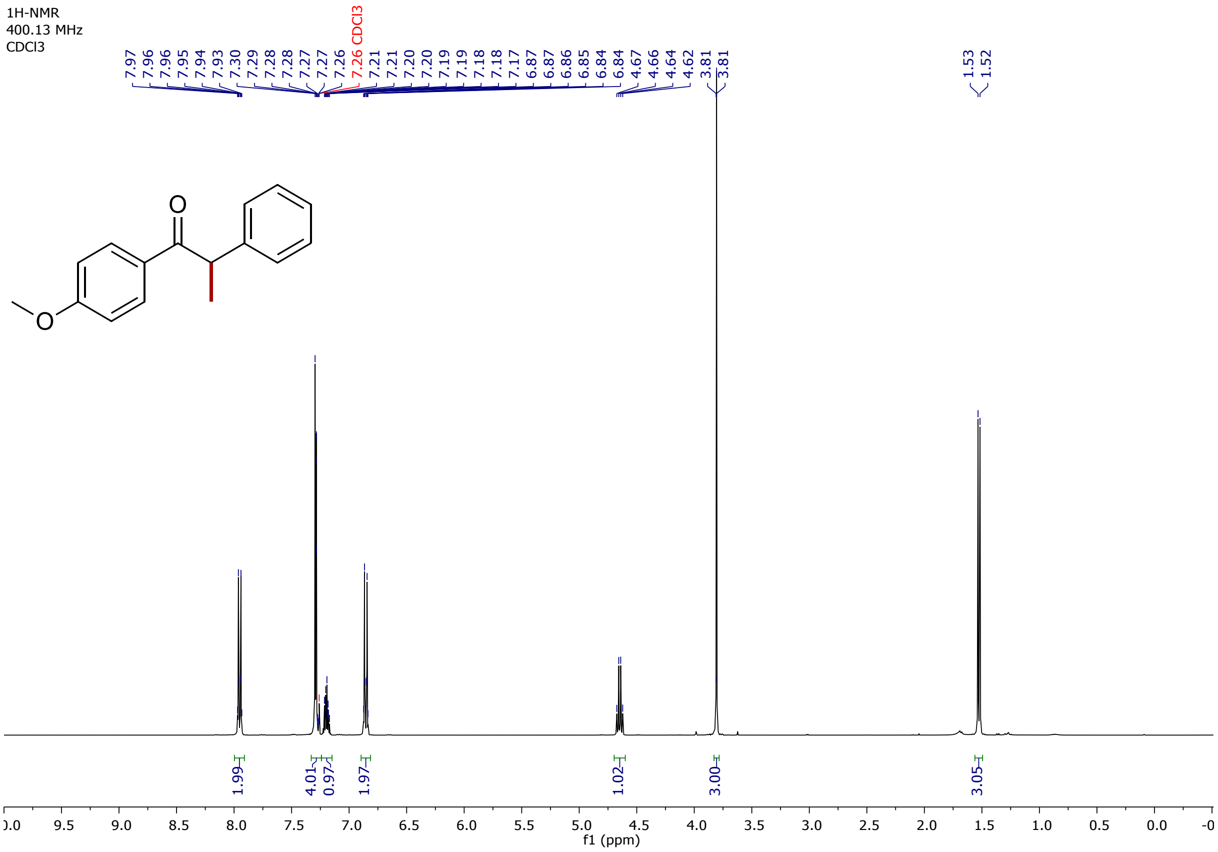


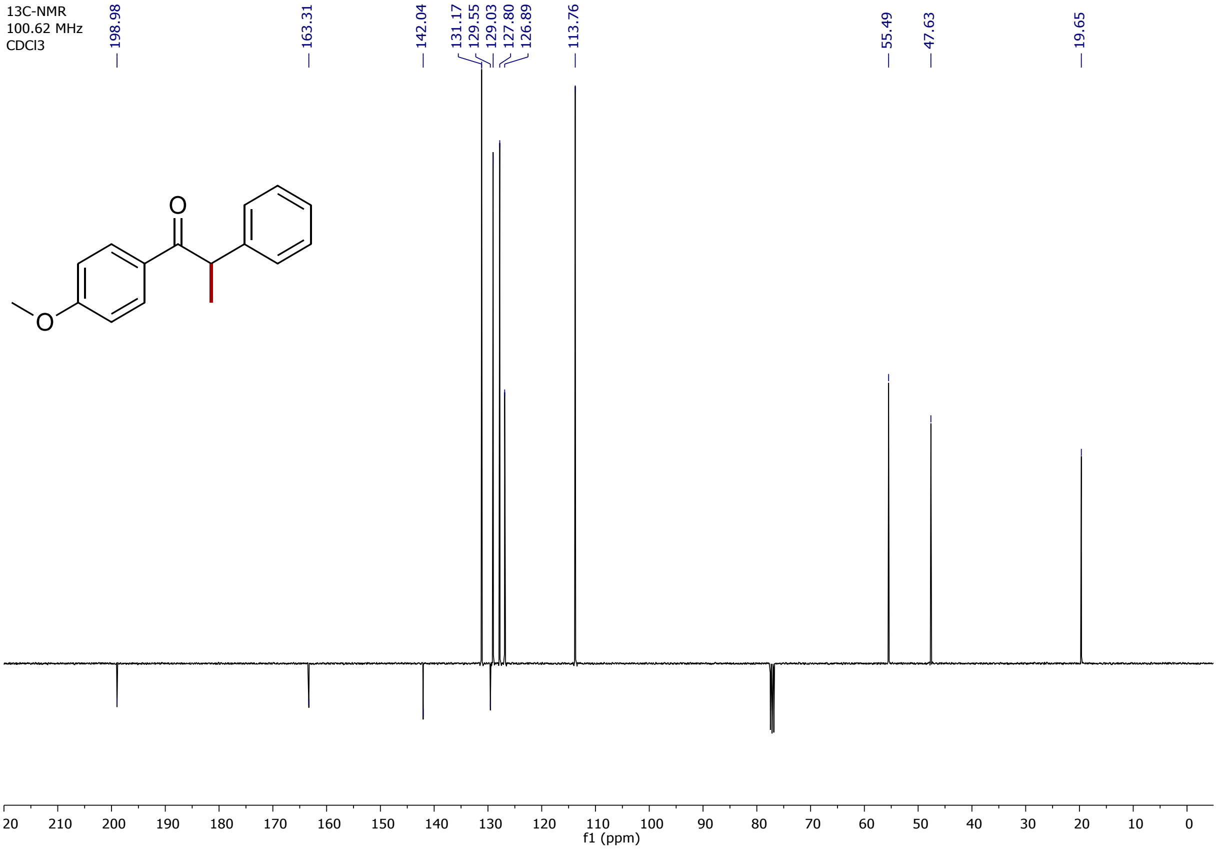

## 2-(4-Fluorophenyl)-1-phenyl-1-propanone (3q)

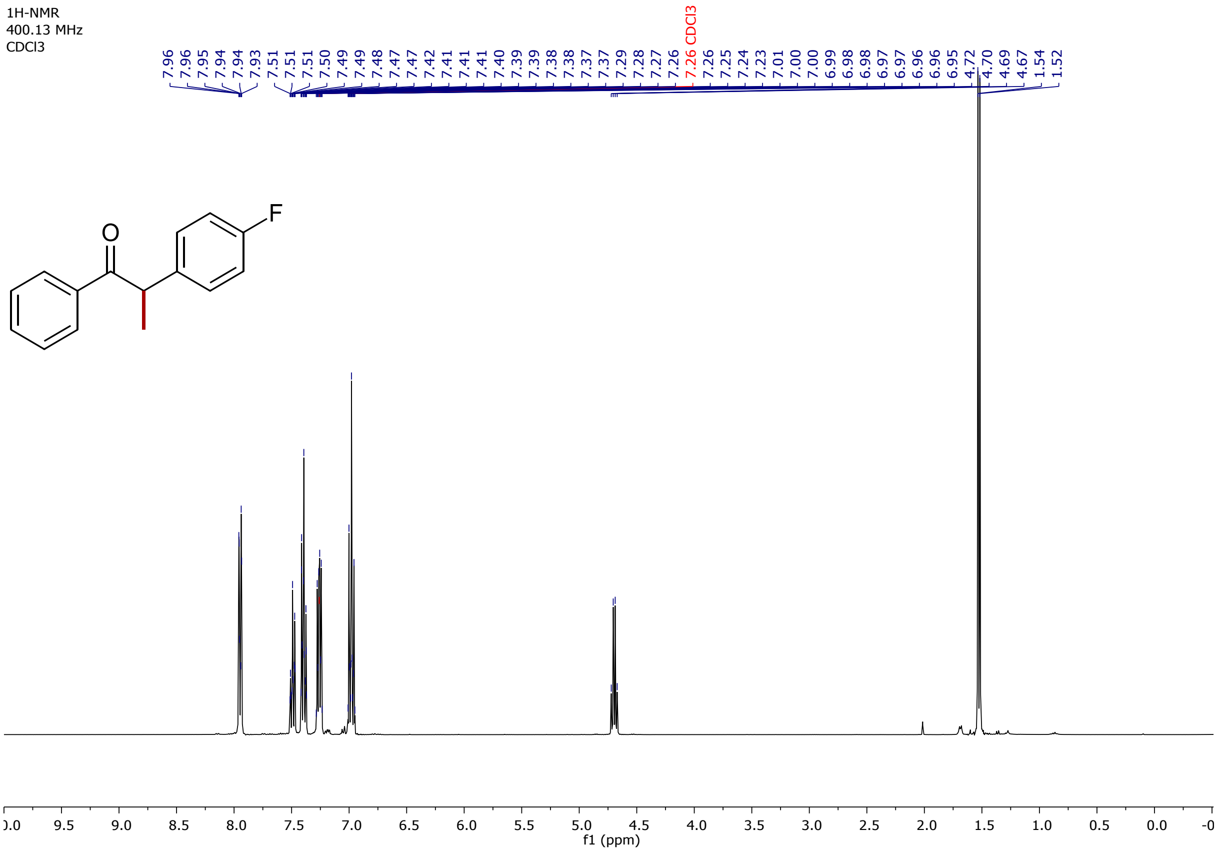


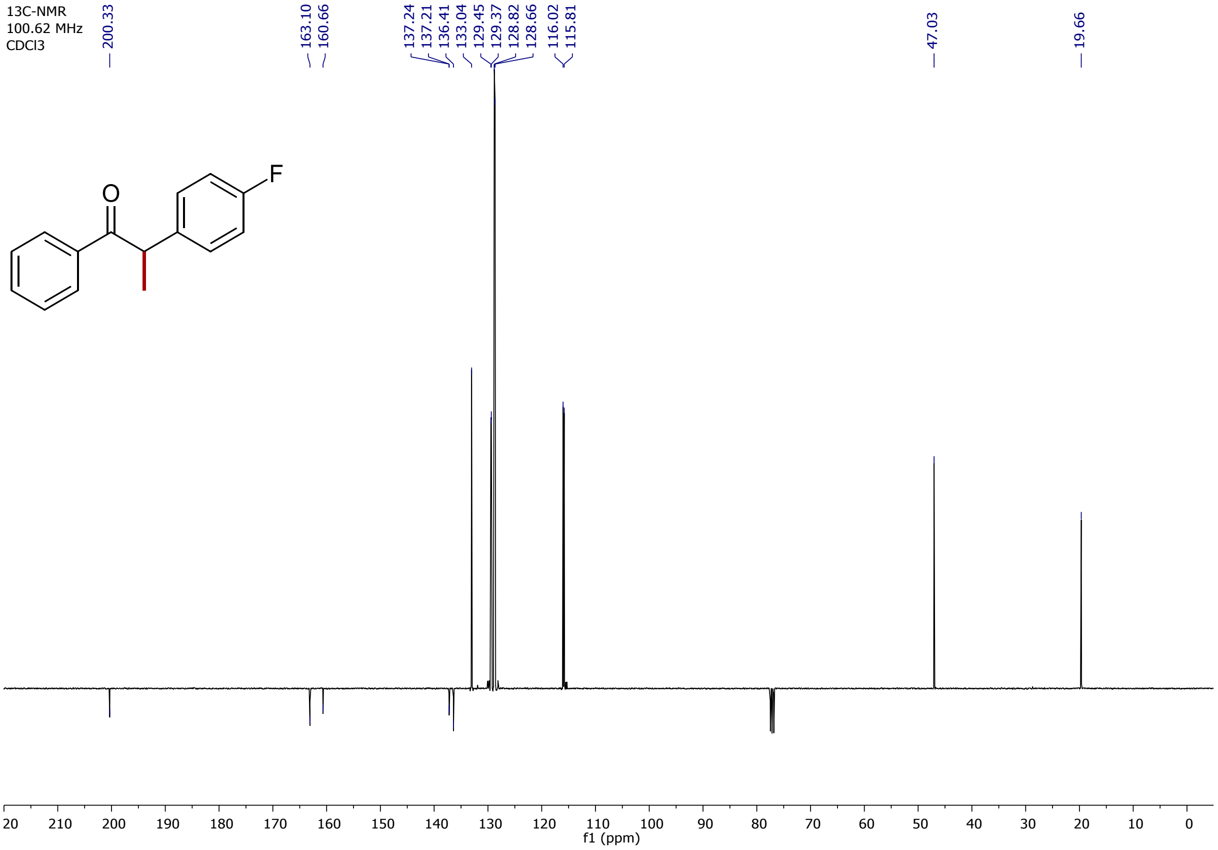

## 1-[1,1'-Biphenyl]-4-yl-2-phenyl-1-propanone (3r)

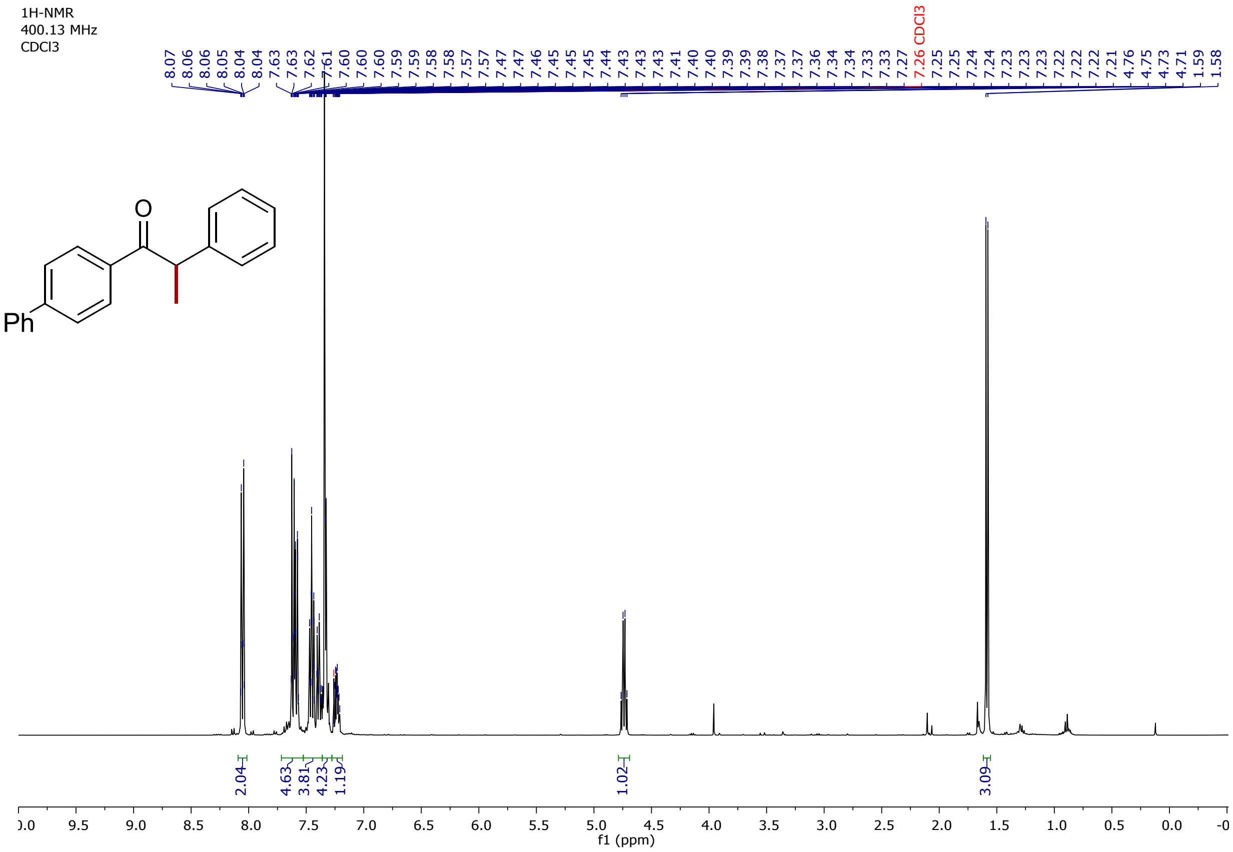


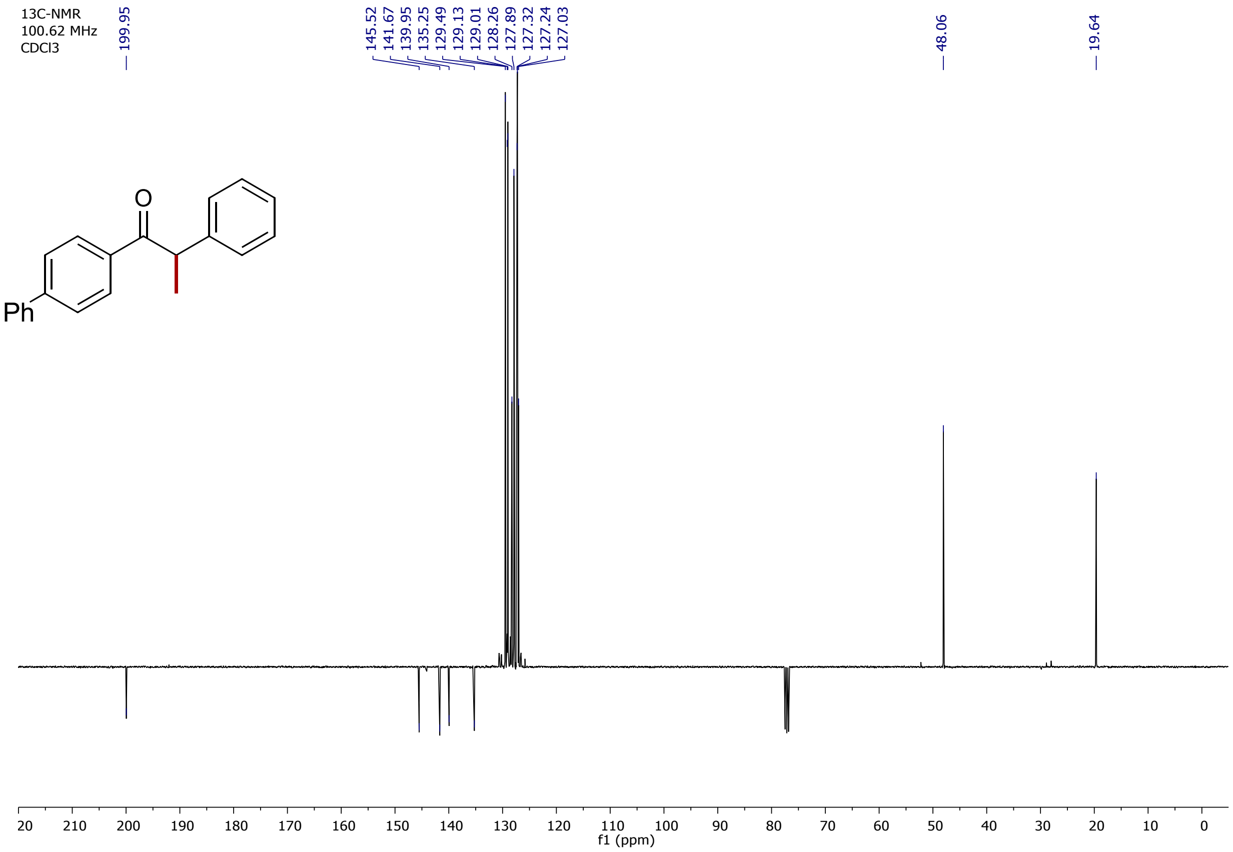

## 2-Methyl-1,3-diphenyl-1,3-propanedione (3s)

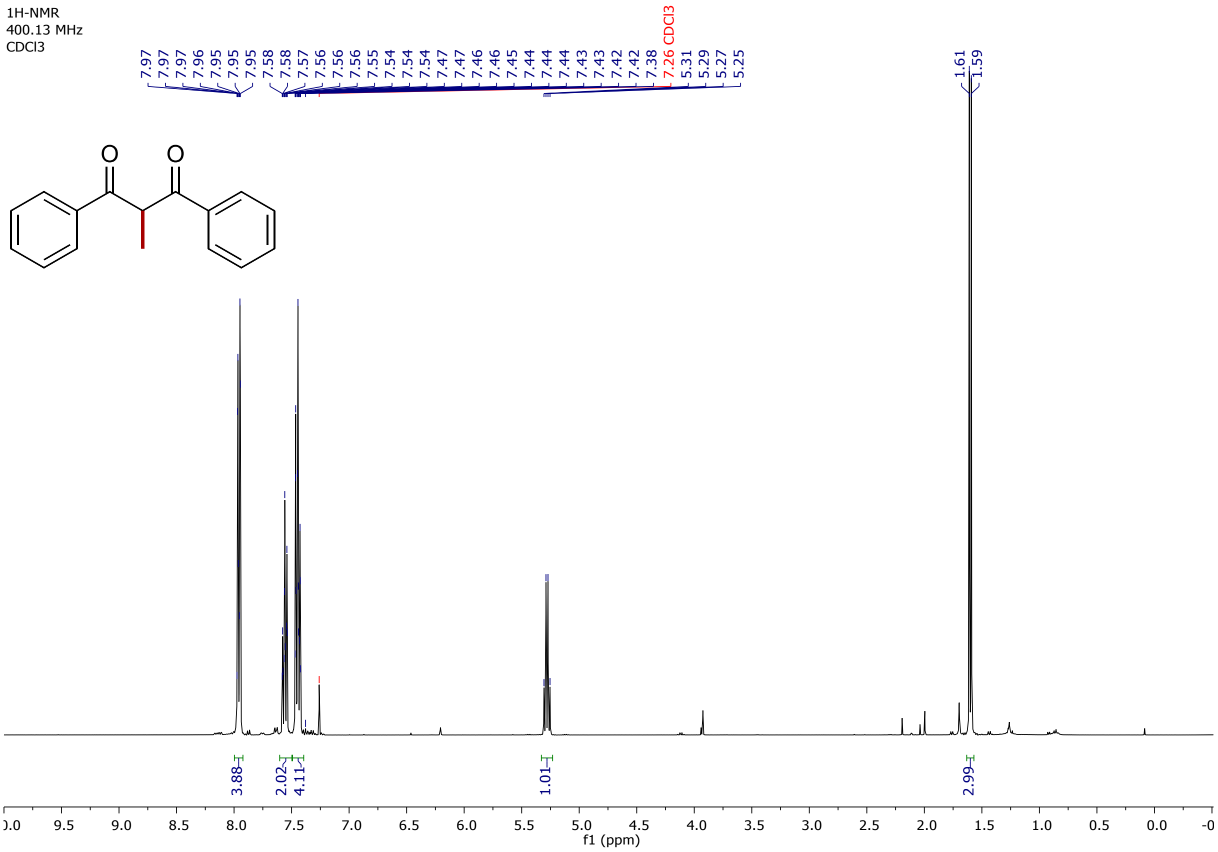


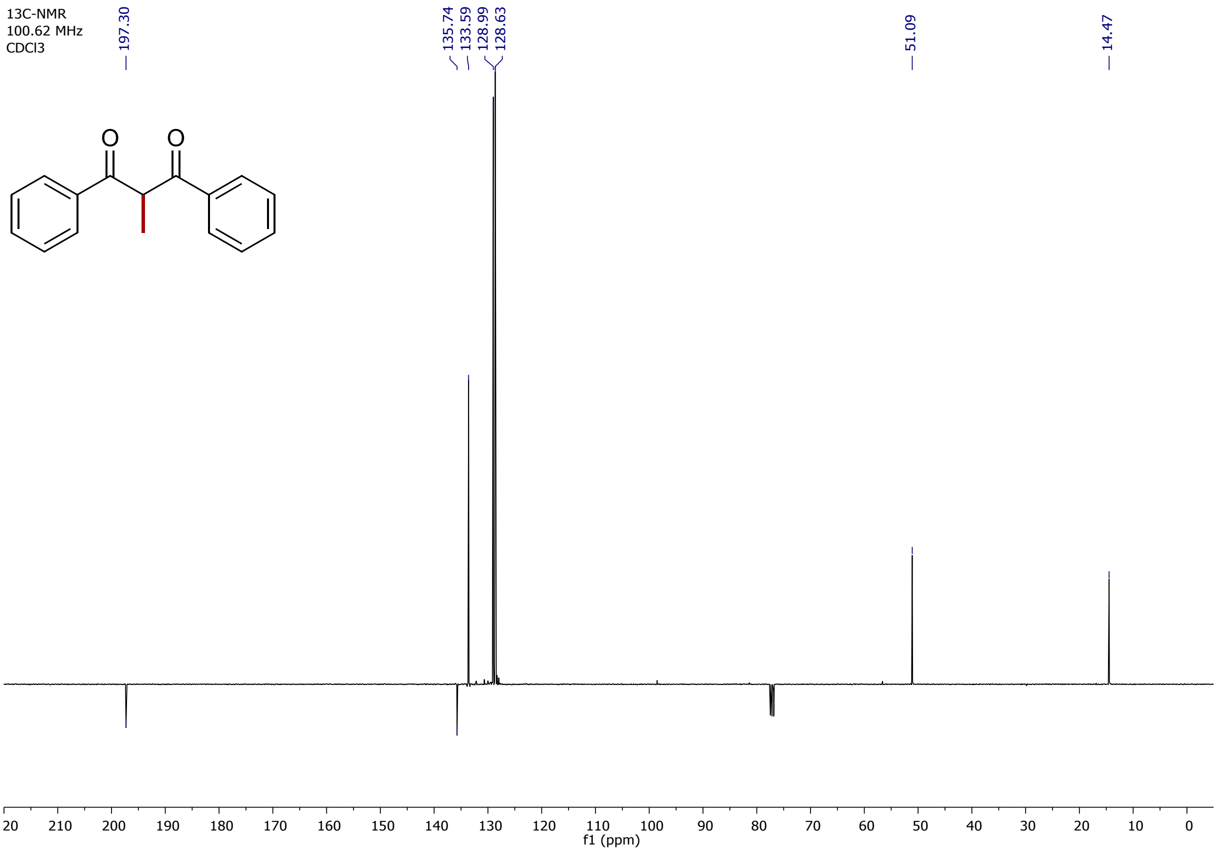

# 2-Methyl-4-phenylcyclohexanone (3t)

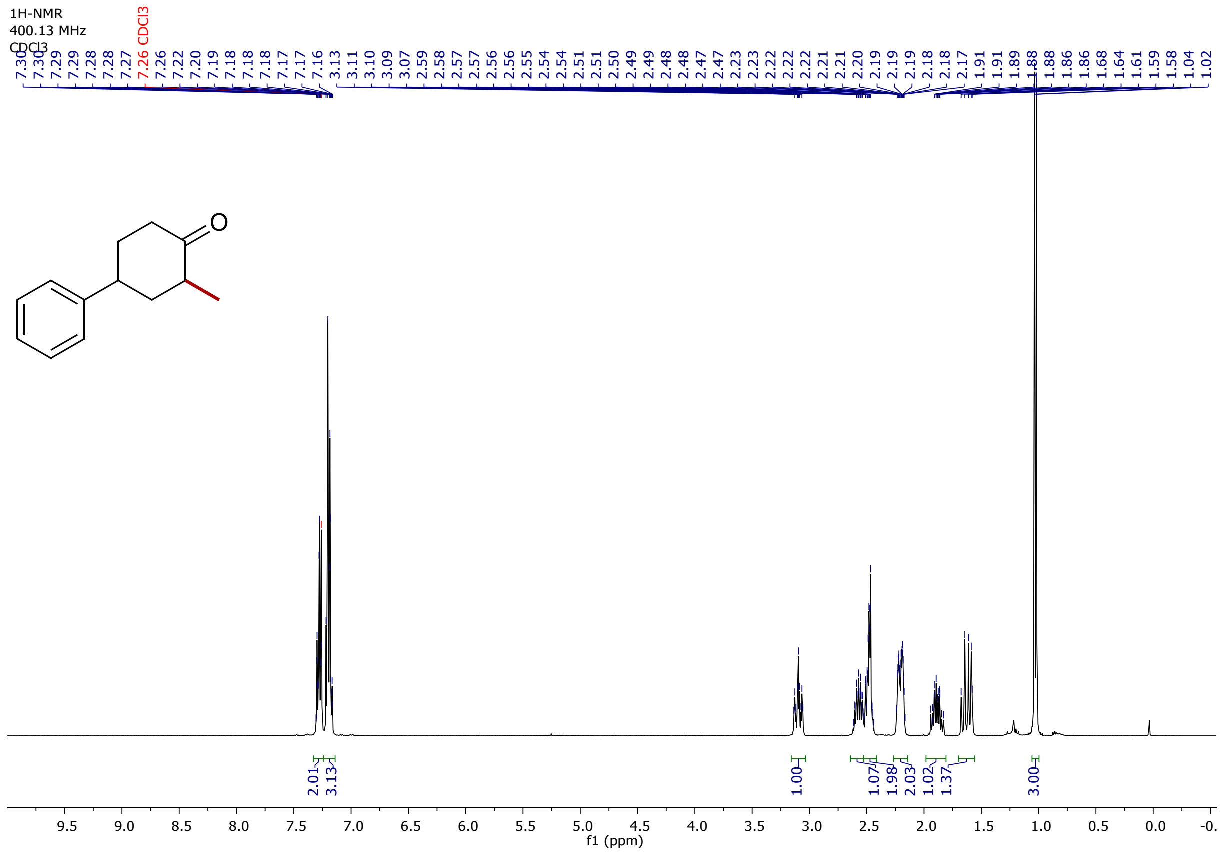


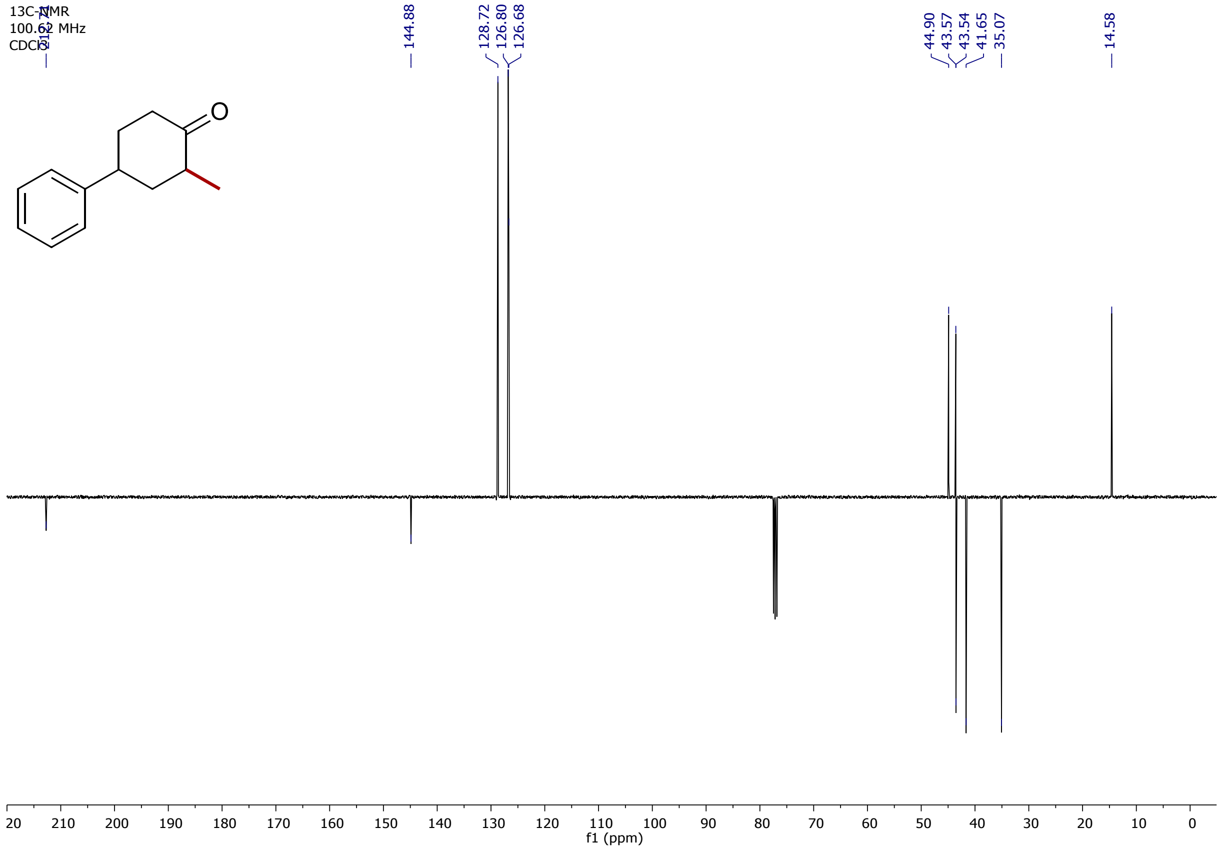

## 5-Methyl-2-phenyl-3-hexanone (3u)

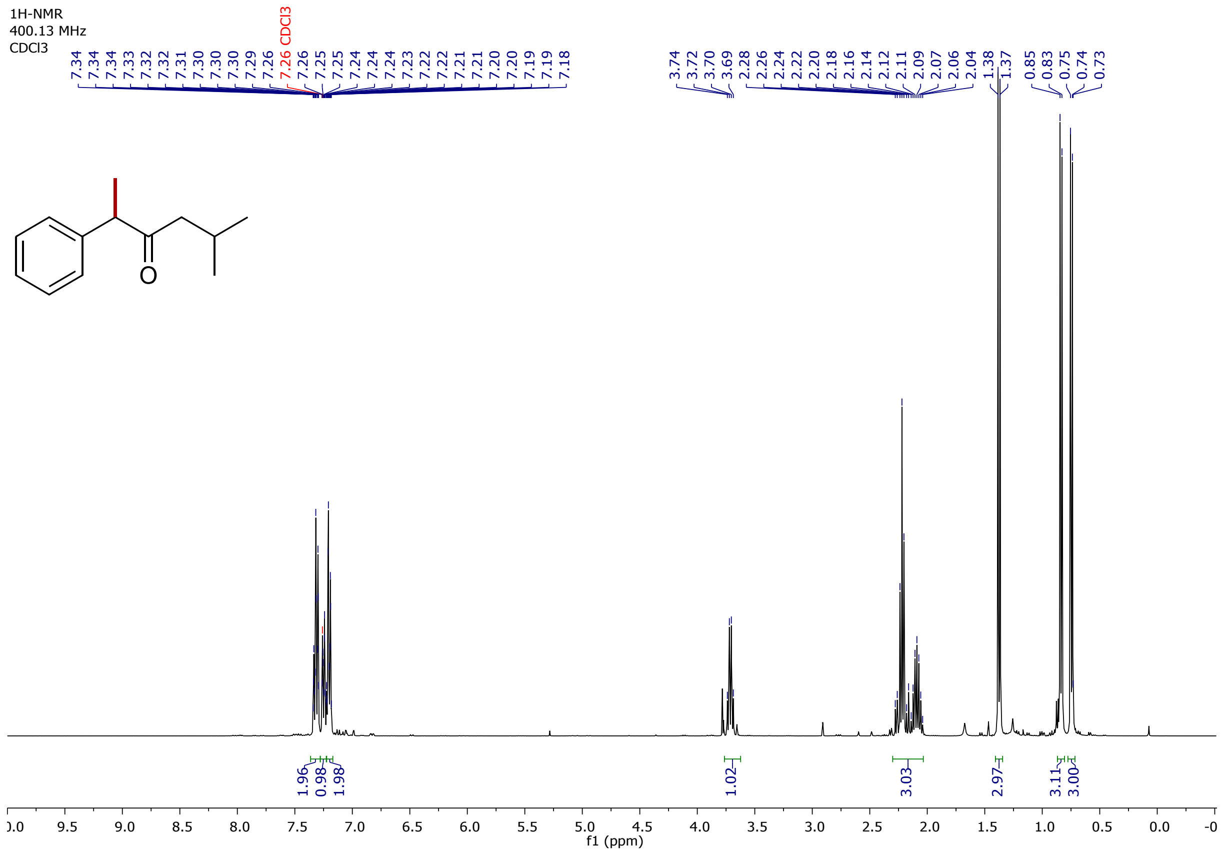


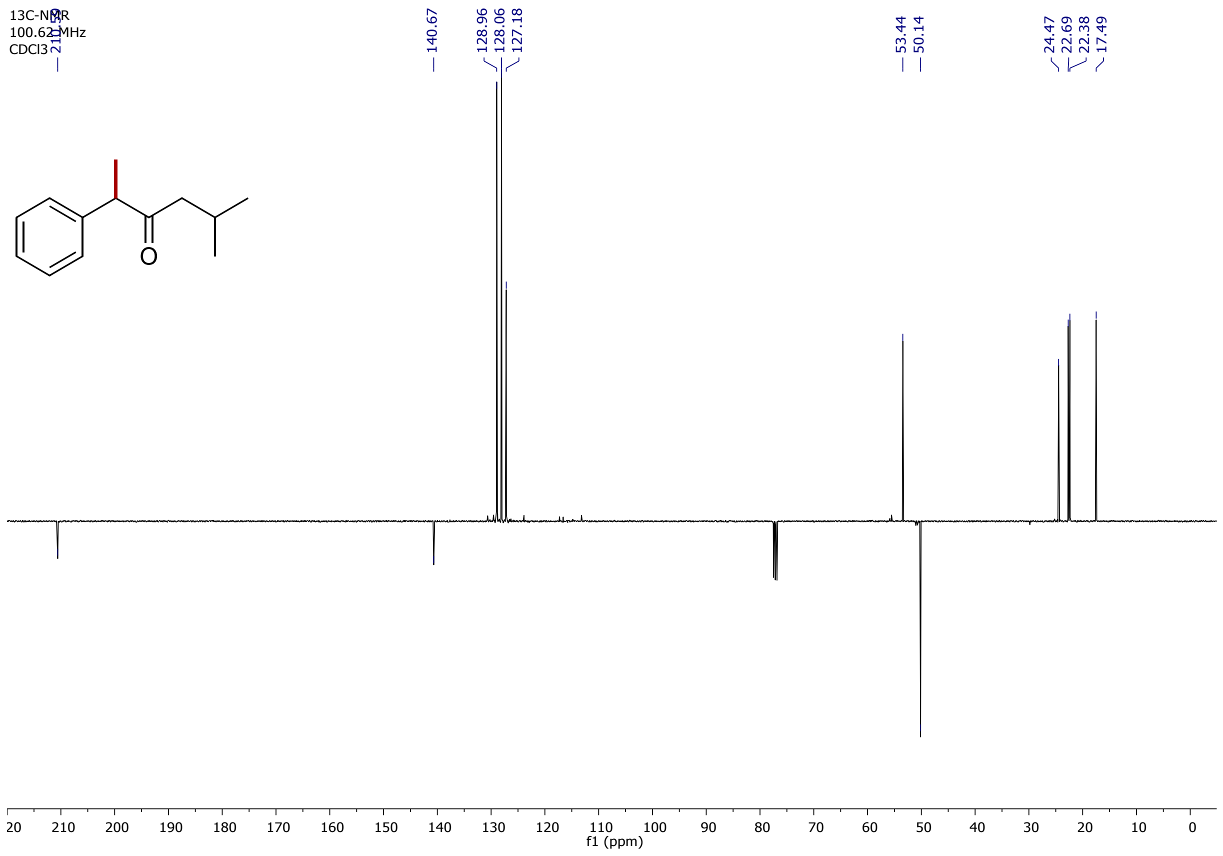

## Methyl 4-(1-methyl-2-oxo-2-phenylethyl)benzoate (3v)

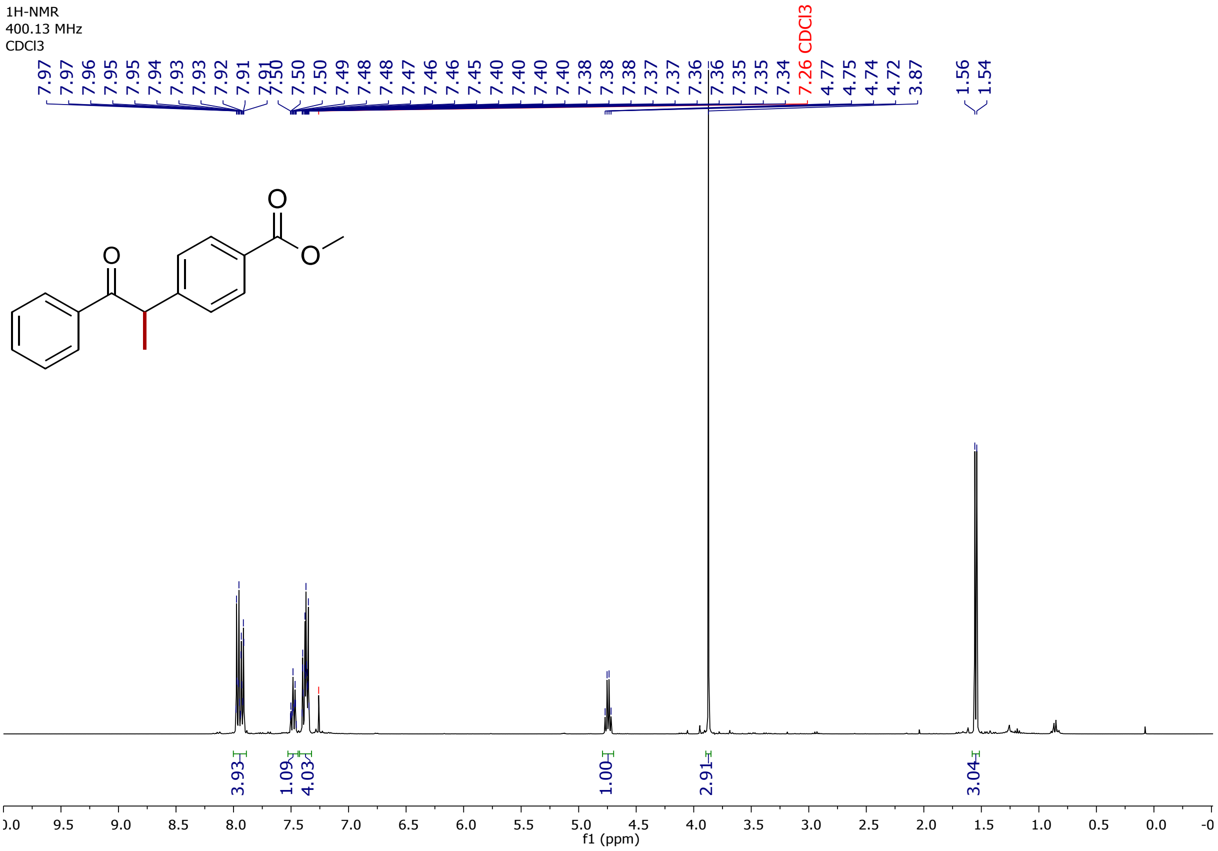


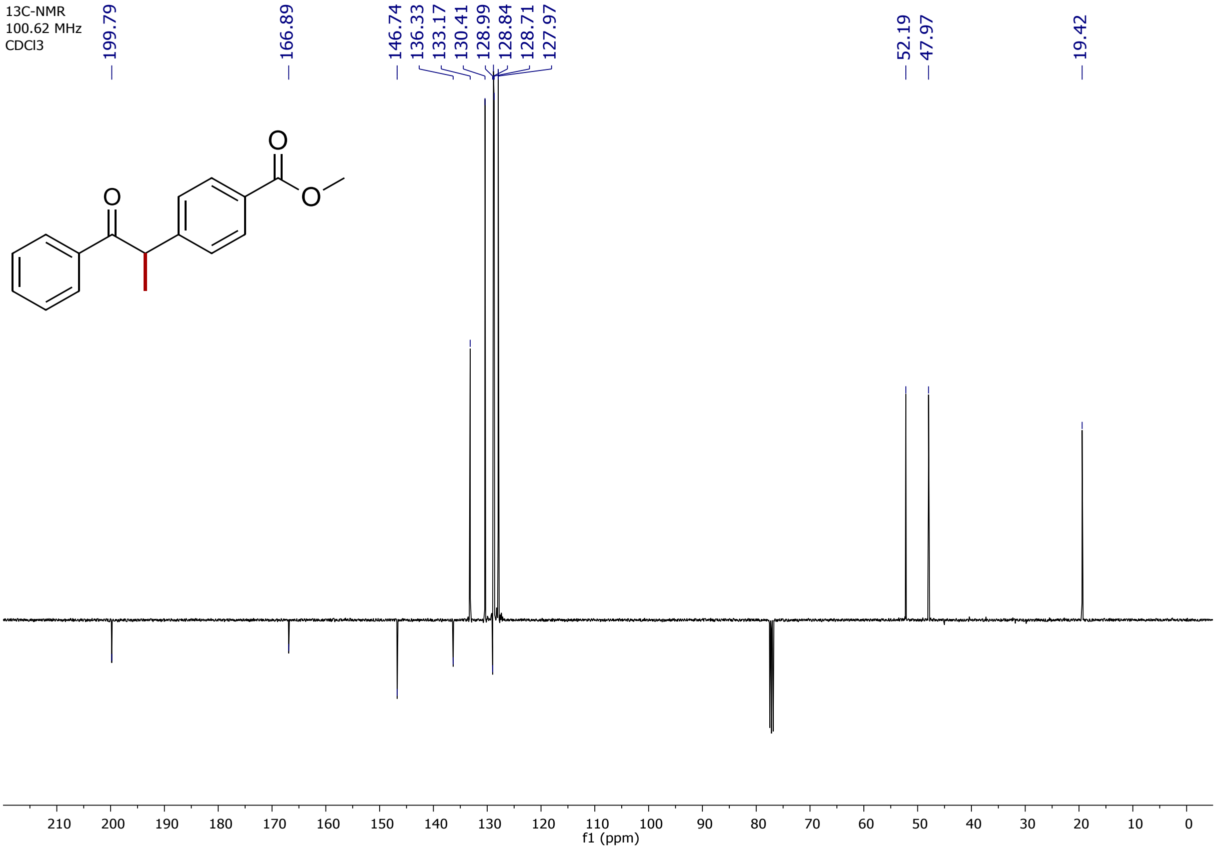

## Ethyl 4-(1-methyl-2-oxo-2-phenylethyl)benzoate (3w)

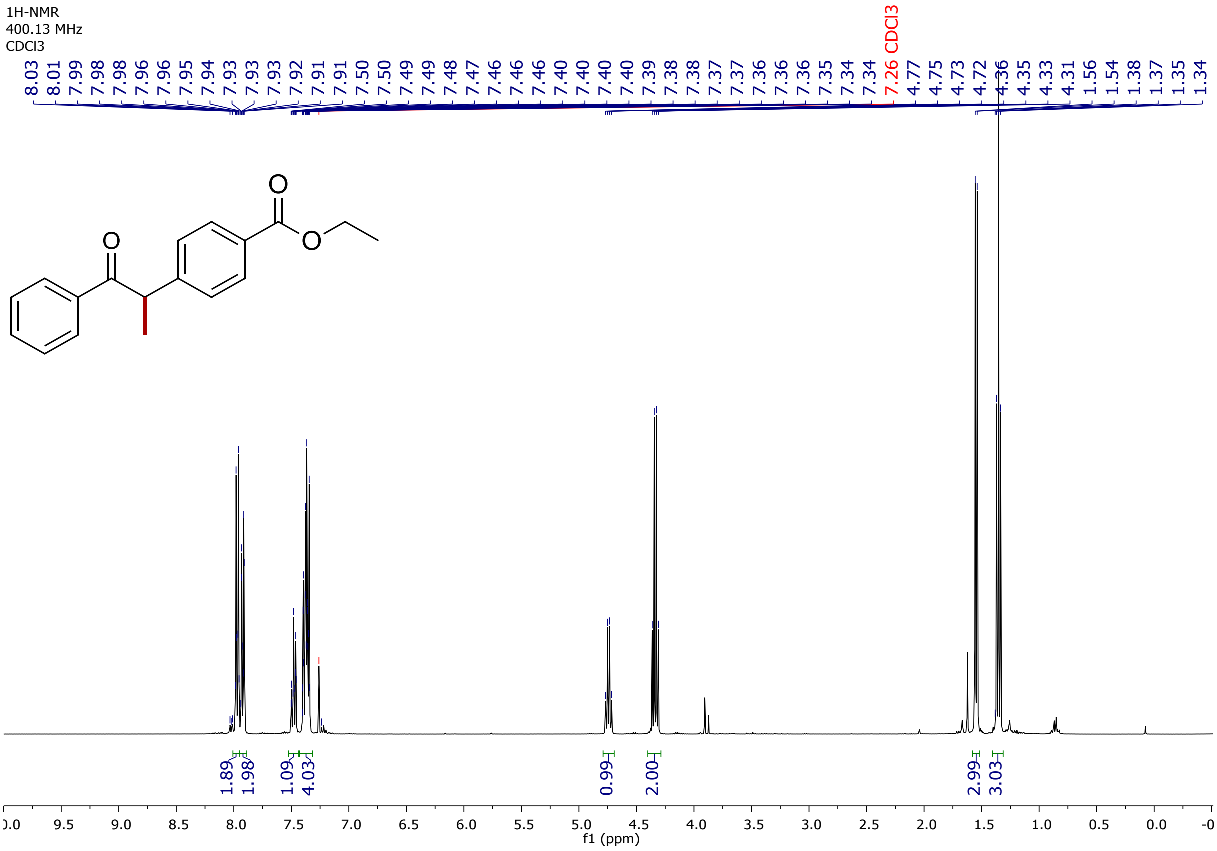


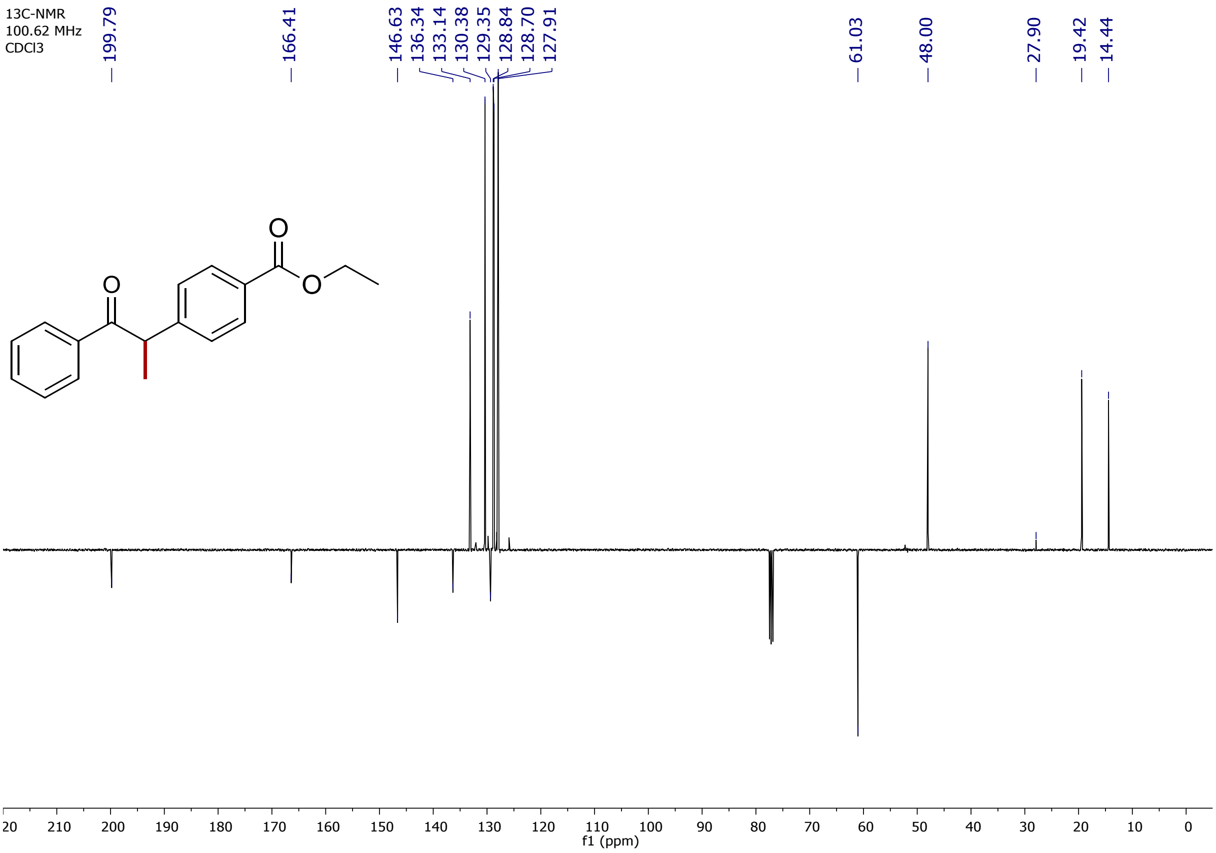

## 1-(4-Methoxyphenyl)-2-phenylethanone (3x)

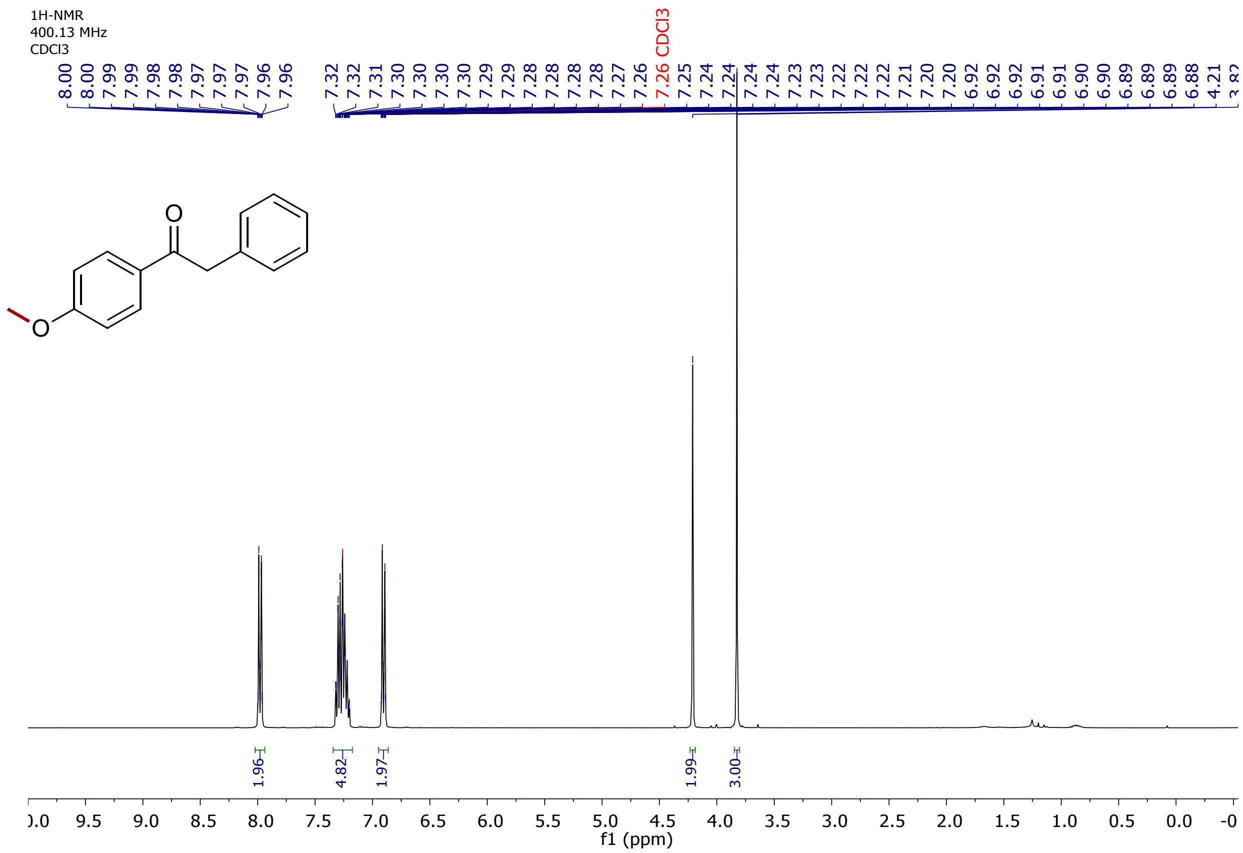


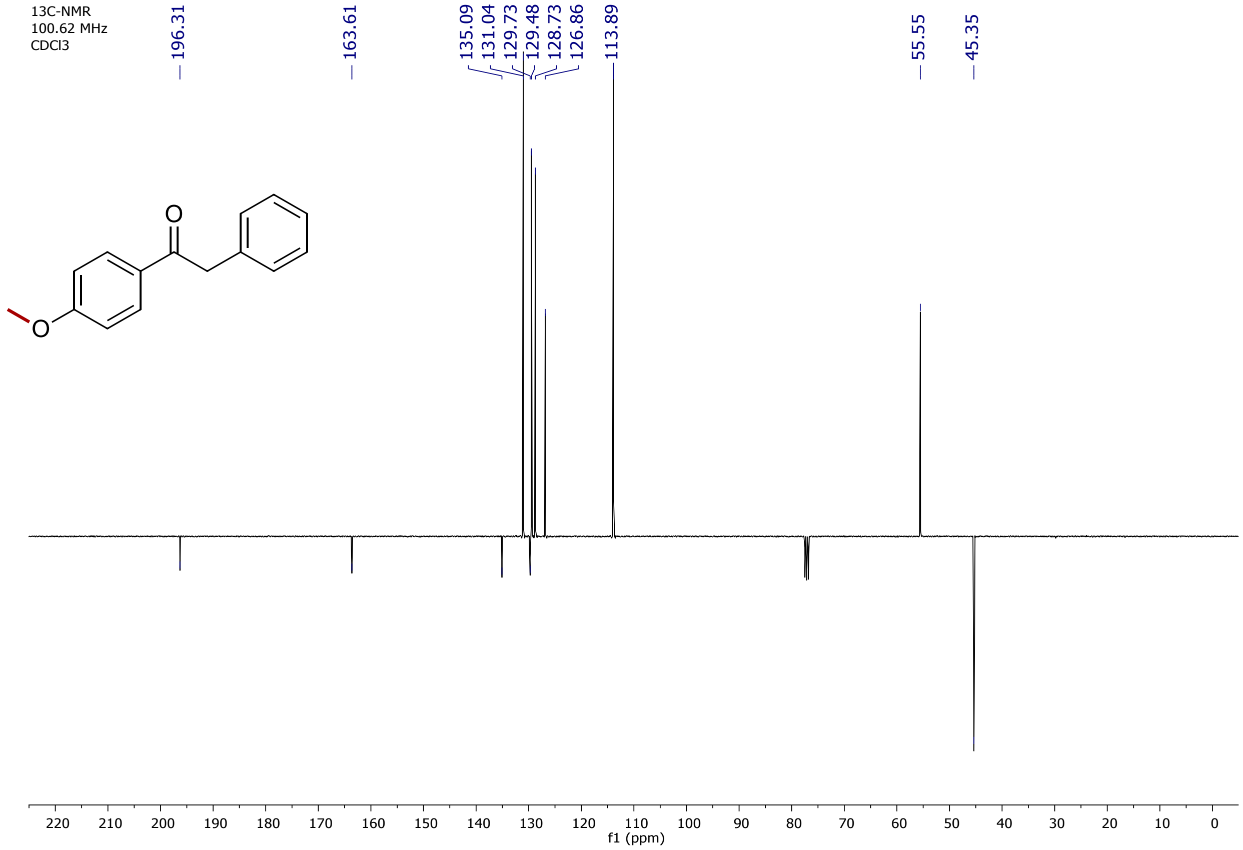

## Methyl 4-(biphenyl-4-yl)-4-oxobutanoate (3y)

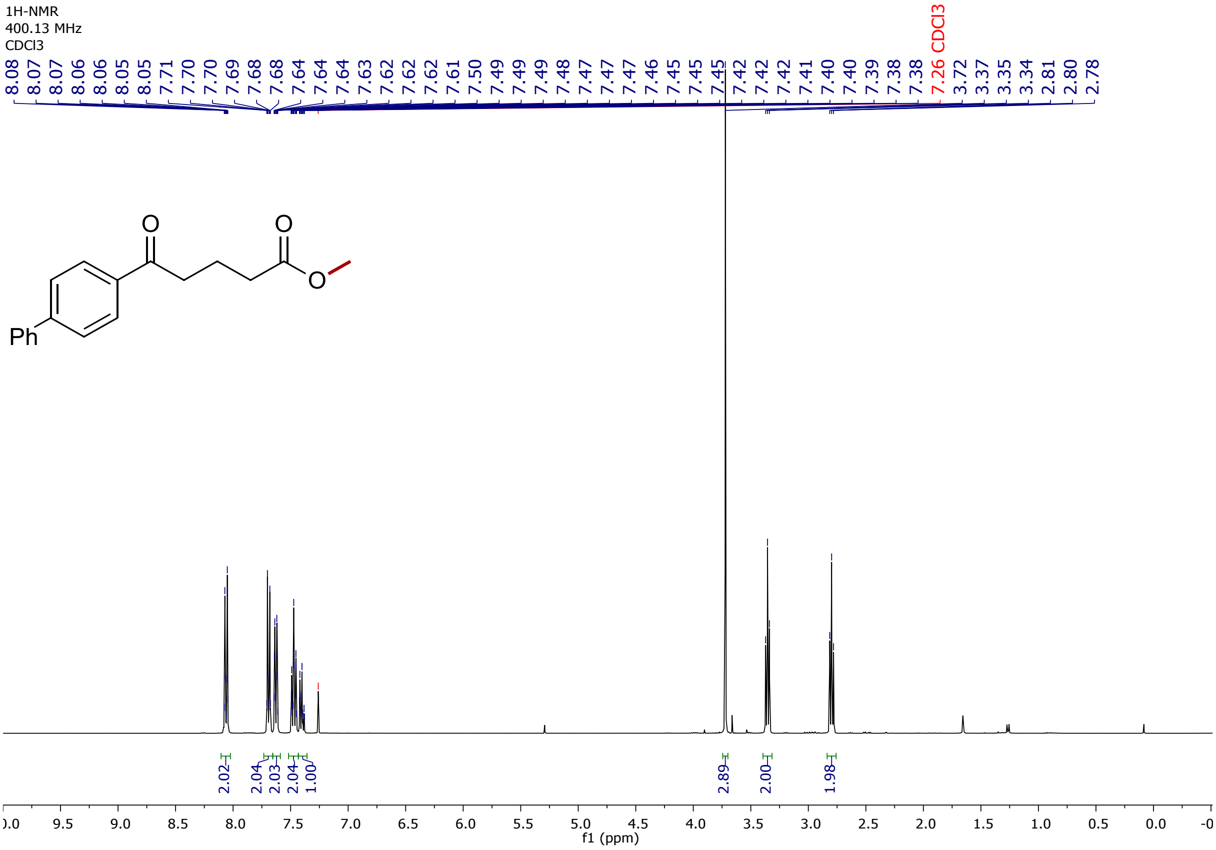


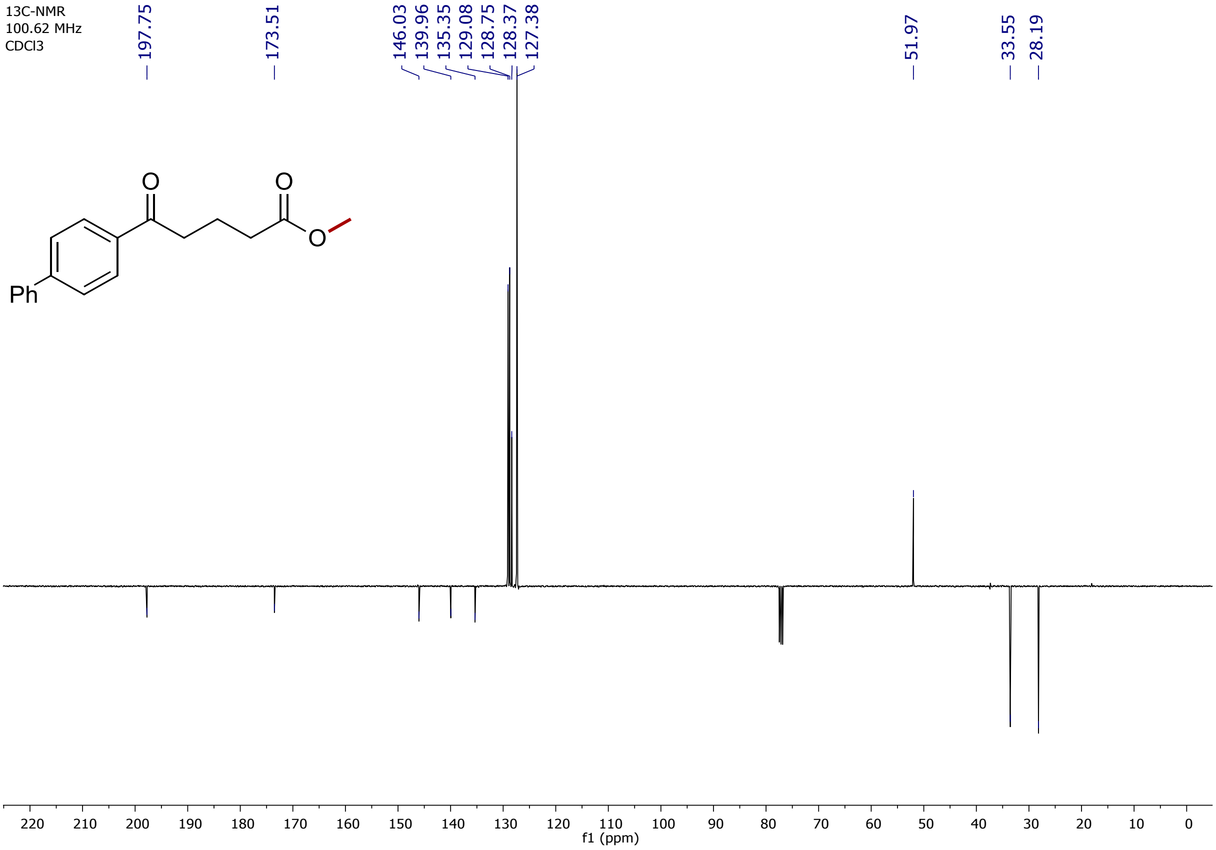

## Methyl 3-methyl-4-oxo-4-(4-phenylphenyl)butanoate (3z)

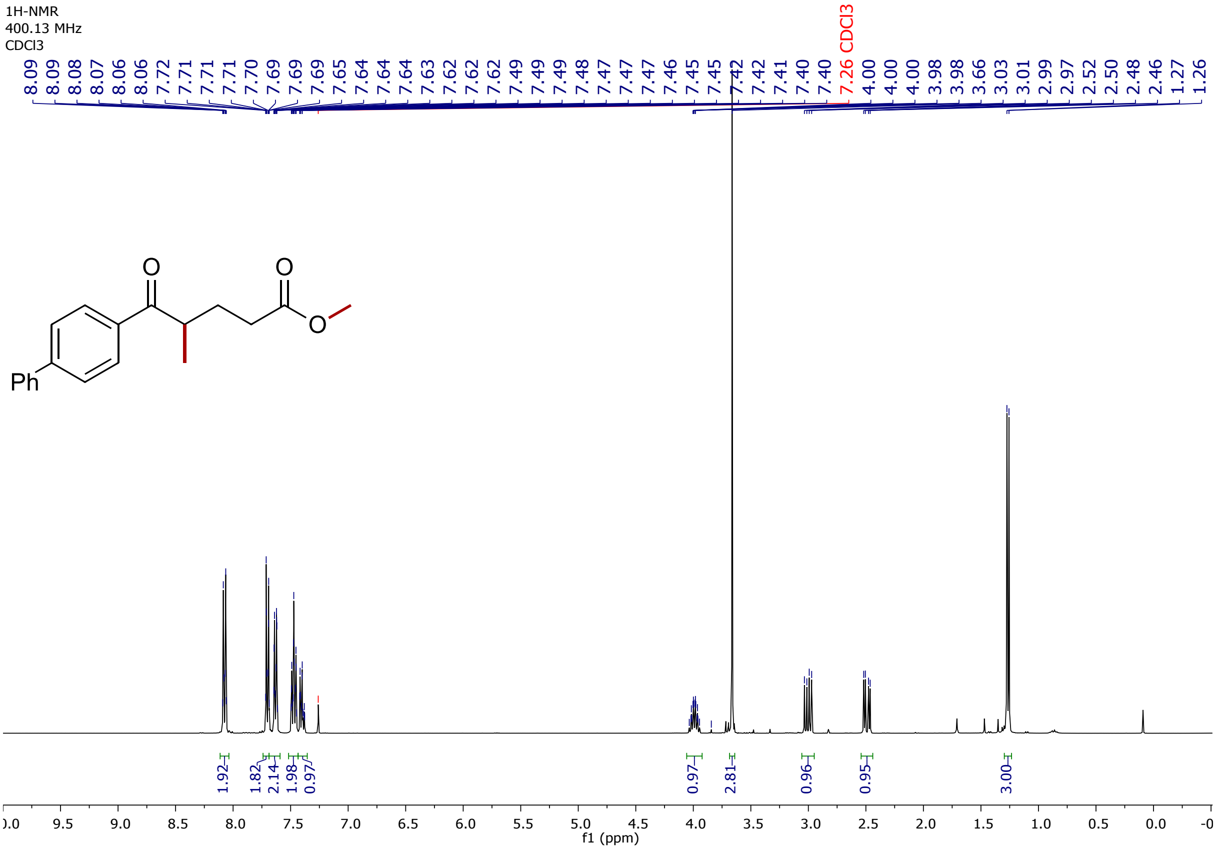


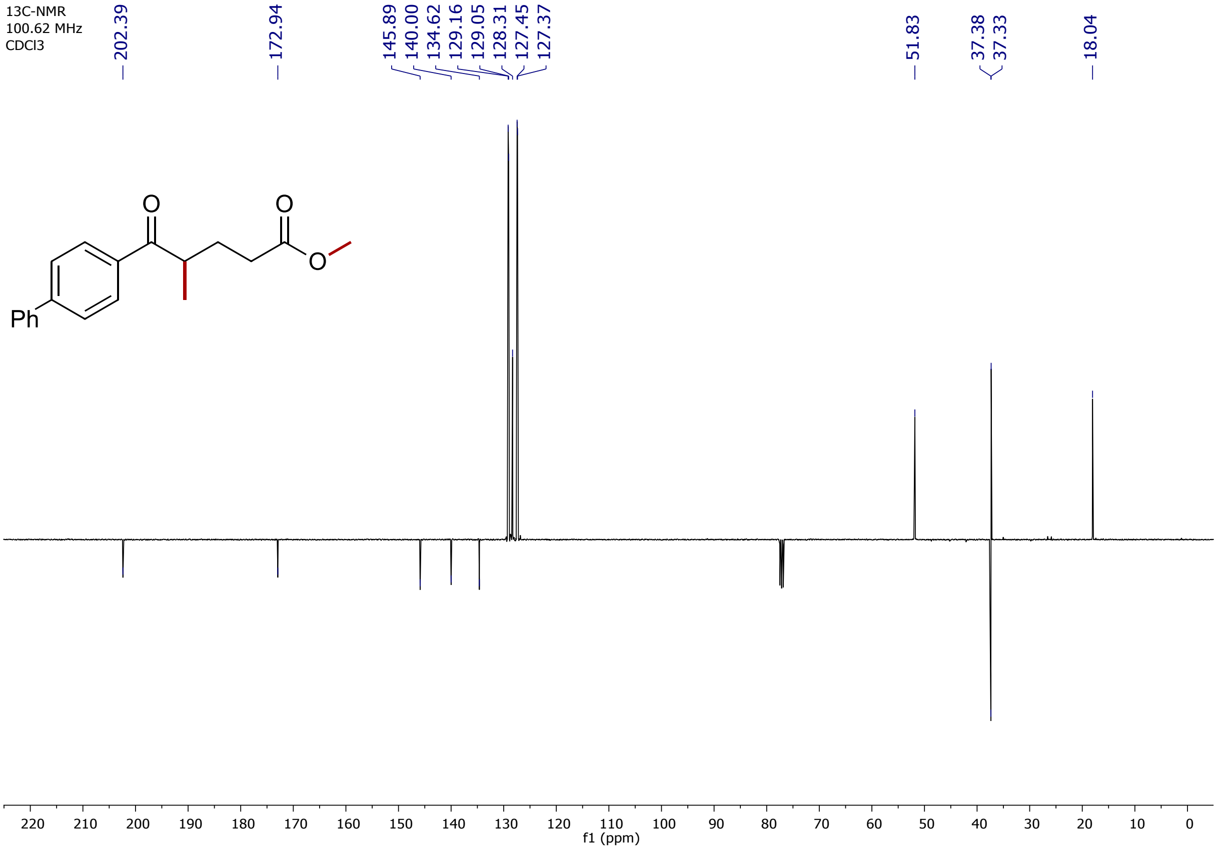

# 1-(4-Fluorophenyl)-2-phenyl-1-butanone (4a)

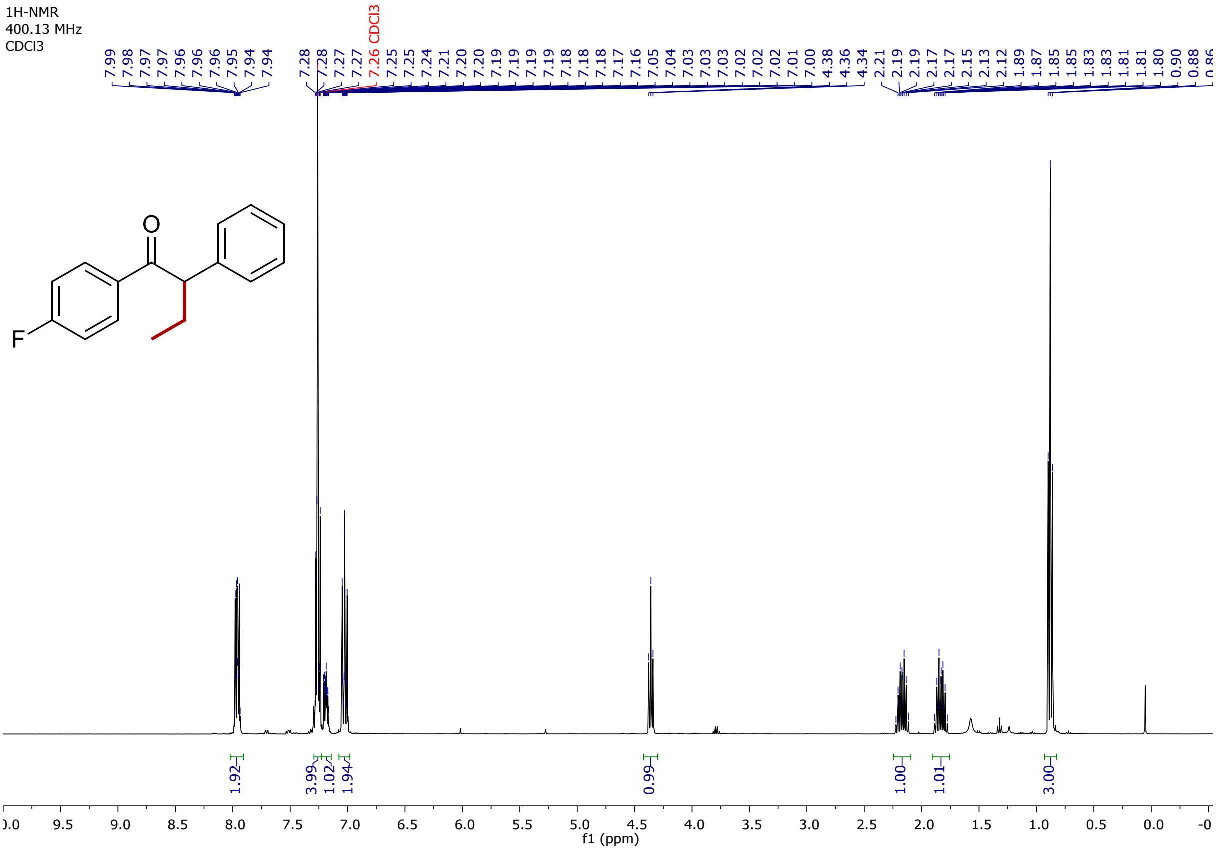


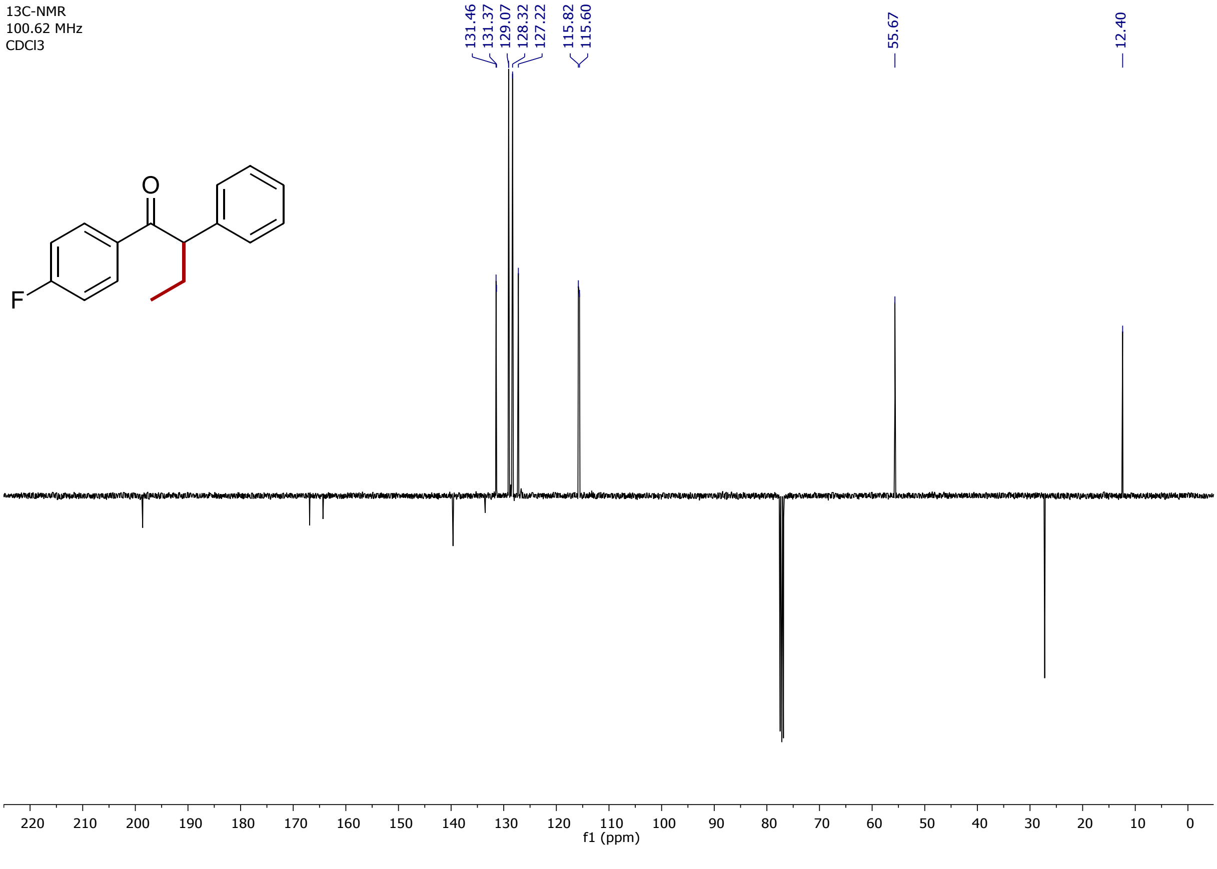

## 1,2-Bis(4-methoxyphenyl)-1-butanone (4b)

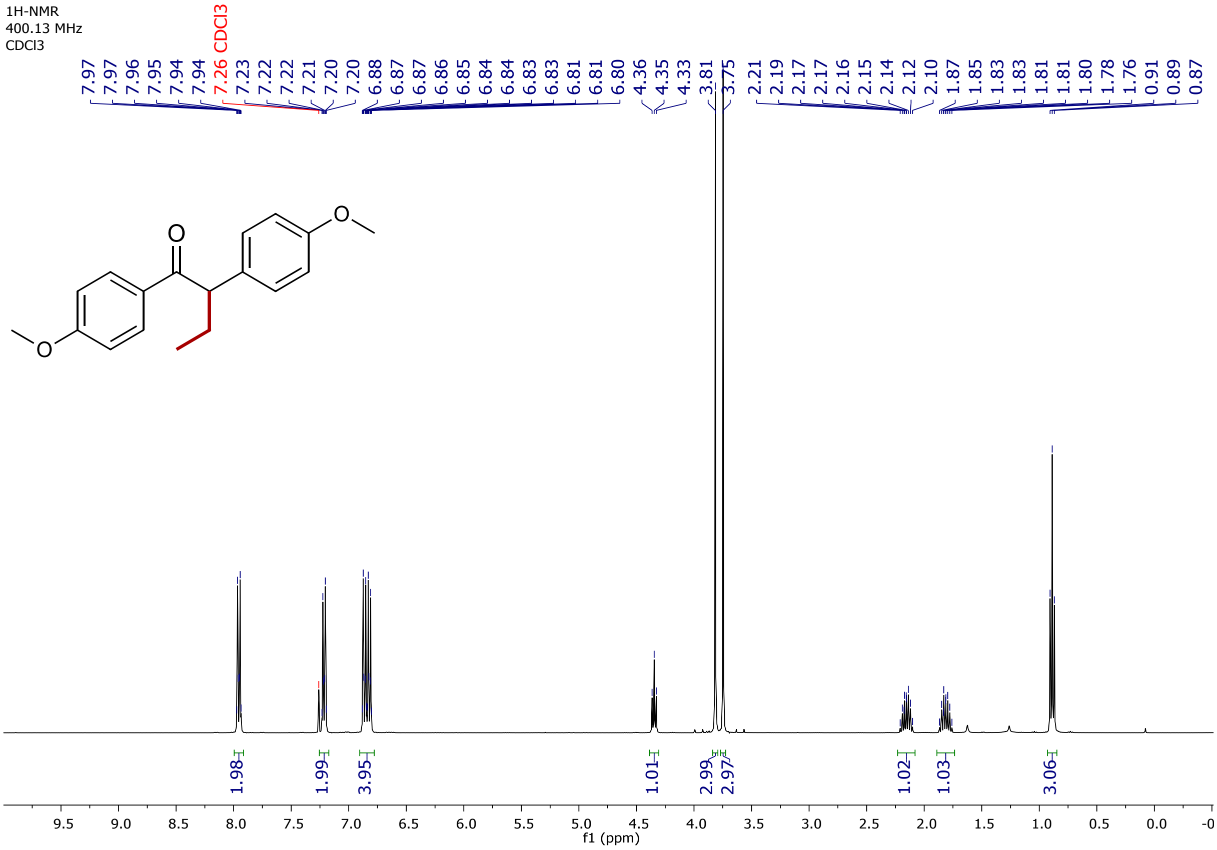


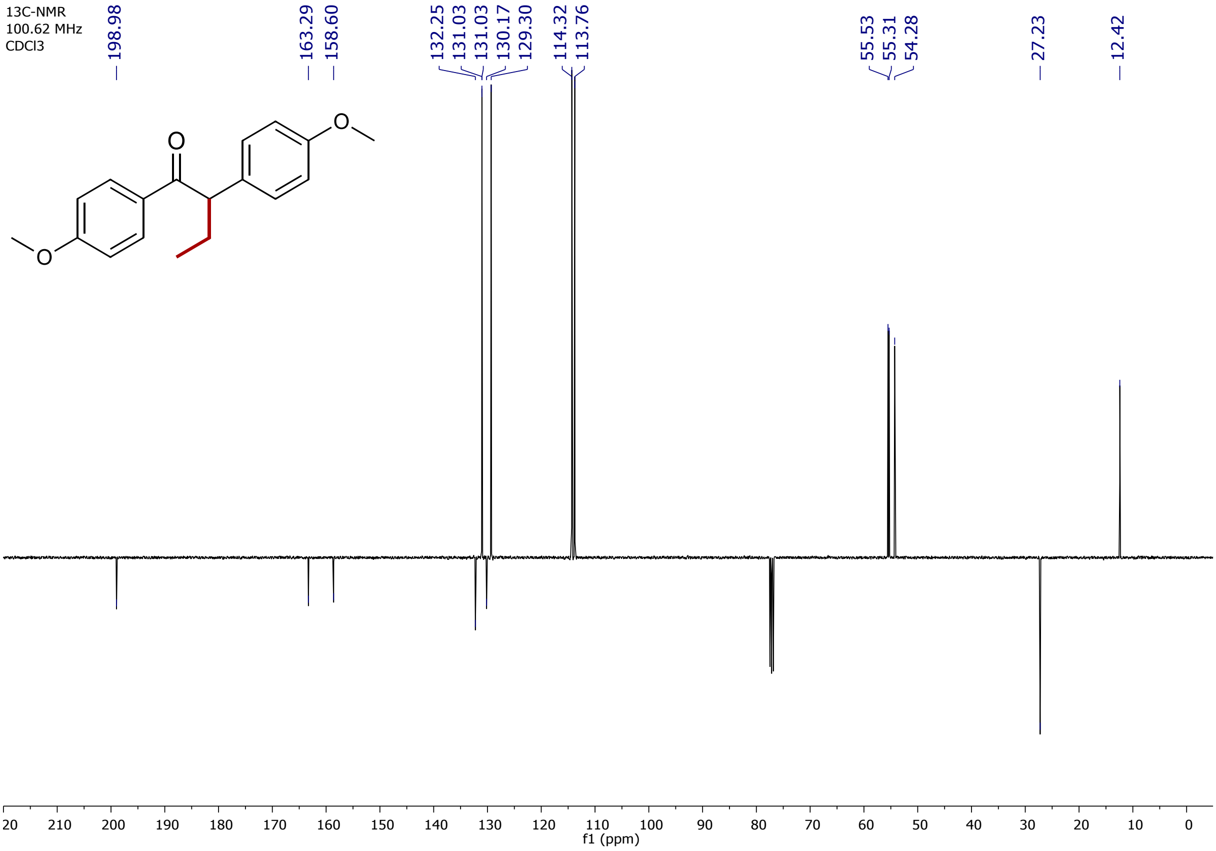

## 2-Methyl-5-phenyl-4-heptanone (4c)

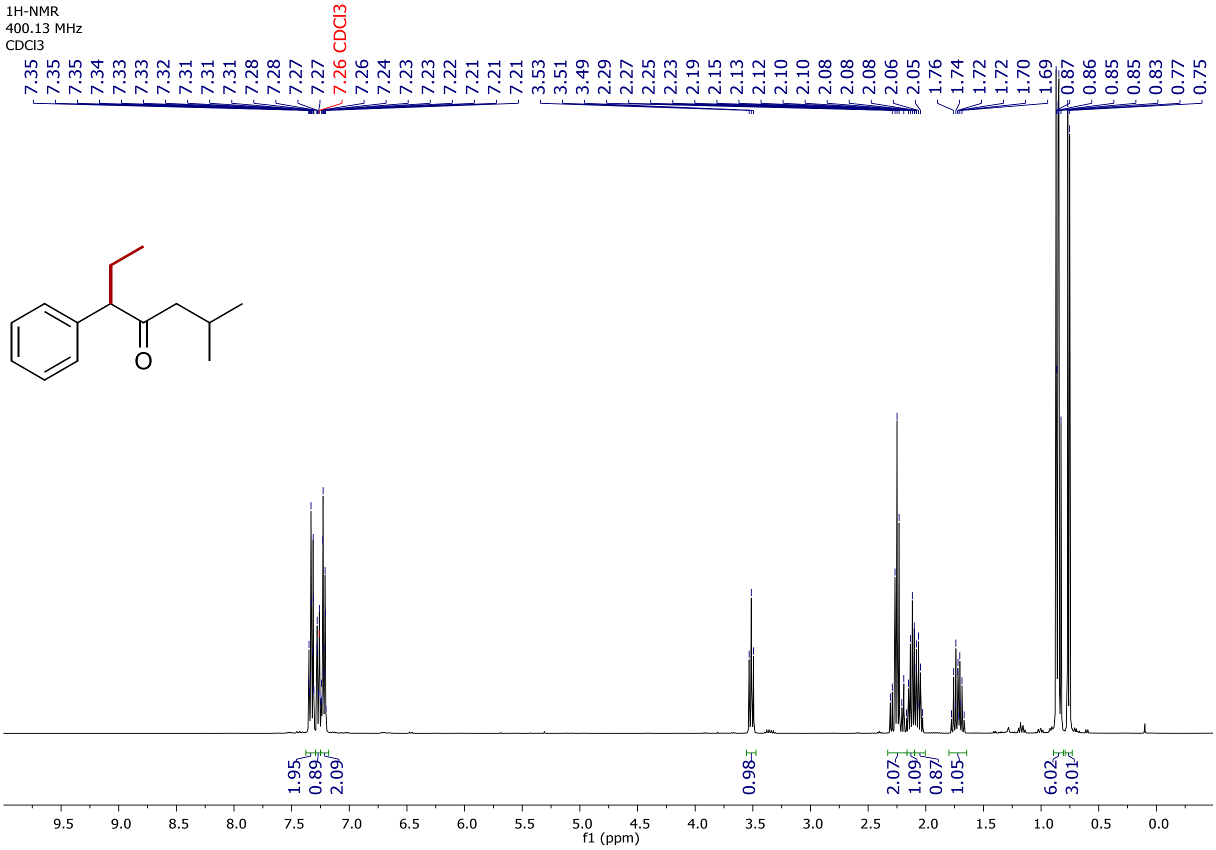


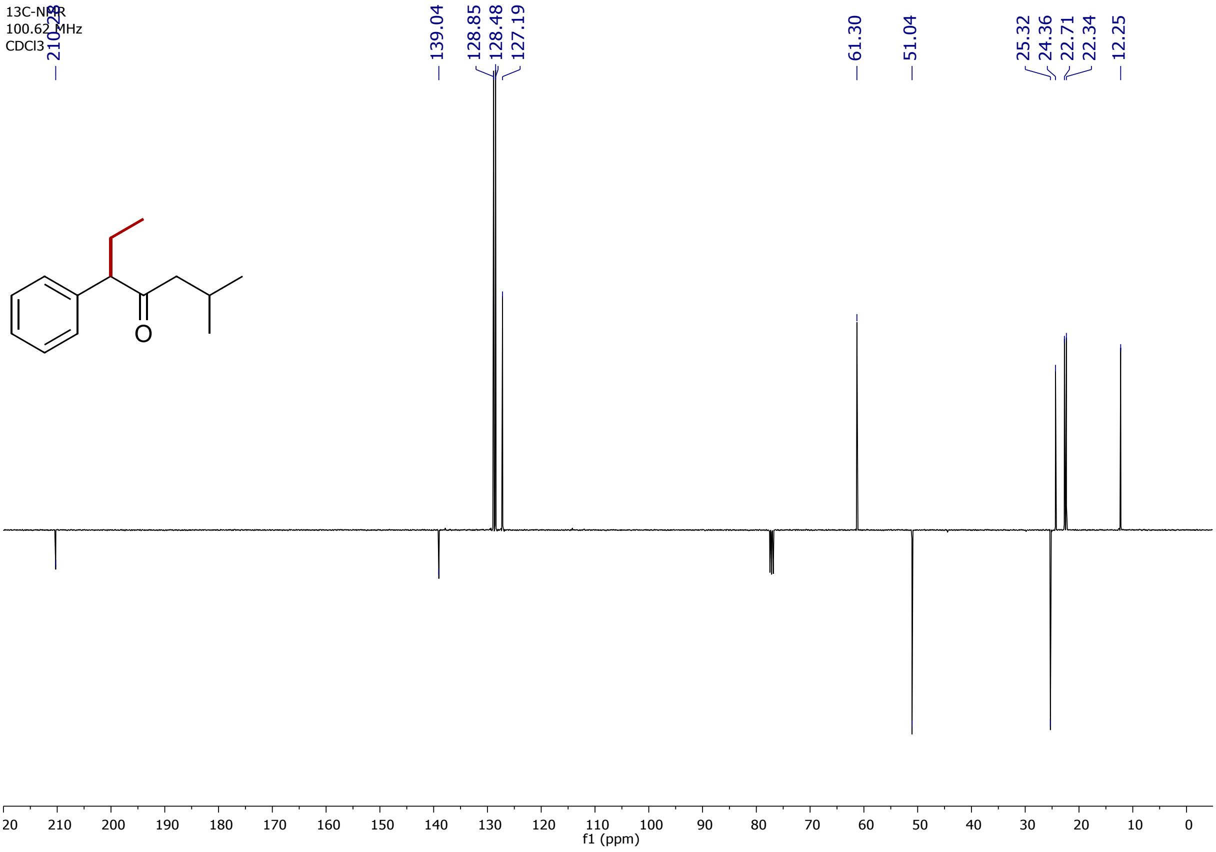

## 1-(4-Fluorophenyl)-2,3-diphenylpropan-1-one (5a)

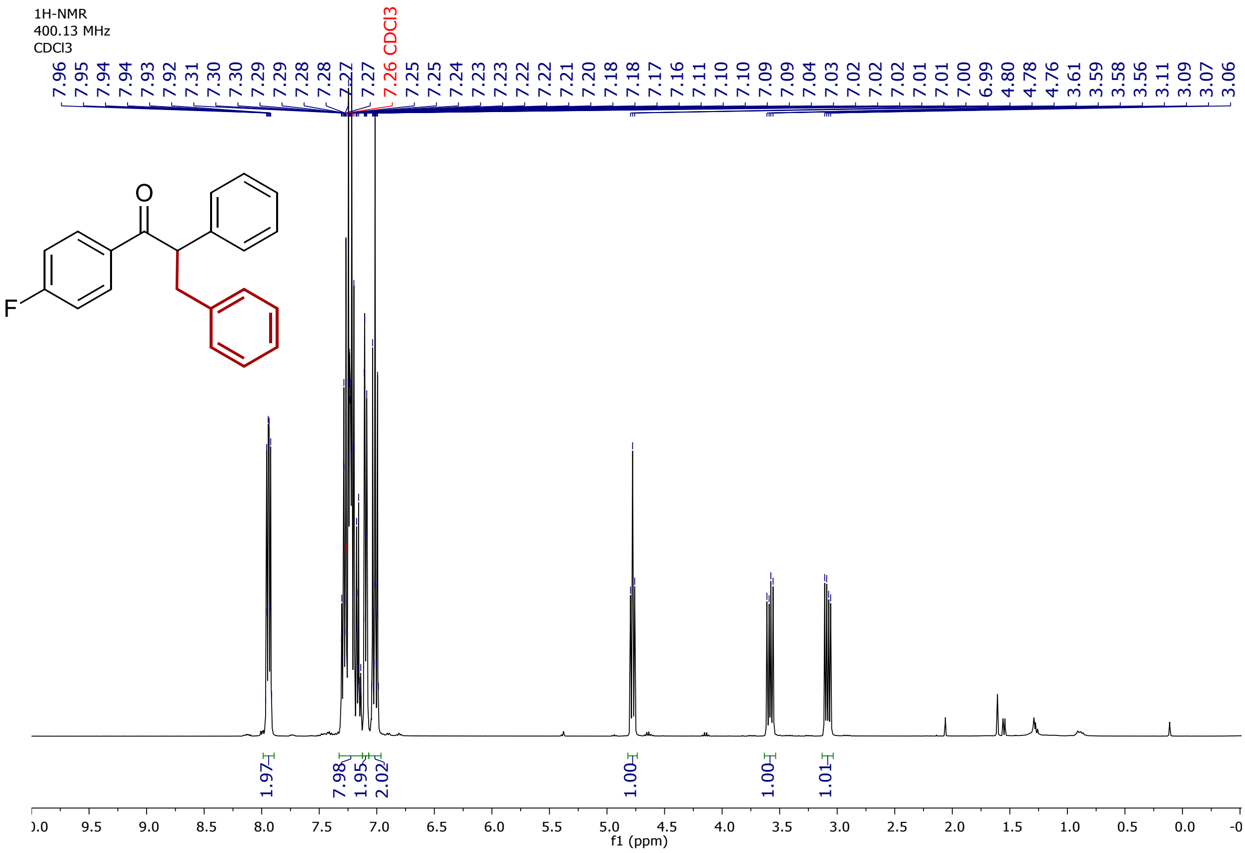


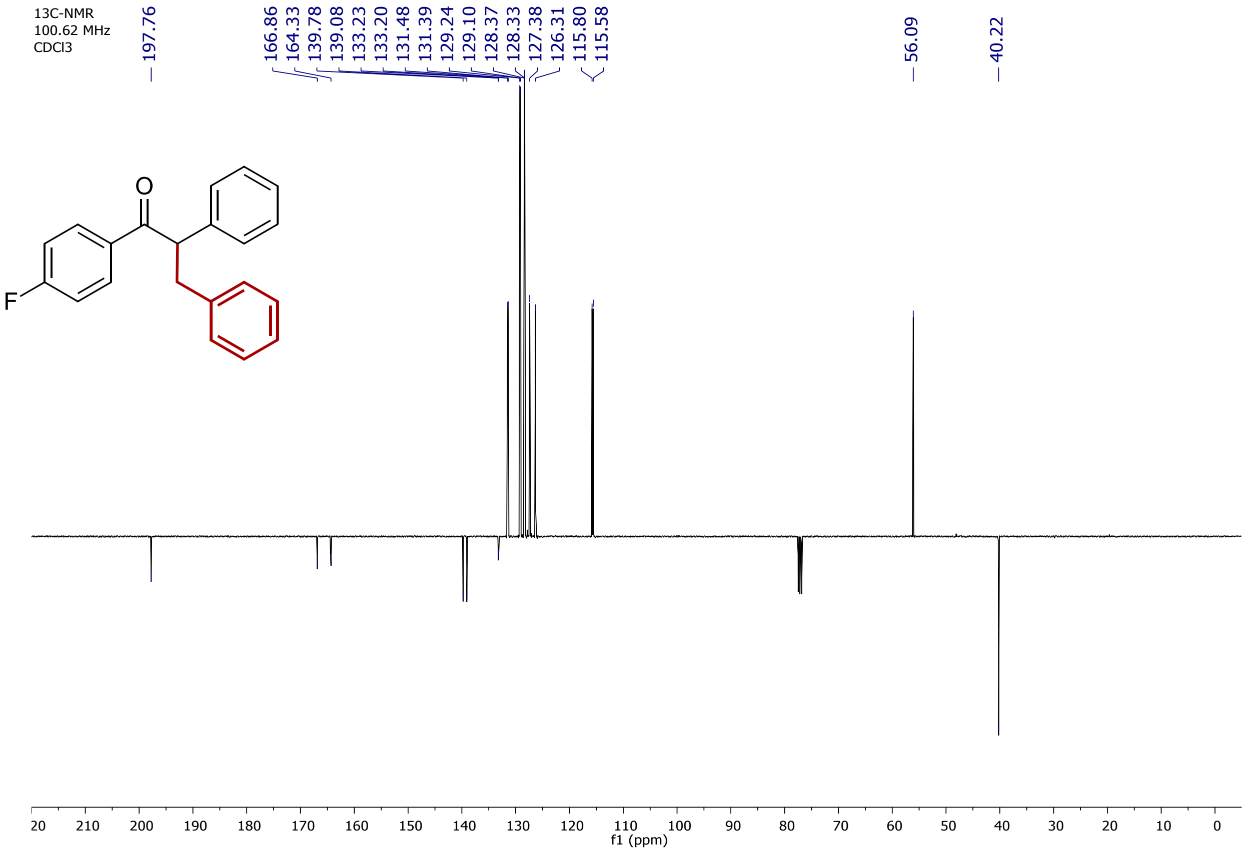

## 1,2-bis-(4-methoxy-phenyl)-3-phenyl-propan-1-one (5b)

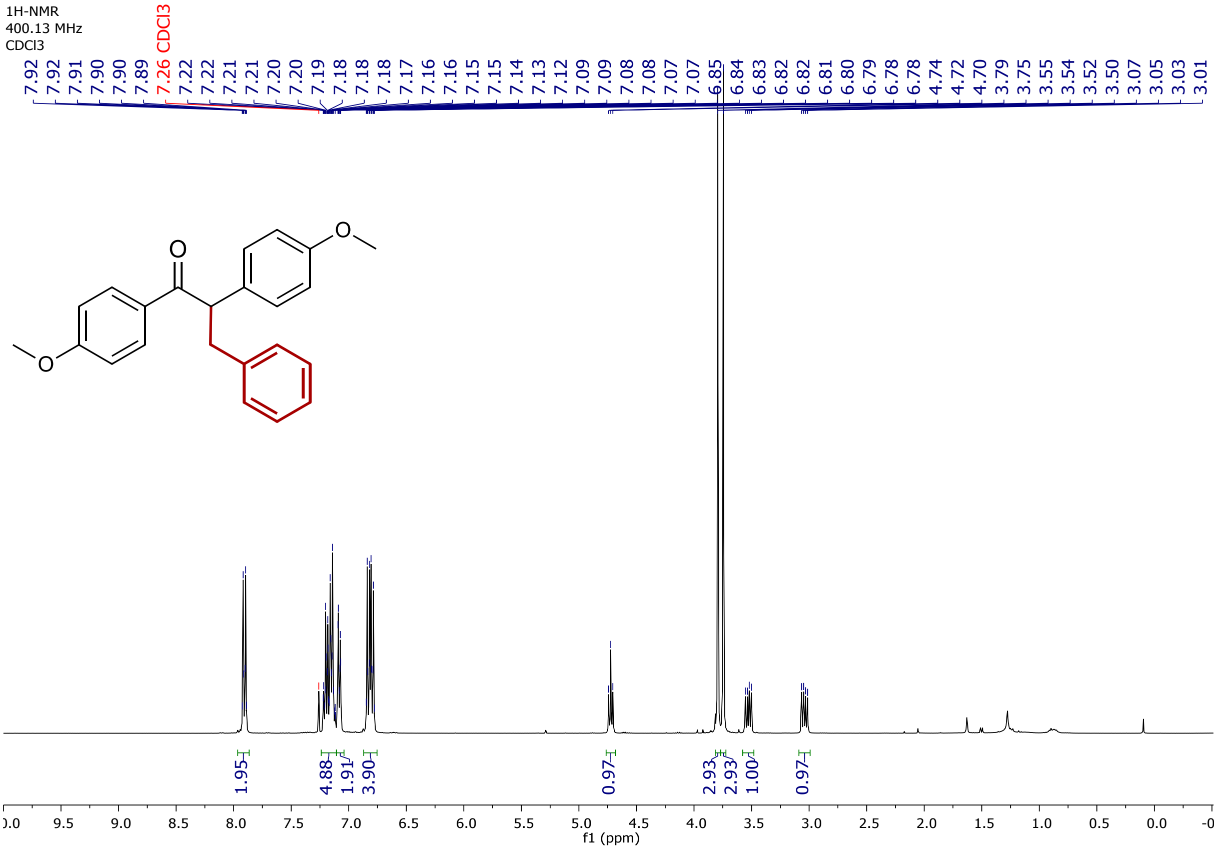


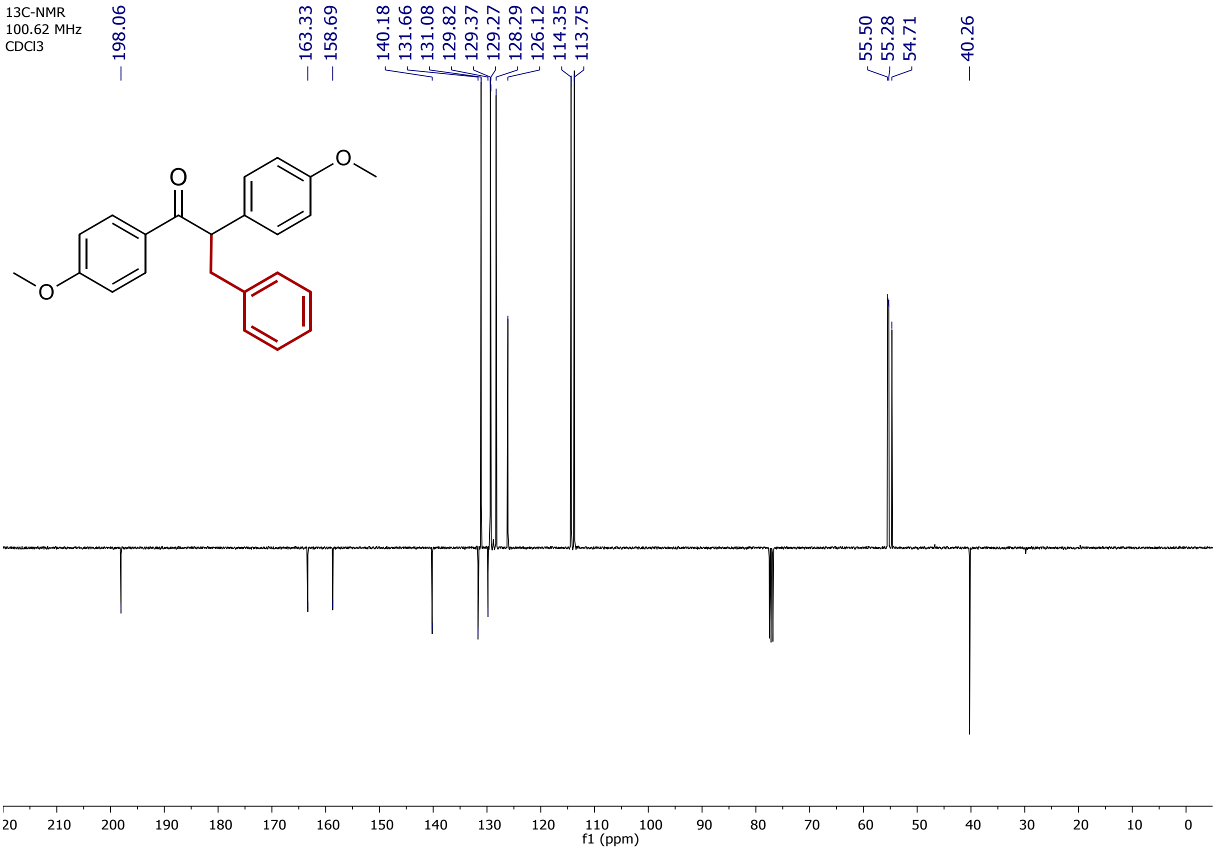

## 5-Methyl-1,2-diphenyl-3-hexanone (5c)

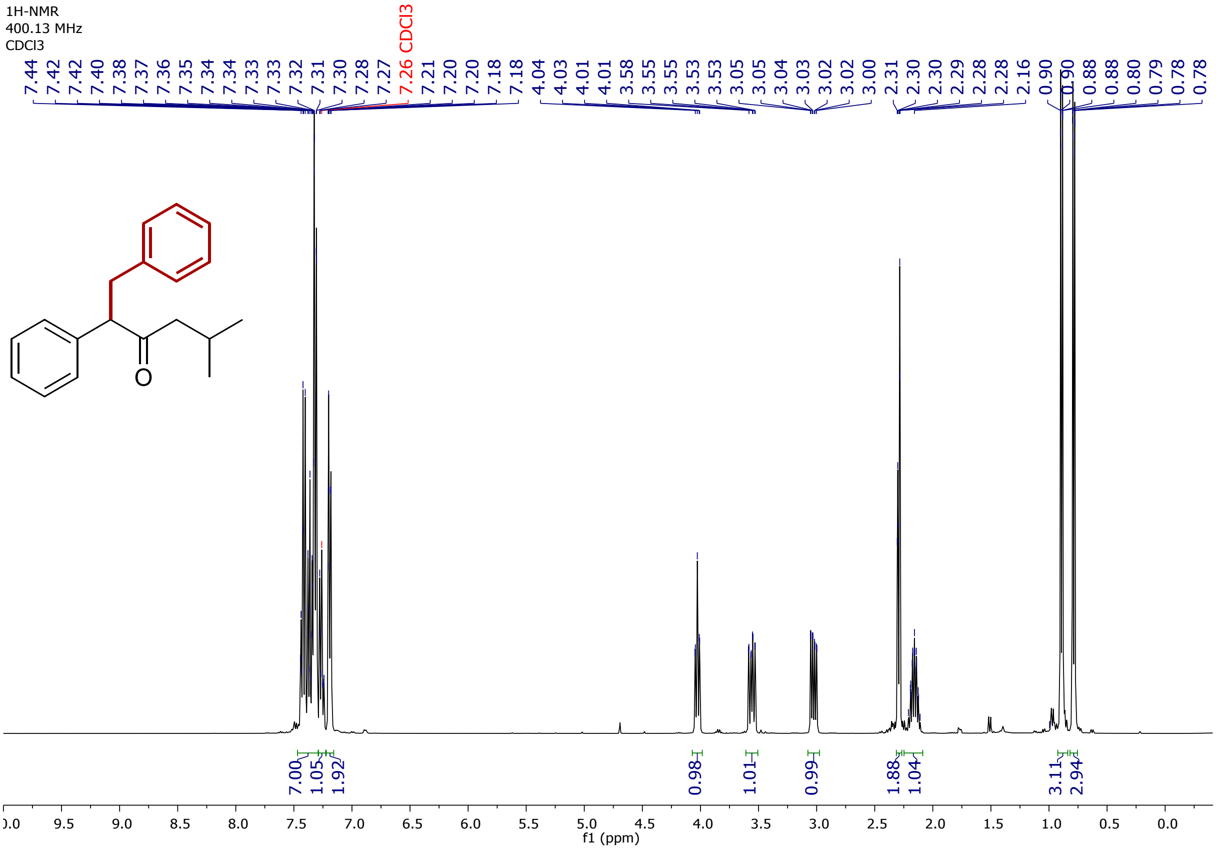


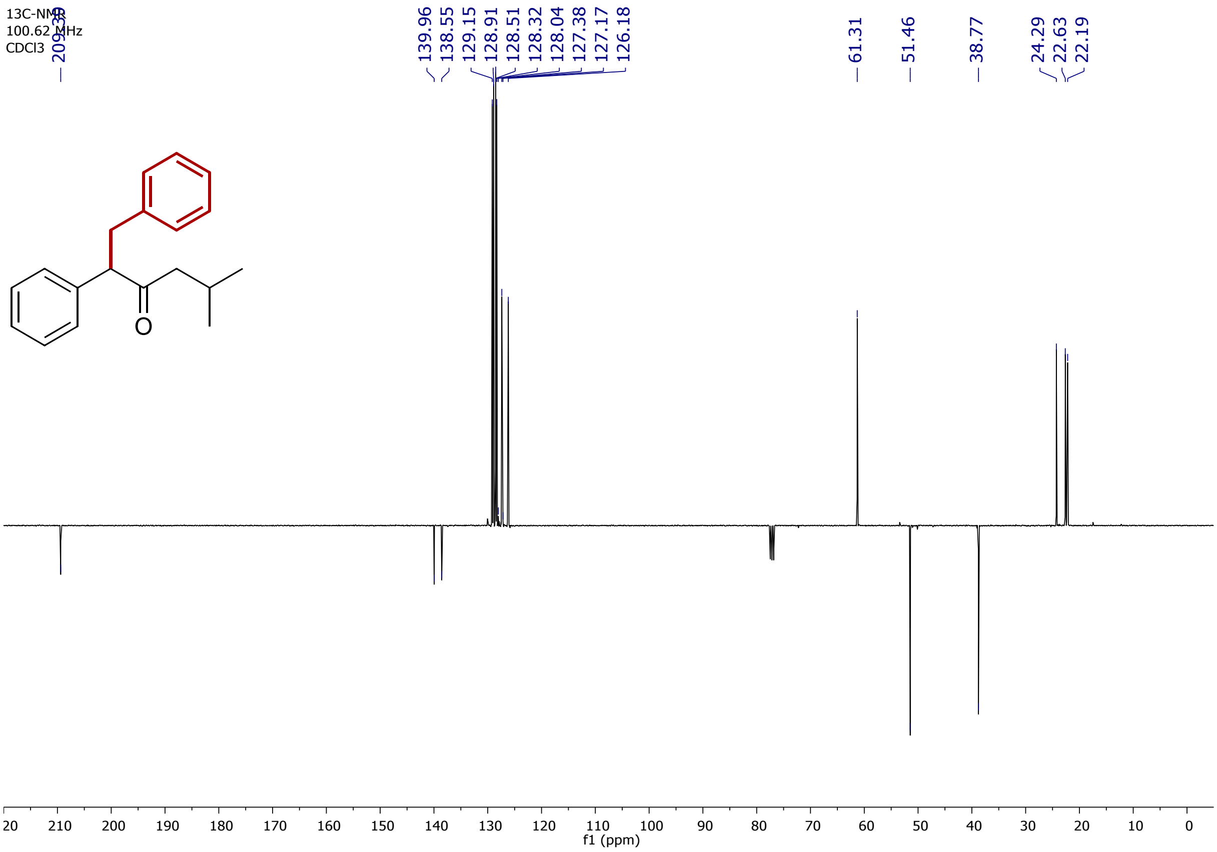

# References


(1) Mattes, A. O.; Russell, D.; Tishchenko, E.; Liu, Y.; Cichewicz, R. H.; Robinson, S. J. Application of 19F quantitative NMR to pharmaceutical analysis. *Concepts in Magnetic Resonance Part A* **2016,** *45A* (5), e21422.

(2) Mehta, V. P.; García-López, J.-A.; Greaney, M. F. Ruthenium-Catalyzed Cascade C�H Functionalization of Phenylacetophenones. *Angewandte Chemie International Edition* **2014,** *53* (6), 1529.

(3) Ding, Y.; Zhang, W.; Li, H.; Meng, Y.; Zhang, T.; Chen, Q.-Y.; Zhu, C. Metal-free synthesis of ketones by visible-light induced aerobic oxidative radical addition of aryl hydrazines to alkenes. *Green Chemistry* **2017,** *19* (13), 2941.

(4) Janeček, M.; Rossmann, M.; Sharma, P.; Emery, A.; Huggins, D. J.; Stockwell, S. R.; Stokes, J. E.; Tan, Y. S.; Almeida, E. G.; Hardwick, B.et al. Allosteric modulation of AURKA kinase activity by a small-molecule inhibitor of its protein-protein interaction with TPX2. *Scientific Reports* **2016,** *6* (1), 28528.

(5) Huang, K.; Li, G.; Huang, W.-P.; Yu, D.-G.; Shi, Z.-J. Arylation of α-pivaloxyl ketones with arylboronic reagents viaNi-catalyzed sp3 C–O activation. *Chemical Communications* **2011,** *47* (25), 7224.

(6) Shu, B.; Wang, X.-T.; Shen, Z.-X.; Che, T.; Zhong, M.; Song, J.-L.; Kang, H.-J.; Xie, H.; Zhang, L.; Zhang, S.-S. Iridium-catalyzed arylation of sulfoxonium ylides and arylboronic acids: a straightforward preparation of α-aryl ketones. *Organic Chemistry Frontiers* **2020,** *7* (14), 1802.

(7) He, C.; Guo, S.; Huang, L.; Lei, A. Copper Catalyzed Arylation/C−C Bond Activation: An Approach toward α-Aryl Ketones. *Journal of the American Chemical Society* **2010,** *132* (24), 8273.

(8) Battace, A.; Feuerstein, M.; Lemhadri, M.; Zair, T.; Doucet, H.; Santelli, M. Heck Reactions of α- or β-Substituted Enol Ethers with Aryl Bromides Catalysed by a Tetraphosphane/Palladium Complex – Direct Access to Acetophenone or 1-Arylpropanone Derivatives. *European Journal of Organic Chemistry* **2007,** *2007* (19), 3122.

(9) Lu, H.-Y.; Shen, A.; Li, Y.-Q.; Hu, Y.-C.; Ni, C.; Cao, Y.-C. N-heterocyclic carbene-palladium-imine complex catalyzed α-arylation of ketones with aryl and heteroaryl chlorides under air atmosphere. *Tetrahedron Letters* **2020,** *61* (29), 152124.

(10) Pichette Drapeau, M.; Fabre, I.; Grimaud, L.; Ciofini, I.; Ollevier, T.; Taillefer, M. Transition-Metal-Free α-Arylation of Enolizable Aryl Ketones and Mechanistic Evidence for a Radical Process. *Angewandte Chemie International Edition* **2015,** *54* (36), 10587.

(11) Peng, C.; Zhang, W.; Yan, G.; Wang, J. Arylation and Vinylation of α-Diazocarbonyl Compounds with Boroxines. *Organic Letters* **2009,** *11* (7), 1667.

(12) Artaud, I.; Ben-Aziza, K.; Mansuy, D. Iron porphyrin-catalyzed oxidation of 1, 2-dimethoxyarenes: a discussion of the different reactions involved and the competition between the formation of methoxyquinones or muconic dimethyl esters. *The Journal of Organic Chemistry* **1993,** *58* (12), 3373.

(13) Cherney, A. H.; Reisman, S. E. Pd-catalyzed Fukuyama cross-coupling of secondary organozinc reagents for the direct synthesis of unsymmetrical ketones. *Tetrahedron* **2014,** *70* (20), 3259.

(14) Yang, Y.; Wang, L.; Chen, Y.; Dai, Y.; Sun, Z. One-pot synthesis of α,α-disubstituted Aryl-1-ethanones via the Wittig-Horner reaction. *Phosphorus, Sulfur, and Silicon and the Related Elements* **2018,** *193* (3), 121.

(15) Buckland, S.; Halton, B.; Mei, Q.; Stang, P. Studies in the Cycloproparene Series: Reactions of Alkylidenecycloproparenes With Electrophiles. *Australian Journal of Chemistry* **1987,** *40* (8), 1375.

(16) Liu, F.; Hu, Y.-Y.; Li, D.; Zhou, Q.; Lu, J.-M. N-Heterocyclic carbene-palladacyclic complexes: synthesis, characterization and their applications in the C-N coupling and α-arylation of ketones using aryl chlorides. *Tetrahedron* **2018,** *74* (39), 5683.

(17) Lou, S.; Fu, G. C. Nickel/Bis(oxazoline)-Catalyzed Asymmetric Kumada Reactions of Alkyl Electrophiles: Cross-Couplings of Racemic α-Bromoketones. *Journal of the American Chemical Society* **2010,** *132* (4), 1264.

(18) Hashmi, A. S. K.; Wang, T.; Shi, S.; Rudolph, M. Regioselectivity Switch: Gold(I)-Catalyzed Oxidative Rearrangement of Propargyl Alcohols to 1,3-Diketones. *The Journal of Organic Chemistry* **2012,** *77* (17), 7761.

(19) Li, B. X.; Le, D. N.; Mack, K. A.; McClory, A.; Lim, N.-K.; Cravillion, T.; Savage, S.; Han, C.; Collum, D. B.; Zhang, H.et al. Highly Stereoselective Synthesis of Tetrasubstituted Acyclic All-Carbon Olefins via Enol Tosylation and Suzuki–Miyaura Coupling. *Journal of the American Chemical Society* **2017,** *139* (31), 10777.

(20) Cherney, A. H.; Kadunce, N. T.; Reisman, S. E. Catalytic Asymmetric Reductive Acyl Cross-Coupling: Synthesis of Enantioenriched Acyclic α,α-Disubstituted Ketones. *Journal of the American Chemical Society* **2013,** *135* (20), 7442.

(21) Ueda, Y.; Iwai, T.; Sawamura, M. Nickel-Copper-Catalyzed Hydroacylation of Vinylarenes with Acyl Fluorides and Hydrosilanes. *Chemistry – A European Journal* **2019,** *25* (40), 9410.

(22) Su, Y.; Sun, X.; Wu, G.; Jiao, N. Catalyst-Controlled Highly Selective Coupling and Oxygenation of Olefins: A Direct Approach to Alcohols, Ketones, and Diketones. *Angewandte Chemie International Edition* **2013,** *52* (37), 9808.

(23) Audubert, C.; Lebel, H. Mild Esterification of Carboxylic Acids via Continuous Flow Diazotization of Amines. *Organic Letters* **2017,** *19* (16), 4407.

(24) Waibel, M.; De Angelis, M.; Stossi, F.; Kieser, K. J.; Carlson, K. E.; Katzenellenbogen, B. S.; Katzenellenbogen, J. A. Bibenzyl- and stilbene-core compounds with non-polar linker atom substituents as selective ligands for estrogen receptor beta. *European Journal of Medicinal Chemistry* **2009,** *44* (9), 3412.

(25) Han, W.; Chen, J.; Jin, F.; Yuan, X. Iodide-Catalyzed Carbonylation–Benzylation of Benzyl Chlorides with Potassium Aryltrifluoroborates under Ambient Pressure of Carbon Monoxide. *Synlett* **2018,** *29* (03), 369.

(26) Liu, T.-L.; Ng, T. W.; Zhao, Y. Rhodium-Catalyzed Enantioselective Isomerization of Secondary Allylic Alcohols. *Journal of the American Chemical Society* **2017,** *139* (10), 3643.
